# A Mechanistic Model of PCR for Accurate Quantification of Quantitative PCR Data

**DOI:** 10.1371/journal.pone.0012355

**Published:** 2010-08-30

**Authors:** Gregory J. Boggy, Peter J. Woolf

**Affiliations:** Department of Chemical Engineering, University of Michigan, Ann Arbor, Michigan, United States of America; King Abdullah University of Science and Technology, Saudi Arabia

## Abstract

**Background:**

Quantitative PCR (qPCR) is a workhorse laboratory technique for measuring the concentration of a target DNA sequence with high accuracy over a wide dynamic range. The gold standard method for estimating DNA concentrations via qPCR is quantification cycle (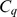) standard curve quantification, which requires the time- and labor-intensive construction of a 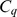 standard curve. In theory, the shape of a qPCR data curve can be used to directly quantify DNA concentration by fitting a model to data; however, current empirical model-based quantification methods are not as reliable as 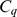 standard curve quantification.

**Principal Findings:**

We have developed a two-parameter mass action kinetic model of PCR (MAK2) that can be fitted to qPCR data in order to quantify target concentration from a single qPCR assay. To compare the accuracy of MAK2-fitting to other qPCR quantification methods, we have applied quantification methods to qPCR dilution series data generated in three independent laboratories using different target sequences. Quantification accuracy was assessed by analyzing the reliability of concentration predictions for targets at known concentrations. Our results indicate that quantification by MAK2-fitting is as reliable as 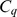 standard curve quantification for a variety of DNA targets and a wide range of concentrations.

**Significance:**

We anticipate that MAK2 quantification will have a profound effect on the way qPCR experiments are designed and analyzed. In particular, MAK2 enables accurate quantification of portable qPCR assays with limited sample throughput, where construction of a standard curve is impractical.

## Introduction

Biological assays to measure DNA and RNA concentrations are readily available in a laboratory environment, but are not yet available in a portable assay format suitable for use in home-based diagnostics or point-of-care diagnostics in resource poor settings. Three key difficulties in developing portable DNA and RNA assays are the size, complexity, and noise sensitivity intrinsic to these assays [1]. For example, currently available microarray assays require relatively large samples and complex sample preprocessing. Additionally, several replicate microarray assays are typically performed to compensate for the effects of experimental noise. Quantitative PCR (qPCR) based techniques represent an attractive option for portable DNA quantification as these assays are readily performed in microfluidic environments. However, the two most accurate qPCR approaches, quantification cycle (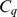) standard curve calibration [2] and digital PCR [3], each require multiple complex liquid-handling steps to generate and measure a series of diluted samples.

Ideally, a portable qPCR assay would only require measurements on a single undiluted sample. As has been suggested by others, the shape of a single qPCR amplification curve should be sufficient to uniquely determine initial DNA concentration in a sample [4]–[8]. In practice, however, the available single-assay qPCR analysis techniques have been less accurate than the gold standard technique of 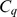 standard curve calibration [9].

Here we show that a 2-parameter mechanistic model of PCR, called MAK2 (for Mass Action Kinetic model with 2 parameters), quantifies DNA samples from a single qPCR assay as accurately as 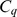 standard curve calibration, which requires multiple assays for quantification. Because MAK2 is a mechanistic model rather than an empirical model, quantifying qPCR data with MAK2 requires no assumptions about the amplification effiency of a qPCR assay. Furthermore, whereas 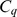 quantification uses a single datapoint in the qPCR curve for quantification, MAK2 is fitted to measurements across many amplification cycles, thereby reducing the influence of detection noise on estimates of DNA concentration.

## Results

### MAK2 models the exponential growth phase of PCR

**Figure 1:**
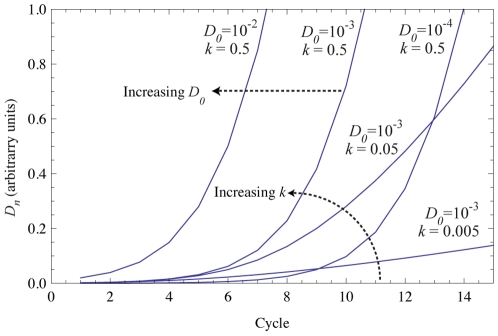
Simulated MAK2 curves with varying 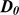 and 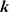 values. Curves are labeled with parameter values. Increasing 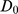 shifts the MAK2 curve to the left, while increasing 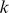 increases the slope of the MAK2 curve.

MAK2 describes the accumulation of amplicon DNA during PCR. The model is derived from reaction kinetics in the anneal/elongation steps of PCR, as is briefly discussed in *Materials and Methods* and detailed in the online supporting document, *Text S1*. MAK2 is expressed as:
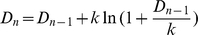
(1)where 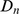 can represent either the amount of double-stranded DNA (dsDNA) after 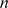 cycles of PCR or the fluorescence associated with dsDNA after 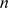 cycles of quantitative PCR. In equation (1), 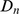 is recursively dependent on 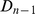, the amount of 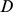 from the previous cycle. The characteristic PCR constant 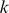 determines the rate of DNA accumulation during PCR. 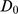, and 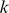 are the only two adjustable parameters that determine 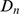 values at every PCR cycle. These parameters have distinct effects on the shape of the MAK2 curve; changing the value of 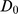 shifts the curve right or left while changing the value of 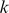 changes the slope of the curve, as shown in Fig. 1.

MAK2 can be used for fitting qPCR fluorescence data when 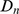 in equation (1) represents the fluorescence associated with dsDNA at cycle 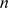. There is often a background fluorescence in qPCR data that is independent of signal associated with target. This background fluorescence is due to fluorescence produced by the reaction system itself (caused by plastics or reagents) [9]. In model-fitting approaches to quantifying qPCR data, the fluorescence is typically assumed to be composed of signal and a background fluorescence [5], [6], [8], [10], [11]. Similarly, for MAK2-fitting of qPCR data, fluorescence is background adjusted by the parameter, 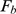 as follows:
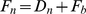
(2)where 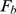 represents constant background fluorescence and 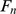 is the the MAK2-predicted fluorescence at cycle 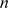, the variable used for fitting qPCR fluorescence data.

**Figure 2:**
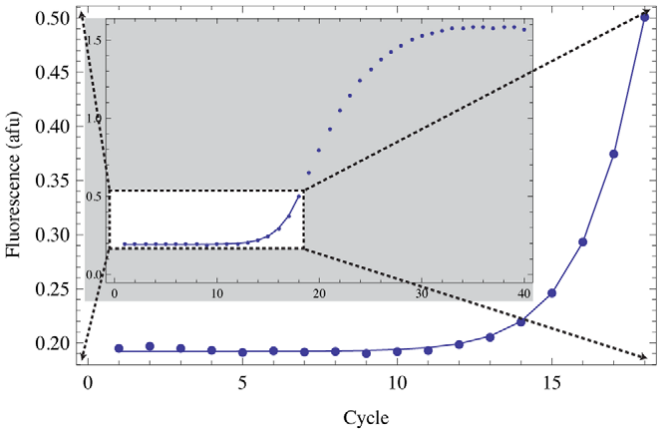
Optimized fit of MAK2 (solid line) to data (points). The gray inset depicts the full data range with the MAK2 fit overlaid. The large curve is a blown up view of the white box in the inset.

Due to assumptions made in deriving MAK2 (see *Materials and Methods* for a brief description or supporting *Text S1* for more detail), the model is applicable only to qPCR data obtained before primer depletion and enzyme saturation are significant effects. Therefore, in our use of MAK2, we have truncated the data to the cycle with the maximum slope increase, relative to the previous cycle (see *Materials and Methods* for more detail). Truncation of the data to be fitted is justified (indeed necessary) based on mechanistic considerations and not based on statistical classification of outliers as in some qPCR model-fitting methods [6], [8], [10]. The region of data over which MAK2 is applicable is often referred to as the exponential growth phase of PCR. An example of an optimized fit of MAK2 to qPCR data is shown in Fig. 2.

### MAK2 predicts declining amplification efficiency

PCR amplification efficiency is often used as a parameter for quantifying target DNA amount from qPCR data. Amplification efficiency is defined on a cycle-by-cycle basis [5] as:
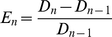
(3)where 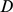 is fluorescence due to dsDNA. Applying the MAK2 expression (1) to the amplification efficiency expression (3) yields:
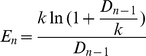
(4)From this expression, amplification efficiency is dependent on DNA concentration, though not linearly as has been previously proposed [8]. Furthermore, amplification efficiency monotonically decreases as DNA concentration increases, in contrast with the assumption that amplification efficiency is constant below the quantification threshold. This assumption of constant amplification efficiency has been the foundation for the development of 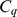 quantification methods such as the relative quantification method developed by Pfaffl [12]. In contrast to such quantification methods, quantification by 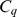 standard curve calibration is theoretically valid because it requires no assumptions about PCR mechanism.

### MAK2 fitting quantifies qPCR data as accurately as 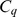 standard curve calibration

**Figure 3:**
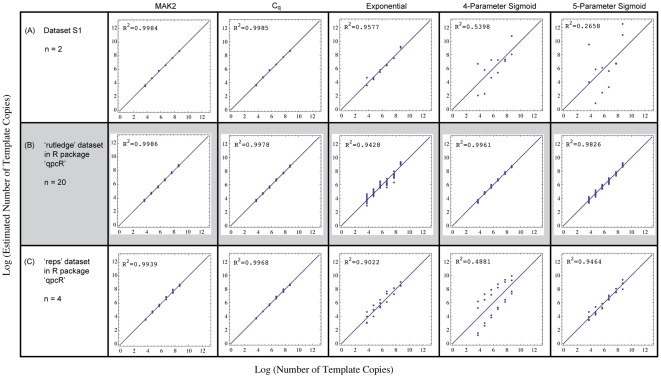
Assessment of quantification accuracy for five quantification methods on three independent datasets. Datasets (rows A–C with n = 2, n = 20, and n = 4 replicates per concentration, respectively) were quantified by five methods (in columns) as follows: MAK2: model-fitting with MAK2; 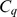: 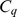 standard curve calibration; Exponential: exponential curve-fitting [4]; 4-Parameter Sigmoid: sigmoidal curve-fitting (SCF) with a 4-parameter log-logistic function [11]; 5-Parameter Sigmoid: SCF with a 5-parameter log-logistic function [11]. Each dataset is from a different target sequence diluted sequentially by ten-fold to obtain data from a concentration range of six orders of magnitude. Panels in the figure contain log-log plots of estimated vs. actual number of template molecules. The line at 45 in each plot represents the line of agreement between prediction and known amount. Rows are labeled with the source of the data. Dataset S1, from experiments performed by the authors, is published online.

To determine how accurately MAK2 fitting performs relative to other qPCR quantification methods, we analyzed three independently generated qPCR dilution series by MAK2 fitting, 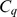 standard curve calibration, exponential curve fitting [4], and sigmoidal curve fitting with 4 and 5 parameter log-logistic functions [11]. The resulting log-log plots of estimated vs. known target amount are shown in the panels of Fig. 3. The first of the three datasets, shown in Fig. 3A, was generated by the authors as described in *Materials and Methods*. The other two datasets used for demonstrating MAK2 were chosen from datasets freely available to researchers in the *R* package *qpcR* [13]. These datasets were assumed to be representative of standard qPCR data because they are included as example datasets in the *qpcR* package for the purpose of demonstrating various model-fitting procedures for quantification of qPCR data.

The plots in Fig. 3 demonstrate the equivalent performance of MAK2 quantification and 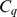 standard curve quantification, and the superior performance of these two methods relative to other model-fitting quantification methods. The third most accurate quantification method was different for each dilution set, indicating how variable the predictions made by these methods can be. Note that quantification by the 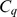 standard curve requires the entire dilution series, while estimates made by the other four quantification methods are based on single qPCR runs at each dilution.

## Discussion

We have demonstrated that fitting qPCR data with a 2-parameter mechanistic model of PCR, MAK2, quantifies single qPCR assays as reliably as 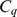 standard curve calibration for a variety of target sequences and a wide range of concentrations. In contrast, quantification by fitting qPCR data with an empirical model, such as an exponential curve or a sigmoidal curve, is not as reliable and accurate quantification is strongly dependent on PCR conditions used.

Empirical model-fitting methods, such as sigmoidal or exponential curve-fitting, fail to reliably quantify qPCR data because they are unable to accurately describe amplification efficiency in early cycles of qPCR where the fluorescence signal is dominated by noise. The model-predicted behavior in these early cycles depends on assumptions about amplification efficiency implicit in the model. For example, fitting qPCR data with an exponential curve implies that amplification efficiency observed in the log-linear region of the qPCR curve is constant through all early PCR cycles while fitting with a sigmoidal curve implies that early cycle amplification efficiency follows a sigmoidal trend. Because these assumptions are not consistent with the mechanism of PCR, empirical model predictions are less reliable than predictions made by mechanistic models such as MAK2.

The two parameters in MAK2, 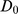 and 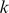, are sufficient to accurately describe complex PCR behavior for early cycles of qPCR, where effects such as primer depletion or polymerase saturation can be neglected. The initial target DNA concentration, 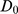, determines where the fluorescence signal rises above noise. The parameter 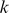, represents the ratio of primer binding and DNA reannealing rate constants and dictates how amplification efficiency changes at every cycle with increasing DNA concentration. While 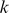 should theoretically remain constant for a given amplicon sequence and primer set, fitting with MAK2 revealed that this is not always the case (see supporting *Fig. S1*). The observed variation in 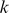 may indicate the presence of unexplained qPCR effects, but further study is needed to determine its significance.

MAK2 is the first mechanistic model of PCR suitable for quantifying qPCR data generated with either nonspecific dyes or specific probes. A mechanistic model of specific probe binding has been developed and used for quantifying qPCR data generated by hydrolysis probes [7]. Detailed mechanistic models of PCR have also been developed and used in simulating PCR [14], [15], however, these models contain many more parameters than MAK2 and attempting to use these models for fitting qPCR data results in data overfitting and non-unique solutions for key parameters such as 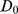. The three parameters used for fitting MAK2 to qPCR data (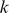; 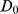; and background fluorescence, 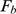) each affect the simulated MAK2 curve in orthogonal ways, so that fitting with MAK2 ensures a unique solution for the optimal parameter set.

The approach used in this work reflects a broader trend in systems biology of trading assay complexity for software complexity. As a well-known example, shotgun sequencing enables sequencing of large DNA segments using simplified experimental methods by shifting complexity to sequence reconstruction software. Similarly, the MAK2 approach enables accurate DNA quantification using significantly less complex experimental methods by carrying out a more complex, mechanistic software analysis. As a result, MAK2 provides a robust single assay method for DNA quantification, overcoming a significant hurdle in the development of a portable nucleic acid assay system.

## Materials and Methods

### Derivation of MAK2 from PCR mass action kinetics

MAK2, expressed as equation (1), results from applying a series of assumptions to an ideal PCR system. We define the ideal PCR system as one in which the following assumptions can be made:

1. Errors occurring during PCR can be neglected
2. The complementary DNA strands 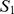 and 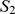 can be treated identically as 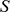

3. PCR primers for 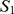 and 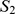, 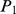 and 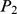 respectively, can be treated identically as 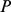

4. Off-target effects of PCR primers can be neglected
5. Thermally-induced degradation of DNA polymerase can be neglected
6. Strand elongation is considered as a single step, rather than as a series of single nucleotide additions
7. Reactions occurring during the anneal/elongation phases of PCR go to completion
8. All double-stranded DNA melts at the high temperature step of PCR

As a result of assumptions 1–6, the mass action kinetic model for the anneal/elongation phases of PCR in an ideal PCR system is:
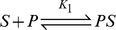
(5)

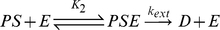
(6)

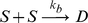
(7)where equation (5) describes equilibrium formation of the primer-strand complex (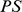), equation (6) describes the irreversible production of new DNA following the equilibrium complexation of DNA polymerase (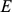) with 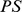, and equation (7) shows the rehybridization of complementary DNA strands that competes with production of new DNA.

We now assume that primer and DNA polymerase are in excess. This assumption is heretofore referred to as the “non-limiting assumption” because the amounts of primers and polymerase do not limit the rate of the elongation reaction. The polymerase and primer kinetic contributions to the model can thus be neglected and equations (5) and (6) are simplified to:
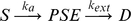
(8)


Because the kinetics of DNA polymerization are slow relative to the kinetics of 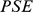 formation and DNA reannealing, it can be assumed that 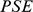 formation and DNA reannealing are competing reactions and that any 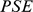 that forms is converted to dsDNA by the slow acting DNA polymerase. The final form of the mechanistic model, from which MAK2 is derived, is thus:
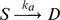
(9)

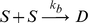
(10)where equations (9) and (10) describe the competition between a first-order reaction for strand synthesis and a second-order reaction for rehybridization, respectively.

Following the anneal/elongation phases of PCR, double-stranded DNA is melted to single-stranded DNA at the high temperature of PCR. As a result of assumptions 7 and 8, the transition between cycles at the high temperature step can be modeled as:
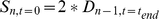
(11)where the single-stranded DNA at the beginning of cycle n is equal to double the amount of double-stranded DNA at the end of the previous cycle. Equation (11) thus allows the model output for a cycle to be fed in as an initial condition to model the next cycle.

A more detailed description of mass action kinetic assumptions and the full mathematical derivation of MAK2 are included in the online supporting document, *Text S1*.

### Justification of assumptions made in the derivation of MAK2

Following the development of any theoretical model of a process, the validity of the assumptions made in formulating that model must be analyzed in order to ensure that the foundation of the model is on solid ground. Here, we justify each assumption made in deriving MAK2, beginning with the non-limiting assumption which asserts that primers and polymerase are in excess and do not limit the rate of reaction.

The non-limiting assumption is valid for early cycles of PCR before target DNA concentrations rise to concentrations comparable to those of polymerase and primers. When DNA concentrations rise to the level of polymerase, the enzyme becomes saturated and cannot efficiently process new strands of DNA. When DNA concentrations rise to the level of primers, the forward process in equation (5) is no longer favored over the reverse process and the effects of changing primer concentration must be considered. A much more complex model is necessary for modeling PCR when primers and polymerase are limiting, because reaction kinetics change dynamically in response to changes in primer and polymerase concentration. The limiting effects of primers and polymerase contribute to late-cycle PCR behavior, such as the onset of the plateau phase of PCR where very little new DNA is generated. MAK2 is therefore only applicable to early cycles of PCR where limiting effects of primers and polymerase can be neglected.

As will become evident, the non-limiting assumption provides critical justification for all other assumptions made in the derivation of MAK2 except assumptions 1 and 8. While the validity of assumptions 2–7 coincides with validity of the non-limiting assumption, assumptions 1 and 8 are valid for all cycles of PCR.

#### Assumption 1: Errors occurring during PCR can be neglected

This assumption is valid when using a non-error prone polymerase. Most commercially-available DNA polymerases used for quantitative PCR have low rates of introducing wrong bases (errors) into DNA product. Error prone polymerases that introduce errors to DNA product (useful in methods such as directed evolution) should not be used for quantitative PCR.

#### Assumptions 2 and 3: PCR primers and target strands can be treated identically

These assumptions follow from the assumption that both primers are in excess (thus favoring 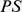 formation over 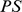 dissociation by Le Châtelier's principle) and the assumption that the forward rate for primer-substrate hybridization is independent of sequence (see references [14], [15]).

Secondary structure in target strands and primers may affect the dynamics of primer hybridization differently for each target strand, so that target strands act differently during the course of the reaction. Although secondary structure can hinder primer hybridization, the excess amount of primer will still drive primer and strand toward 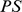 formation by Le Châtelier's principle. Given the assumption that all reactions go to completion (assumption 7, which follows from the non-limiting assumption), all single-stranded DNA will end up as double-stranded DNA at the end of the cycle, and both target strands can therefore be treated identically at the end of each cycle, which is the time-point modeled by MAK2.

#### Assumptions 4 and 5: Primer off-target effects and polymerase degradation can be neglected

These assumptions follow from the non-limiting assumption. If primers are in excess, removal of free primer by off-target hybridization will not have a noticeable effect on the reaction dynamics. Likewise, if polymerase is in excess, a small amount of thermally-induced degradation will not have a noticeable effect on reaction dynamics.

#### Assumption 6: Strand elongation can be considered as a single step

This assumption follows from the assumption that all reactions go to completion (assumption 7, which follows from the non-limiting assumption). If the elongation process goes to completion, there are no partially elongated strands remaining at the end of the elongation step of PCR. Therefore, it is unnecessary to treat elongation as the series of single nucleotide additions that it is in reality, and elongation can be approximated as a single step.

#### Assumption 7: Reactions occuring in the anneal/elongation phases go to completion

This assumption follows from the non-limiting assumption because when primers are in excess, any single-stranded DNA that does not reanneal to form dsDNA will form 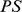 through primer hybridization (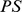 formation is favored over 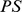 dissociation by Le Châtelier's principle); and because the polymerase is not saturated with 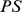 substrate, it is able to complete the elongation reaction during the elongation phase of PCR. Because the elongation reaction is the rate-limiting step in the production of a new strand of DNA, all other reactions can be assumed to go to completion.

#### Assumption 8: All double-stranded DNA melts at the high temperature step of PCR

This assumption allows the starting amount of ssDNA for cycle n to be related to the amount of dsDNA after cycle n-1, providing the link between consecutive cycles. This assumption is valid when the high temperature step of PCR incubates the reaction at a temperature much higher than the melting temperature of the target DNA for a sufficient amount of time. Using the protocol for the high temperature step suggested by the polymerase manufacturer is likely sufficient for this assumption to be valid.

#### Practical implications of the non-limiting assumption for PCR analysis

One consequence of the non-limiting assumption is that the actual concentrations of primer and polymerase are irrelevant to quantification by MAK2. This attribute of MAK2 is beneficial because enzyme manufacturers typically provide polymerase concentrations in terms of arbitrary units instead of SI units, so that modeling concentration dependent behavior of polymerase can be difficult.

Another consequence of the non-limiting assumption is that MAK2 is applicable to fitting a limited amount of qPCR data. The slope of a qPCR curve initially increases with each cycle until an inflection point is reached, at which point the slope gradually decreases until it is flat. MAK2, on the other hand, predicts that the slope of the qPCR curve increases constantly. This can be seen if equation (1) is rewritten as:
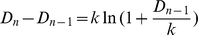
(12)to obtain the first-derivative of 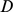 with respect to cycle. The expression on the right-hand side increases monotonically with increasing values of 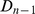. Because MAK2 does not predict an inflection point in the qPCR curve, it is no longer an accurate model when the inflection point is reached in qPCR data. Analysis of qPCR data reveals that the inflection point is reached soon after the maximum slope increase occurs. Thus, we have used the cycle with the maximum slope increase, relative to the previous cycle, as the cutoff point for MAK2-fitting. Experimenting with various cutoff cycles has indicated that setting the cutoff one or two cycles above or below this cycle does not significantly affect MAK2 concentration predictions.

### Quantitative PCR data

#### qPCR assays

Quantitative PCR assays shown in Fig. 3A were performed by the authors in 25 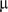
L samples on an MJ Research (BioRad) Chromo4 thermal cycler. Reaction buffer was composed of 0.1 units/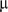
L HotStart Paq5000 DNA Polymerase (Stratagene, La Jolla, CA) in the supplied reaction buffer, 0.2 mM of each dNTP (Promega, Madison, WI), 2 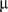
M of the dsDNA dye SYTO-13 (Invitrogen, Carlsbad, CA) and 400 nM of each primer. The inital DNA concentration used in these qPCR dilution series experiments ranged from 5*10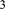 to 5*10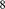 copies per well in 10-fold increments. Assays for each concentration were run in duplicate.

The thermal cycling protocol contained a two-minute incubation period at 95.0

C followed by forty cycles with a 20s incubation at 95.0

C and a 60s incubation at 64.0

C with 4 plate reads obtained at 15s intervals. A melt profile was obtained after the 11th cycle and again after every third cycle thereafter (for a total of 10 melt profiles). The melt profile consisted of plate reads obtained after a 5s incubation at temperatures ranging from 79.0 to 83.8

C in 0.2

C increments, and reads at 84.0, 84.5, and 85.0

C obtained after a 10s incubation.

The target DNA was a synthetic sequence designed by generating a random sequence and minimizing secondary structure and off-target primer binding by modifying the sequence. Secondary structure and off-target primer binding were identified and their thermodynamic properties were calculated using Visual OMP software from DNA Software (Ann Arbor, MI). Primer and target DNA were obtained from Integrated DNA Technologies (Coralville, IA). Primer and target DNA sequences are published in online supporting data as *Table S1*. Raw data are also provided as supporting data, *Dataset S1*.

#### Independent qPCR dilution data sets

In addition to the dataset generated as described above, two additional data sets were used in the comparison of quantification methods shown in Fig. 3. These datasets were obtained from the *rutledge* (row B in Fig. 3) and *reps* (row C in Fig. 3) datasets in the *R* package *qpcR* [13]. The *rutledge* dataset is from Supplemental Data 1 of [6] and contains data from six 10-fold dilutions of a 102-bp sequence generated in five independent experiments with four replicates each.

The *reps* dataset is an unpublished dataset that contains seven 10-fold dilutions of an S27a housekeeping gene target, with four replicates each. Quantification of the most dilute condition of the *reps* dataset was not used for comparison because inclusion significantly affected R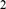 values obtained for the three methods that most accurately quantified this data. The values plotted in Fig. 3 for the *rutledge* and *reps* datasets are relative values, scaled for comparison to our data, generated as described above.

### Quantification of qPCR data

The quantification plots in Fig. 3 depict the accuracy of quantification by the various methods. To generate these plots, quantification metrics 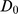 or 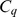 were generated as described in the sections below. Next, the best fit linear relationship between 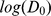 and 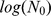 (where 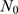 is the initial amount of target DNA) or between 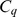 and 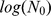 was found by linear model-fitting (function *LinearModelFit*) in Mathematica. Finally, the trend equation was then used to calculate an estimated 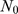 for each known 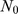. The plots in Fig. 3 are log-log plots of estimated vs. known 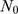.

#### MAK2 model-fitting

The parameters in the MAK2 model were fit using custom developed Mathematica code in online supporting data, *Dataset S2*. This dataset also contains results of fitting qPCR data with MAK2. The 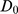 values obtained were used in generating plots for MAK2 quantification shown in Fig. 3.

The sum of squared residuals was used as a cost function for optimization. Each iteration of optimization tested values for parameters 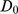, 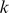, and 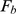 by performing a simulation of MAK2 with these values and calculating the associated cost function value. Parameter values resulting in the minimum cost function value found in 5000 iterations of Nelder-Mead optimization were considered the correct parameter set. Additional optimization iterations yielded no significant improvement in data fit.

The data included for optimization was truncated to the cycle with the maximum slope increase, relative to the previous cycle. Values for slope (equivalent to the first derivative with respect to cycle) were obtained by subtracting fluorescence at the previous cycle from the current fluorescence. Values for slope increase (equivalent to the second derivative with respect to cycle) were obtained by subtracting the previous cycle's slope value from the current cycle's slope value.

#### Quantification cycle (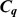) determination

To generate 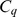 values, first a quantification threshold was chosen that represented about 10% of the maximum signal achieved in a dataset (0.1 for our data, 0.05 for *rutledge* data and 1 for *reps* data). Background intensity was determined as described above for determining data to include in MAK2 model-fitting. The 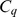 was calculated as the fractional cycle (linearly interpolated) where (intensity - background intensity) was equal to the quantification threshold.

Code for calculating 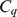 and results are published online as supporting data, *Dataset S3*. The 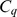 values in Dataset S3 were used in generating plots for 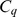 quantification in Fig. 3.

#### Exponential model-fitting

The exponential function for fitting qPCR data is:
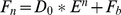
(13)where 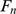 is the fluorescence intensity at cycle 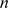, 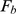 is background fluorescence, 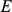 is the constant amplification efficiency of the reaction, and 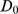 is the initial fluorescence.

Data were fit with equation (13) using nonlinear model-fitting (*NonlinearModelFit* function) in Mathematica. The data used for fitting was the minimum amount of data (beginning with cycle 1) that resulted in a nonlinear fit of the data. Results are published online as supporting data, *Dataset S4*. The 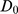 values in *Dataset S4* were used in generating plots for quantification by exponential-fitting in Fig. 3.

#### Fitting with log-logistic models

The equation for the five-parameter log-logistic function is:
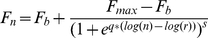
(14)where 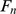, 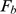, and 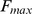 are the fluorescence at cycle 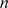, background fluorescence, and maximum fluorescence, respectively; and parameters 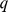, 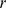, and 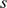 adjust the shape of the curve. The logistic model is identical to the log-logistic model in equation (14) except the 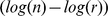 term is replaced by 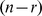. Parameter 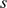 in equation (14) accounts for asymmetry in qPCR data and the four-parameter model is a special case of the five-parameter model, where 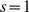. The first reported sigmoidal model for quantifying qPCR data [5] was a 4-parameter logistic model. Spiess et al. found that log-logistic models often perform better at data-fitting than logistic models [11], so 4 and 5-parameter log-logistic functions were used in our comparison of quantification methods.

Fitting data with four and five-parameter log-logistic functions was performed in the *R* package *qpcR*. The function *pcrbatch* was used for batch fitting an entire dataset and the value for *sig.init2* was used for estimating the initial fluorescence for each run. This estimate is generated by fitting qPCR data with the log-logistic model and then fitting the log-logistic model with the exponential model in (13) to find 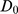.

## Supporting Information

Text S1 Derivation of MAK2.
(0.26 MB PDF)

Figure S1 Dependence of k on D_0_ for the three datasets used. The plots show k vs. log(D_0_), for the three different datasets, following optimization of MAK2 to the data.
(0.21 MB TIF)

Table S1 A table of sequences used for the qPCR experiments performed in the authors' lab.
(0.01 MB XLS)

Dataset S1 Original dilution series data from the authors' lab.
(0.03 MB XLS)

Dataset S2 Data resulting from fitting raw qPCR data with MAK2. D_0_ values obtained were used in generating figure 3.
(1.33 MB PDF)

Dataset S3 C_q_ values obtained for the qPCR data. These C_q_ values were used in generating figure 3.
(0.11 MB PDF)

Dataset S4 Data resulting from fitting raw qPCR data with an exponential model. D_0_ values obtained were used in generating figure 3.
(1.38 MB PDF)
