# Supplementary material for: A Mechanistic Model of PCR for Accurate Quantification of Quantitative PCR Data
Source: PLoS One. 2010 Aug 30;5(8):e12355. doi: 10.1371/journal.pone.0012355 (PMC2930010; doi:10.1371/journal.pone.0012355)
Supplement: Text S1 — Derivation of MAK2. (0.26 MB PDF) [file pone.0012355.s001.pdf]

Supporting Text

Simplified Mass Action Kinetics of PCR

The Polymerase Chain Reaction (PCR) is a commonly used method in biotechnology for amplifying DNA using a thermostable DNA polymerase. Quantitative PCR (qPCR) is merely PCR performed with a dye that indicates the concentration of DNA in real-time. The typical three-step PCR cycle is shown in figure 1.

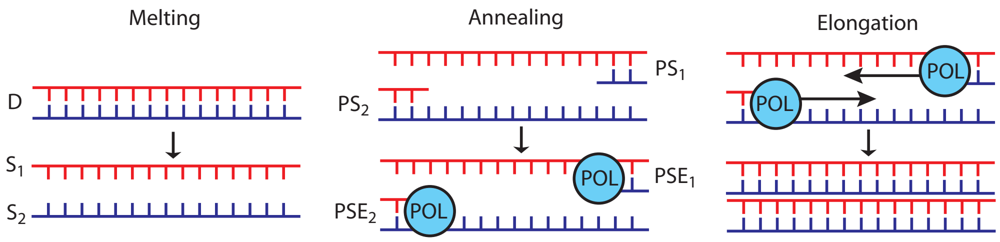

Figure 1: The PCR cycle. During the melting step, double-stranded DNA (D) melts to single strands S_1 and S_2. During the annealing step, primers P_1 and P_2 anneal to S_1 and S_2 to form primer-strand complexes PS_1 and PS_2. DNA polymerase (POL) also complexes with PS_1 and PS_2 during the annealing step to form primer-strand-enzyme complexes PSE_1 and PSE_2. During elongation, DNA polymerase extends primers into a new DNA strand, using the long strand as a template.

The mechanistic model we have developed for fitting qPCR data is derived from the chemical kinetics involved in the production of double-stranded DNA during the annealing and elongation steps of a PCR protocol. These steps of facilitate all reactions involved in the production of double-stranded DNA from single-stranded DNA, as shown below:

$$S_1 + P_1 \underset{k_{-11}}{\overset{k_{11}}{\rightleftharpoons}} PS_1 \qquad S_2 + P_2 \underset{k_{-12}}{\overset{k_{12}}{\rightleftharpoons}} PS_2 \tag{1}$$

$$PS_1 + E \underset{k_{-2}}{\overset{k_2}{\rightleftharpoons}} PSE_1 \xrightarrow{k_{ext}} D + E \qquad PS_1 + E \underset{k_{-2}}{\overset{k_2}{\rightleftharpoons}} PSE_1 \xrightarrow{k_{ext}} D + E \tag{2}$$

$$S_1 + S_2 \xrightarrow{k_b} D \tag{3}$$

where S_1 and S_2 are the two single-strands of DNA, P_1 and P_2 are their associated primers, E is DNA polymerase, PS_1 and PS_2 are primer-strand complexes, PSE_1 and PSE_2 are primer-strand-enzyme complexes, and D is double-stranded DNA. The model depicted above is a simplified version of previously proposed mechanistic models of PCR [1, 2]. Simulating many cycles of this model, (with complete melting of double-stranded DNA at each cycle), results in a curve with the characteristic sigmoidal shape of qPCR data. In such simulations, reaction-efficiency steadily declines due to the competition of the reannealing reaction (3) with the primer hybridization reaction (1), and the plateau-phase is brought on by depletion of primer. Attempting to fit qPCR data with this model, however, results in overfitting the

data because the model contains too many kinetic rate constants to be fitted. Thus, it has been very difficult to meaningfully fit qPCR data with a mechanistic model of PCR.

We have employed many simplifying assumptions to arrive at a simple model that captures the essential dynamics of PCR. The first simplifying assumption used is that the two primers and the two complementary DNA strands can be treated identically. The reaction thus simplifies to:

$$S + P \underset{k_{-1}}{\overset{k_1}{\rightleftharpoons}} PS \tag{4}$$

$$PS + E \underset{k_{-2}}{\overset{k_2}{\rightleftharpoons}} PSE \xrightarrow{k_{ext}} D + E \tag{5}$$

$$S + S \xrightarrow{k_b} D \tag{6}$$

Here, a limitation is imposed on the model that qPCR data to be fitted is restricted to data obtained before DNA concentration builds up to the level of primer concentration. This restriction justifies the assumption that primer and enzyme are in great excess and that changes in their concentration are minimal and do not affect the dynamics of the reaction during a cycle. Thus, concentration of enzyme and primer do not need to be considered in the production of the PSE complex and this process can be treated as first-order in strand concentration, resulting in:

$$S \xrightarrow{k_a} PSE \xrightarrow{k_{ext}} D \tag{7}$$

$$S + S \xrightarrow{k_b} D \tag{8}$$

The kinetics of the elongation step are slow relative to the kinetics of PSE complex formation and of reannealing. It can therefore be assumed that PSE complex formation competes with strand reannealing, but that any PSE complex that forms is converted to DNA by the slow action of DNA polymerase. The final form of the reaction is thus:

$$S \xrightarrow{k_a} D \tag{9}$$

$$S + S \xrightarrow{k_b} D \tag{10}$$

Derivation of MAK2

By simplifying the model of PCR to one with only two species, S and D, the mathematical representation of the model contains only two differential equations that can be solved analytically:

$$S' = \frac{dS}{dt} = -k_a S - k_b S^2, \quad S(0) = S_0 \tag{11}$$

$$D' = \frac{dD}{dt} = k_a S + \frac{1}{2} k_b S^2, \quad D(0) = 0 \tag{12}$$

To solve for $S(t)$ in (11), we first recognize the equation as a Bernoulli equation and divide both sides by S^2 and rearrange:

$$\frac{S'}{S^2} + k_a S^{-1} = -k_b \tag{13}$$

We now define variable v as:

$$v = S^{-1}, \quad v' = -\frac{S'}{S^2} \tag{14}$$

Substituting v for S, we obtain a linear differential equation in v:

$$-v' + k_a v = -k_b \tag{15}$$

Multiplying both sides by $-e^{-k_a t}$, we obtain:

$$v' e^{-k_a t} - k_a v e^{-k_a t} = k_b e^{-k_a t} \tag{16}$$

Recognizing that $(ve^{-k_a t})' = v' e^{-k_a t} - k_a v e^{-k_a t}$, we obtain:

$$(ve^{-k_a t})' = k_b e^{-k_a t} \tag{17}$$

Integrating (17) yields:

$$ve^{-k_a t} = -\frac{k_b}{k_a} e^{-k_a t} + c \tag{18}$$

Multiplying by $e^{k_a t}$ yields:

$$v = -\frac{k_b}{k_a} + ce^{k_a t} \tag{19}$$

Substituting back for $S(t)$ yields:

$$S(t) = (-\frac{k_b}{k_a} + ce^{k_a t})^{-1} \tag{20}$$

Applying the initial boundary condition, $S(0) = S_0$ yields:

$$S(0) = S_0 = (-\frac{k_b}{k_a} + c)^{-1} \tag{21}$$

Solving for c yields:

$$c = \frac{1}{S_0} + \frac{k_b}{k_a} \tag{22}$$

Plugging (22) into (20), we obtain the final formula for $S(t)$:

$$S(t) = \frac{k_a S_0 e^{-k_a t}}{k_a + k_b S_0 - k_b S_0 e^{-k_a t}} \tag{23}$$

We now attempt to solve for $D(t)$. We begin by rearranging (11) to obtain:

$$k_b S^2 = -S' - k_a S \tag{24}$$

Plugging (24) into (12) we obtain:

$$D' = k_a S + \frac{1}{2}(-S' - k_a S) = \frac{1}{2}(k_a S - S') \tag{25}$$

Integrating yields:

$$D(t) = \frac{k_a}{2} \int_0^t S(\tau)\, d\tau - \frac{1}{2}(S(t) - S_0) \tag{26}$$

Plugging (23) in for S yields the following expression:

$$\int_0^t S(\tau)\, d\tau = \int_0^t \frac{k_a S_0 e^{-k_a \tau}}{k_a + k_b S_0 - k_b S_0 e^{-k_a \tau}}\, d\tau \tag{27}$$

We now define variable u as:

$$u = k_a + k_b S_0 - k_b S_0 e^{-k_a \tau} \qquad du = k_a k_b S_0 e^{-k_a \tau}\, d\tau \tag{28}$$

Substituting u into (27) yields:

$$\frac{1}{k_b} \int_{u(0)}^{u(t)} \frac{du}{u} = \int_0^t S(\tau)\, d\tau = \frac{1}{k_b} \ln(u) \Big|_{u(0)}^{u(t)} \tag{29}$$

Then:

$$\int_0^t S(\tau)\, d\tau = \frac{1}{k_b} \ln(\frac{k_a + k_b S_0 - k_b S_0 e^{-k_a t}}{k_a}) \tag{30}$$

Plugging (30) into (26) yields:

$$D(t) = \frac{k_a}{2k_b} \ln(\frac{k_a + k_b S_0 - k_b S_0 e^{-k_a t}}{k_a}) - \frac{1}{2}(S(t) - S_0) \tag{31}$$

The equations up to this point have been for following the changes in concentration of single- and double-stranded DNA during a single cycle. Assuming that reactions (9) and (10) go to completion, an expression is obtained for double-stranded DNA at the end of any cycle, n:

$$D_n = \lim_{t \to \infty} D(t) = \frac{1}{2}(S_0 + \frac{k_a \ln(1 + \frac{k_b S_0}{k_a})}{k_b}) \tag{32}$$

Assuming that all double-stranded DNA melts to single-stranded DNA during the high-temperature step of PCR, S_0 can be set to $2D_{n-1}$, resulting in:

$$D_n = D_{n-1} + \frac{k_a \ln(1 + \frac{2k_b D_{n-1}}{k_a})}{2k_b} \tag{33}$$

The final expression for the model is obtained by substituting a constant, k, for the ratio $\frac{k_a}{2k_b}$ to obtain:

$$D_n = D_{n-1} + k \ln(1 + \frac{D_{n-1}}{k}) \tag{34}$$

The final expression, (34), is a recursive model in which the concentration of double-stranded DNA at the end of any cycle is dependent only on the amount of double-stranded DNA at the end of the previous cycle and the value of the constant k, a parameter that characterizes the dynamics of the PCR reaction. This is the model, MAK2, used to model PCR.

References

[1] J. L. Gevertz, S. M. Dunn, and C. M. Roth. Mathematical model of real-time pcr kinetics. *BIOTECHNOLOGY AND BIOENGINEERING*, 92(3):346–355, Nov 2005.

[2] S. Mehra and W. S. Hu. A kinetic model of quantitative real-time polymerase chain reaction. *BIOTECHNOLOGY AND BIOENGINEERING*, 91(7):848–860, Sep 2005.
