# Supplementary figures and images for: A Mechanistic Model of PCR for Accurate Quantification of Quantitative PCR Data
Source: PLoS One. 2010 Aug 30;5(8):e12355. doi: 10.1371/journal.pone.0012355 (PMC2930010; doi:10.1371/journal.pone.0012355)

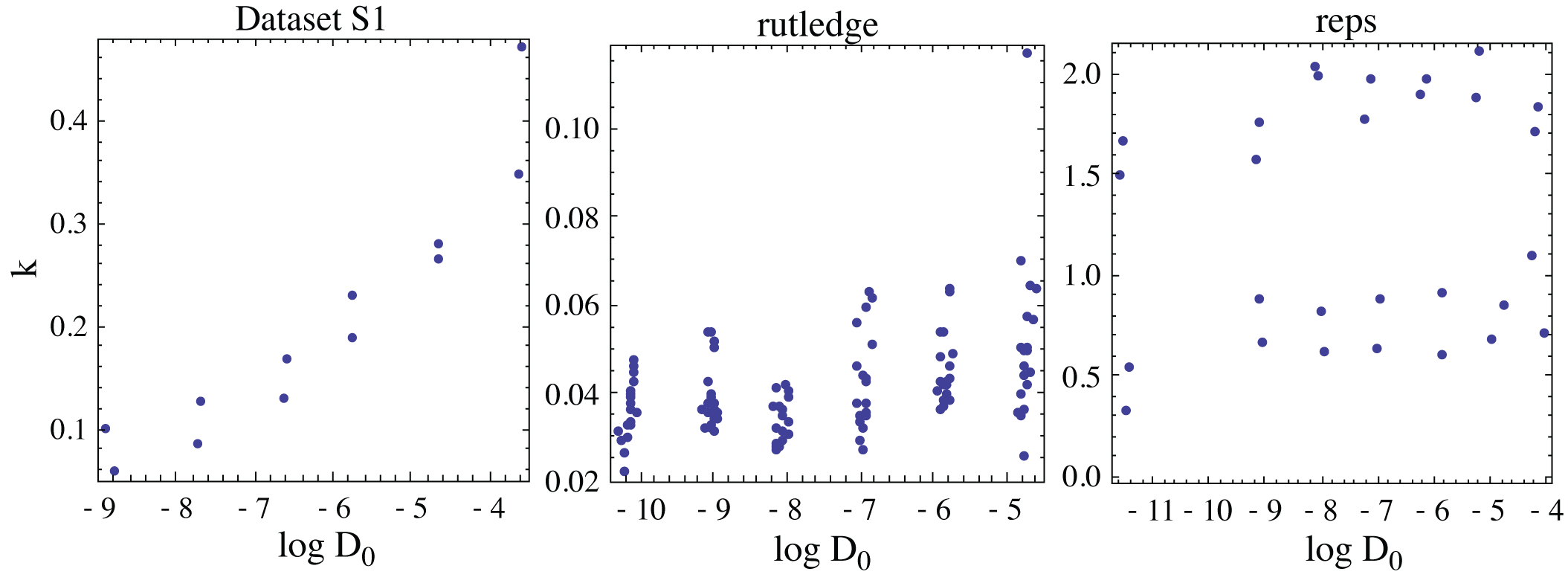

Supplement: Figure S1 — Dependence of k on D0 for the three datasets used. The plots show k vs. log(D0), for the three different datasets, following optimization of MAK2 to the data. (0.21 MB TIF) [file pone.0012355.s002.tif]
