# Supplementary material for: A Mechanistic Model of PCR for Accurate Quantification of Quantitative PCR Data
Source: PLoS One. 2010 Aug 30;5(8):e12355. doi: 10.1371/journal.pone.0012355 (PMC2930010; doi:10.1371/journal.pone.0012355)
Supplement: Dataset S2 — Data resulting from fitting raw qPCR data with MAK2. D0 values obtained were used in generating figure 3. (1.33 MB PDF) [file pone.0012355.s005.pdf]

```
(*************************************************************************************)
(*  This Mathematica notebook contains code written for fitting qPCR data with MAK2, the mechanistic 2   *)
(*  parameter model derived from the mass action kinetics of PCR and trials of fitting qPCR data with          *)
(*  MAK2 curves.                                                                                                                                   *)
(*                                                                                                                                                       *)
(*  The 'd0' values resulting from the trials are used to develop the plots in figure 3 of the MAK2 paper.        *)
(*                                                                                                                                                       *)
(******  Written by Gregory Boggy (November 2009) , based on optimization code written Avi Mayo *****)
(**************************************************************************************)
```

dataCTfunc is a function to define the data to be fitted. The variable 'data' is the name of the variable to be evaluated. The variable 'frac' defines the fraction of the maximum signal the threshold for excluding data is set. Determination of the data to fit is described in Materials and Methods of the MAK2 article.

```
dataCTfunc[data_] :=
  {
   b = Abs[data[[1, 2]]] + 0.0001;
   datadiff = {};
   For[i = 1, i < Length[data], i++;
    diff = data[[i, 2]] - data[[i - 1, 2]]; datadiff = Append[datadiff, diff]];
   datadiffdiff = {};
   avdiffdiff = {};
   For[i = 1, i < Length[data] - 1, i++; diffdiff = datadiff[[i]] - datadiff[[i - 1]];
    datadiffdiff = Append[datadiffdiff, diffdiff]];
   For[i = 1, i < Length[data] - 2, i++; av = datadiffdiff[[i]] + datadiffdiff[[i - 1]];
    avdiffdiff = Append[avdiffdiff, av]];
   pos = Position[avdiffdiff, Max[avdiffdiff]][[1]][[1]];
   CT = Position[datadiffdiff, Max[datadiffdiff[[pos ;; pos + 1]]]][[1]][[1]] + 2;
   data[[1 ;; CT]]}[[1]];
```

Now, we define a cost function. It is set as the residual sum of squares. The variable 'yList' is the list of DNA values and the variable 'intensity' is a list of the corresponding fluorescence intensity values. These lists are populated in each iteration of optimization when a simulation with the current parameter values is performed. The for loop performs this operation. The variable q is the current dsDNA value and d is the value for the previous cycle. The data used to evaluate the cost function is determined by 'dataCTfunc' above.

```
c[x : {_?NumberQ ..}] := TimeConstrained[Module[{}, {
      yList = {d0} /. Thread[par -> x];
      intensity = {};
      q = d + k * Log[1 + d / k] /. Thread[par → x];
      For[count = 1, count < Length[dataCT] + 1, count++, y1 = q /. d → yList[[count]];
       yList = Append[yList, y1];
       intensity = Append[intensity, (y1 + base) /. Thread[par -> x]]];
      Total[((#[[2]] - intensity[[#[[1]]]])^2 & /@ dataCT)]}][[1]], 2];
```

Here, we define the optimization method. It is set to Nelder-Mead. Initialize number of loops with 'NumberOfLoops'. If the optimization algorithm gets stuck in a local minimum, a new loop will begin and start optimizing from a new point. Set 'PertuAmp', the variable that affects the window size for an initial parameter value for a new loop.

```
method =
  {{"DifferentialEvolution", "SearchPoints" → 400, "RandomSeed" → RandomInteger[{1, 100}]},
   {"SimulatedAnnealing", "PerturbationScale" → 3, "BoltzmannExponent" →
     Function[{i, df, f0}, -df/e^(i/10)], "RandomSeed" → RandomInteger[{1, 100}]},
   {"NelderMead", "ShrinkRatio" → 0.95`, "ContractRatio" → 0.95`,
    "ReflectRatio" → 1.5`, "RandomSeed" → RandomInteger[{1, 100}]},
   {Automatic, "RandomSeed" → RandomInteger[{1, 100}]}};
MethodType = 3;
NumberOfLoops = 0;
PertuAmp = 0.9;(*you can fix amplitude of perturbation
 or make it a decreasing function of the loop number 1/10^NumberOfLoops *)
```

MAK2 is the main function. The variables 'data' and 'frac' are passed to 'dataCTfunc' and 'dirname' is the name of the directory that data is written to--a new foler is made for each experimental well.

The three parameters and corresponding constraints must be defined. An initial parameter set is also defined with "current_fit". Maximum number of optimization iterations are set with 'MaxIter'. No new loops (see below) will be started after this value has been reached.

If the current cost function value is lower than any previous value, then the display is updated.

```
MAK2[data_] :=
  {SelectionMove[nb, All, GeneratedCell];
   coutlow = 1;
   dataCT = dataCTfunc[data];

   par = {d0, k, base}; dhigh = .01; dlow = 10^(-12);
   constraints = Join[{d0 < dhigh, d0 > dlow,
      k < Max[data[[All, 2]]], k > 0.001, base > -Abs[1.1 * b], base < Abs[1.1 * b]}];
   {d0 → dlow * 100, k → 1.0, base → b}[[All, 2]] >> "current_fit";
   MaxIter = 5000;

   ShowStatus[status_] := LinkWrite[$ParentLink,
     SetNotebookStatusLine[FrontEnd`EvaluationNotebook[], ToString[status]]];
   nb = EvaluationNotebook[];
   spar = ToString /@ par;

   i = 0;
   NumberOfLoops = 0;
   While[i < MaxIter, NumberOfLoops++;

    curr = ReadList["current_fit"][[1]];
    v = Flatten[#, 1] & /@ Transpose[{par, N[{1 - PertuAmp, 1 + PertuAmp} (#)] & /@ Abs[curr]}];
    rt = SessionTime[];
    NMinimize[{c[par], constraints}, v, Method → method[[3]],
     MaxIterations → 1000, StepMonitor :→ (par >> "current_fit";
       cout = N[Round[c[par] * 10^8] / (10^8)];
       If[cout ≤ coutlow * 0.99999,
        lowoutRoutine = {Module[{st = "c=" <> ToString[N[(c[par])]] <> "    " <> "i=" <>
               ToString[i] <> "    " <> "t=" <> ToString[Round[SessionTime[] - rt]] <>
               "     " <> "loops=" <> ToString[NumberOfLoops]},
           ShowStatus[st]; NotebookDelete[nb];

           Print[Show[{ListPlot[data, PlotLabel -> st,
               PlotRange → All], ListLinePlot[intensity, PlotRange → All]}]];
           rt = SessionTime[];]; Print[Thread[spar → par]]; SelectionMove[
           nb, All, GeneratedCell]; coutlow = cout};];
       i++)]];
  };
```

Now, we set the filepath and import data from a .csv file. First column of the file should be cycles. The column data are named. Data are transposed with the cycle numbers so that the data for an experimental well is a 2 x n array with column numbers and corresponding fluorescence.

The following data in 'growth.csv' was generated by the authors.

```
filepath = "Desktop/data/";
SetDirectory[filepath];
indata = Import["growth.csv"];
cycle = indata[[All, 1]];

D3data = Transpose[{cycle, indata[[All, 2]]}][[2 ;; 41]];
D4data = Transpose[{cycle, indata[[All, 3]]}][[2 ;; 41]];
D5data = Transpose[{cycle, indata[[All, 4]]}][[2 ;; 41]];
D6data = Transpose[{cycle, indata[[All, 5]]}][[2 ;; 41]];
D7data = Transpose[{cycle, indata[[All, 6]]}][[2 ;; 41]];
D8data = Transpose[{cycle, indata[[All, 7]]}][[2 ;; 41]];
E3data = Transpose[{cycle, indata[[All, 8]]}][[2 ;; 41]];
E4data = Transpose[{cycle, indata[[All, 9]]}][[2 ;; 41]];
E5data = Transpose[{cycle, indata[[All, 10]]}][[2 ;; 41]];
E6data = Transpose[{cycle, indata[[All, 11]]}][[2 ;; 41]];
E7data = Transpose[{cycle, indata[[All, 12]]}][[2 ;; 41]];
E8data = Transpose[{cycle, indata[[All, 13]]}][[2 ;; 41]];
```

What follows are actual trials that can be reevaluated.

```
MAK2[D3data]
```

c=0.0000729625 i=3627 t=0 loops=4

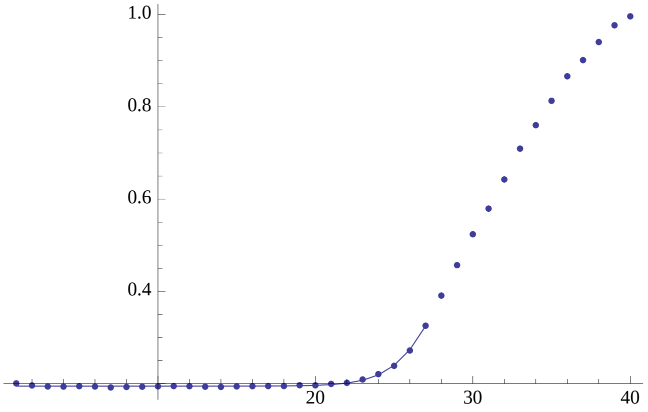

{d0 → 1.59848×10^{-9}, k → 0.0635667, base → 0.194352}

{Null}

```
MAK2[D4data]
```

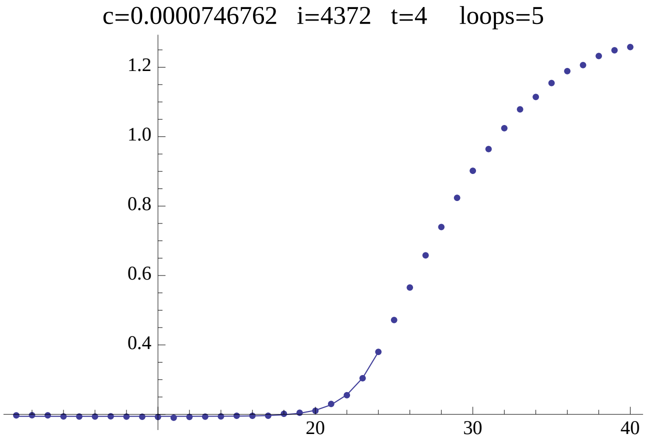

{d0 → 1.71786 × 10^{-8}, k → 0.102162, base → 0.194352}

{Null}

MAK2[D5data]

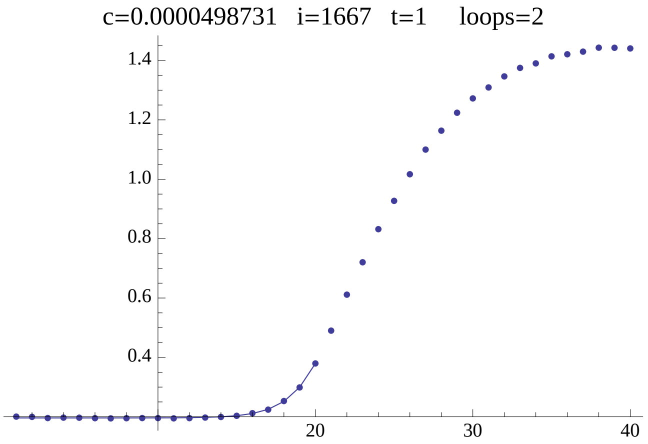

{d0 → 2.30757 × 10^{-7}, k → 0.162611, base → 0.196127}

{Null}

MAK2[D6data]

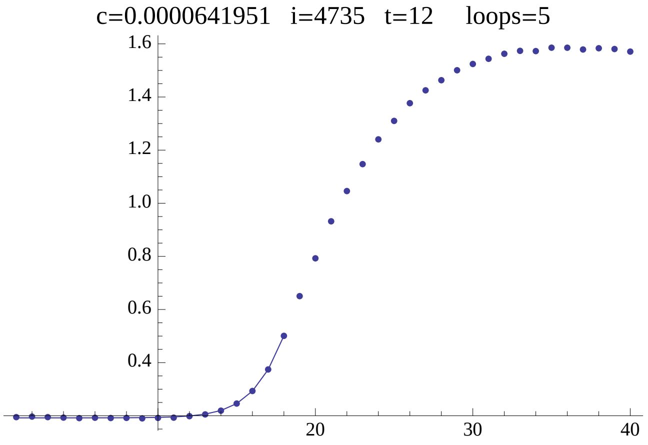

{d0 → 1.75799 × 10^{-6}, k → 0.188528, base → 0.192083}

{Null}

MAK2[**D7data**]

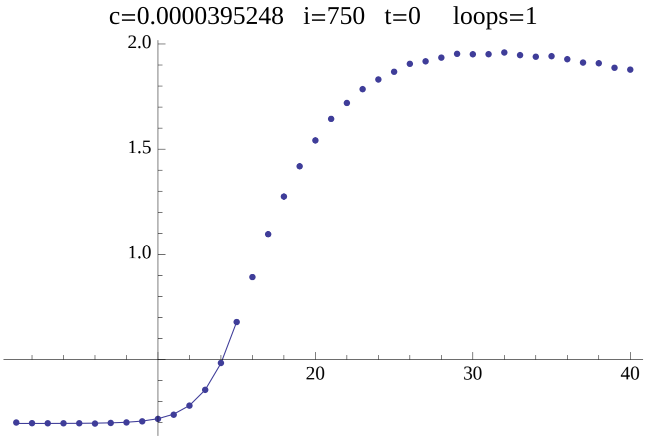

{d0 → 0.0000224502, k → 0.279824, base → 0.195958}

{Null}

MAK2[**D8data**]

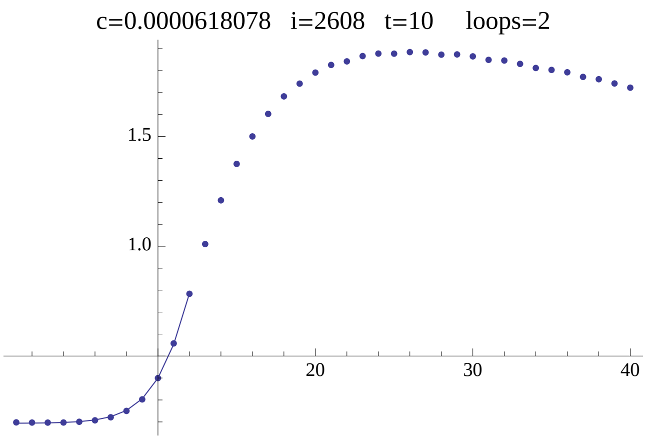

{d0 → 0.000241108, k → 0.279332, base → 0.19366}

{Null}

MAK2[E3data]

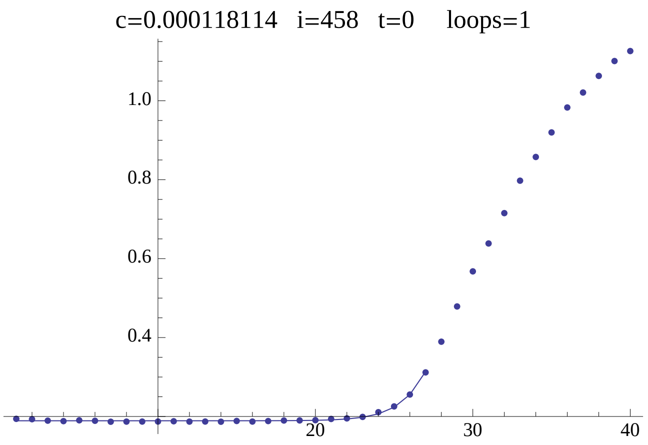

{d0 → 1.05864 × 10^{-9}, k → 0.233823, base → 0.189163}

{Null}

MAK2[E4data]

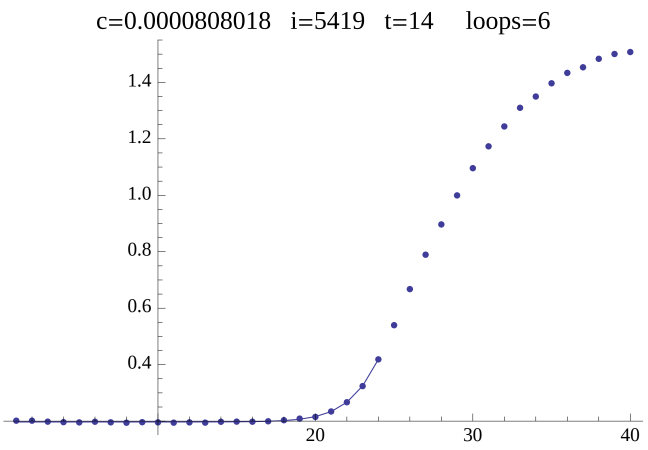

{d0 → 1.85471 × 10^-8, k → 0.159537, base → 0.196981}

{Null}

MAK2[E5data]

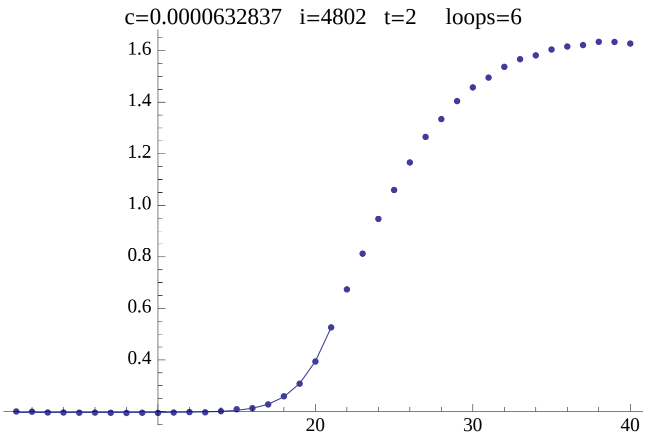

{d0 → 2.52569 × 10^-7, k → 0.16845, base → 0.196262}

{Null}

MAK2[E6data]

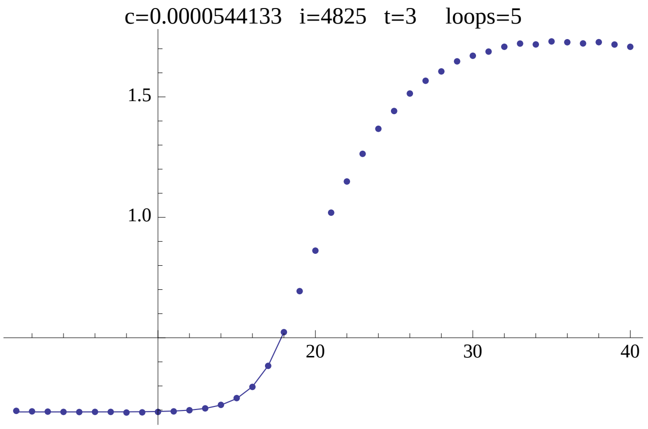

{d0 → 1.79533 × 10^{-6}, k → 0.230369, base → 0.192131}

{Null}

MAK2[E7data]

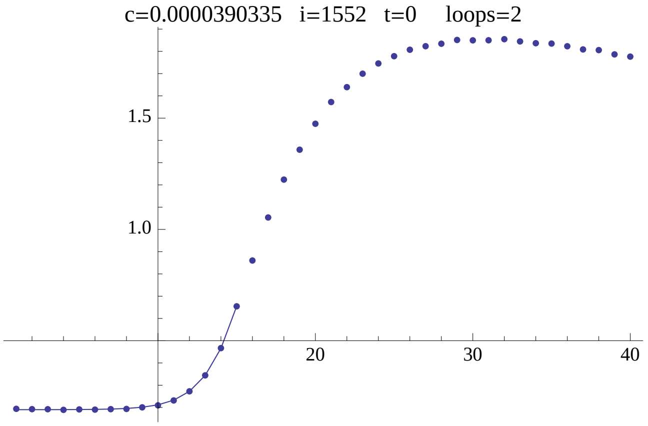

{d0 → 0.0000217174, k → 0.265815, base → 0.190084}

{Null}

MAK2[E8data]

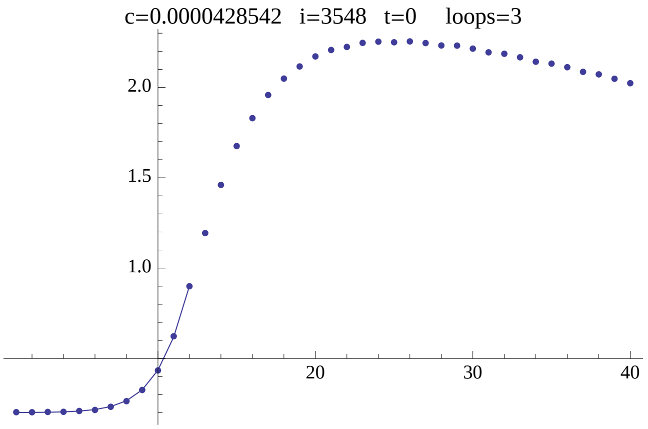


```
{d0 → 0.000267742, k → 0.380452, base → 0.200492}
```

```
{Null}
```

The following are data and trials from the 'rutledge.csv' dataset.

```
indata = Import["rutledge.csv"];
cycle = indata[[All, 1]][[2 ;;]];

X111data = Transpose[{cycle, indata[[All, 3]][[2 ;;]]}];
X112data = Transpose[{cycle, indata[[All, 4]][[2 ;;]]}];
X113data = Transpose[{cycle, indata[[All, 5]][[2 ;;]]}];
X114data = Transpose[{cycle, indata[[All, 6]][[2 ;;]]}];
X121data = Transpose[{cycle, indata[[All, 7]][[2 ;;]]}];
X122data = Transpose[{cycle, indata[[All, 8]][[2 ;;]]}];
X123data = Transpose[{cycle, indata[[All, 9]][[2 ;;]]}];
X124data = Transpose[{cycle, indata[[All, 10]][[2 ;;]]}];
X131data = Transpose[{cycle, indata[[All, 11]][[2 ;;]]}];
X132data = Transpose[{cycle, indata[[All, 12]][[2 ;;]]}];
X133data = Transpose[{cycle, indata[[All, 13]][[2 ;;]]}];
X134data = Transpose[{cycle, indata[[All, 14]][[2 ;;]]}];
X141data = Transpose[{cycle, indata[[All, 15]][[2 ;;]]}];
X142data = Transpose[{cycle, indata[[All, 16]][[2 ;;]]}];
X143data = Transpose[{cycle, indata[[All, 17]][[2 ;;]]}];
X144data = Transpose[{cycle, indata[[All, 18]][[2 ;;]]}];
X151data = Transpose[{cycle, indata[[All, 19]][[2 ;;]]}];
X152data = Transpose[{cycle, indata[[All, 20]][[2 ;;]]}];
X153data = Transpose[{cycle, indata[[All, 21]][[2 ;;]]}];
X154data = Transpose[{cycle, indata[[All, 22]][[2 ;;]]}];
X211data = Transpose[{cycle, indata[[All, 23]][[2 ;;]]}];
X212data = Transpose[{cycle, indata[[All, 24]][[2 ;;]]}];
X213data = Transpose[{cycle, indata[[All, 25]][[2 ;;]]}];
X214data = Transpose[{cycle, indata[[All, 26]][[2 ;;]]}];
X221data = Transpose[{cycle, indata[[All, 27]][[2 ;;]]}];
X222data = Transpose[{cycle, indata[[All, 28]][[2 ;;]]}];
```

```
X223data = Transpose[{cycle, indata[[All, 29]][[2 ;;]]}];
X224data = Transpose[{cycle, indata[[All, 30]][[2 ;;]]}];
X231data = Transpose[{cycle, indata[[All, 31]][[2 ;;]]}];
X232data = Transpose[{cycle, indata[[All, 32]][[2 ;;]]}];
X233data = Transpose[{cycle, indata[[All, 33]][[2 ;;]]}];
X234data = Transpose[{cycle, indata[[All, 34]][[2 ;;]]}];
X241data = Transpose[{cycle, indata[[All, 35]][[2 ;;]]}];
X242data = Transpose[{cycle, indata[[All, 36]][[2 ;;]]}];
X243data = Transpose[{cycle, indata[[All, 37]][[2 ;;]]}];
X244data = Transpose[{cycle, indata[[All, 38]][[2 ;;]]}];
X251data = Transpose[{cycle, indata[[All, 39]][[2 ;;]]}];
X252data = Transpose[{cycle, indata[[All, 40]][[2 ;;]]}];
X253data = Transpose[{cycle, indata[[All, 41]][[2 ;;]]}];
X254data = Transpose[{cycle, indata[[All, 42]][[2 ;;]]}];
X311data = Transpose[{cycle, indata[[All, 43]][[2 ;;]]}];
X312data = Transpose[{cycle, indata[[All, 44]][[2 ;;]]}];
X313data = Transpose[{cycle, indata[[All, 45]][[2 ;;]]}];
X314data = Transpose[{cycle, indata[[All, 46]][[2 ;;]]}];
X321data = Transpose[{cycle, indata[[All, 47]][[2 ;;]]}];
X322data = Transpose[{cycle, indata[[All, 48]][[2 ;;]]}];
X323data = Transpose[{cycle, indata[[All, 49]][[2 ;;]]}];
X324data = Transpose[{cycle, indata[[All, 50]][[2 ;;]]}];
X331data = Transpose[{cycle, indata[[All, 51]][[2 ;;]]}];
X332data = Transpose[{cycle, indata[[All, 52]][[2 ;;]]}];
X333data = Transpose[{cycle, indata[[All, 53]][[2 ;;]]}];
X334data = Transpose[{cycle, indata[[All, 54]][[2 ;;]]}];
X341data = Transpose[{cycle, indata[[All, 55]][[2 ;;]]}];
X342data = Transpose[{cycle, indata[[All, 56]][[2 ;;]]}];
X343data = Transpose[{cycle, indata[[All, 57]][[2 ;;]]}];
X344data = Transpose[{cycle, indata[[All, 58]][[2 ;;]]}];
X351data = Transpose[{cycle, indata[[All, 59]][[2 ;;]]}];
X352data = Transpose[{cycle, indata[[All, 60]][[2 ;;]]}];
X353data = Transpose[{cycle, indata[[All, 61]][[2 ;;]]}];
X354data = Transpose[{cycle, indata[[All, 62]][[2 ;;]]}];
X411data = Transpose[{cycle, indata[[All, 63]][[2 ;;]]}];
X412data = Transpose[{cycle, indata[[All, 64]][[2 ;;]]}];
X413data = Transpose[{cycle, indata[[All, 65]][[2 ;;]]}];
X414data = Transpose[{cycle, indata[[All, 66]][[2 ;;]]}];
X421data = Transpose[{cycle, indata[[All, 67]][[2 ;;]]}];
X422data = Transpose[{cycle, indata[[All, 68]][[2 ;;]]}];
X423data = Transpose[{cycle, indata[[All, 69]][[2 ;;]]}];
X424data = Transpose[{cycle, indata[[All, 70]][[2 ;;]]}];
X431data = Transpose[{cycle, indata[[All, 71]][[2 ;;]]}];
X432data = Transpose[{cycle, indata[[All, 72]][[2 ;;]]}];
X433data = Transpose[{cycle, indata[[All, 73]][[2 ;;]]}];
X434data = Transpose[{cycle, indata[[All, 74]][[2 ;;]]}];
X441data = Transpose[{cycle, indata[[All, 75]][[2 ;;]]}];
X442data = Transpose[{cycle, indata[[All, 76]][[2 ;;]]}];
X443data = Transpose[{cycle, indata[[All, 77]][[2 ;;]]}];
X444data = Transpose[{cycle, indata[[All, 78]][[2 ;;]]}];
X451data = Transpose[{cycle, indata[[All, 79]][[2 ;;]]}];
```

```
X452data = Transpose[{cycle, indata[[All, 80]][[2 ;;]]}];
X453data = Transpose[{cycle, indata[[All, 81]][[2 ;;]]}];
X454data = Transpose[{cycle, indata[[All, 82]][[2 ;;]]}];
X511data = Transpose[{cycle, indata[[All, 83]][[2 ;;]]}];
X512data = Transpose[{cycle, indata[[All, 84]][[2 ;;]]}];
X513data = Transpose[{cycle, indata[[All, 85]][[2 ;;]]}];
X514data = Transpose[{cycle, indata[[All, 86]][[2 ;;]]}];
X521data = Transpose[{cycle, indata[[All, 87]][[2 ;;]]}];
X522data = Transpose[{cycle, indata[[All, 88]][[2 ;;]]}];
X523data = Transpose[{cycle, indata[[All, 89]][[2 ;;]]}];
X524data = Transpose[{cycle, indata[[All, 90]][[2 ;;]]}];
X531data = Transpose[{cycle, indata[[All, 91]][[2 ;;]]}];
X532data = Transpose[{cycle, indata[[All, 92]][[2 ;;]]}];
X533data = Transpose[{cycle, indata[[All, 93]][[2 ;;]]}];
X534data = Transpose[{cycle, indata[[All, 94]][[2 ;;]]}];
X541data = Transpose[{cycle, indata[[All, 95]][[2 ;;]]}];
X542data = Transpose[{cycle, indata[[All, 96]][[2 ;;]]}];
X543data = Transpose[{cycle, indata[[All, 97]][[2 ;;]]}];
X544data = Transpose[{cycle, indata[[All, 98]][[2 ;;]]}];
X551data = Transpose[{cycle, indata[[All, 99]][[2 ;;]]}];
X552data = Transpose[{cycle, indata[[All, 100]][[2 ;;]]}];
X553data = Transpose[{cycle, indata[[All, 101]][[2 ;;]]}];
X554data = Transpose[{cycle, indata[[All, 102]][[2 ;;]]}];
X611data = Transpose[{cycle, indata[[All, 103]][[2 ;;]]}];
X612data = Transpose[{cycle, indata[[All, 104]][[2 ;;]]}];
X613data = Transpose[{cycle, indata[[All, 105]][[2 ;;]]}];
X614data = Transpose[{cycle, indata[[All, 106]][[2 ;;]]}];
X621data = Transpose[{cycle, indata[[All, 107]][[2 ;;]]}];
X622data = Transpose[{cycle, indata[[All, 108]][[2 ;;]]}];
X623data = Transpose[{cycle, indata[[All, 109]][[2 ;;]]}];
X624data = Transpose[{cycle, indata[[All, 110]][[2 ;;]]}];
X631data = Transpose[{cycle, indata[[All, 111]][[2 ;;]]}];
X632data = Transpose[{cycle, indata[[All, 112]][[2 ;;]]}];
X633data = Transpose[{cycle, indata[[All, 113]][[2 ;;]]}];
X634data = Transpose[{cycle, indata[[All, 114]][[2 ;;]]}];
X641data = Transpose[{cycle, indata[[All, 115]][[2 ;;]]}];
X642data = Transpose[{cycle, indata[[All, 116]][[2 ;;]]}];
X643data = Transpose[{cycle, indata[[All, 117]][[2 ;;]]}];
X644data = Transpose[{cycle, indata[[All, 118]][[2 ;;]]}];
X651data = Transpose[{cycle, indata[[All, 119]][[2 ;;]]}];
X652data = Transpose[{cycle, indata[[All, 120]][[2 ;;]]}];
X653data = Transpose[{cycle, indata[[All, 121]][[2 ;;]]}];
X654data = Transpose[{cycle, indata[[All, 122]][[2 ;;]]}];
```

```
MAK2[X111data]
```

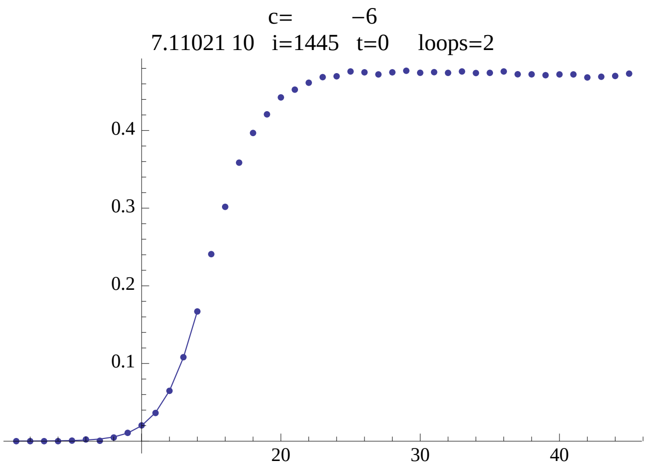

{d0 → 0.0000211516, k → 0.0547555, base → 0.0000725995}

{Null}

MAK2[X112data]

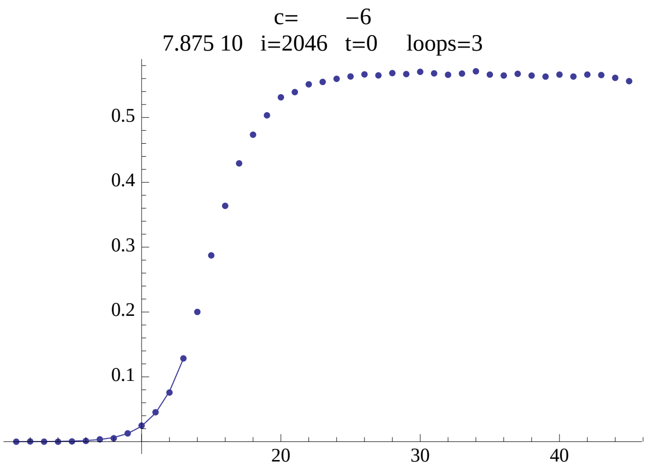

{d0 → 0.0000255827, k → 0.0627979, base → 0.0000744994}

{Null}

MAK2[X113data]

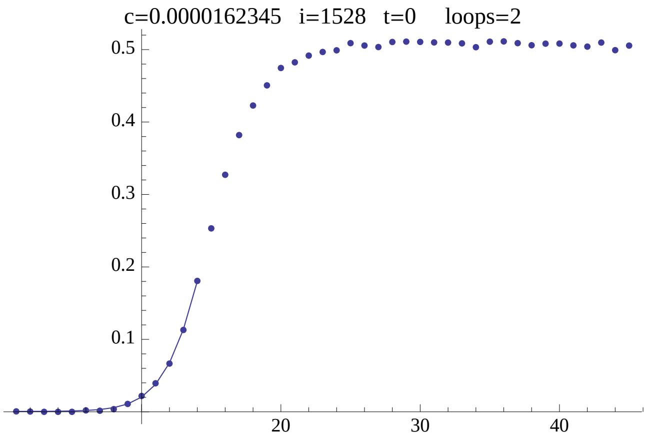

{d0 → 0.0000209167, k → 0.0669527, base → 0.000518441}

{Null}

MAK2[X114data]

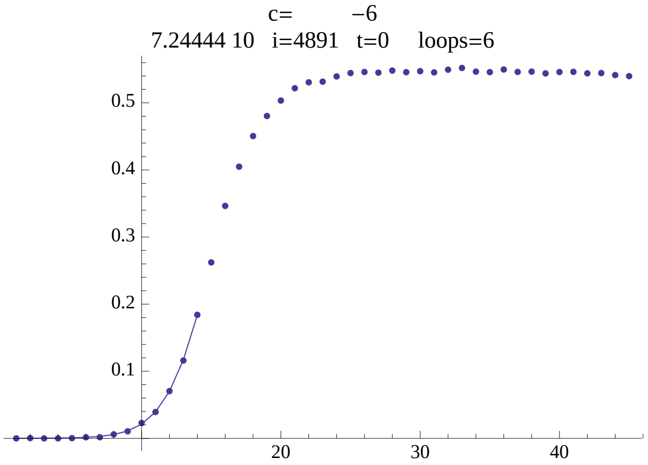

{d0 → 0.0000224298, k → 0.0634366, base → 0.0000705597}

{Null}

MAK2[X121data]

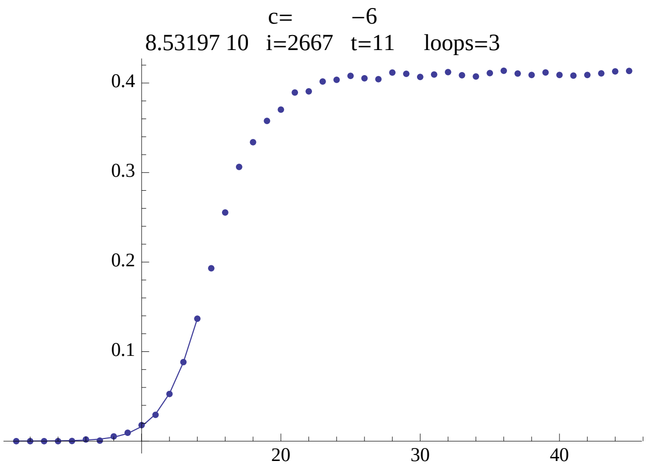

{d0 → 0.0000173814, k → 0.0445128, base → 0.0000790486}

{Null}

MAK2[X122data]

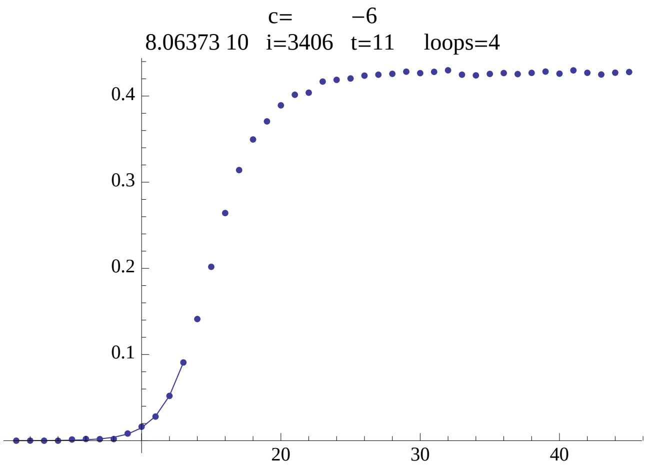

{d0 → 0.0000153504, k → 0.0679371, base → 0.0000796993}

{Null}

MAK2[X123data]

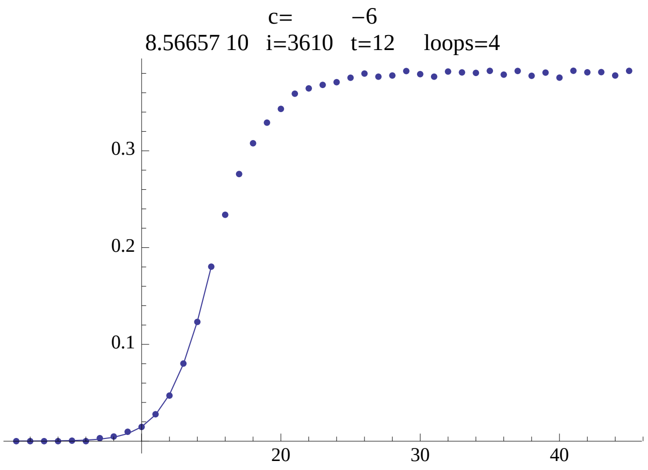

{d0 → 0.0000157156, k → 0.0402342, base → 0.0000758361}

{Null}

MAK2[X124data]

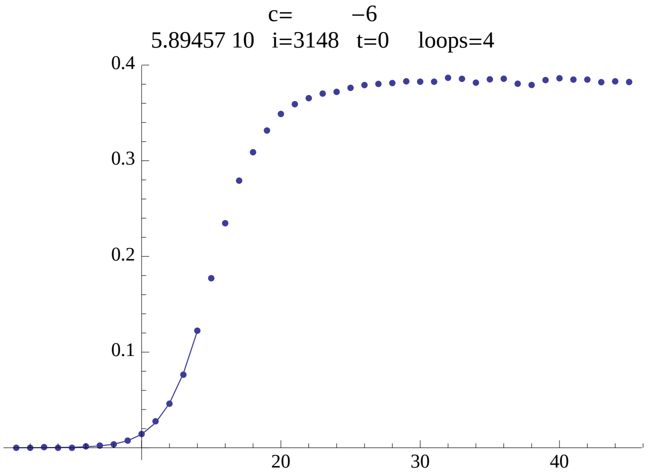

{d0 → 0.0000148131, k → 0.0423767, base → 0.0000800989}

{Null}

MAK2[X131data]

c=0.0000160346 i=5510 t=12 loops=7

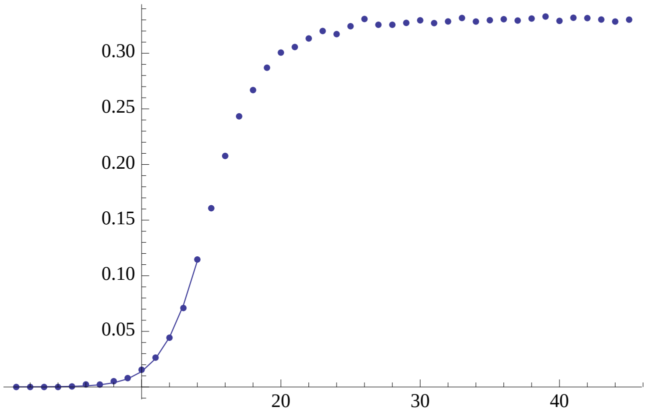

{d0 → 0.0000147834, k → 0.0355564, base → 0.0000841035}

{Null}

MAK2[X132data]

c=0.0000109123 i=5176 t=12 loops=6

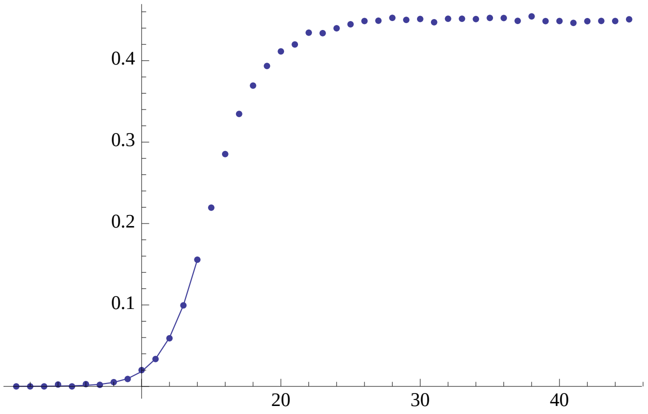

{d0 → 0.0000193173, k → 0.0522334, base → 0.0000758587}

{Null}

MAK2[X133data]

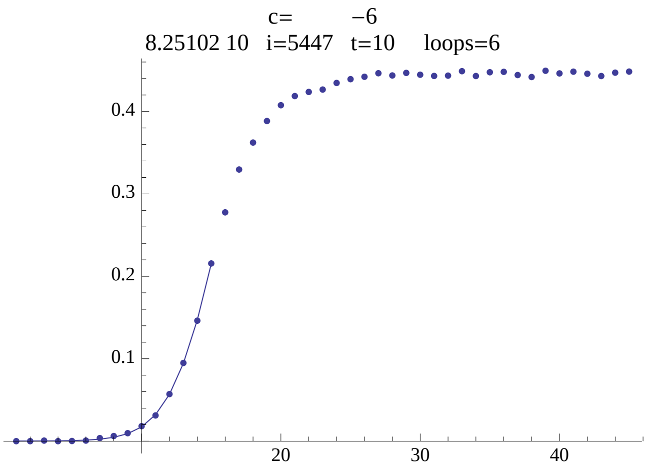

{d0 → 0.0000184043, k → 0.0490376, base → 0.0000813033}

{Null}

MAK2[X134data]

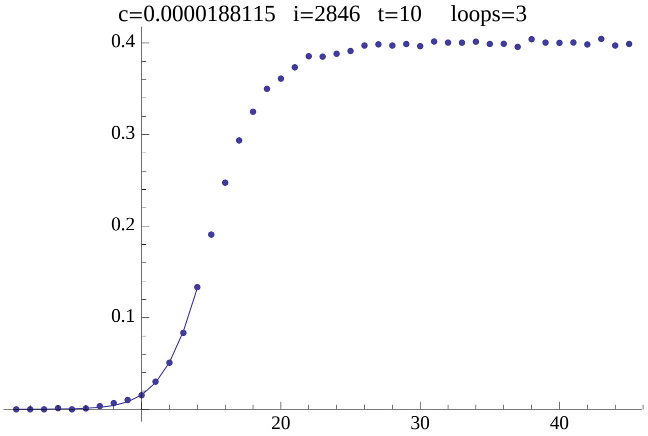

{d0 → 0.0000166774, k → 0.0437521, base → 0.0000802299}

{Null}

MAK2[X141data]

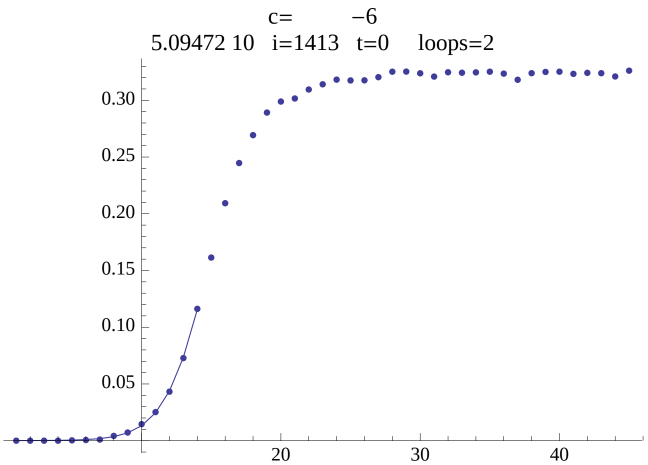

{d0 → 0.00001388, k → 0.041005, base → 0.0000762559}

{Null}

MAK2[X142data]

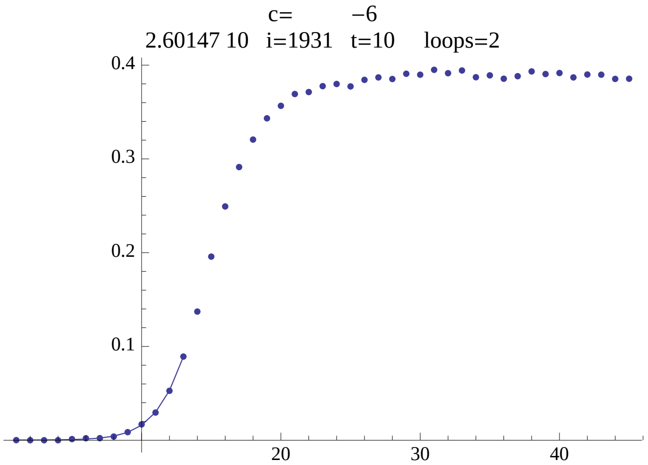

{d0 → 0.0000167966, k → 0.0494791, base → 0.000154325}

{Null}

MAK2[X143data]

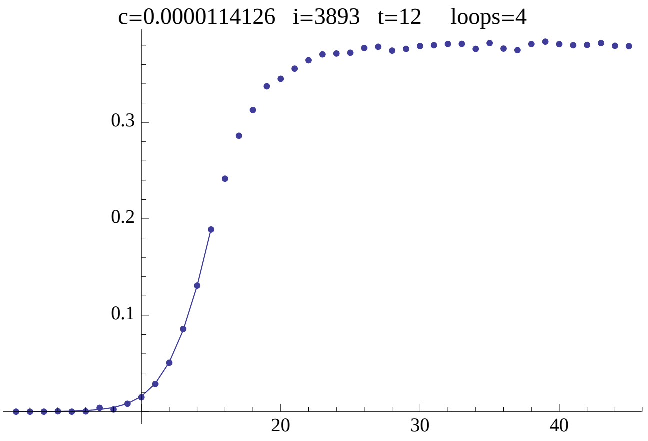

{d0 → 0.0000169211, k → 0.0412664, base → 0.0000701969}

{Null}

MAK2[X144data]

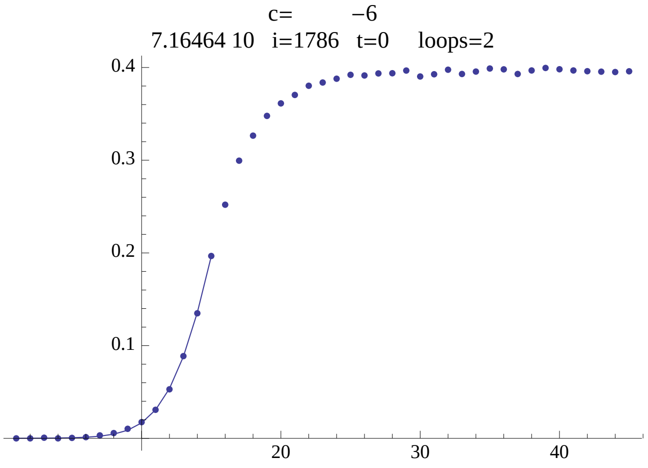

{d0 → 0.0000178998, k → 0.041991, base → 0.0000783979}

{Null}

MAK2[X151data]

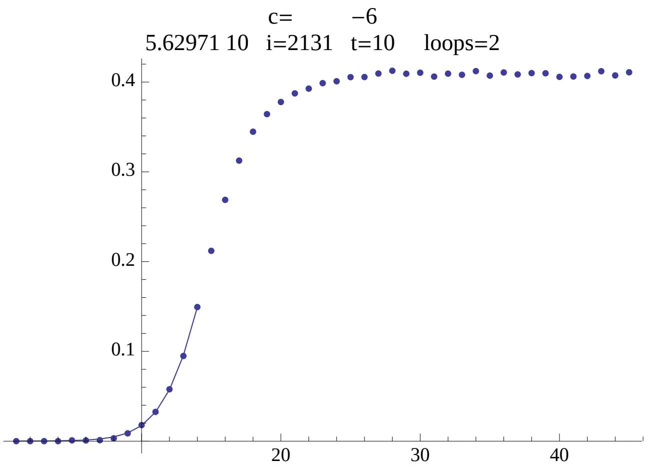

{d0 → 0.0000184738, k → 0.0503875, base → 0.0000735895}

{Null}

MAK2[X152data]

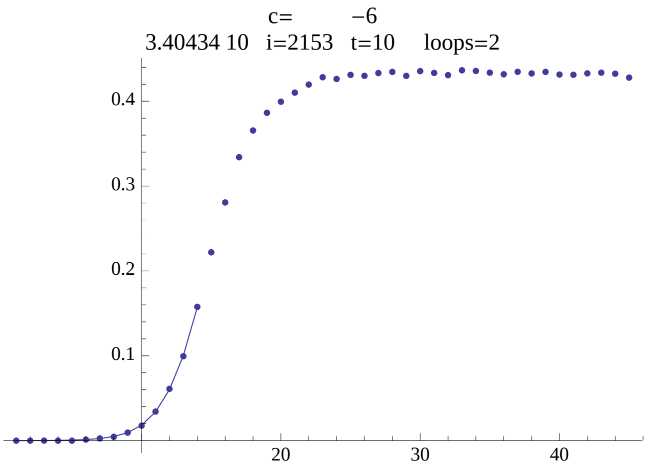

{d0 → 0.0000193295, k → 0.0537811, base → 0.0000732598}

{Null}

MAK2[X153data]

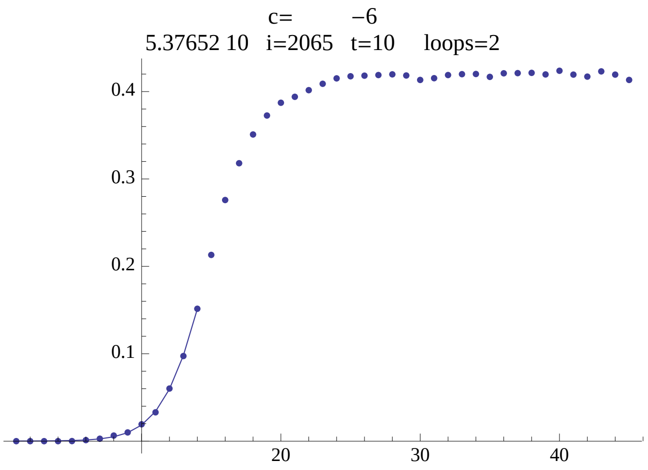

{d0 → 0.0000197776, k → 0.047303, base → 0.0000736628}

{Null}

MAK2[X154data]

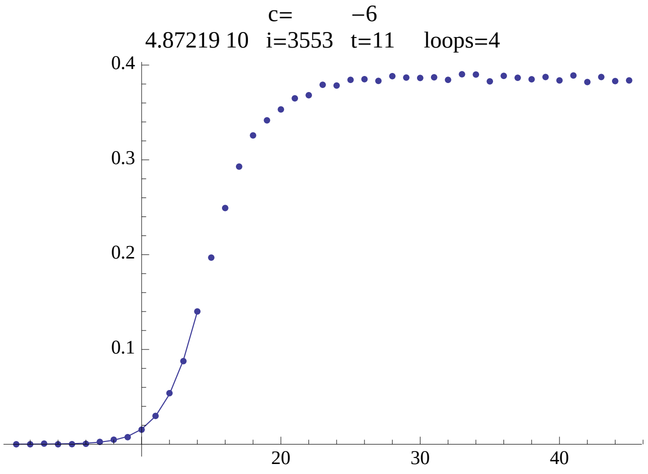

{d0 → 0.0000166967, k → 0.0498706, base → 0.0000797315}

{Null}

MAK2[X211data]

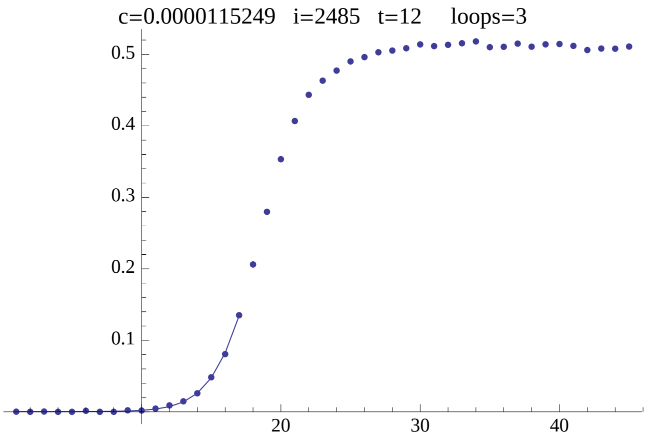

{d0 → 1.74897 × 10^{-6}, k → 0.0603787, base → 0.000301597}

{Null}

MAK2[X212data]

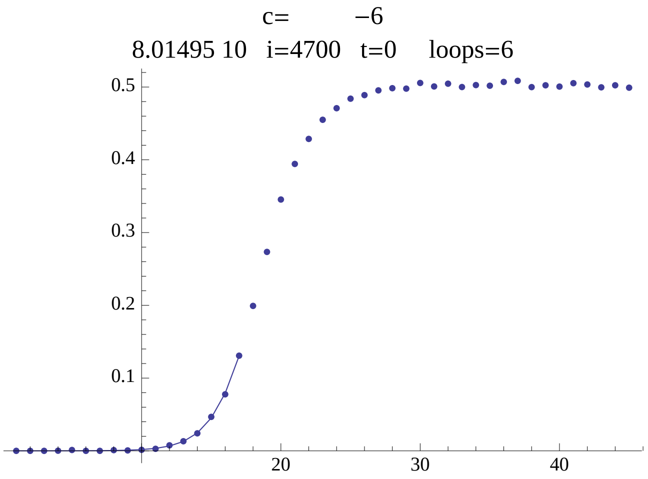

{d0 → 1.64595 × 10^{-6}, k → 0.0627888, base → 0.0000758373}

{Null}

MAK2[X213data]

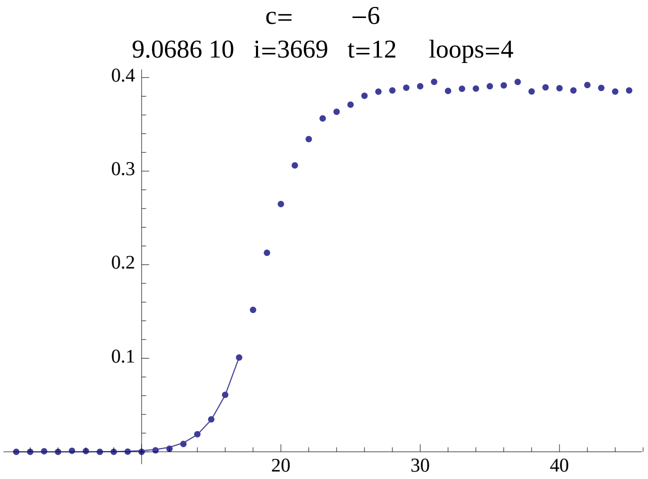

{d0 → 1.22445 × 10^{-6}, k → 0.0529717, base → -0.0000169154}

{Null}

MAK2[X214data]

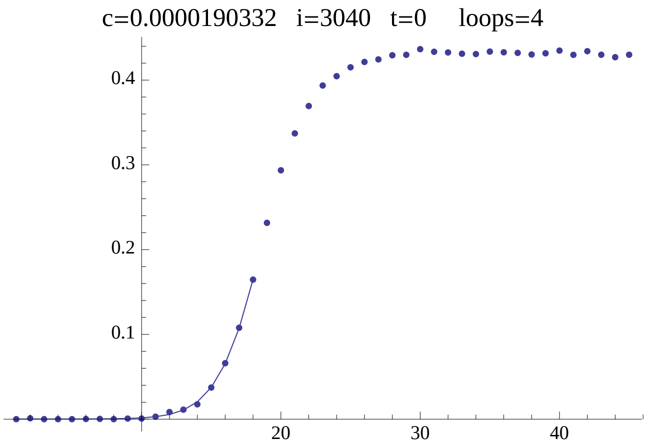

{d0 → 1.36899 × 10^{-6}, k → 0.0504158, base → 0.000109602}

{Null}

MAK2[X221data]

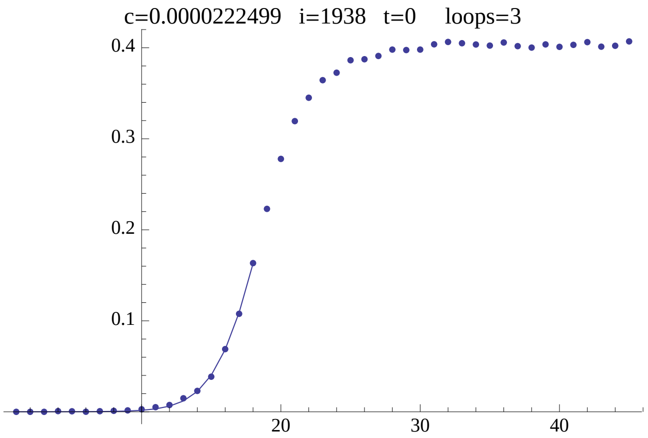

{d0 → 1.54948 × 10^{-6}, k → 0.0421241, base → 0.00011}

{Null}

MAK2[X222data]

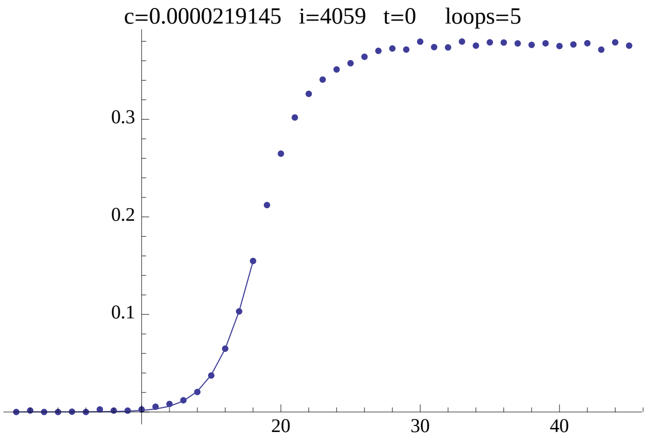

{d0 → 1.45936 × 10^{-6}, k → 0.0402708, base → 0.000109986}

{Null}

MAK2[X223data]

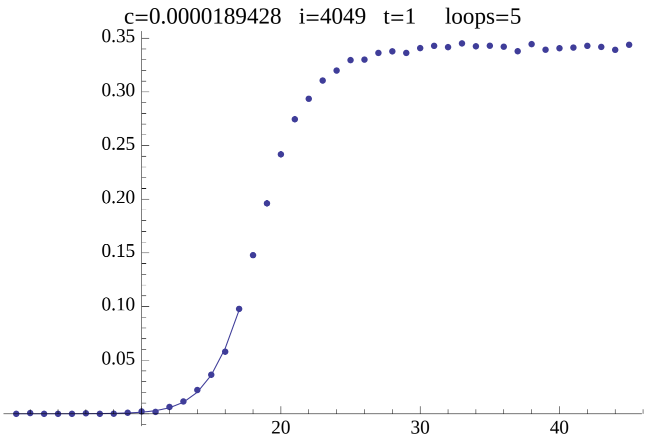

{d0 → 1.40305 × 10^{-6}, k → 0.0360402, base → 0.0000796797}

{Null}

MAK2[X224data]

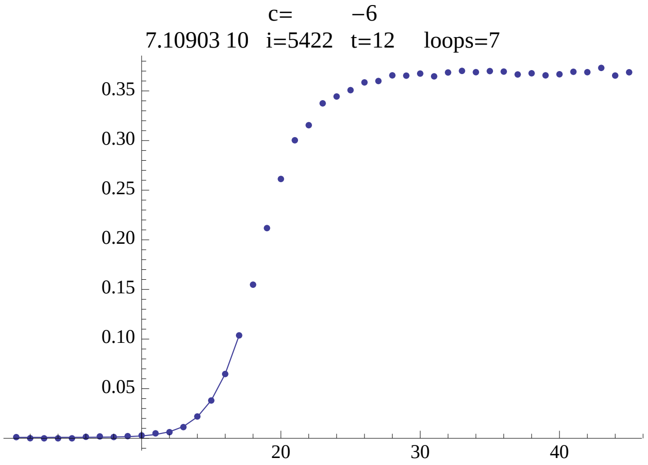

{d0 → 1.42086 × 10^{-6}, k → 0.0415489, base → 0.000947494}

{Null}

MAK2[X231data]

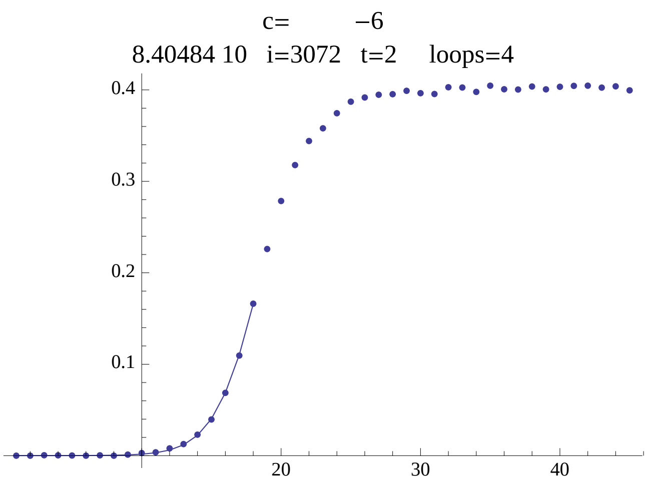

{d0 → 1.53024 × 10^{-6}, k → 0.0444653, base → 0.00011}

{Null}

MAK2[X232data]

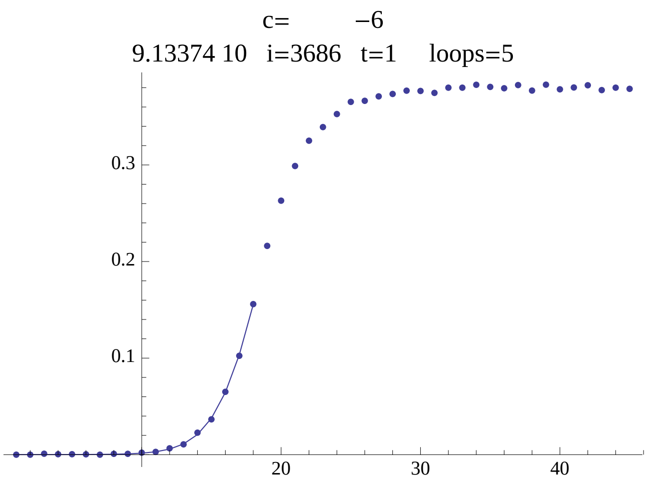

{d0 → 1.4335 × 10^{-6}, k → 0.0417002, base → 0.00011}

{Null}

MAK2[X233data]

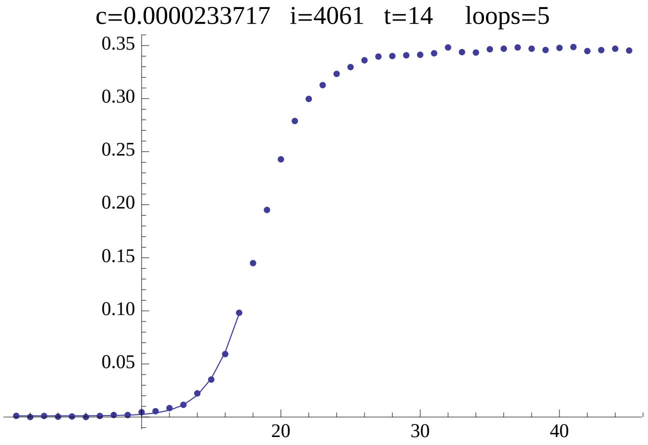

{d0 → 1.34529 × 10^{-6}, k → 0.0382281, base → 0.0010868}

{Null}

MAK2[X234data]

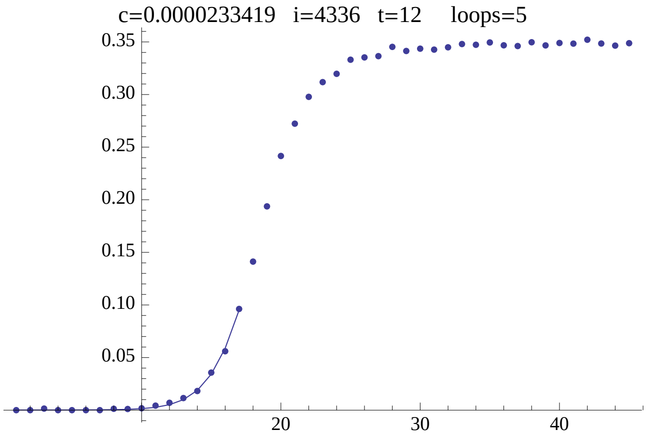

{d0 → 1.28182 × 10^{-6}, k → 0.0402749, base → 0.00011}

{Null}

MAK2[X241data]

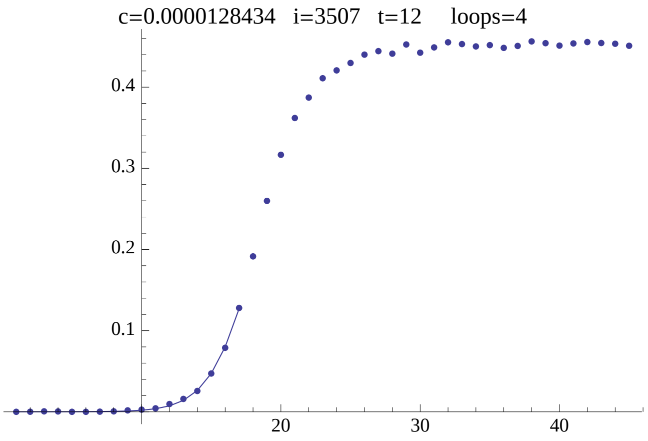

{d0 → 1.83375 × 10^-6, k → 0.0478746, base → 0.00011}

{Null}

MAK2[X242data]

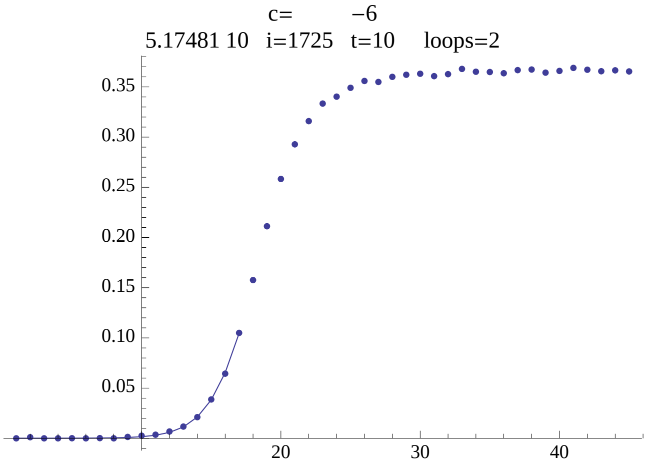

{d0 → 1.4645 × 10^-6, k → 0.0414065, base → 0.00011}

{Null}

MAK2[X243data]

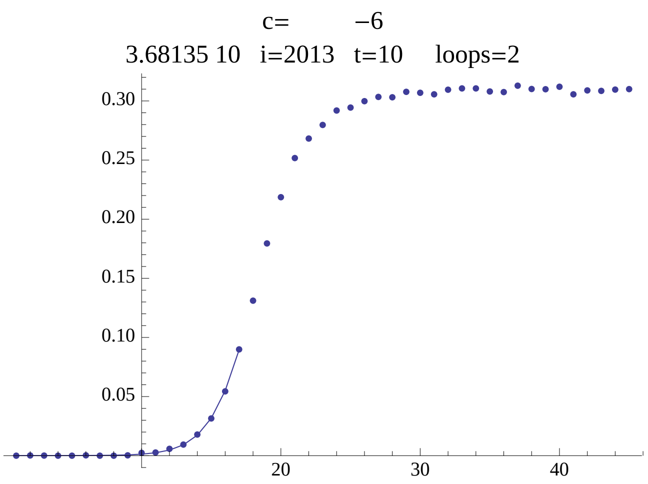

{d0 → 1.17296 × 10^{-6}, k → 0.0399297, base → 0.0000965587}

{Null}

MAK2[X244data]

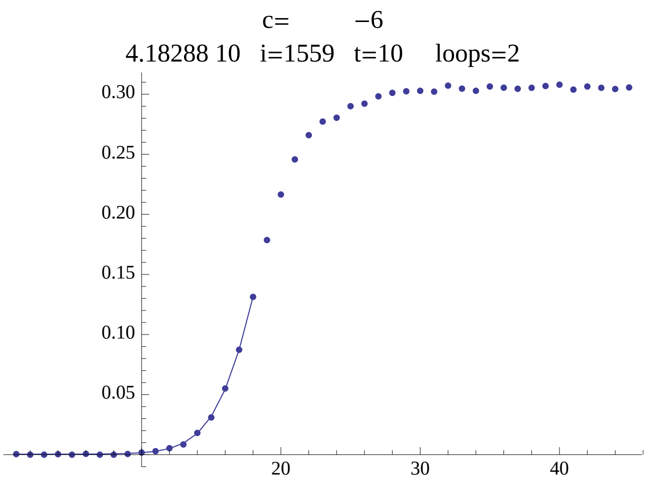

{d0 → 1.19103 × 10^{-6}, k → 0.0357843, base → 0.000405981}

{Null}

MAK2[X251data]

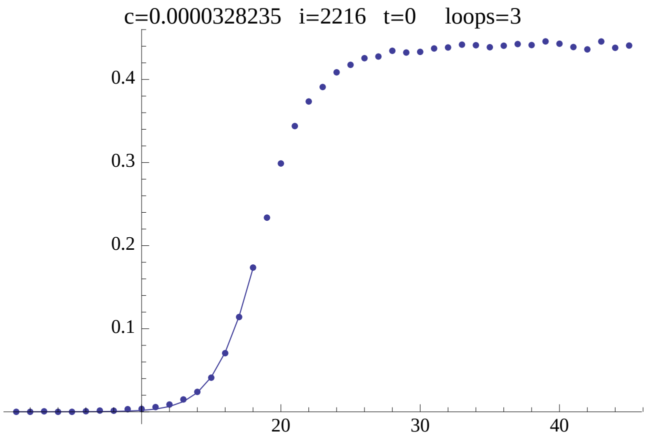

{d0 → 1.58669 × 10^{-6}, k → 0.0466028, base → 0.00011}

{Null}

MAK2[X252data]

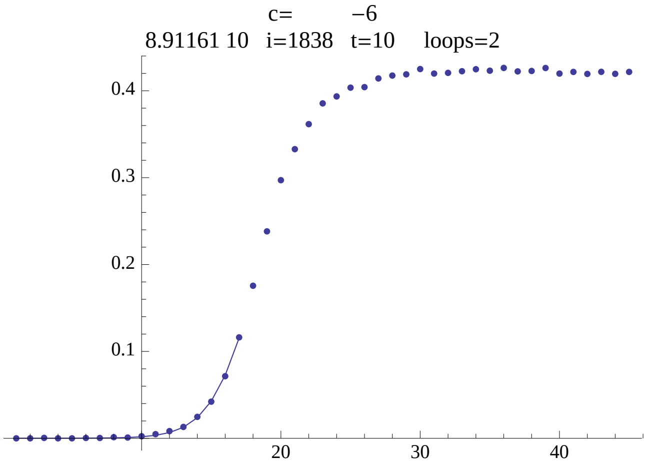

{d0 → 1.65132 × 10^{-6}, k → 0.0440925, base → 0.00011}

{Null}

MAK2[X253data]

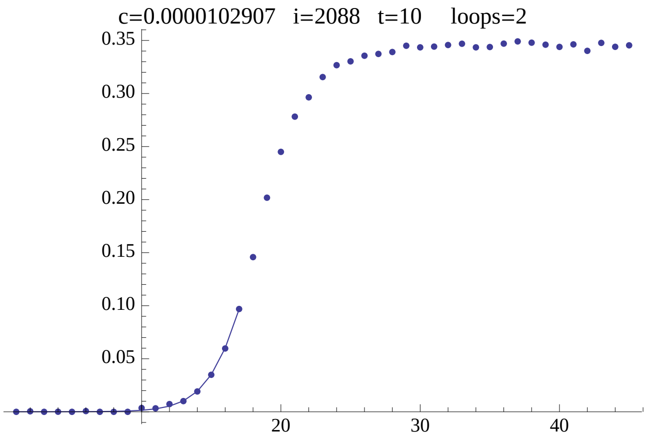

{d0 → 1.32244 × 10^{-6}, k → 0.0398722, base → 0.00011}

{Null}

MAK2[X254data]

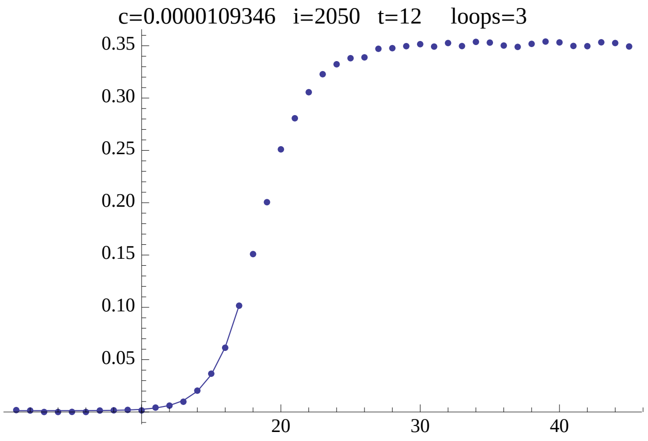

{d0 → 1.23903 × 10^{-6}, k → 0.0507799, base → 0.00126325}

{Null}

MAK2[X311data]

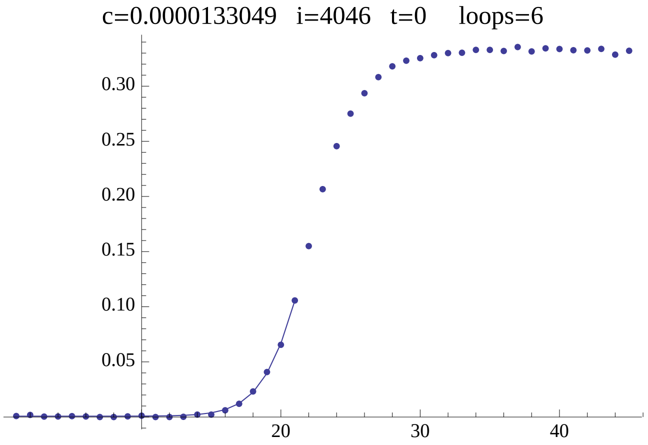

{d0 → 9.47733 × 10^{-8}, k → 0.0393325, base → 0.00074481}

{Null}

MAK2[X312data]

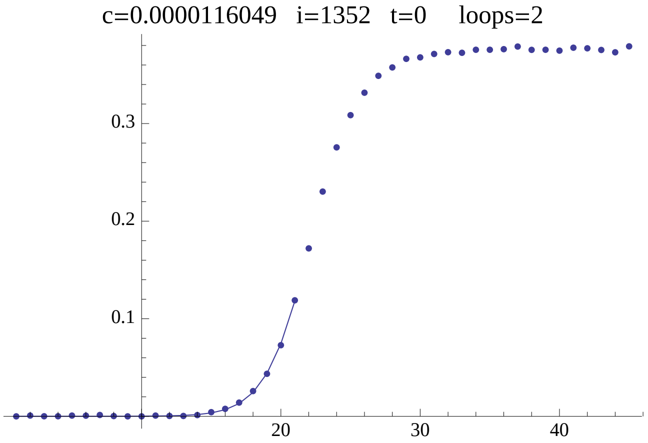

{d0 → 1.07018 × 10^{-7}, k → 0.0441896, base → 0.0000750592}

{Null}

MAK2[X313data]

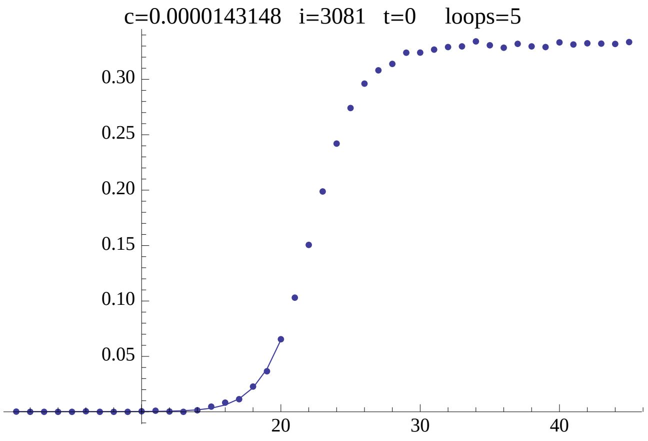

{d0 → 9.49828 × 10^{-8}, k → 0.0365064, base → 0.000229541}

{Null}

MAK2[**X314data**]

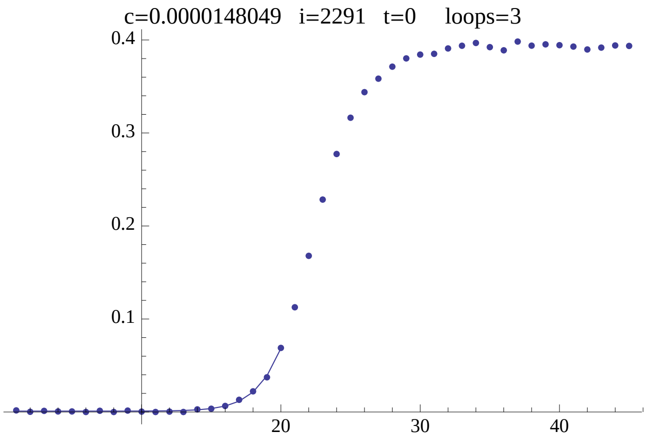

{d0 → 8.51898 × 10^{-8}, k → 0.0589681, base → 0.000915049}

{Null}

MAK2[**X321data**]

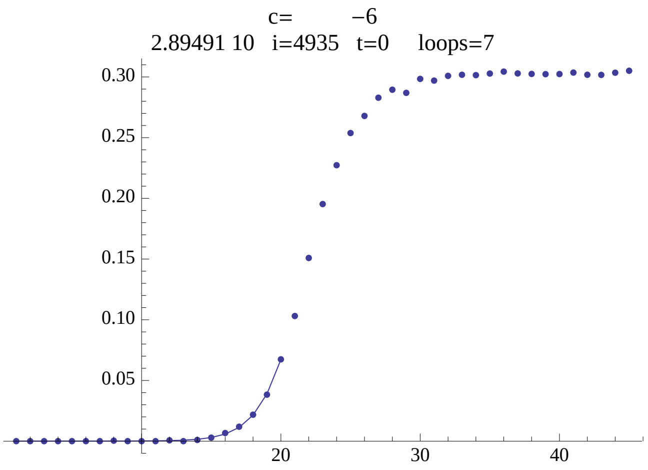

{d0 → 9.14548 × 10^{-8}, k → 0.0461974, base → 0.0000436121}

{Null}

MAK2[X322data]

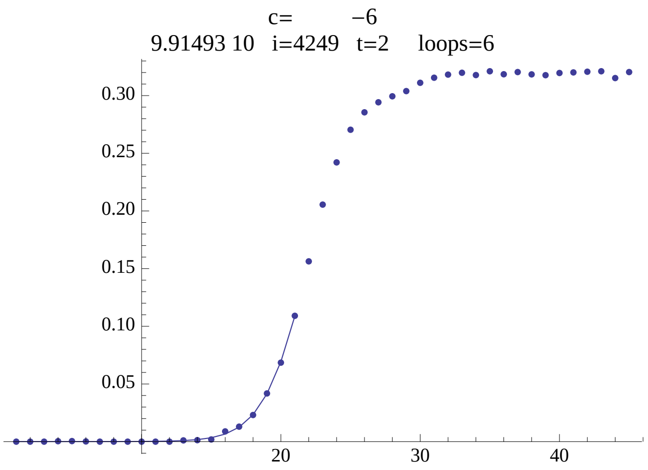

{d0 → 1.03398 × 10^{-7}, k → 0.0377521, base → 0.0000794433}

{Null}

MAK2[X323data]

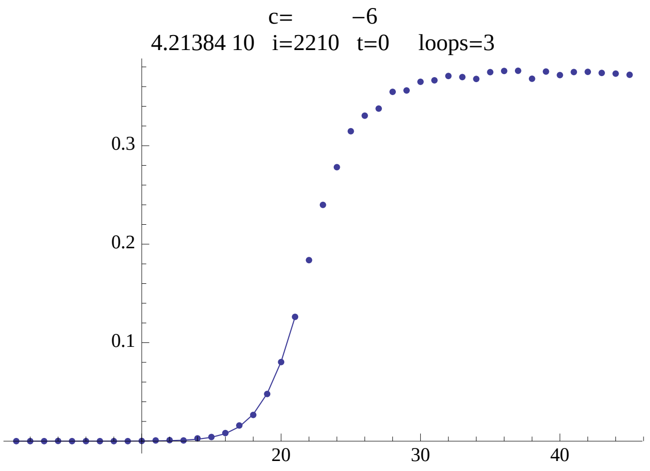

{d0 → 1.21782 × 10^{-7}, k → 0.042771, base → − 0.0000291252}

{Null}

MAK2[X324data]

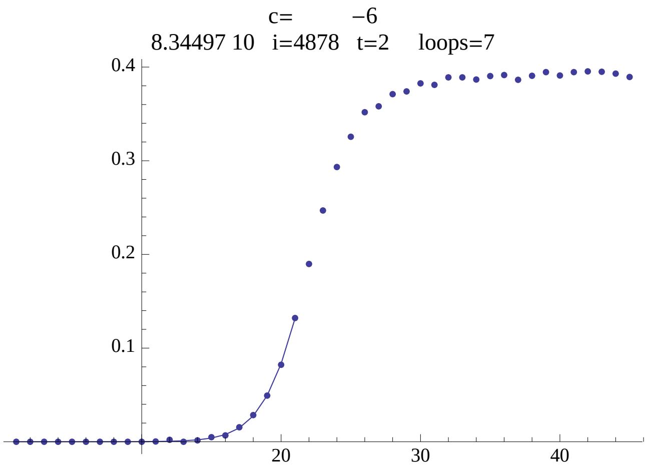

{d0 → 1.20887 × 10^{-7}, k → 0.048242, base → 0.0000760514}

{Null}

MAK2[X331data]

c= −6
6.88424 10 i=1346 t=0 loops=2

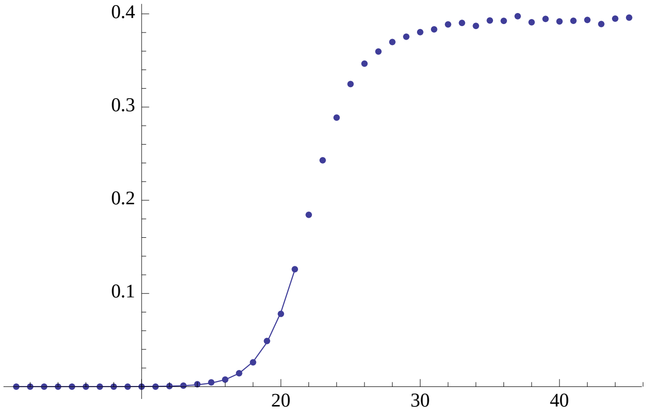

{d0 → 1.17568 × 10^{-7}, k → 0.044436, base → 0.000077565}

{Null}

MAK2[X332data]

c= −6
4.98427 10 i=2675 t=0 loops=4

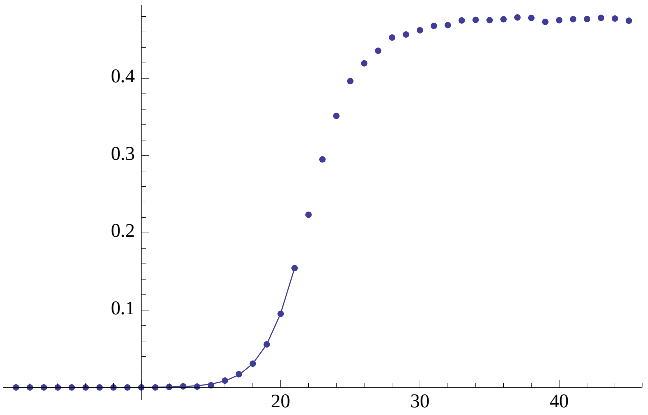

{d0 → 1.31331 × 10^{-7}, k → 0.064237, base → 0.0000761153}

{Null}

MAK2[X333data]

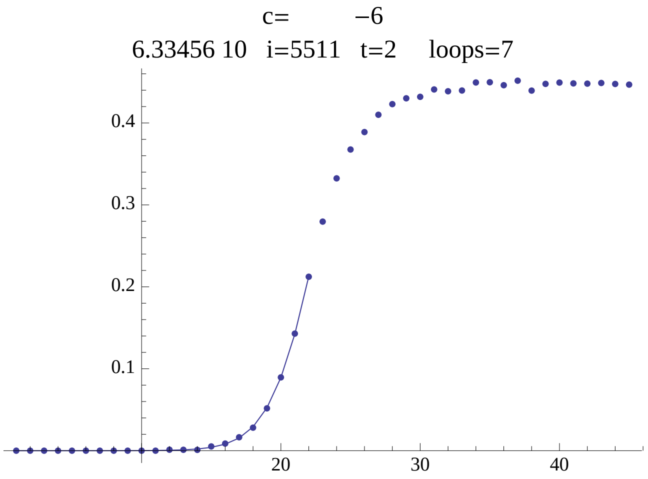

{d0 → 1.26519 × 10^{-7}, k → 0.0550656, base → 1.32218 × 10^{-8}}

{Null}

MAK2[X334data]

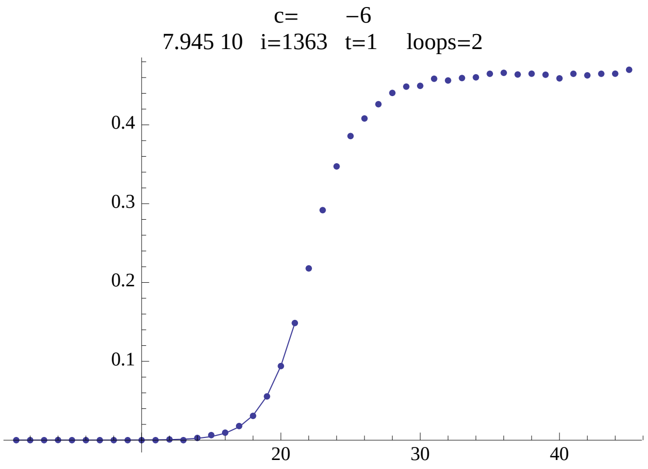

{d0 → 1.38001 × 10^{-7}, k → 0.0532945, base → 0.000074867}

{Null}

MAK2[X341data]

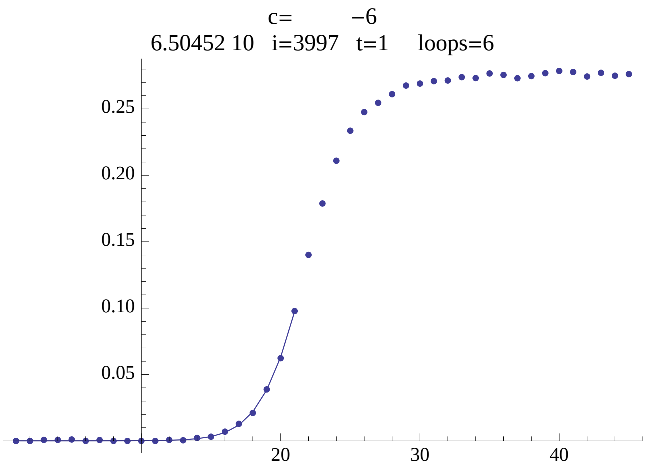

{d0 → 9.95116 × 10^{-8}, k → 0.0303874, base → 0.0000853477}

{Null}

MAK2[X342data]

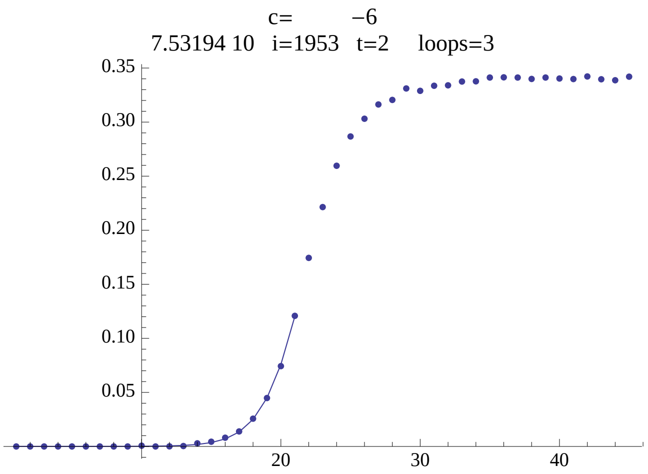

{d0 → 1.09334 × 10^{-7}, k → 0.0447068, base → 0.0000767723}

{Null}

MAK2[X343data]

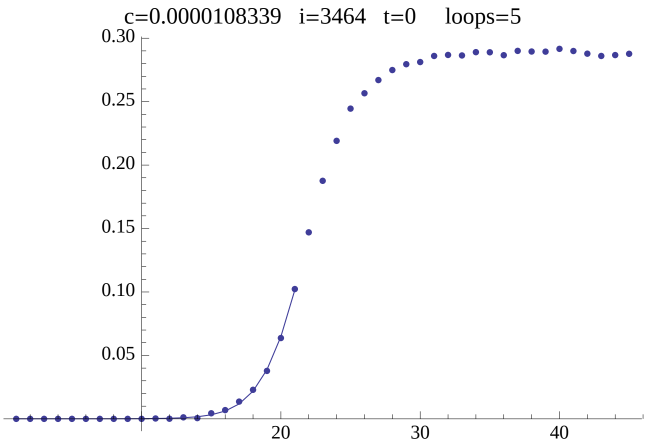

{d0 → 9.68507 × 10^{-8}, k → 0.0350697, base → 0.000079433}

{Null}

MAK2[X344data]

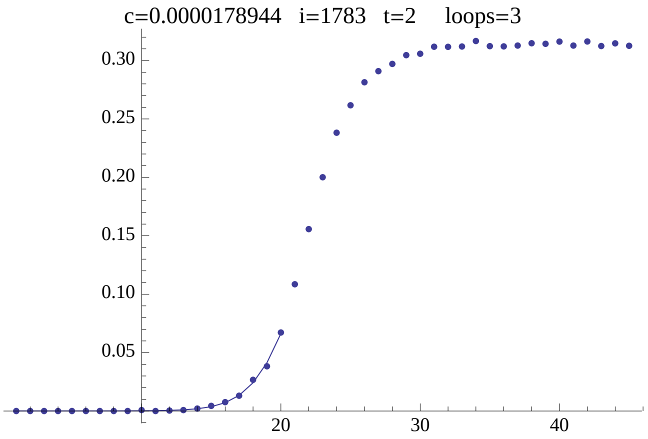

{d0 → 1.14916 × 10^{-7}, k → 0.0266979, base → 0.0000757949}

{Null}

MAK2[X351data]

c= −6
5.31468 10 i=4770 t=0 loops=7

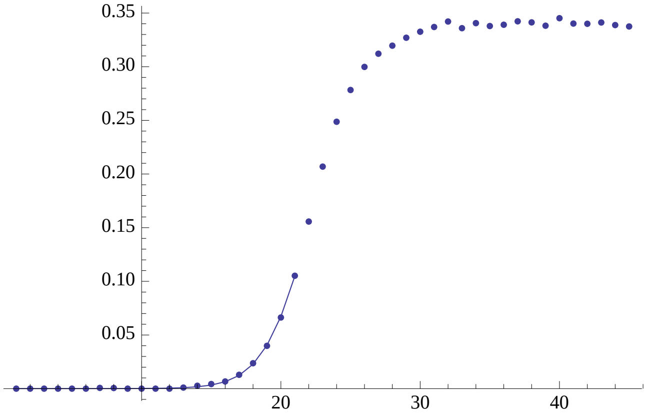

{d0 → 1.01881 × 10^-7, k → 0.0351937, base → 0.0000794228}

{Null}

MAK2[X352data]

c= −6
3.67486 10 i=4242 t=2 loops=6

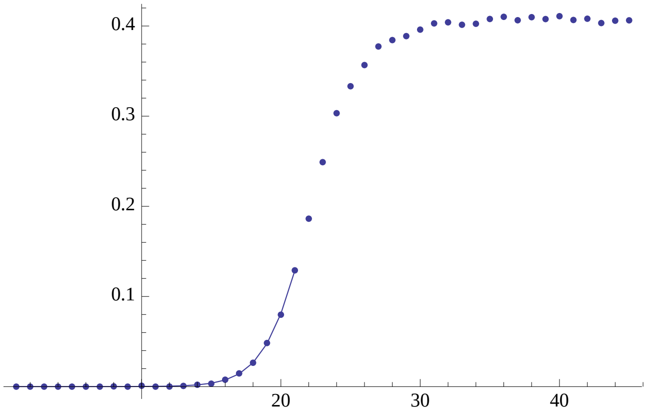

{d0 → 1.15451 × 10^-7, k → 0.0489108, base → 0.000075758}

{Null}

MAK2[X353data]

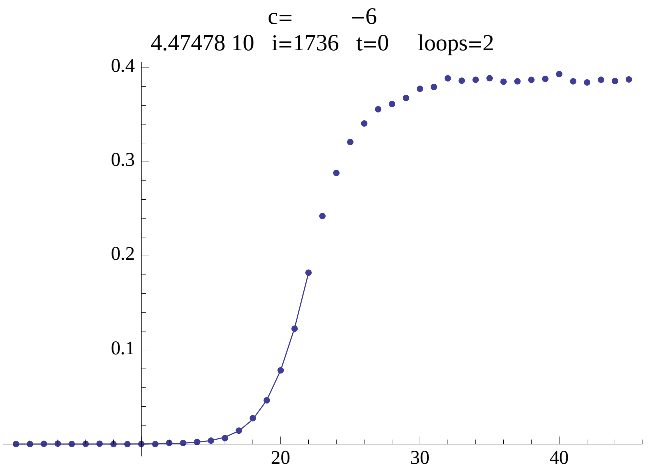

{d0 → 1.15382 × 10^{-7}, k → 0.0438013, base → 0.0000815814}

{Null}

MAK2[X354data]

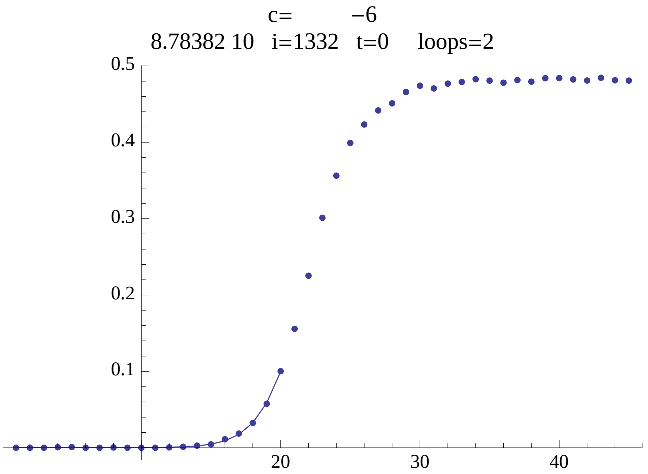

{d0 → 1.42635 × 10^{-7}, k → 0.0598717, base → 0.0000857774}

{Null}

MAK2[X411data]

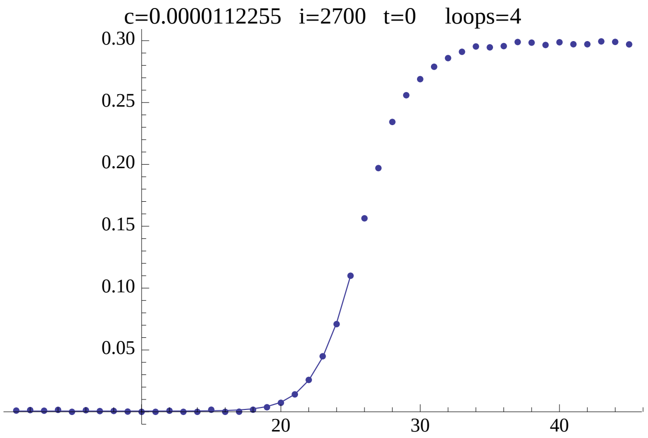

{d0 → 7.15979 × 10^{-9}, k → 0.0331137, base → 0.000578736}

{Null}

MAK2[X412data]

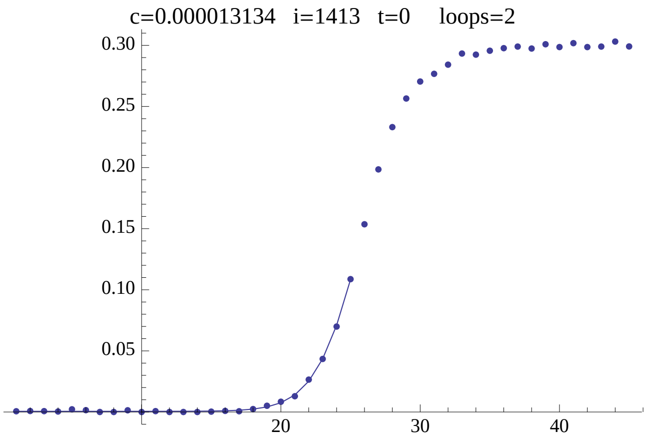

{d0 → 7.09357 × 10^{-9}, k → 0.0325253, base → 0.000521952}

{Null}

MAK2[X413data]

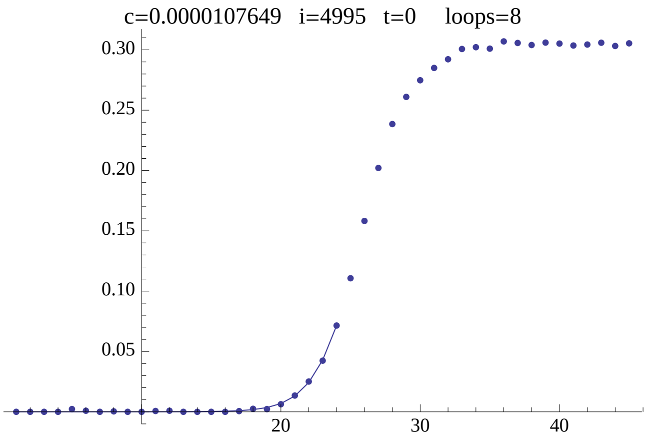

{d0 → 6.78437 × 10^{-9}, k → 0.0372647, base → 0.0000742077}

{Null}

{d0 → 6.77392 × 10^{-9}, k → 0.036774, base → 0.000162683}

{Null}

MAK2[X414data]

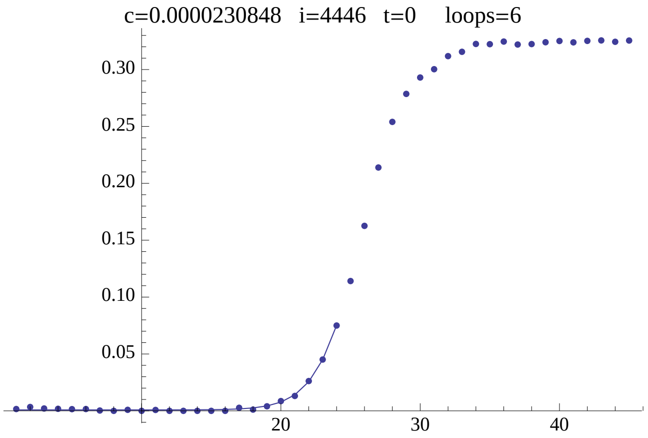

{d0 → 6.86123 × 10^{-9}, k → 0.0413581, base → 0.000800836}

{Null}

MAK2[X421data]

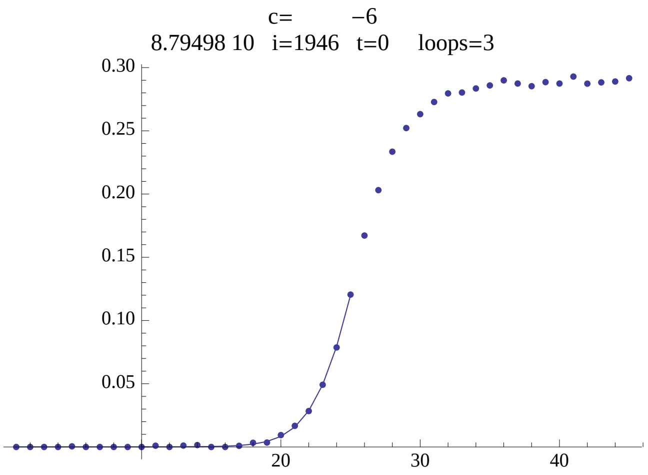

{d0 → 8.41739 × 10^{-9}, k → 0.0332881, base → 0.000078565}

{Null}

MAK2[X422data]

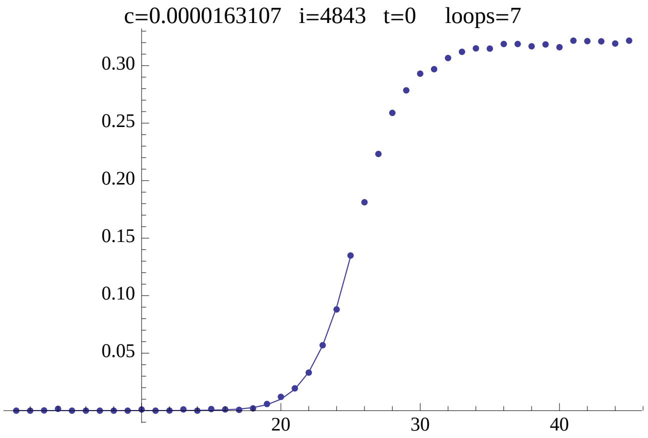

{d0 → 1.0139 × 10^{-8}, k → 0.0339028, base → 0.0000858234}

{Null}

MAK2[X423data]

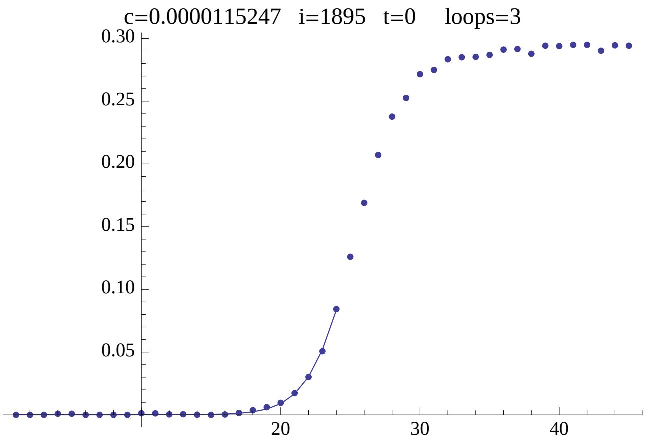

{d0 → 8.96456 × 10^{-9}, k → 0.0342922, base → 0.0000776909}

{Null}

MAK2[X424data]

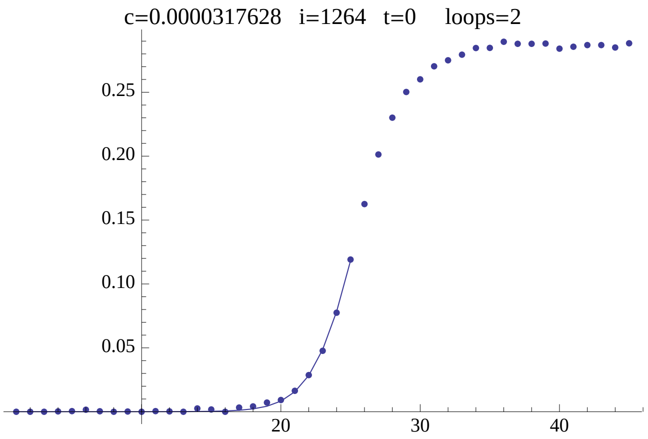

{d0 → 8.38342 × 10^{-9}, k → 0.0323229, base → 0.0000800686}

{Null}

MAK2[X431data]

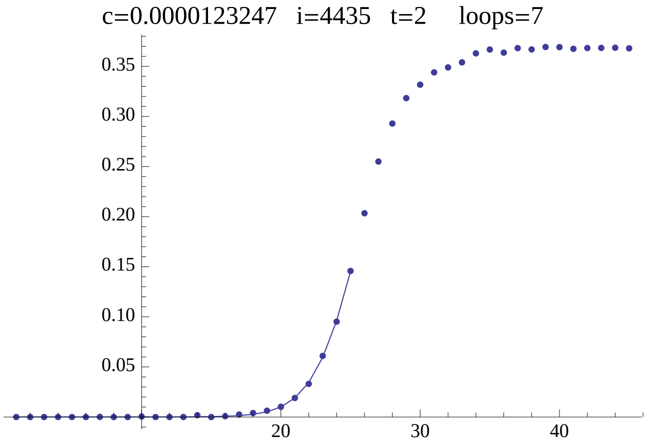

{d0 → 1.0119 × 10^{-8}, k → 0.0406919, base → 0.0000749599}

{Null}

MAK2[X432data]

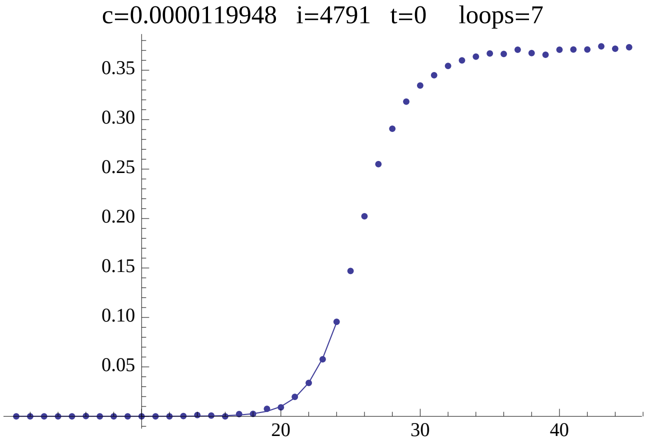

{d0 → 9.9279 × 10^{-9}, k → 0.0407538, base → 0.0000788918}

{Null}

MAK2[X433data]

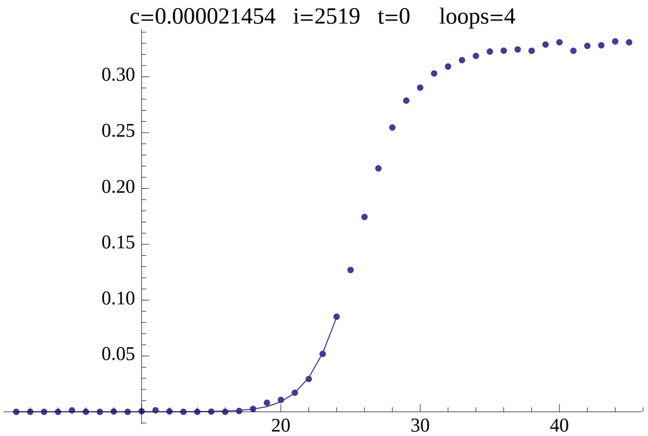

{d0 → 9.06503 × 10^{-9}, k → 0.0347197, base → 0.0000780145}

{Null}

MAK2[**X434data**]

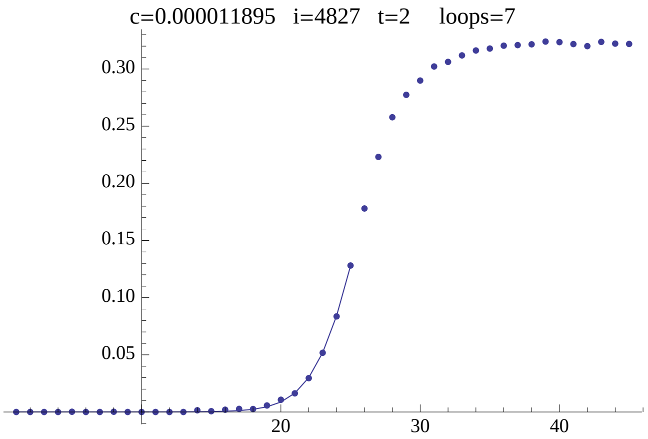

{d0 → 8.75385 × 10^{-9}, k → 0.0364814, base → 0.0000787434}

{Null}

MAK2[**X441data**]

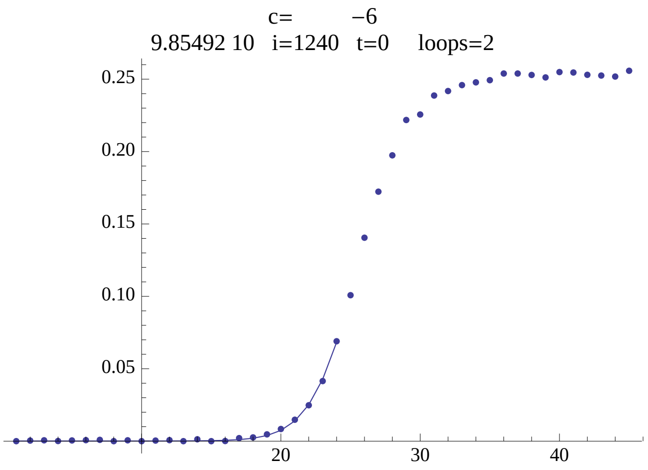

{d0 → 7.5403 × 10^{-9}, k → 0.0266258, base → 0.0000800432}

{Null}

MAK2[X442data]

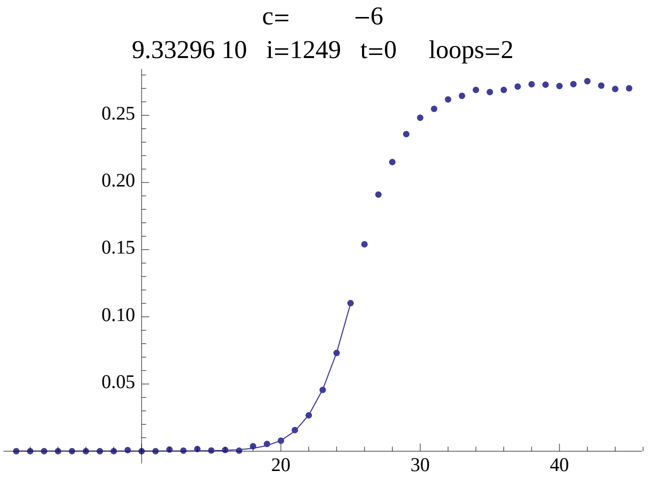

{d0 → 8.03275 × 10^{-9}, k → 0.0289249, base → 0.000078654}

{Null}

MAK2[X443data]

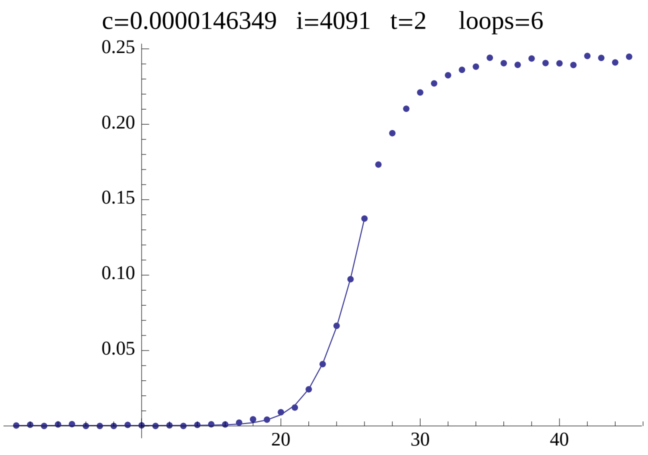

{d0 → 7.29877 × 10^{-9}, k → 0.0250262, base → 0.00029937}

{Null}

MAK2[X444data]

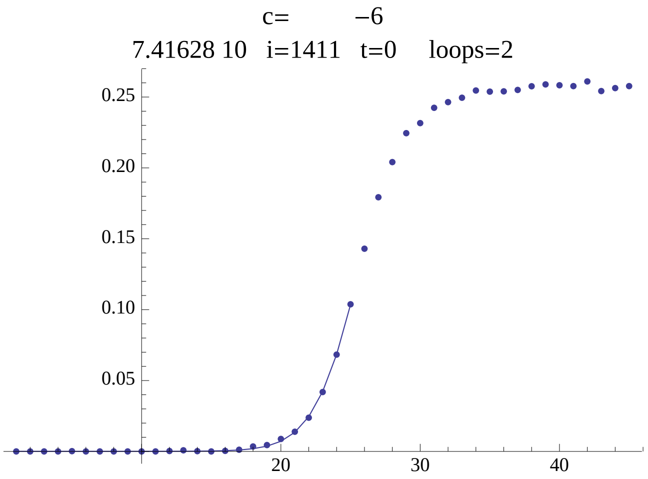

{d0 → 7.18654 × 10^{-9}, k → 0.0290718, base → 0.0000750314}

{Null}

MAK2[X451data]

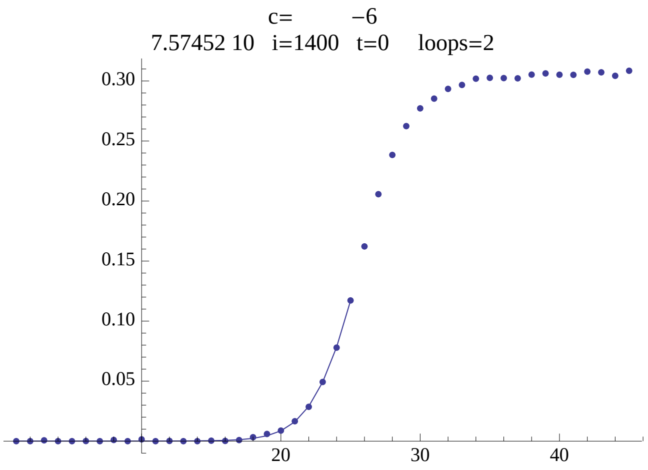

{d0 → 8.72372 × 10^-9, k → 0.03015, base → 0.0000807904}

{Null}

MAK2[X452data]

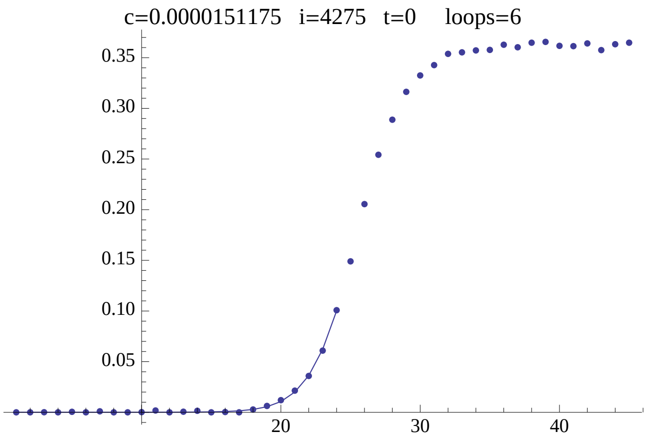

{d0 → 1.07316 × 10^-8, k → 0.0411241, base → 0.0000768858}

{Null}

MAK2[X453data]

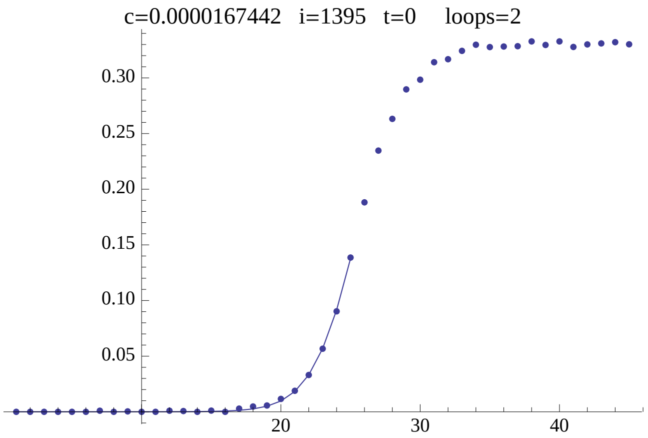

{d0 → 9.82303 × 10^-9, k → 0.037433, base → 0.0000763102}

{Null}

MAK2[X454data]

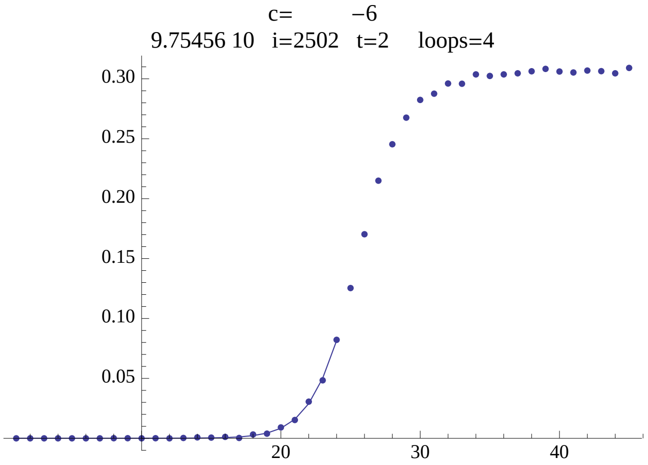

{d0 → 8.40227 × 10^-9, k → 0.0359267, base → 0.0000764992}

{Null}

MAK2[X511data]

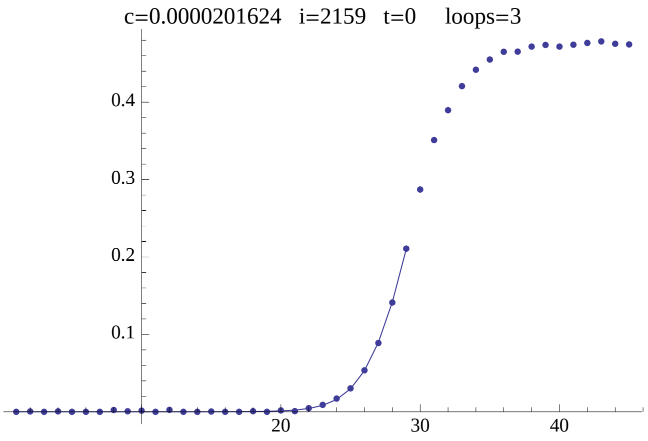

{d0 → 1.01817 × 10^{-9}, k → 0.051871, base → 0.000109388}

{Null}

MAK2[X512data]

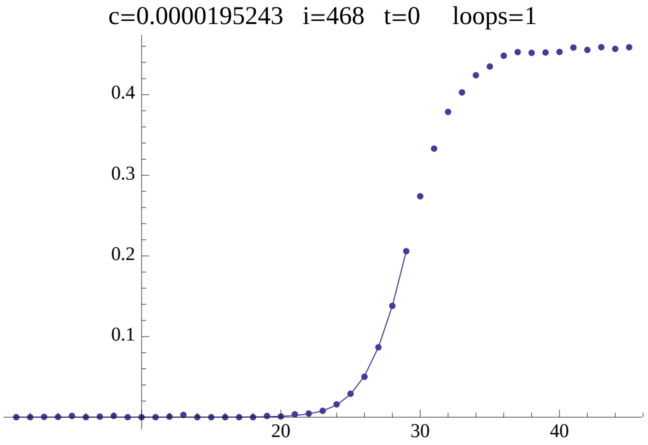

{d0 → 9.64079 × 10^{-10}, k → 0.0527627, base → 0.0000358996}

{Null}

MAK2[X513data]

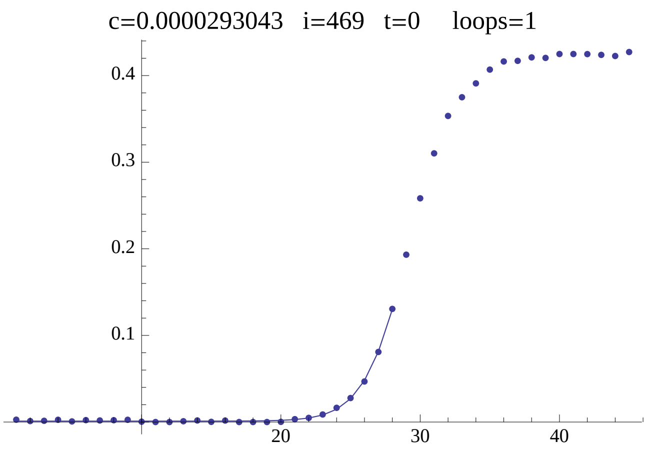

{d0 → 8.73125 × 10^{-10}, k → 0.0520975, base → 0.00102143}

{Null}

MAK2[X514data]

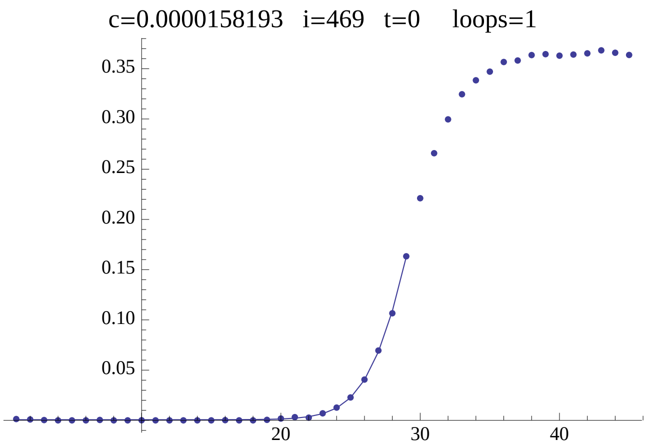

{d0 → 7.42546 × 10^{-10}, k → 0.0426781, base → 0.00060821}

{Null}

MAK2[X521data]

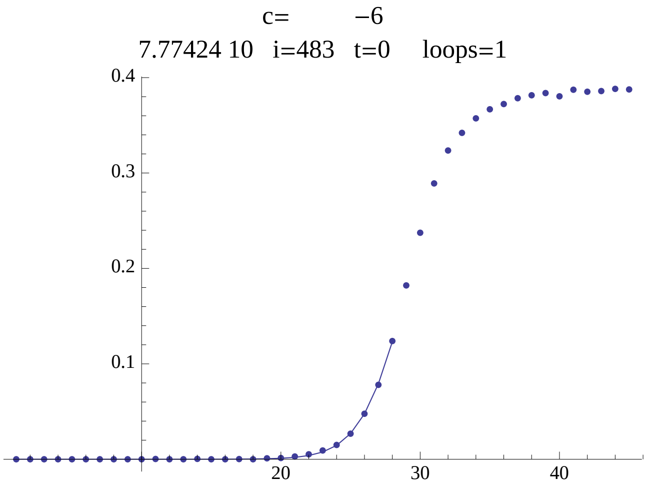

{d0 → 9.43437 × 10^{-10}, k → 0.0408623, base → 0.0000404776}

{Null}

MAK2[X522data]

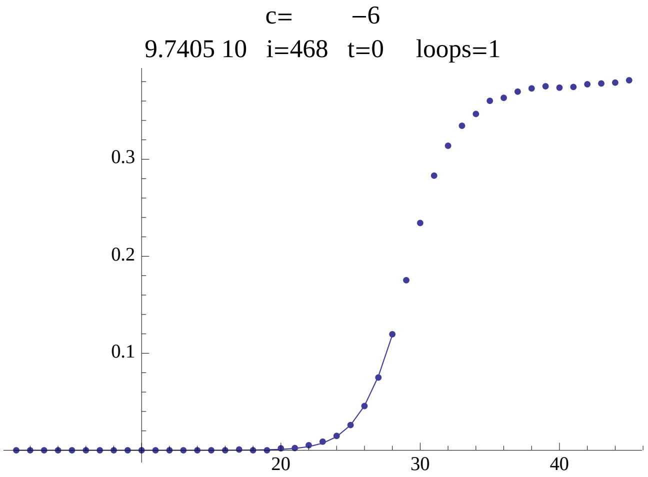

{d0 → 9.07201 × 10^{-10}, k → 0.0395968, base → 0.0000242696}

{Null}

MAK2[X523data]

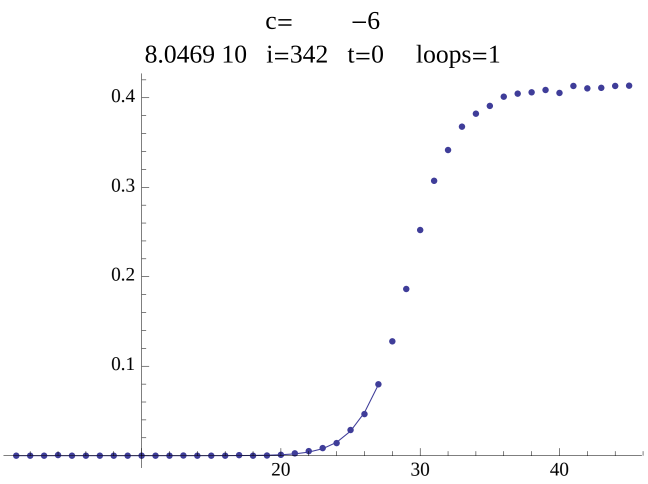

{d0 → 9.7828 × 10^-10, k → 0.0382294, base → 0.0000158112}

{Null}

MAK2[X524data]

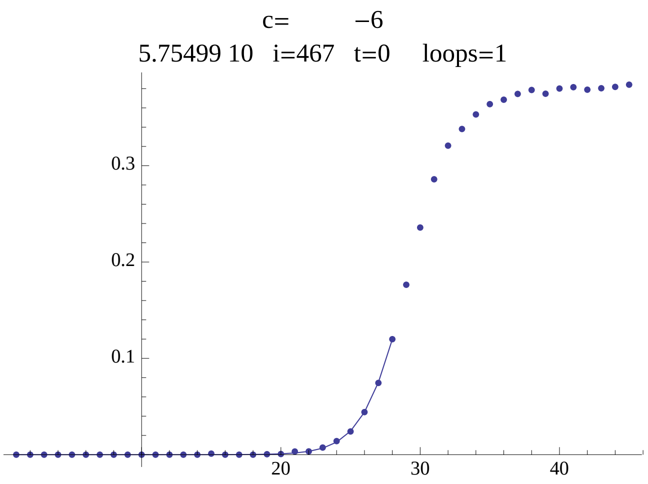

{d0 → 8.31151 × 10^-10, k → 0.0463062, base → 0.0000253395}

{Null}

MAK2[X531data]

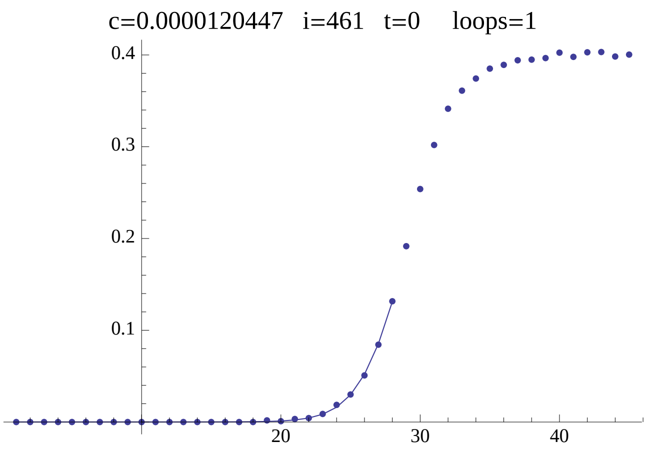

{d0 → 1.05911 × 10^{-9}, k → 0.0404126, base → 0.0000428602}

{Null}

MAK2[X532data]

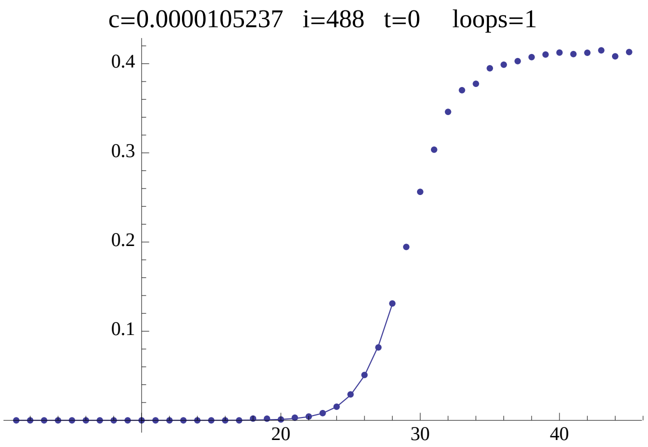

{d0 → 9.87064 × 10^{-10}, k → 0.044004, base → 0.0000437685}

{Null}

MAK2[X533data]

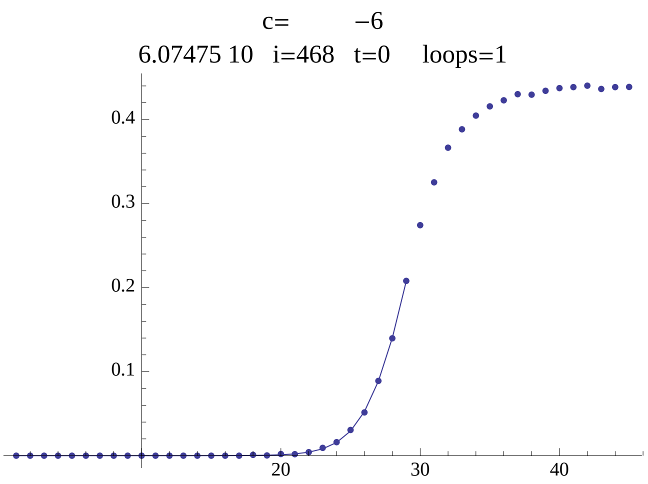

{d0 → 1.00714 × 10^{-9}, k → 0.0512414, base → 0.0000369569}

{Null}

MAK2[X534data]

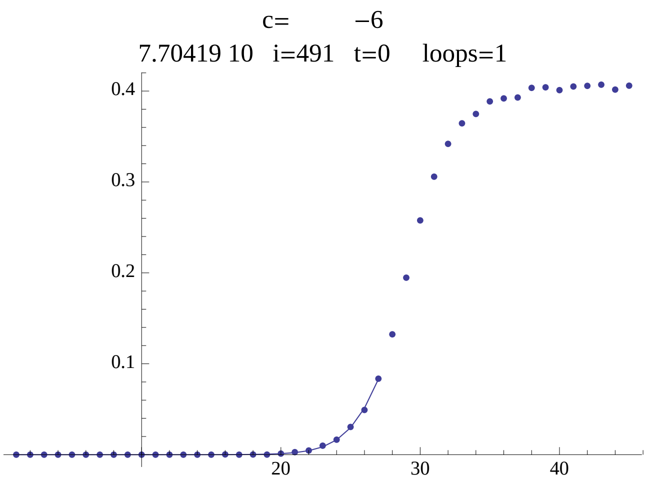

{d0 → 1.08795 × 10^{-9}, k → 0.0354791, base → −4.64611 × 10^{-6}}

{Null}

MAK2[X541data]

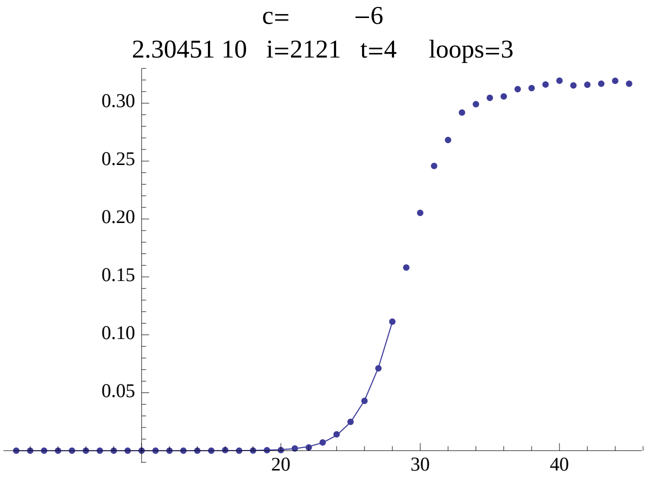

{d0 → 8.66032 × 10^{-10}, k → 0.0360034, base → −0.0000174536}

{Null}

MAK2[X542data]

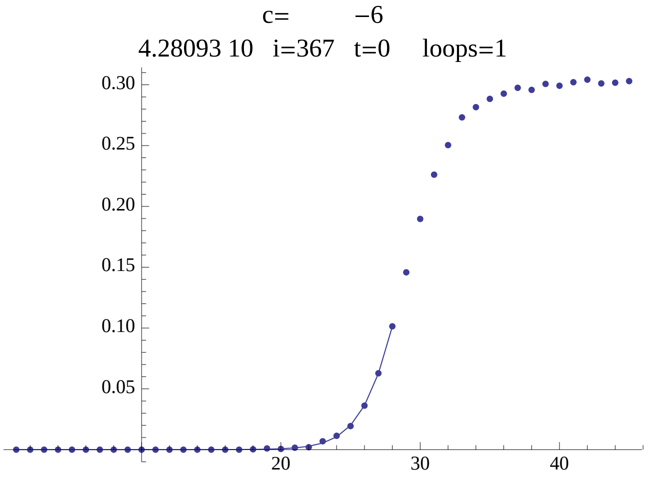

{d0 → 6.66163 × 10^{-10}, k → 0.0429498, base → 0.0000453044}

{Null}

MAK2[X543data]

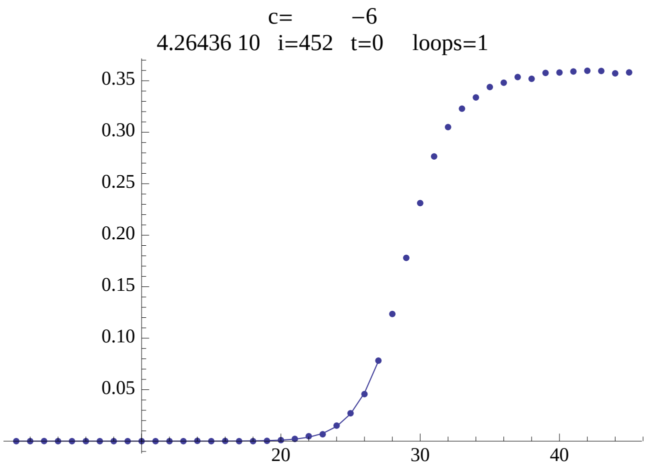

{d0 → 9.3136 × 10^{-10}, k → 0.0396673, base → 0.0000202784}

{Null}

MAK2[X544data]

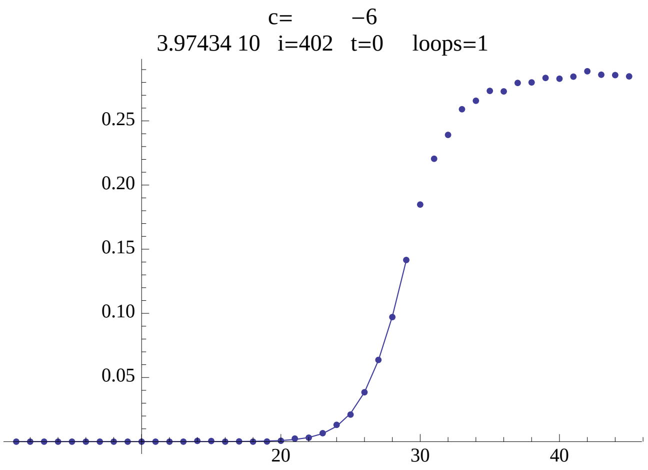

{d0 → 7.81348 × 10^{-10}, k → 0.0303894, base → 0.0000580525}

{Null}

MAK2[X551data]

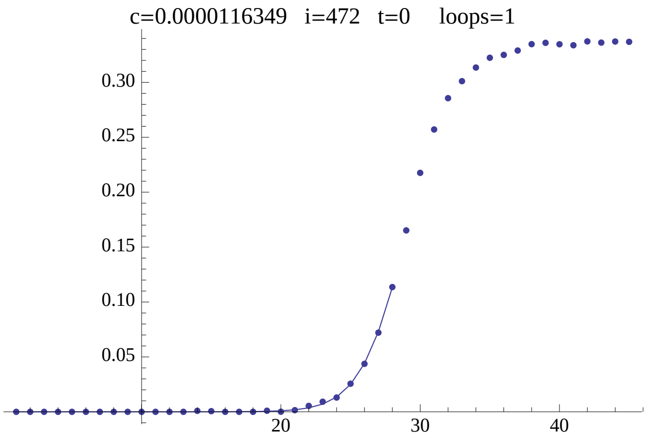

{d0 → 8.78468 × 10^{-10}, k → 0.0369423, base → 0.0000289688}

{Null}

MAK2[X552data]

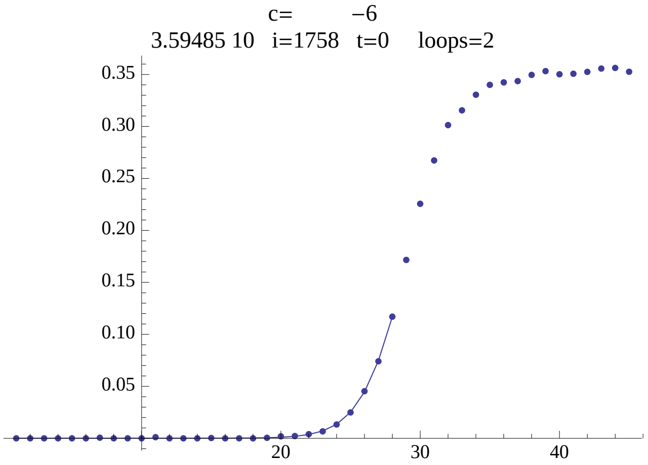

{d0 → 8.68859 × 10^{-10}, k → 0.0405325, base → 0.00011}

{Null}

MAK2[X553data]

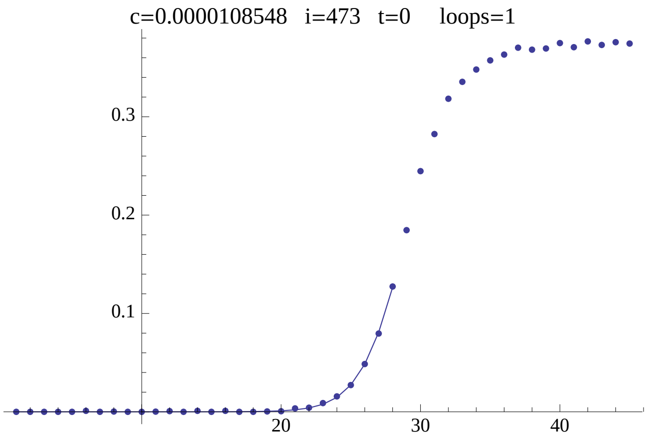

{d0 → 9.49239 × 10^{-10}, k → 0.0433327, base → 0.0000393072}

{Null}

MAK2[X554data]

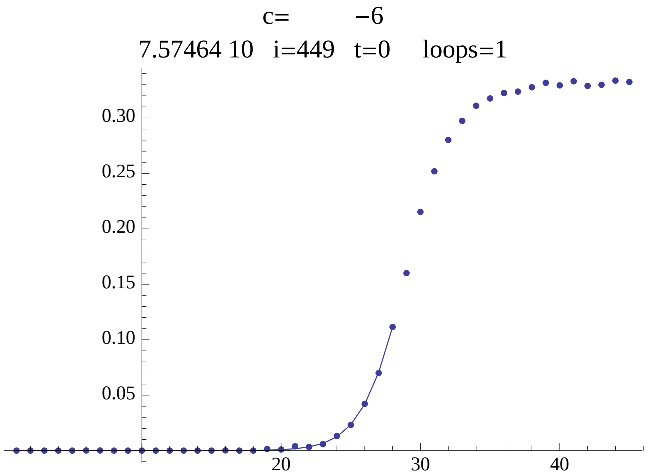

{d0 → 8.0202 × 10^{-10}, k → 0.0406658, base → 0.0000293812}

{Null}

MAK2[X611data]

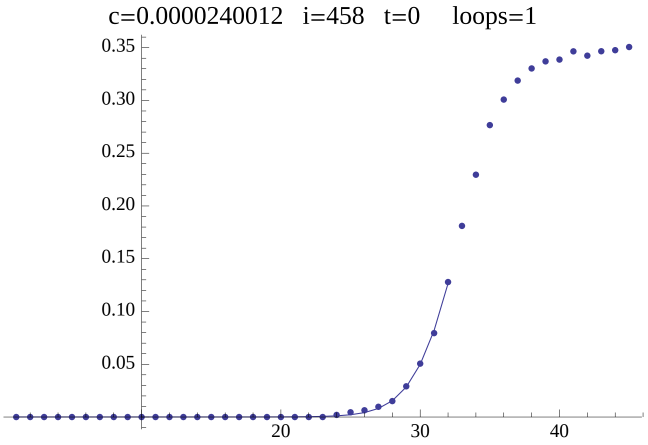

{d0 → 6.31701 × 10^{-11}, k → 0.039586, base → 0.0000581207}

{Null}

MAK2[X612data]

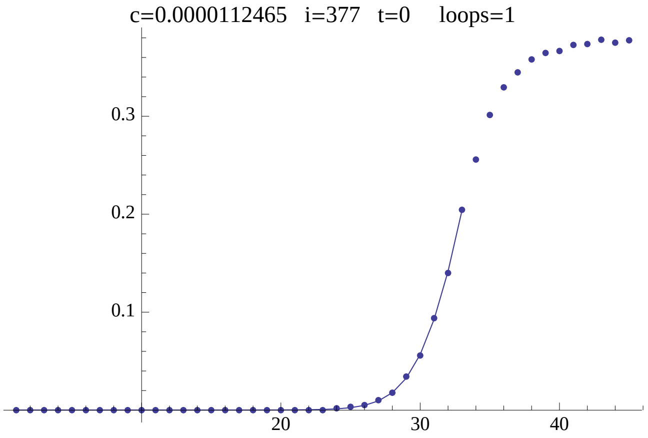

{d0 → 7.35119 × 10^{-11}, k → 0.0421222, base → 0.0000805136}

{Null}

MAK2[X613data]

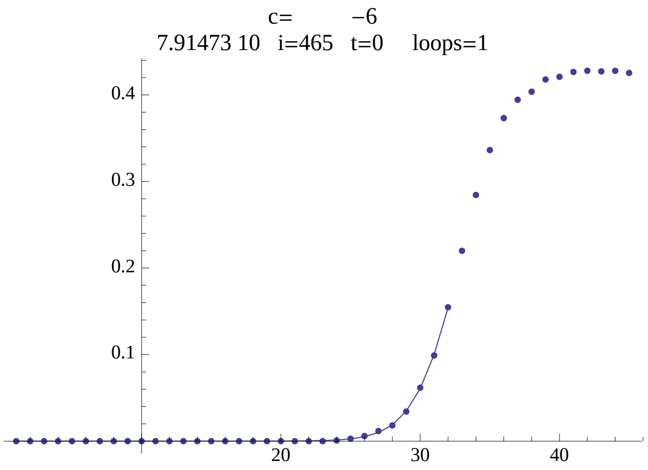

{d0 → 7.71399 × 10^-11, k → 0.0480589, base → 0.0000440631}

{Null}

MAK2[X614data]

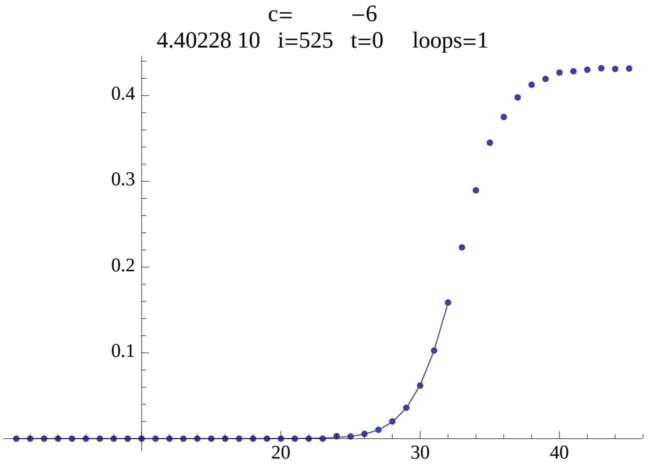

{d0 → 7.89714 × 10^-11, k → 0.0497116, base → 0.0000593716}

{Null}

MAK2[X621data]

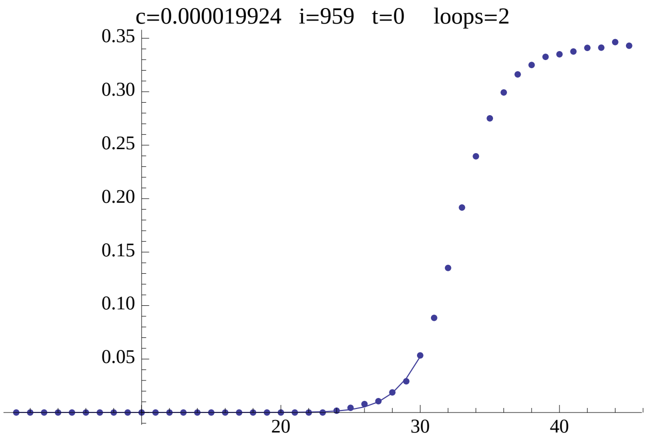

{d0 → 8.14092 × 10^-11, k → 0.0245, base → 0.0000735998}

{Null}

MAK2[X622data]

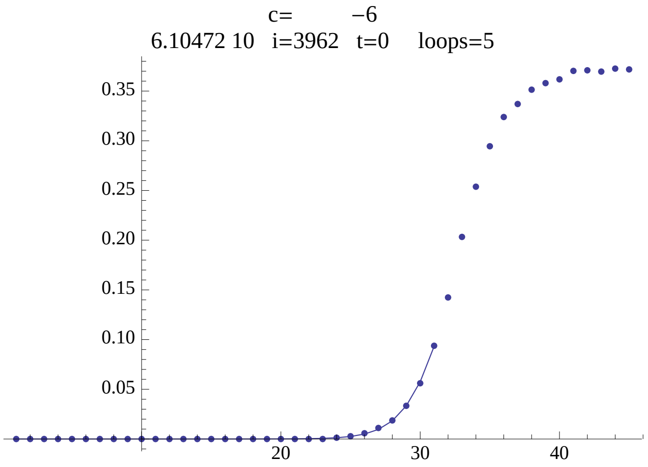

{d0 → 7.64004 × 10^-11, k → 0.0398124, base → 0.0000127666}

{Null}

MAK2[X623data]

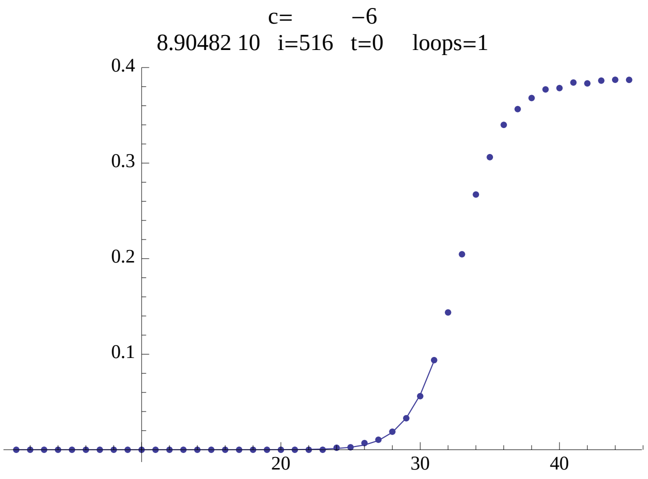

{d0 → 7.54947 × 10^{-11}, k → 0.0404688, base → 0.0000892971}

{Null}

MAK2[X624data]

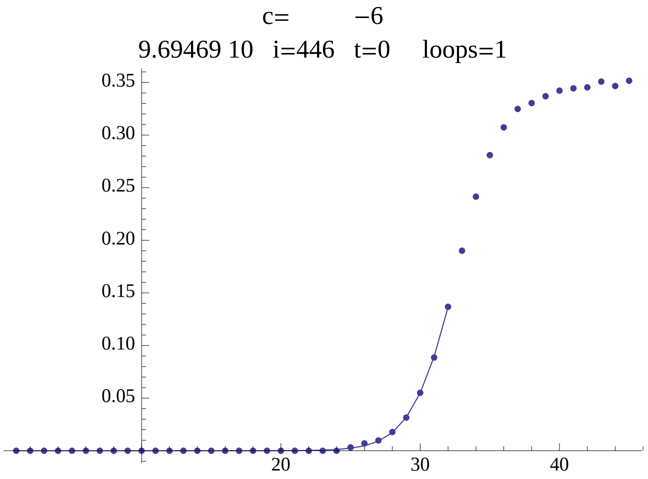

{d0 → 7.16146 × 10^{-11}, k → 0.039938, base → 0.0000305108}

{Null}

MAK2[X631data]

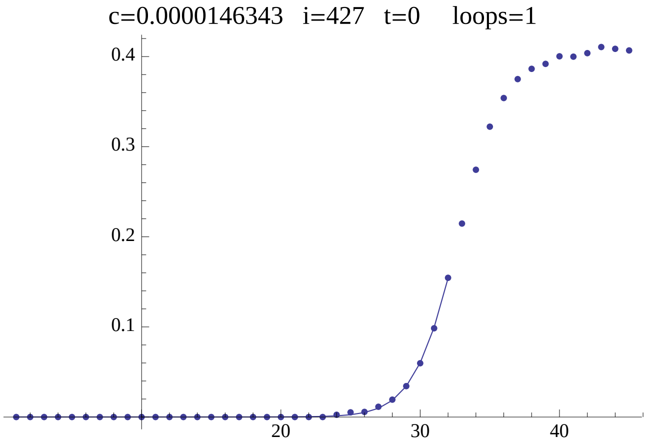

{d0 → 7.5387 × 10^-11, k → 0.0494609, base → 0.0000530316}

{Null}

MAK2[X632data]

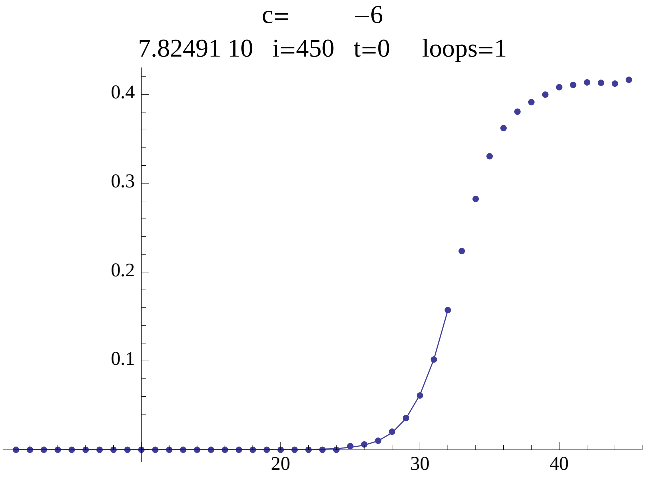

{d0 → 7.84571 × 10^-11, k → 0.048957, base → 0.0000569023}

{Null}

MAK2[X633data]

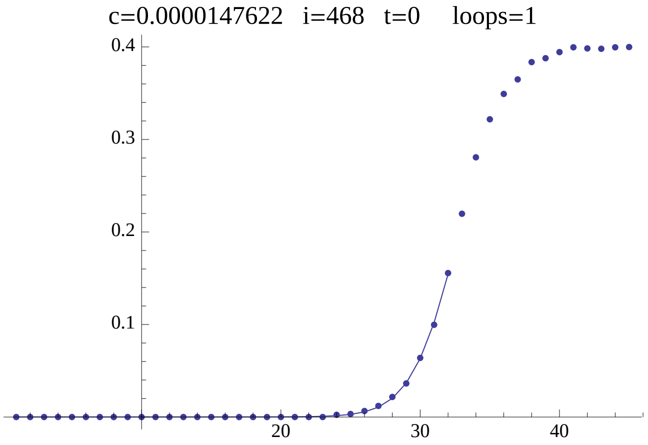

{d0 → 8.43425 × 10^{-11}, k → 0.042922, base → 0.0000607115}

{Null}

MAK2[X634data]

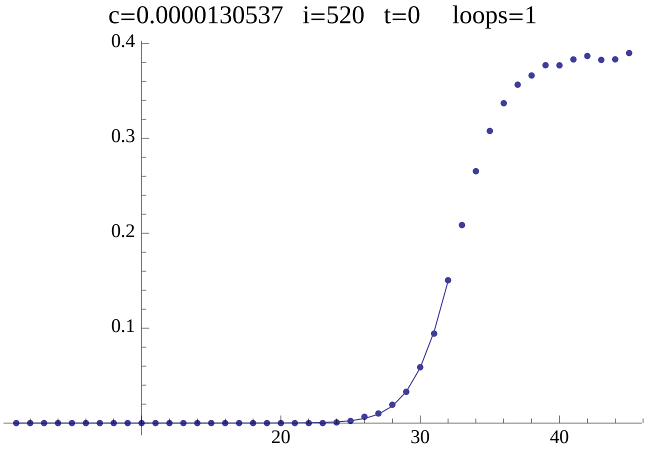

{d0 → 7.27038 × 10^{-11}, k → 0.0484093, base → 0.0000198556}

{Null}

MAK2[X641data]

c= −6
5.7062 10 i=2629 t=0 loops=4

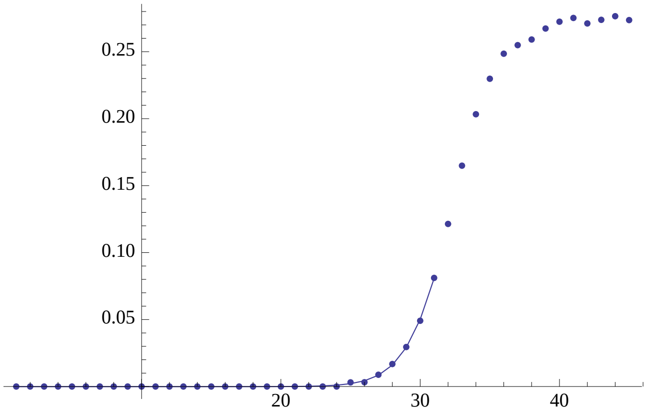

{d0 → 6.77079 × 10^{-11}, k → 0.0332395, base → − 0.00010404}

{Null}

MAK2[X642data]

c= −6
2.81491 10 i=5688 t=6 loops=6

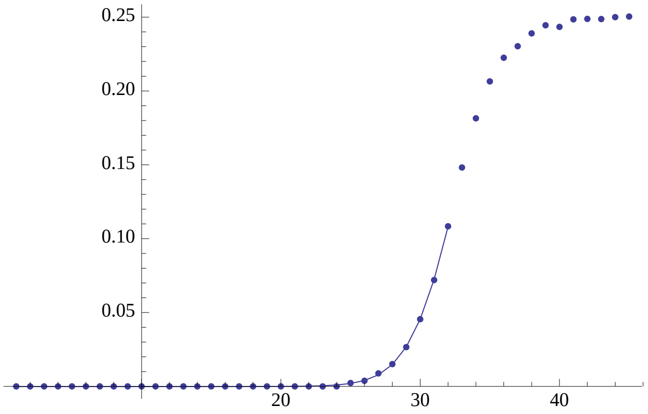

{d0 → 6.34769 × 10^{-11}, k → 0.0277117, base → − 0.00010999}

{Null}

MAK2[X643data]

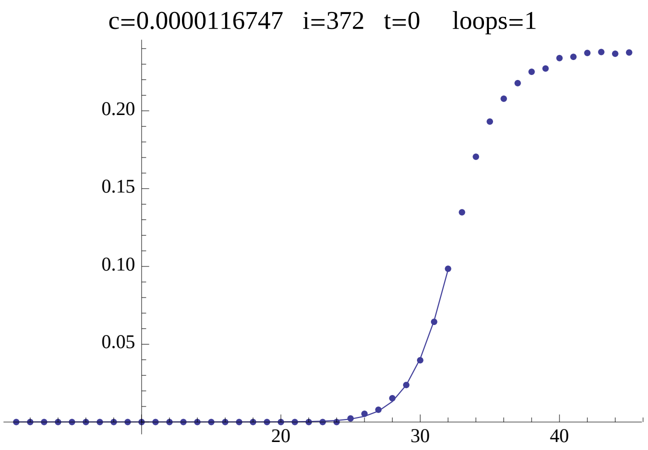

{d0 → 5.5191 × 10^{-11}, k → 0.0261687, base → 0.0000821592}

{Null}

MAK2[X644data]

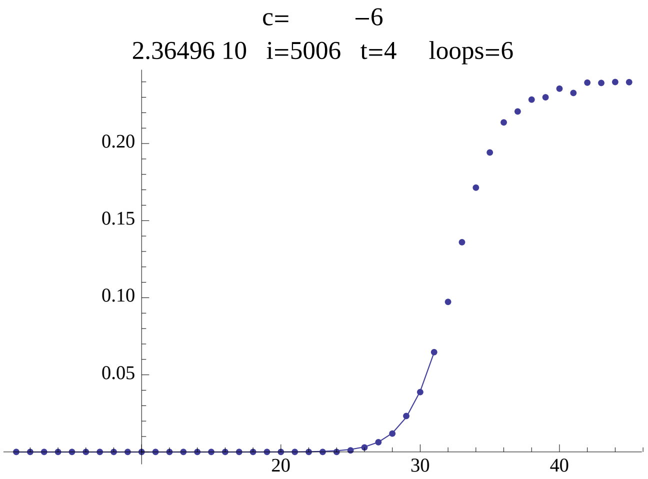

{d0 → 5.03784 × 10^{-11}, k → 0.0303454, base → -0.0000646715}

{Null}

MAK2[X651data]

c= −6
4.37457 10 i=2608 t=0 loops=4

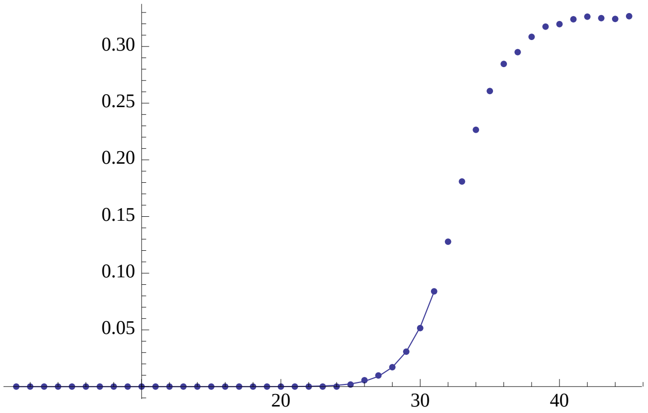

{d0 → 7.25606 × 10^{-11}, k → 0.0325295, base → -0.0000738649}

{Null}

MAK2[X652data]

c= −6
8.53483 10 i=4484 t=4 loops=7

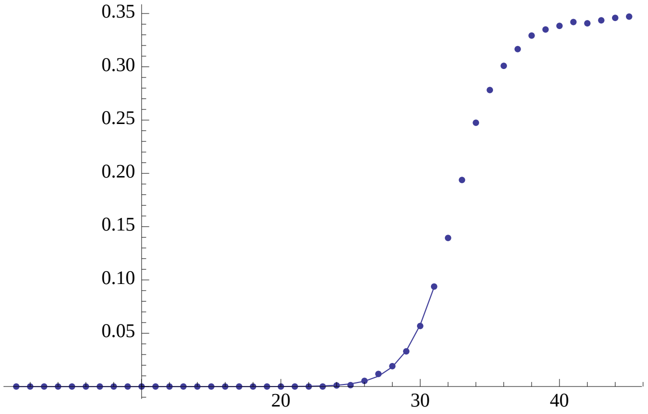

{d0 → 7.73279 × 10^{-11}, k → 0.0391158, base → -0.0000802227}

{Null}

MAK2[X653data]

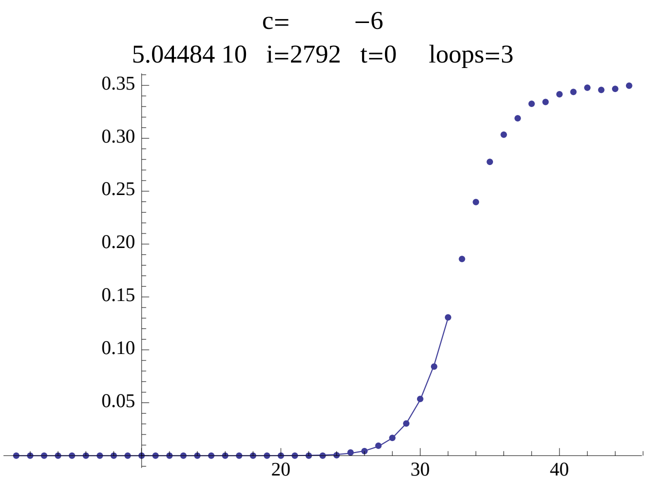

{d0 → 6.94069 × 10^-11, k → 0.0373462, base → -0.0000350165}

{Null}

MAK2[X654data]

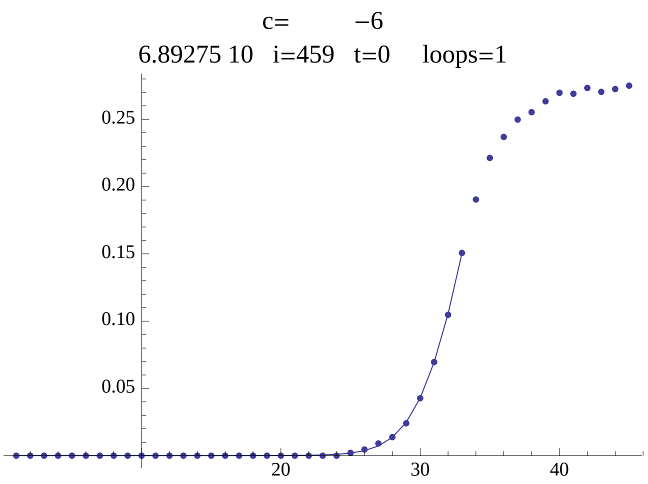

{d0 → 5.66489 × 10^-11, k → 0.0298845, base → 0.0000617769}

{Null}

The following are data and trials from the 'reps.csv' dataset.

```
indata = Import["reps.csv"];
cycle = indata[[All, 1]][[2 ;;]];

F11data = Transpose[{cycle, indata[[All, 3]][[2 ;;]]}];
F12data = Transpose[{cycle, indata[[All, 4]][[2 ;;]]}];
F13data = Transpose[{cycle, indata[[All, 5]][[2 ;;]]}];
F14data = Transpose[{cycle, indata[[All, 6]][[2 ;;]]}];
F21data = Transpose[{cycle, indata[[All, 7]][[2 ;;]]}];
F22data = Transpose[{cycle, indata[[All, 8]][[2 ;;]]}];
F23data = Transpose[{cycle, indata[[All, 9]][[2 ;;]]}];
F24data = Transpose[{cycle, indata[[All, 10]][[2 ;;]]}];
F31data = Transpose[{cycle, indata[[All, 11]][[2 ;;]]}];
F32data = Transpose[{cycle, indata[[All, 12]][[2 ;;]]}];
F33data = Transpose[{cycle, indata[[All, 13]][[2 ;;]]}];
F34data = Transpose[{cycle, indata[[All, 14]][[2 ;;]]}];
F41data = Transpose[{cycle, indata[[All, 15]][[2 ;;]]}];
F42data = Transpose[{cycle, indata[[All, 16]][[2 ;;]]}];
F43data = Transpose[{cycle, indata[[All, 17]][[2 ;;]]}];
F44data = Transpose[{cycle, indata[[All, 18]][[2 ;;]]}];
F51data = Transpose[{cycle, indata[[All, 19]][[2 ;;]]}];
F52data = Transpose[{cycle, indata[[All, 20]][[2 ;;]]}];
F53data = Transpose[{cycle, indata[[All, 21]][[2 ;;]]}];
F54data = Transpose[{cycle, indata[[All, 22]][[2 ;;]]}];
F61data = Transpose[{cycle, indata[[All, 23]][[2 ;;]]}];
F62data = Transpose[{cycle, indata[[All, 24]][[2 ;;]]}];
F63data = Transpose[{cycle, indata[[All, 25]][[2 ;;]]}];
F64data = Transpose[{cycle, indata[[All, 26]][[2 ;;]]}];
F71data = Transpose[{cycle, indata[[All, 27]][[2 ;;]]}];
F72data = Transpose[{cycle, indata[[All, 28]][[2 ;;]]}];
F73data = Transpose[{cycle, indata[[All, 29]][[2 ;;]]}];
F74data = Transpose[{cycle, indata[[All, 30]][[2 ;;]]}];

MAK2[F11data]
```

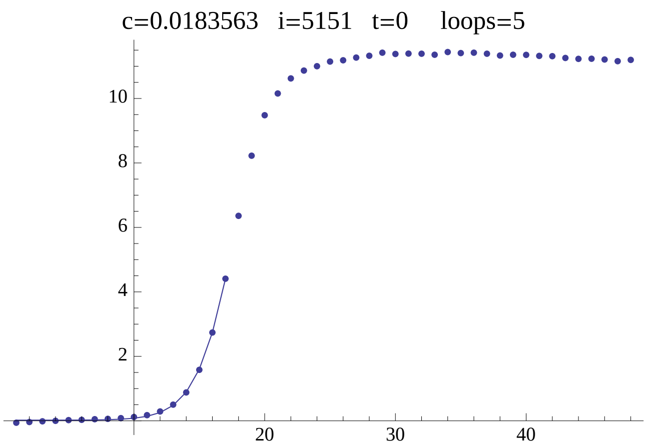

{d0 → 0.0000598625, k → 1.81921, base → 0.0195575}

{Null}

MAK2[F12data]

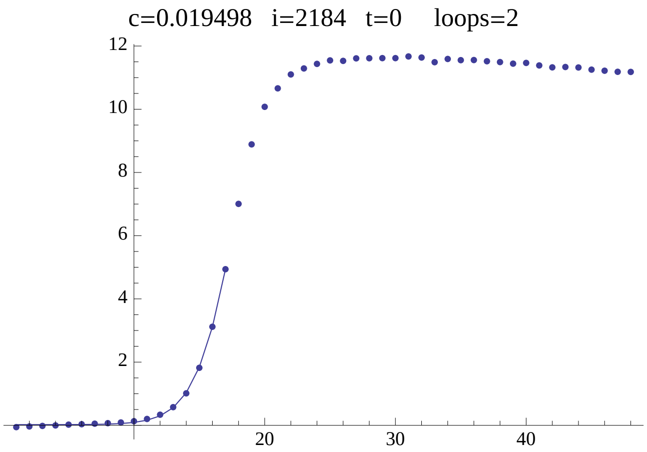

{d0 → 0.0000705565, k → 1.8739, base → 0.0203246}

{Null}

MAK2[F13data]

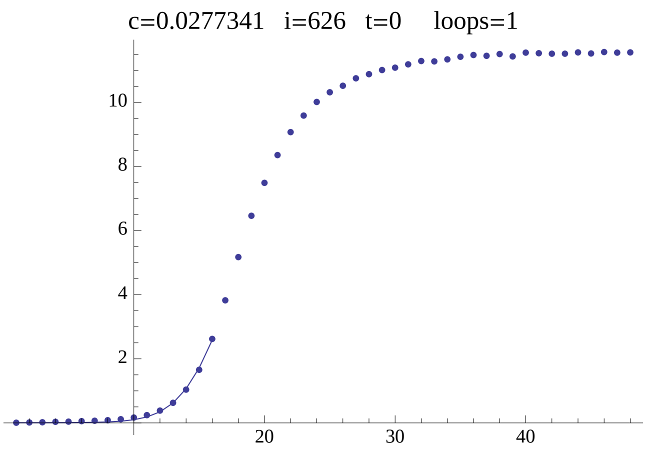

{d0 → 0.0000930618, k → 0.712197, base → 0.00662347}

{Null}

MAK2[F14data]

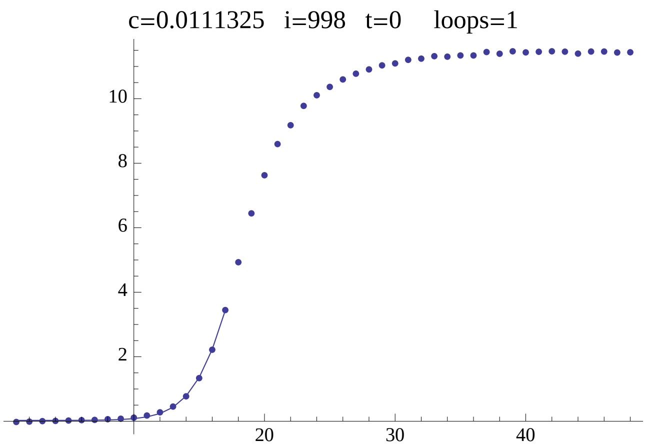

{d0 → 0.0000546615, k → 1.10039, base → 0.0252435}

{Null}

MAK2[F21data]

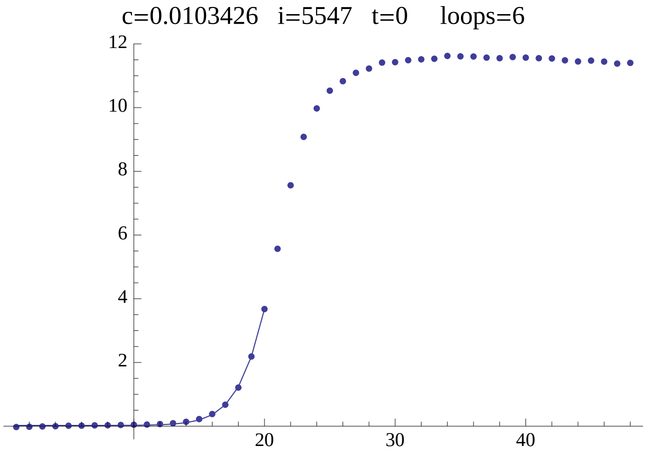

{d0 → 5.34387 × 10^{-6}, k → 2.07584, base → 0.0224431}

{Null}

MAK2[F22data]

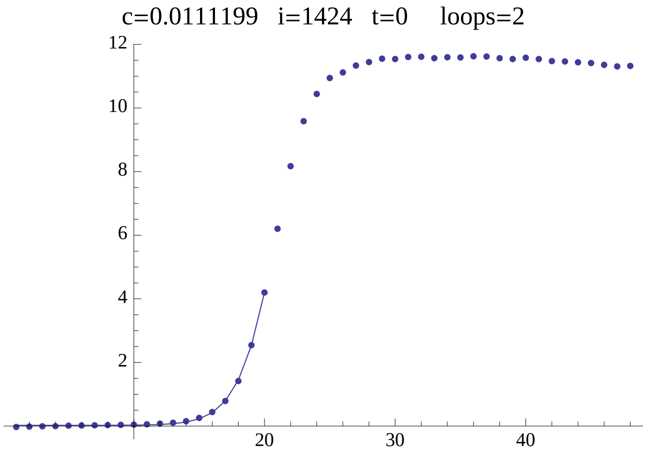

{d0 → 6.39456 × 10^{-6}, k → 2.14373, base → 0.0219707}

{Null}

MAK2[F23data]

c=0.0209377   i=5002   t=4   loops=4

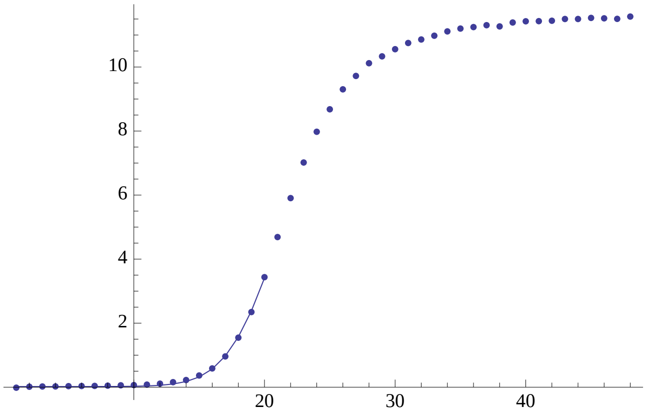

{d0 → 0.0000102571, k → 0.686167, base → 0.0175253}

{Null}

MAK2[F24data]

c=0.0220832   i=3096   t=0   loops=3

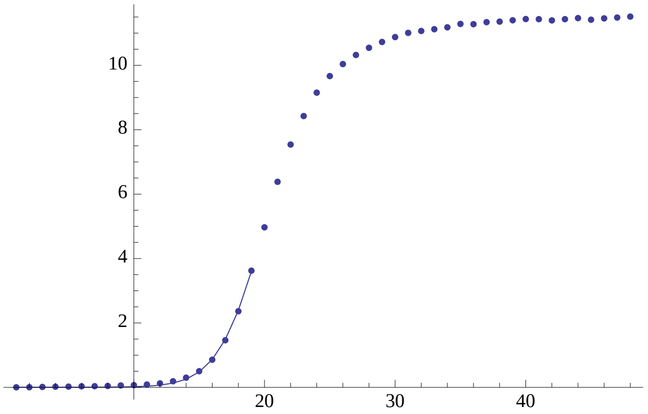

{d0 → 0.0000163518, k → 0.985408, base → 0.00281425}

{Null}

MAK2[F31data]

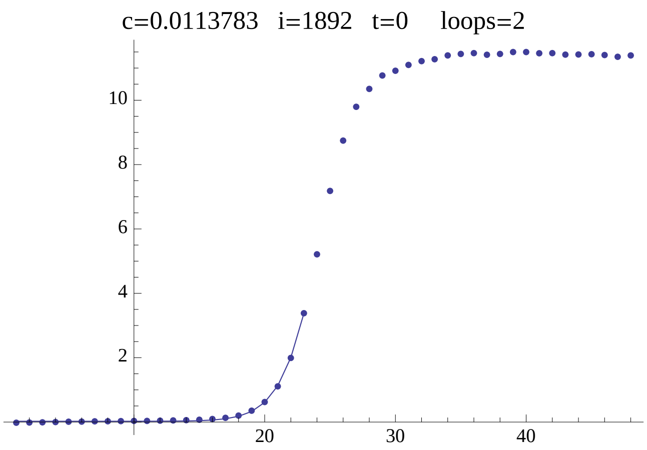

{d0 → 6.04607 × 10^-7, k → 1.9737, base → 0.0232698}

{Null}

MAK2[F32data]

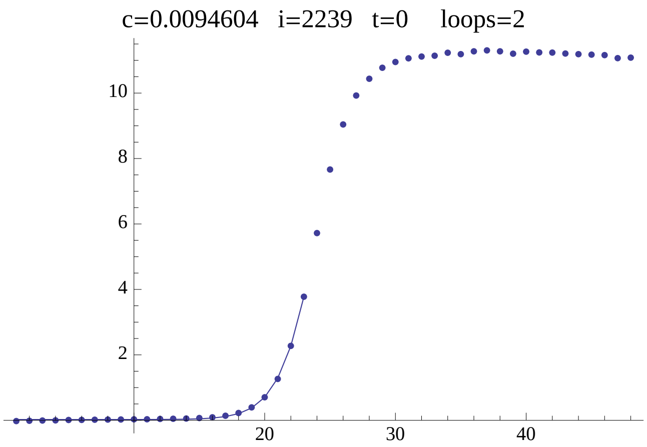

{d0 → 7.06169 × 10^-7, k → 1.99994, base → 0.022778}

{Null}

MAK2[F33data]

c=0.0311595   i=4027   t=5   loops=4

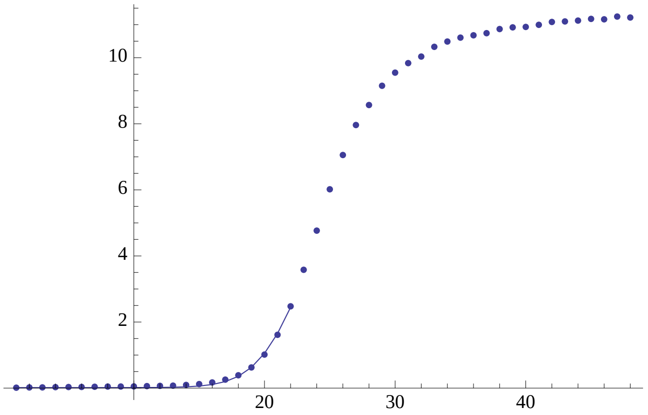

{d0 → 1.50674 × 10^{-6}, k → 0.601586, base → 0.0134873}

{Null}

MAK2[F34data]

c=0.0358389   i=1955   t=0   loops=2

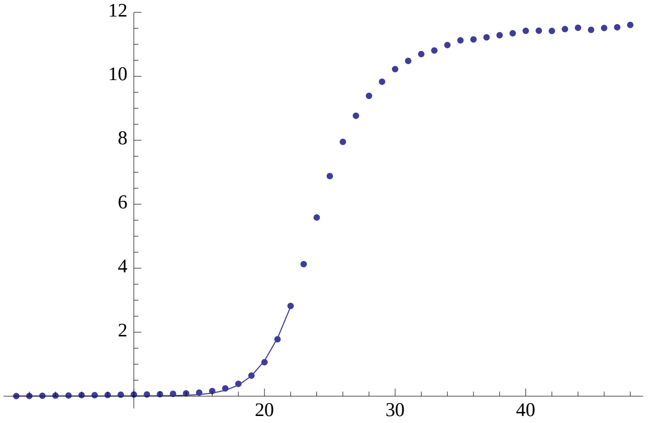

{d0 → 1.43264 × 10^{-6}, k → 0.868825, base → 0.00347211}

{Null}

MAK2[F41data]

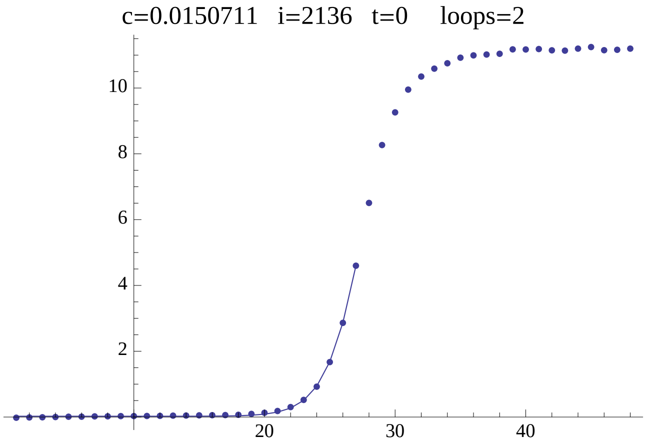

$\{d0 \to 6.1472 \times 10^{-8},\ k \to 1.86829,\ base \to 0.0232914\}$

{Null}

MAK2[F42data]

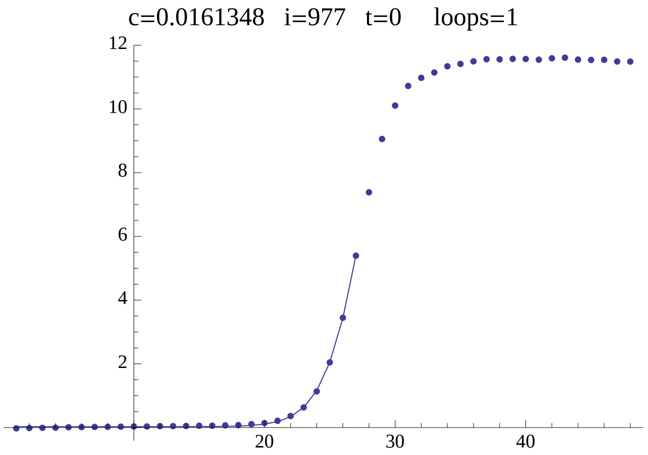

$\{d0 \to 7.80635 \times 10^{-8},\ k \to 1.9315,\ base \to 0.0274261\}$

{Null}

MAK2[F43data]

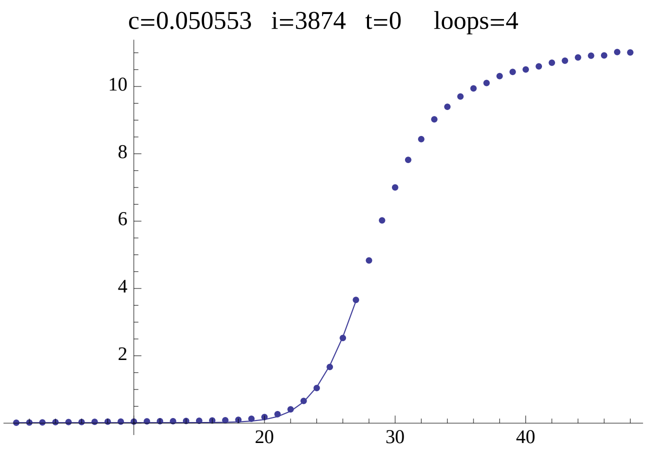

{d0 → 9.203 × 10^{-8}, k → 0.683405, base → 0.013557}

{Null}

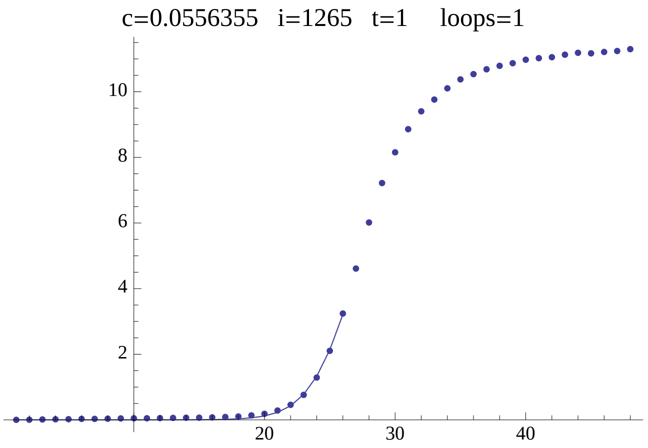

{d0 → 1.15039 × 10^{-7}, k → 0.867647, base → 0.00217796}

MAK2[F51data]

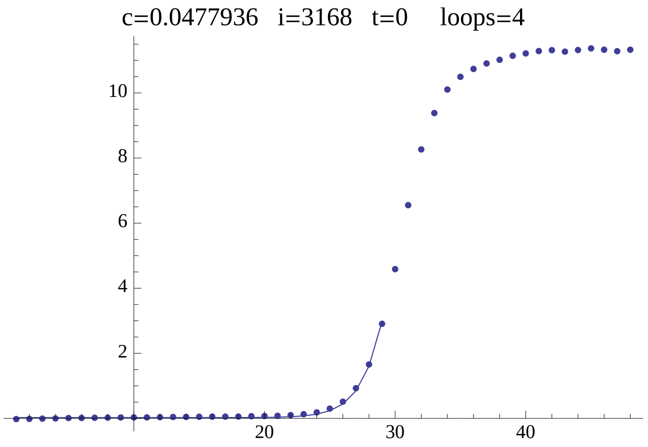

{d0 → 6.38367 × 10^{-9}, k → 4.97693, base → 0.0231121}

{Null}

MAK2[F52data]

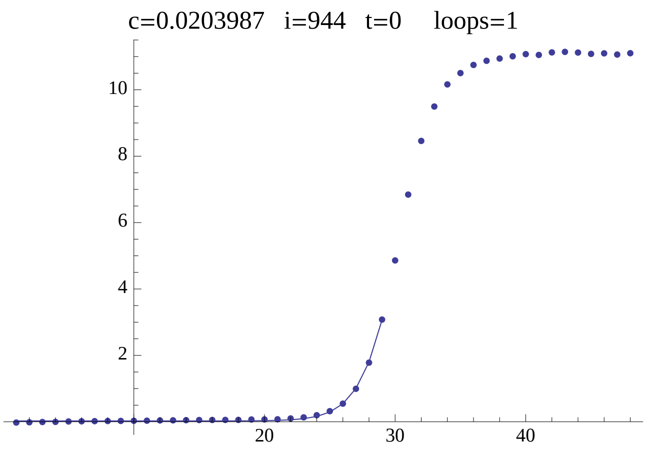

{d0 → 8.23303 × 10^-9, k → 1.99813, base → 0.025555}

{Null}

MAK2[F53data]

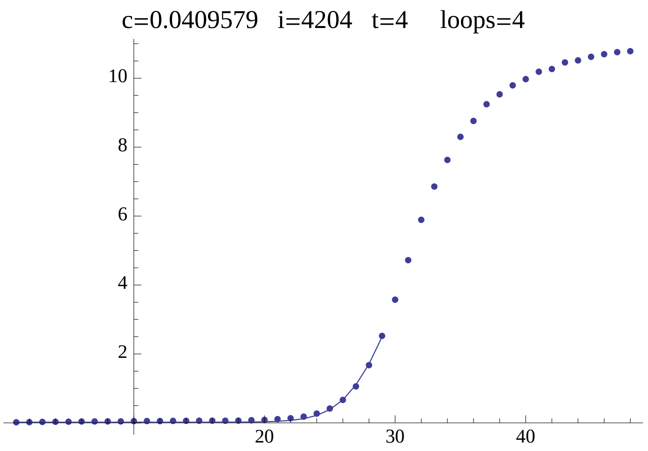

{d0 → 1.28184 × 10^-8, k → 0.569539, base → 0.0192616}

{Null}

MAK2[F54data]

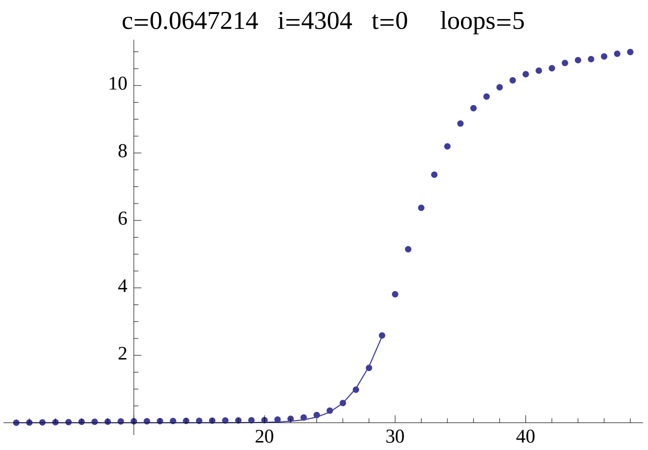

{d0 → 1.04103 × 10^{-8}, k → 0.782183, base → 0.00188566}

{Null}

MAK2[F61data]

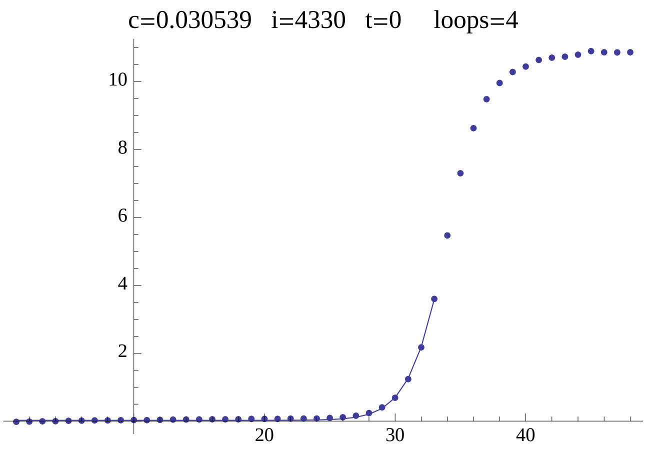

{d0 → 6.85304 × 10^{-10}, k → 1.72884, base → 0.0226451}

{Null}

MAK2[F62data]

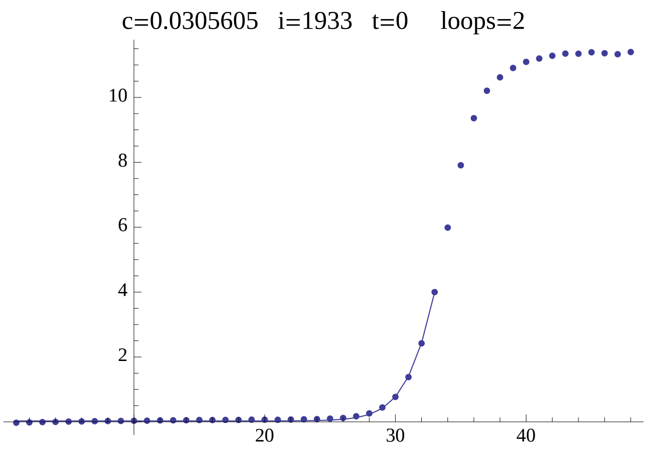

{d0 → 7.61309 × 10^{-10}, k → 1.92027, base → 0.0287448}

{Null}

MAK2[F63data]

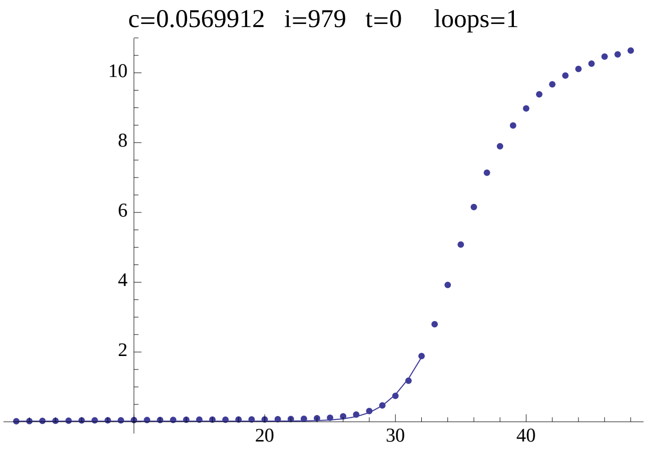

{d0 → 1.03567 × 10^{-9}, k → 0.48951, base → 0.0184934}

{Null}

MAK2[F64data]

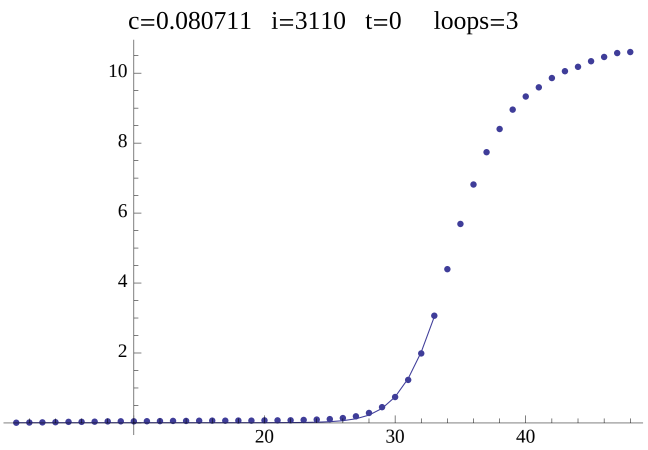

{d0 → 8.62968 × 10^-10, k → 0.802417, base → 0.00750668}

{Null}

MAK2[F71data]

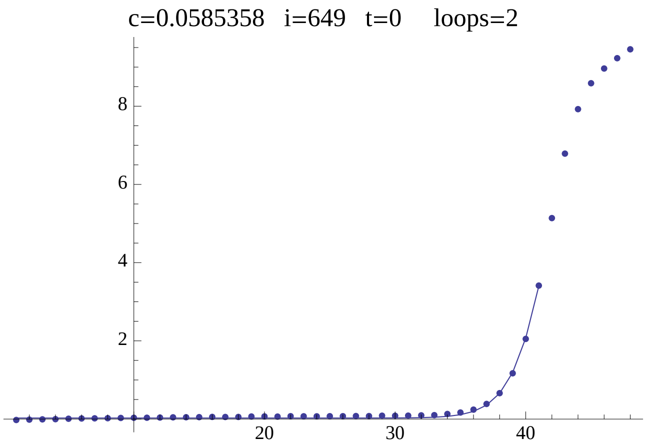

{d0 → 2.53156 × 10^-12, k → 1.63562, base → 0.0257086}

{Null}

MAK2[F72data]

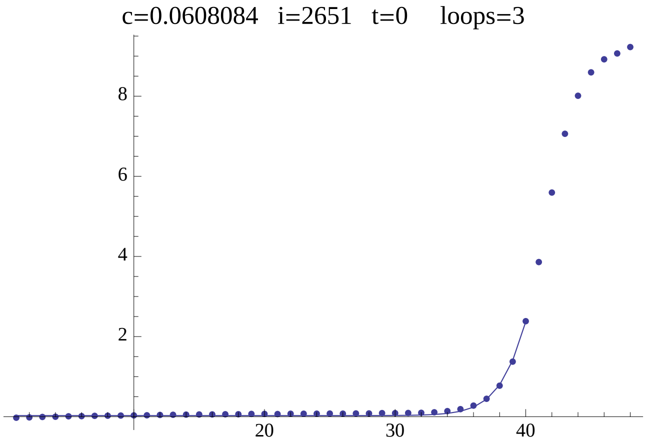

{d0 → 3.17764 × 10^{-12}, k → 1.42958, base → 0.0279425}

{Null}

MAK2[F73data]

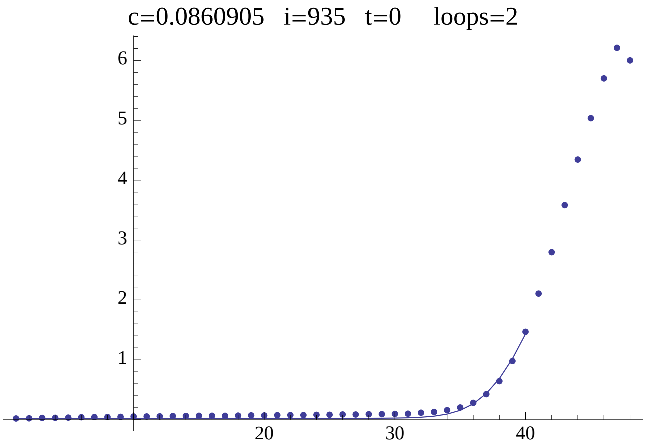

{d0 → 4.43143 × 10^{-12}, k → 0.264402, base → 0.0220891}

{Null}

MAK2[F74data]

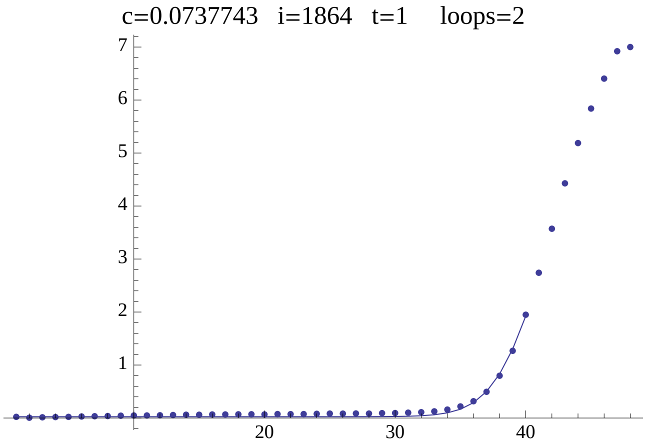

{d0 → 4.44709 × 10^{-12}, k → 0.474396, base → 0.0240224}

{Null}
