# Supplementary material for: A Mechanistic Model of PCR for Accurate Quantification of Quantitative PCR Data
Source: PLoS One. 2010 Aug 30;5(8):e12355. doi: 10.1371/journal.pone.0012355 (PMC2930010; doi:10.1371/journal.pone.0012355)
Supplement: Dataset S3 — Cq values obtained for the qPCR data. These Cq values were used in generating figure 3. (0.11 MB PDF) [file pone.0012355.s006.pdf]

```
(*****************************************************************************************)
(*  This Mathematica notebook contains code for calculating the quantification cycle from qPCR data and   *)
(*        results of these calculations.                                                                    *)
(*  The 'd0' values resulting from these trials are used to develop the plots in figure 3 of the MAK2 paper. *)
(*                                                                                                          *)
(******                     Written by Gregory Boggy (November 2009)                               *****)
(*****************************************************************************************)
```

```
CTRoutine[data_, signal_] :=
  {For[i = Position[Abs[0.5 * Max[data[[All, 2]]] - data[[All, 2]]],
        Min[Abs[0.5 * Max[data[[All, 2]]] - data[[All, 2]]]]][[1]][[
      1]], data[[i, 2]] > data[[i - 1, 2]], i--];
   base = Mean[data[[1 ;; i, 2]]];
   For[j = i + 1, data[[j, 2]] - base <= signal, j++];
   j - 1 + (signal + base - data[[j - 1, 2]]) / (data[[j, 2]] - data[[j - 1, 2]])
  }[[1]];

filepath = "Desktop/supplement/";
SetDirectory[filepath];
indata = Import["growth.csv"];
cycle = indata[[All, 1]];

D3data = Transpose[{cycle, indata[[All, 2]]}][[2 ;; 41]];
D4data = Transpose[{cycle, indata[[All, 3]]}][[2 ;; 41]];
D5data = Transpose[{cycle, indata[[All, 4]]}][[2 ;; 41]];
D6data = Transpose[{cycle, indata[[All, 5]]}][[2 ;; 41]];
D7data = Transpose[{cycle, indata[[All, 6]]}][[2 ;; 41]];
D8data = Transpose[{cycle, indata[[All, 7]]}][[2 ;; 41]];
E3data = Transpose[{cycle, indata[[All, 8]]}][[2 ;; 41]];
E4data = Transpose[{cycle, indata[[All, 9]]}][[2 ;; 41]];
E5data = Transpose[{cycle, indata[[All, 10]]}][[2 ;; 41]];
E6data = Transpose[{cycle, indata[[All, 11]]}][[2 ;; 41]];
E7data = Transpose[{cycle, indata[[All, 12]]}][[2 ;; 41]];
E8data = Transpose[{cycle, indata[[All, 13]]}][[2 ;; 41]];
```

```
CTD = {CTRoutine[D3data, .1], CTRoutine[D4data, .1], CTRoutine[D5data, .1],
  CTRoutine[D6data, .1], CTRoutine[D7data, .1], CTRoutine[D8data, .1],
  CTRoutine[E3data, .1], CTRoutine[E4data, .1], CTRoutine[E5data, .1],
  CTRoutine[E6data, .1], CTRoutine[E7data, .1], CTRoutine[E8data, .1]}
```

```
{26.4221, 22.7996, 18.9484, 15.9941, 12.2206,
 8.89905, 26.5871, 22.5262, 18.763, 15.931, 12.2648, 8.62924}
```

```
indata = Import["rutledge.csv"];
cycle = indata[[All, 1]][[2 ;;]];

X111data = Transpose[{cycle, indata[[All, 3]][[2 ;;]]}];
X112data = Transpose[{cycle, indata[[All, 4]][[2 ;;]]}];
X113data = Transpose[{cycle, indata[[All, 5]][[2 ;;]]}];
X114data = Transpose[{cycle, indata[[All, 6]][[2 ;;]]}];
X121data = Transpose[{cycle, indata[[All, 7]][[2 ;;]]}];
X122data = Transpose[{cycle, indata[[All, 8]][[2 ;;]]}];
X123data = Transpose[{cycle, indata[[All, 9]][[2 ;;]]}];
X124data = Transpose[{cycle, indata[[All, 10]][[2 ;;]]}];
X131data = Transpose[{cycle, indata[[All, 11]][[2 ;;]]}];
X132data = Transpose[{cycle, indata[[All, 12]][[2 ;;]]}];
X133data = Transpose[{cycle, indata[[All, 13]][[2 ;;]]}];
X134data = Transpose[{cycle, indata[[All, 14]][[2 ;;]]}];
X141data = Transpose[{cycle, indata[[All, 15]][[2 ;;]]}];
X142data = Transpose[{cycle, indata[[All, 16]][[2 ;;]]}];
X143data = Transpose[{cycle, indata[[All, 17]][[2 ;;]]}];
X144data = Transpose[{cycle, indata[[All, 18]][[2 ;;]]}];
X151data = Transpose[{cycle, indata[[All, 19]][[2 ;;]]}];
X152data = Transpose[{cycle, indata[[All, 20]][[2 ;;]]}];
X153data = Transpose[{cycle, indata[[All, 21]][[2 ;;]]}];
X154data = Transpose[{cycle, indata[[All, 22]][[2 ;;]]}];
X211data = Transpose[{cycle, indata[[All, 23]][[2 ;;]]}];
X212data = Transpose[{cycle, indata[[All, 24]][[2 ;;]]}];
X213data = Transpose[{cycle, indata[[All, 25]][[2 ;;]]}];
X214data = Transpose[{cycle, indata[[All, 26]][[2 ;;]]}];
X221data = Transpose[{cycle, indata[[All, 27]][[2 ;;]]}];
X222data = Transpose[{cycle, indata[[All, 28]][[2 ;;]]}];
X223data = Transpose[{cycle, indata[[All, 29]][[2 ;;]]}];
X224data = Transpose[{cycle, indata[[All, 30]][[2 ;;]]}];
X231data = Transpose[{cycle, indata[[All, 31]][[2 ;;]]}];
X232data = Transpose[{cycle, indata[[All, 32]][[2 ;;]]}];
X233data = Transpose[{cycle, indata[[All, 33]][[2 ;;]]}];
X234data = Transpose[{cycle, indata[[All, 34]][[2 ;;]]}];
X241data = Transpose[{cycle, indata[[All, 35]][[2 ;;]]}];
X242data = Transpose[{cycle, indata[[All, 36]][[2 ;;]]}];
X243data = Transpose[{cycle, indata[[All, 37]][[2 ;;]]}];
```

```
X244data = Transpose[{cycle, indata[[All, 38]][[2 ;;]]}];
X251data = Transpose[{cycle, indata[[All, 39]][[2 ;;]]}];
X252data = Transpose[{cycle, indata[[All, 40]][[2 ;;]]}];
X253data = Transpose[{cycle, indata[[All, 41]][[2 ;;]]}];
X254data = Transpose[{cycle, indata[[All, 42]][[2 ;;]]}];
X311data = Transpose[{cycle, indata[[All, 43]][[2 ;;]]}];
X312data = Transpose[{cycle, indata[[All, 44]][[2 ;;]]}];
X313data = Transpose[{cycle, indata[[All, 45]][[2 ;;]]}];
X314data = Transpose[{cycle, indata[[All, 46]][[2 ;;]]}];
X321data = Transpose[{cycle, indata[[All, 47]][[2 ;;]]}];
X322data = Transpose[{cycle, indata[[All, 48]][[2 ;;]]}];
X323data = Transpose[{cycle, indata[[All, 49]][[2 ;;]]}];
X324data = Transpose[{cycle, indata[[All, 50]][[2 ;;]]}];
X331data = Transpose[{cycle, indata[[All, 51]][[2 ;;]]}];
X332data = Transpose[{cycle, indata[[All, 52]][[2 ;;]]}];
X333data = Transpose[{cycle, indata[[All, 53]][[2 ;;]]}];
X334data = Transpose[{cycle, indata[[All, 54]][[2 ;;]]}];
X341data = Transpose[{cycle, indata[[All, 55]][[2 ;;]]}];
X342data = Transpose[{cycle, indata[[All, 56]][[2 ;;]]}];
X343data = Transpose[{cycle, indata[[All, 57]][[2 ;;]]}];
X344data = Transpose[{cycle, indata[[All, 58]][[2 ;;]]}];
X351data = Transpose[{cycle, indata[[All, 59]][[2 ;;]]}];
X352data = Transpose[{cycle, indata[[All, 60]][[2 ;;]]}];
X353data = Transpose[{cycle, indata[[All, 61]][[2 ;;]]}];
X354data = Transpose[{cycle, indata[[All, 62]][[2 ;;]]}];
X411data = Transpose[{cycle, indata[[All, 63]][[2 ;;]]}];
X412data = Transpose[{cycle, indata[[All, 64]][[2 ;;]]}];
X413data = Transpose[{cycle, indata[[All, 65]][[2 ;;]]}];
X414data = Transpose[{cycle, indata[[All, 66]][[2 ;;]]}];
X421data = Transpose[{cycle, indata[[All, 67]][[2 ;;]]}];
X422data = Transpose[{cycle, indata[[All, 68]][[2 ;;]]}];
X423data = Transpose[{cycle, indata[[All, 69]][[2 ;;]]}];
X424data = Transpose[{cycle, indata[[All, 70]][[2 ;;]]}];
X431data = Transpose[{cycle, indata[[All, 71]][[2 ;;]]}];
X432data = Transpose[{cycle, indata[[All, 72]][[2 ;;]]}];
X433data = Transpose[{cycle, indata[[All, 73]][[2 ;;]]}];
X434data = Transpose[{cycle, indata[[All, 74]][[2 ;;]]}];
X441data = Transpose[{cycle, indata[[All, 75]][[2 ;;]]}];
X442data = Transpose[{cycle, indata[[All, 76]][[2 ;;]]}];
X443data = Transpose[{cycle, indata[[All, 77]][[2 ;;]]}];
X444data = Transpose[{cycle, indata[[All, 78]][[2 ;;]]}];
X451data = Transpose[{cycle, indata[[All, 79]][[2 ;;]]}];
X452data = Transpose[{cycle, indata[[All, 80]][[2 ;;]]}];
X453data = Transpose[{cycle, indata[[All, 81]][[2 ;;]]}];
X454data = Transpose[{cycle, indata[[All, 82]][[2 ;;]]}];
X511data = Transpose[{cycle, indata[[All, 83]][[2 ;;]]}];
X512data = Transpose[{cycle, indata[[All, 84]][[2 ;;]]}];
X513data = Transpose[{cycle, indata[[All, 85]][[2 ;;]]}];
X514data = Transpose[{cycle, indata[[All, 86]][[2 ;;]]}];
X521data = Transpose[{cycle, indata[[All, 87]][[2 ;;]]}];
X522data = Transpose[{cycle, indata[[All, 88]][[2 ;;]]}];
```

```
X523data = Transpose[{cycle, indata[[All, 89]][[2 ;;]]}];
X524data = Transpose[{cycle, indata[[All, 90]][[2 ;;]]}];
X531data = Transpose[{cycle, indata[[All, 91]][[2 ;;]]}];
X532data = Transpose[{cycle, indata[[All, 92]][[2 ;;]]}];
X533data = Transpose[{cycle, indata[[All, 93]][[2 ;;]]}];
X534data = Transpose[{cycle, indata[[All, 94]][[2 ;;]]}];
X541data = Transpose[{cycle, indata[[All, 95]][[2 ;;]]}];
X542data = Transpose[{cycle, indata[[All, 96]][[2 ;;]]}];
X543data = Transpose[{cycle, indata[[All, 97]][[2 ;;]]}];
X544data = Transpose[{cycle, indata[[All, 98]][[2 ;;]]}];
X551data = Transpose[{cycle, indata[[All, 99]][[2 ;;]]}];
X552data = Transpose[{cycle, indata[[All, 100]][[2 ;;]]}];
X553data = Transpose[{cycle, indata[[All, 101]][[2 ;;]]}];
X554data = Transpose[{cycle, indata[[All, 102]][[2 ;;]]}];
X611data = Transpose[{cycle, indata[[All, 103]][[2 ;;]]}];
X612data = Transpose[{cycle, indata[[All, 104]][[2 ;;]]}];
X613data = Transpose[{cycle, indata[[All, 105]][[2 ;;]]}];
X614data = Transpose[{cycle, indata[[All, 106]][[2 ;;]]}];
X621data = Transpose[{cycle, indata[[All, 107]][[2 ;;]]}];
X622data = Transpose[{cycle, indata[[All, 108]][[2 ;;]]}];
X623data = Transpose[{cycle, indata[[All, 109]][[2 ;;]]}];
X624data = Transpose[{cycle, indata[[All, 110]][[2 ;;]]}];
X631data = Transpose[{cycle, indata[[All, 111]][[2 ;;]]}];
X632data = Transpose[{cycle, indata[[All, 112]][[2 ;;]]}];
X633data = Transpose[{cycle, indata[[All, 113]][[2 ;;]]}];
X634data = Transpose[{cycle, indata[[All, 114]][[2 ;;]]}];
X641data = Transpose[{cycle, indata[[All, 115]][[2 ;;]]}];
X642data = Transpose[{cycle, indata[[All, 116]][[2 ;;]]}];
X643data = Transpose[{cycle, indata[[All, 117]][[2 ;;]]}];
X644data = Transpose[{cycle, indata[[All, 118]][[2 ;;]]}];
X651data = Transpose[{cycle, indata[[All, 119]][[2 ;;]]}];
X652data = Transpose[{cycle, indata[[All, 120]][[2 ;;]]}];
X653data = Transpose[{cycle, indata[[All, 121]][[2 ;;]]}];
X654data = Transpose[{cycle, indata[[All, 122]][[2 ;;]]}];
```

```
CTD = {CTRoutine[X111data, .05], CTRoutine[X112data, .05], CTRoutine[X113data, .05],
  CTRoutine[X114data, .05], CTRoutine[X121data, .05], CTRoutine[X122data, .05],
  CTRoutine[X123data, .05], CTRoutine[X124data, .05], CTRoutine[X131data, .05],
  CTRoutine[X132data, .05], CTRoutine[X133data, .05], CTRoutine[X134data, .05],
  CTRoutine[X141data, .05], CTRoutine[X142data, .05], CTRoutine[X143data, .05],
  CTRoutine[X144data, .05], CTRoutine[X151data, .05], CTRoutine[X152data, .05],
  CTRoutine[X153data, .05], CTRoutine[X154data, .05], CTRoutine[X211data, .05],
  CTRoutine[X212data, .05], CTRoutine[X213data, .05], CTRoutine[X214data, .05],
  CTRoutine[X221data, .05], CTRoutine[X222data, .05], CTRoutine[X223data, .05],
  CTRoutine[X224data, .05], CTRoutine[X231data, .05], CTRoutine[X232data, .05],
  CTRoutine[X233data, .05], CTRoutine[X234data, .05], CTRoutine[X241data, .05],
  CTRoutine[X242data, .05], CTRoutine[X243data, .05], CTRoutine[X244data, .05],
  CTRoutine[X251data, .05], CTRoutine[X252data, .05], CTRoutine[X253data, .05],
  CTRoutine[X254data, .05], CTRoutine[X311data, .05], CTRoutine[X312data, .05],
  CTRoutine[X313data, .05], CTRoutine[X314data, .05], CTRoutine[X321data, .05],
  CTRoutine[X322data, .05], CTRoutine[X323data, .05], CTRoutine[X324data, .05],
  CTRoutine[X331data, .05], CTRoutine[X332data, .05], CTRoutine[X333data, .05],
  CTRoutine[X334data, .05], CTRoutine[X341data, .05], CTRoutine[X342data, .05],
  CTRoutine[X343data, .05], CTRoutine[X344data, .05], CTRoutine[X351data, .05],
  CTRoutine[X352data, .05], CTRoutine[X353data, .05], CTRoutine[X354data, .05],
  CTRoutine[X411data, .05], CTRoutine[X412data, .05], CTRoutine[X413data, .05],
  CTRoutine[X414data, .05], CTRoutine[X421data, .05], CTRoutine[X422data, .05],
  CTRoutine[X423data, .05], CTRoutine[X424data, .05], CTRoutine[X431data, .05],
  CTRoutine[X432data, .05], CTRoutine[X433data, .05], CTRoutine[X434data, .05],
  CTRoutine[X441data, .05], CTRoutine[X442data, .05], CTRoutine[X443data, .05],
  CTRoutine[X444data, .05], CTRoutine[X451data, .05], CTRoutine[X452data, .05],
  CTRoutine[X453data, .05], CTRoutine[X454data, .05], CTRoutine[X511data, .05],
  CTRoutine[X512data, .05], CTRoutine[X513data, .05], CTRoutine[X514data, .05],
  CTRoutine[X521data, .05], CTRoutine[X522data, .05], CTRoutine[X523data, .05],
  CTRoutine[X524data, .05], CTRoutine[X531data, .05], CTRoutine[X532data, .05],
  CTRoutine[X533data, .05], CTRoutine[X534data, .05], CTRoutine[X541data, .05],
  CTRoutine[X542data, .05], CTRoutine[X543data, .05], CTRoutine[X544data, .05],
  CTRoutine[X551data, .05], CTRoutine[X552data, .05], CTRoutine[X553data, .05],
  CTRoutine[X554data, .05], CTRoutine[X611data, .05], CTRoutine[X612data, .05],
  CTRoutine[X613data, .05], CTRoutine[X614data, .05], CTRoutine[X621data, .05],
  CTRoutine[X622data, .05], CTRoutine[X623data, .05], CTRoutine[X624data, .05],
  CTRoutine[X631data, .05], CTRoutine[X632data, .05], CTRoutine[X633data, .05],
  CTRoutine[X634data, .05], CTRoutine[X641data, .05], CTRoutine[X642data, .05],
  CTRoutine[X643data, .05], CTRoutine[X644data, .05], CTRoutine[X651data, .05],
  CTRoutine[X652data, .05], CTRoutine[X653data, .05], CTRoutine[X654data, .05]}
```

```
{11.4977, 11.1546, 11.4133, 11.3473, 11.9007, 11.9482, 12.0901, 12.1333, 12.2402, 11.6824,
 11.7326, 11.9691, 12.2297, 11.8847, 12.005, 11.8767, 11.7017, 11.5866, 11.625, 11.8448,
 15.0707, 15.1172, 15.5943, 15.4526, 15.3845, 15.4873, 15.6592, 15.4738, 15.3638, 15.4865,
 15.6481, 15.7258, 15.0946, 15.4465, 15.8111, 15.7967, 15.3191, 15.2795, 15.6389, 15.5798,
 19.4032, 19.2337, 19.4703, 19.4211, 19.405, 19.3105, 19.0715, 19.0269, 19.0344, 18.7806,
 18.938, 18.7808, 19.492, 19.1784, 19.4771, 19.4074, 19.3867, 19.0547, 19.1196, 18.7045,
 23.2209, 23.2715, 23.2803, 23.197, 23.0358, 22.7279, 22.9831, 23.0944, 22.6115, 22.6849,
 22.9272, 22.9437, 23.3241, 23.1724, 23.3925, 23.3106, 23.0334, 22.5781, 22.7272, 23.0534,
 25.87, 26.0116, 26.1281, 26.3432, 26.0776, 26.1511, 26.1076, 26.1934, 25.968, 25.97,
 25.9398, 26.0242, 26.2502, 26.5224, 26.1333, 26.4592, 26.2264, 26.1671, 26.0527, 26.2971,
 29.9721, 29.7302, 29.5745, 29.549, 29.8595, 29.7313, 29.7359, 29.7839, 29.6181, 29.563,
 29.4964, 29.6602, 30.0281, 30.1698, 30.417, 30.4324, 29.9231, 29.7143, 29.8448, 30.2714}
```

```
indata = Import["reps.csv"];
cycle = indata[[All, 1]][[2 ;;]];

F11data = Transpose[{cycle, indata[[All, 3]][[2 ;;]]}];
F12data = Transpose[{cycle, indata[[All, 4]][[2 ;;]]}];
F13data = Transpose[{cycle, indata[[All, 5]][[2 ;;]]}];
F14data = Transpose[{cycle, indata[[All, 6]][[2 ;;]]}];
F21data = Transpose[{cycle, indata[[All, 7]][[2 ;;]]}];
F22data = Transpose[{cycle, indata[[All, 8]][[2 ;;]]}];
F23data = Transpose[{cycle, indata[[All, 9]][[2 ;;]]}];
F24data = Transpose[{cycle, indata[[All, 10]][[2 ;;]]}];
F31data = Transpose[{cycle, indata[[All, 11]][[2 ;;]]}];
F32data = Transpose[{cycle, indata[[All, 12]][[2 ;;]]}];
F33data = Transpose[{cycle, indata[[All, 13]][[2 ;;]]}];
F34data = Transpose[{cycle, indata[[All, 14]][[2 ;;]]}];
F41data = Transpose[{cycle, indata[[All, 15]][[2 ;;]]}];
F42data = Transpose[{cycle, indata[[All, 16]][[2 ;;]]}];
F43data = Transpose[{cycle, indata[[All, 17]][[2 ;;]]}];
F44data = Transpose[{cycle, indata[[All, 18]][[2 ;;]]}];
F51data = Transpose[{cycle, indata[[All, 19]][[2 ;;]]}];
F52data = Transpose[{cycle, indata[[All, 20]][[2 ;;]]}];
F53data = Transpose[{cycle, indata[[All, 21]][[2 ;;]]}];
F54data = Transpose[{cycle, indata[[All, 22]][[2 ;;]]}];
F61data = Transpose[{cycle, indata[[All, 23]][[2 ;;]]}];
F62data = Transpose[{cycle, indata[[All, 24]][[2 ;;]]}];
F63data = Transpose[{cycle, indata[[All, 25]][[2 ;;]]}];
F64data = Transpose[{cycle, indata[[All, 26]][[2 ;;]]}];
F71data = Transpose[{cycle, indata[[All, 27]][[2 ;;]]}];
F72data = Transpose[{cycle, indata[[All, 28]][[2 ;;]]}];
F73data = Transpose[{cycle, indata[[All, 29]][[2 ;;]]}];
F74data = Transpose[{cycle, indata[[All, 30]][[2 ;;]]}];

CTD = {CTRoutine[F11data, 1], CTRoutine[F12data, 1], CTRoutine[F13data, 1],
  CTRoutine[F14data, 1], CTRoutine[F21data, 1], CTRoutine[F22data, 1], CTRoutine[F23data, 1],
  CTRoutine[F24data, 1], CTRoutine[F31data, 1], CTRoutine[F32data, 1], CTRoutine[F33data, 1],
  CTRoutine[F34data, 1], CTRoutine[F41data, 1], CTRoutine[F42data, 1], CTRoutine[F43data, 1],
  CTRoutine[F44data, 1], CTRoutine[F51data, 1], CTRoutine[F52data, 1], CTRoutine[F53data, 1],
  CTRoutine[F54data, 1], CTRoutine[F61data, 1], CTRoutine[F62data, 1],
  CTRoutine[F63data, 1], CTRoutine[F64data, 1], CTRoutine[F71data, 1],
  CTRoutine[F72data, 1], CTRoutine[F73data, 1], CTRoutine[F74data, 1]}

{14.0861, 13.8418, 13.9187, 14.3627, 17.557, 17.2929, 17.0396, 16.2389, 20.7992, 20.4868,
 19.9947, 19.8947, 24.0745, 23.6808, 23.9871, 23.4542, 27.1304, 26.9617, 26.9653,
 27.0546, 30.6235, 30.4318, 30.6999, 30.6195, 38.7517, 38.4553, 39.1615, 38.5463}
```
