# Supplementary material for: A Mechanistic Model of PCR for Accurate Quantification of Quantitative PCR Data
Source: PLoS One. 2010 Aug 30;5(8):e12355. doi: 10.1371/journal.pone.0012355 (PMC2930010; doi:10.1371/journal.pone.0012355)

```
(*****************************************************************************************)
(*  This Mathematica notebook contains trials of fitting qPCR data with an exponential model.      *)
(*  The 'd0' values resulting from these trials are used to develop the plots in figure 3 of the MAK2 paper.    *)
(*                                                                                         *)
(******                    Written by Gregory Boggy (November 2009)                   *****)
(*****************************************************************************************)
```

```
filepath = "Desktop/supplement/";
SetDirectory[filepath];
indata = Import["growth.csv"];
cycle = indata[[All, 1]];

D3data = Transpose[{cycle, indata[[All, 2]]}][[2 ;; 41]];
D4data = Transpose[{cycle, indata[[All, 3]]}][[2 ;; 41]];
D5data = Transpose[{cycle, indata[[All, 4]]}][[2 ;; 41]];
D6data = Transpose[{cycle, indata[[All, 5]]}][[2 ;; 41]];
D7data = Transpose[{cycle, indata[[All, 6]]}][[2 ;; 41]];
D8data = Transpose[{cycle, indata[[All, 7]]}][[2 ;; 41]];
E3data = Transpose[{cycle, indata[[All, 8]]}][[2 ;; 41]];
E4data = Transpose[{cycle, indata[[All, 9]]}][[2 ;; 41]];
E5data = Transpose[{cycle, indata[[All, 10]]}][[2 ;; 41]];
E6data = Transpose[{cycle, indata[[All, 11]]}][[2 ;; 41]];
E7data = Transpose[{cycle, indata[[All, 12]]}][[2 ;; 41]];
E8data = Transpose[{cycle, indata[[All, 13]]}][[2 ;; 41]];
```

```
D3exp = NonlinearModelFit[D3data[[1 ;; 28]], b + d0 * a^x, {b, d0, a}, x, MaxIterations → 1000]
```

FittedModel[$0.193431 + 2.77761\times10^{-7}$ ≪19≫^{x}]

```
D3exp["BestFit"]
```

$0.193431 + 2.77761\times10^{-7}\, 1.61913^{x}$

```
Show[ListPlot[D3data[[1 ;; 28]], PlotRange → All], Plot[D3exp[x], {x, 1, 31}]]
```

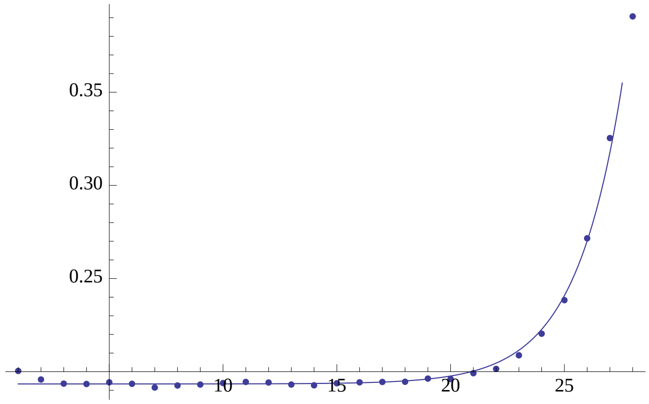


```
D4exp = NonlinearModelFit[D4data[[1 ;; 24]], b + d0 * a^x, {b, d0, a}, x, MaxIterations → 10 000]
```

FittedModel[$0.193799 + 2.99676\times10^{-7}$ ≪19≫^{x}]

```
D4exp["BestFit"]
```

0.193799 + 2.99676 × 10^-7 1.74385^x

```
Show[ListPlot[D4data[[1 ;; 24]], PlotRange → All], Plot[D4exp[x], {x, 1, 28}]]
```

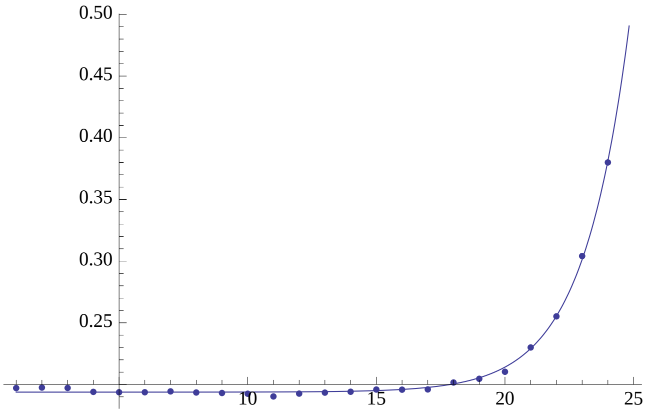


```
D5exp = NonlinearModelFit[D5data[[1 ;; 20]], b + d0 * a^x, {b, d0, a}, x, MaxIterations → 10 000]
```

FittedModel[0.195746 + 1.08399 × 10^-6 ≪18≫^x]

```
D5exp["BestFit"]
```

0.195746 + 1.08399 × 10^-6 1.82639^x

```
Show[ListPlot[D5data[[1 ;; 20]], PlotRange → All], Plot[D5exp[x], {x, 1, 23}]]
```

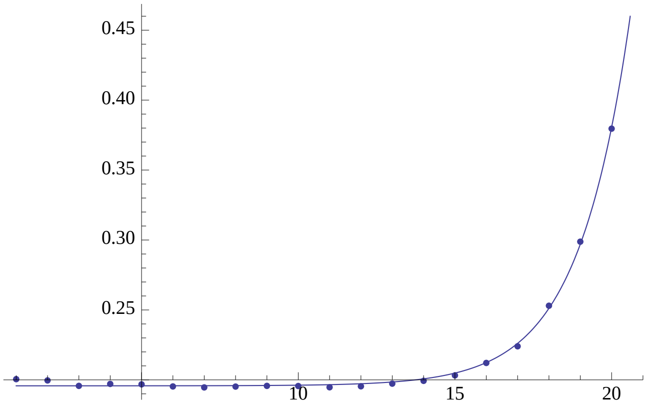


```
D6exp = NonlinearModelFit[D6data[[1 ;; 17]], b + d0 * a^x, {b, d0, a}, x, MaxIterations → 10 000]
```

FittedModel[0.191898 + 4.55187 × 10^-6 ≪19≫^x]

```
D6exp["BestFit"]
```

0.191898 + 4.55187 × 10^-6 1.86618^x

```
Show[ListPlot[D6data[[1 ;; 17]], PlotRange → All], Plot[D6exp[x], {x, 1, 19}]]
```

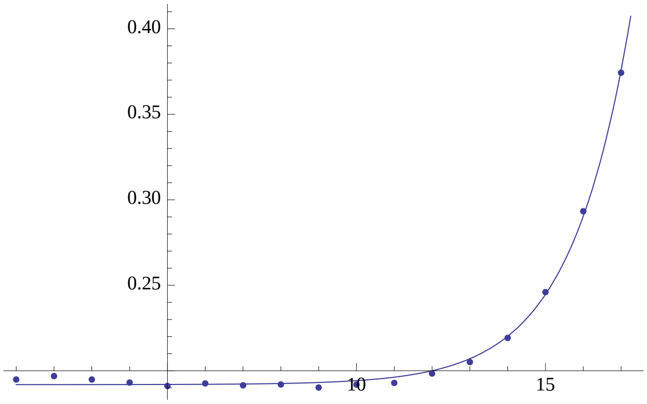


```
D7exp = NonlinearModelFit[D7data[[1 ;; 13]], b + d0 * a^x, {b, d0, a}, x, MaxIterations → 1000]
```

FittedModel[0.196345 + 0.0000262309 ≪19≫^{x}]

```
D7exp["BestFit"]
```

$0.196345 + 0.0000262309\ 1.95542^{x}$

```
Show[ListPlot[D7data[[1 ;; 13]], PlotRange → All], Plot[D7exp[x], {x, 1, 15}]]
```

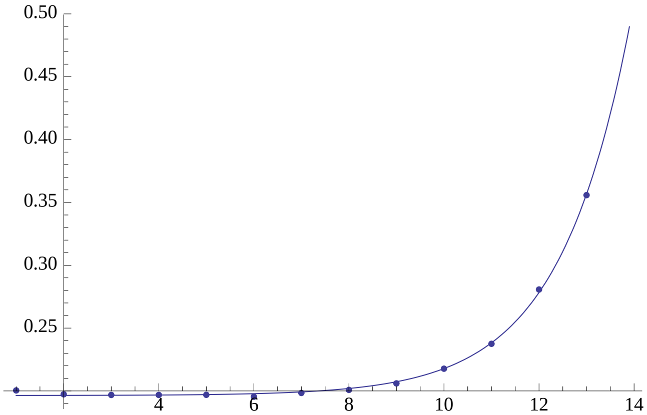


```
D8exp = NonlinearModelFit[D8data[[1 ;; 10]], b + d0 * a^x, {b, d0, a}, x, MaxIterations → 10 000]
```

FittedModel[0.194705 + 0.000267799 ≪19≫^{x}]

```
D8exp["BestFit"]
```

$0.194705 + 0.000267799\ 1.94344^{x}$

```
Show[ListPlot[D8data[[1 ;; 10]], PlotRange → All], Plot[D8exp[x], {x, 1, 10}]]
```

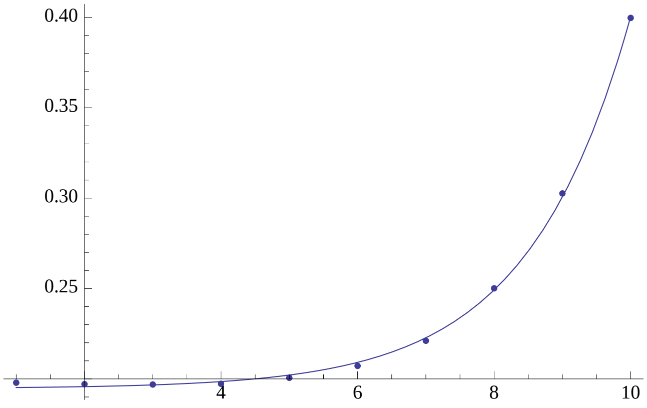


```
E3exp = NonlinearModelFit[E3data[[1 ;; 28]], b + d0 * a^x, {b, d0, a}, x, MaxIterations → 10 000]
```

FittedModel[$0.188313 + 4.72056 \times 10^{-8} \ll 18 \gg^{x}$]

```
E3exp["BestFit"]
```

$0.188313 + 4.72056 \times 10^{-8}\, 1.72559^{x}$

```
Show[ListPlot[E3data[[1 ;; 28]], PlotRange → All], Plot[E3exp[x], {x, 1, 31}]]
```

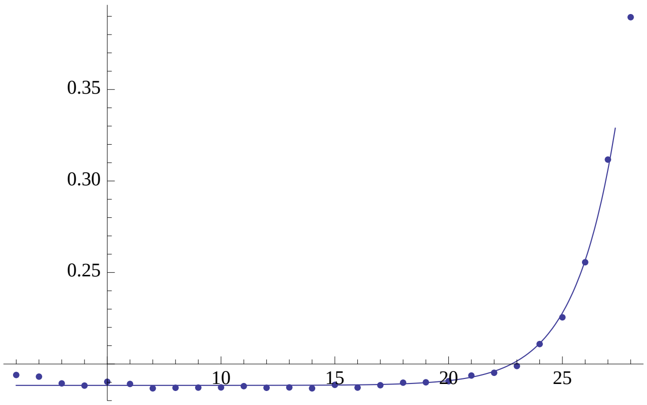


```
E4exp = NonlinearModelFit[E4data[[1 ;; 24]], b + d0 * a^x, {b, d0, a}, x, MaxIterations → 10 000]
```

FittedModel[$0.196468 + 1.87325 \times 10^{-7} \ll 18 \gg^{x}$]

```
E4exp["BestFit"]
```

$0.196468 + 1.87325 \times 10^{-7}\, 1.79141^{x}$

```
Show[ListPlot[E4data[[1 ;; 24]], PlotRange → All], Plot[E4exp[x], {x, 1, 28}]]
```

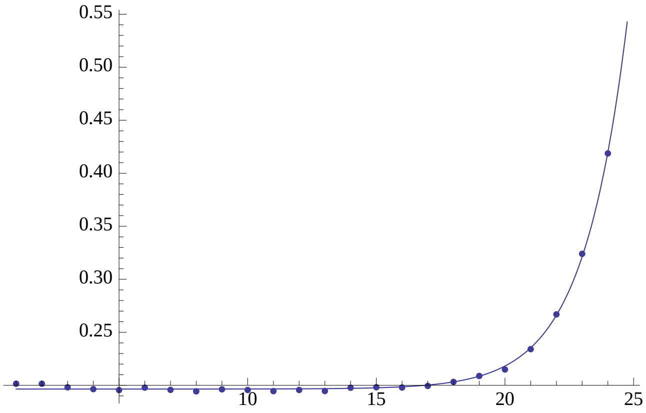


```
E5exp = NonlinearModelFit[E5data[[1 ;; 20]], b + d0 * a^x, {b, d0, a}, x, MaxIterations → 10 000]
```

FittedModel[$0.195601 + 1.61624\times10^{-6} \ll 19 \gg^{x}$]

```
E5exp["BestFit"]
```

$0.195601 + 1.61624 \times 10^{-6}\, 1.79682^{x}$

```
Show[ListPlot[E5data[[1 ;; 20]], PlotRange → All], Plot[E5exp[x], {x, 1, 23}]]
```

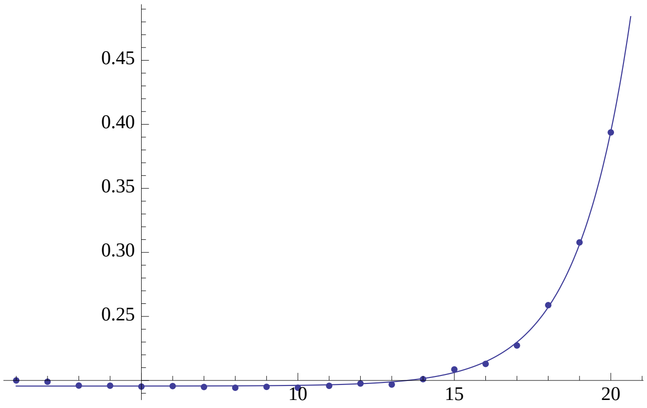


```
E6exp = NonlinearModelFit[E6data[[1 ;; 17]], b + d0 * a^x, {b, d0, a}, x, MaxIterations → 10 000]
```

FittedModel[$0.191688 + 5.26034\times10^{-6} \ll 18 \gg^{x}$]

```
E6exp["BestFit"]
```

$0.191688 + 5.26034 \times 10^{-6}\, 1.8553^{x}$

```
Show[ListPlot[E6data[[1 ;; 17]], PlotRange → All], Plot[E6exp[x], {x, 1, 19}]]
```

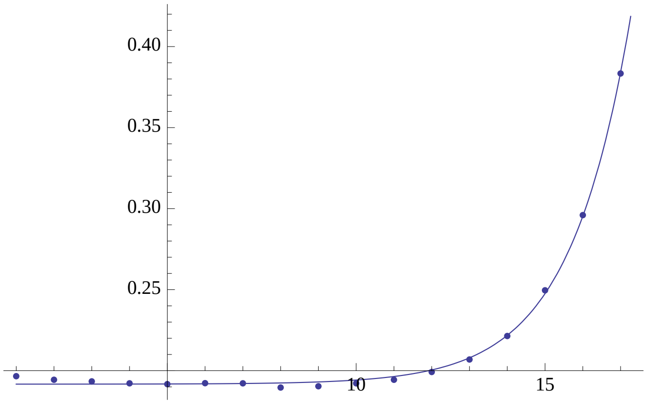


```
E7exp = NonlinearModelFit[E7data[[1 ;; 13]], b + d0 * a^x, {b, d0, a}, x, MaxIterations → 10 000]
```

FittedModel[0.190447 + 0.0000264797 ≪19≫^x]

```
E7exp["BestFit"]
```

$0.190447 + 0.0000264797\ 1.94885^{x}$

```
Show[ListPlot[E7data[[1 ;; 13]], PlotRange → All], Plot[E7exp[x], {x, 1, 15}]]
```

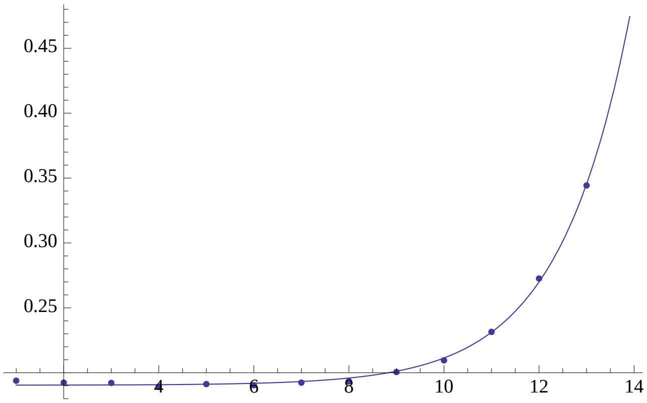


```
E8exp = NonlinearModelFit[E8data[[1 ;; 10]], b + d0 * a^x, {b, d0, a}, x, MaxIterations → 10 000]
```

FittedModel[0.200804 + 0.000345749 ≪18≫^x]

```
E8exp["BestFit"]
```

$0.200804 + 0.000345749\ 1.91906^{x}$

```
Show[ListPlot[E8data[[1 ;; 10]], PlotRange → All], Plot[E8exp[x], {x, 1, 10}]]
```

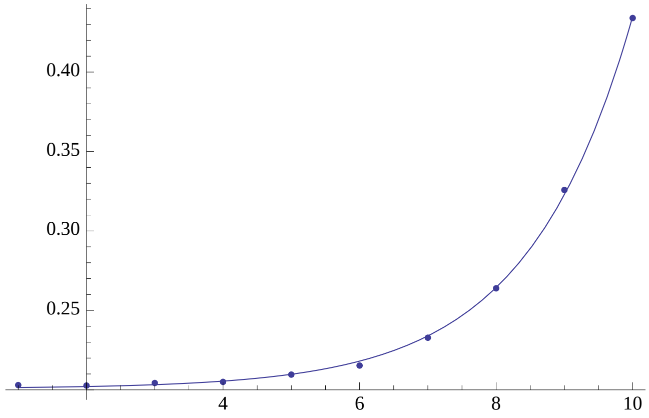


```
indata = Import["rutledge.csv"];
cycle = indata[[All, 1]][[2 ;;]];

X111data = Transpose[{cycle, indata[[All, 3]][[2 ;;]]}];
X112data = Transpose[{cycle, indata[[All, 4]][[2 ;;]]}];
X113data = Transpose[{cycle, indata[[All, 5]][[2 ;;]]}];
X114data = Transpose[{cycle, indata[[All, 6]][[2 ;;]]}];
X121data = Transpose[{cycle, indata[[All, 7]][[2 ;;]]}];
X122data = Transpose[{cycle, indata[[All, 8]][[2 ;;]]}];
X123data = Transpose[{cycle, indata[[All, 9]][[2 ;;]]}];
X124data = Transpose[{cycle, indata[[All, 10]][[2 ;;]]}];
X131data = Transpose[{cycle, indata[[All, 11]][[2 ;;]]}];
X132data = Transpose[{cycle, indata[[All, 12]][[2 ;;]]}];
X133data = Transpose[{cycle, indata[[All, 13]][[2 ;;]]}];
X134data = Transpose[{cycle, indata[[All, 14]][[2 ;;]]}];
X141data = Transpose[{cycle, indata[[All, 15]][[2 ;;]]}];
X142data = Transpose[{cycle, indata[[All, 16]][[2 ;;]]}];
X143data = Transpose[{cycle, indata[[All, 17]][[2 ;;]]}];
X144data = Transpose[{cycle, indata[[All, 18]][[2 ;;]]}];
X151data = Transpose[{cycle, indata[[All, 19]][[2 ;;]]}];
X152data = Transpose[{cycle, indata[[All, 20]][[2 ;;]]}];
X153data = Transpose[{cycle, indata[[All, 21]][[2 ;;]]}];
X154data = Transpose[{cycle, indata[[All, 22]][[2 ;;]]}];
X211data = Transpose[{cycle, indata[[All, 23]][[2 ;;]]}];
X212data = Transpose[{cycle, indata[[All, 24]][[2 ;;]]}];
```

```
X213data = Transpose[{cycle, indata[[All, 25]][[2 ;;]]}];
X214data = Transpose[{cycle, indata[[All, 26]][[2 ;;]]}];
X221data = Transpose[{cycle, indata[[All, 27]][[2 ;;]]}];
X222data = Transpose[{cycle, indata[[All, 28]][[2 ;;]]}];
X223data = Transpose[{cycle, indata[[All, 29]][[2 ;;]]}];
X224data = Transpose[{cycle, indata[[All, 30]][[2 ;;]]}];
X231data = Transpose[{cycle, indata[[All, 31]][[2 ;;]]}];
X232data = Transpose[{cycle, indata[[All, 32]][[2 ;;]]}];
X233data = Transpose[{cycle, indata[[All, 33]][[2 ;;]]}];
X234data = Transpose[{cycle, indata[[All, 34]][[2 ;;]]}];
X241data = Transpose[{cycle, indata[[All, 35]][[2 ;;]]}];
X242data = Transpose[{cycle, indata[[All, 36]][[2 ;;]]}];
X243data = Transpose[{cycle, indata[[All, 37]][[2 ;;]]}];
X244data = Transpose[{cycle, indata[[All, 38]][[2 ;;]]}];
X251data = Transpose[{cycle, indata[[All, 39]][[2 ;;]]}];
X252data = Transpose[{cycle, indata[[All, 40]][[2 ;;]]}];
X253data = Transpose[{cycle, indata[[All, 41]][[2 ;;]]}];
X254data = Transpose[{cycle, indata[[All, 42]][[2 ;;]]}];
X311data = Transpose[{cycle, indata[[All, 43]][[2 ;;]]}];
X312data = Transpose[{cycle, indata[[All, 44]][[2 ;;]]}];
X313data = Transpose[{cycle, indata[[All, 45]][[2 ;;]]}];
X314data = Transpose[{cycle, indata[[All, 46]][[2 ;;]]}];
X321data = Transpose[{cycle, indata[[All, 47]][[2 ;;]]}];
X322data = Transpose[{cycle, indata[[All, 48]][[2 ;;]]}];
X323data = Transpose[{cycle, indata[[All, 49]][[2 ;;]]}];
X324data = Transpose[{cycle, indata[[All, 50]][[2 ;;]]}];
X331data = Transpose[{cycle, indata[[All, 51]][[2 ;;]]}];
X332data = Transpose[{cycle, indata[[All, 52]][[2 ;;]]}];
X333data = Transpose[{cycle, indata[[All, 53]][[2 ;;]]}];
X334data = Transpose[{cycle, indata[[All, 54]][[2 ;;]]}];
X341data = Transpose[{cycle, indata[[All, 55]][[2 ;;]]}];
X342data = Transpose[{cycle, indata[[All, 56]][[2 ;;]]}];
X343data = Transpose[{cycle, indata[[All, 57]][[2 ;;]]}];
X344data = Transpose[{cycle, indata[[All, 58]][[2 ;;]]}];
X351data = Transpose[{cycle, indata[[All, 59]][[2 ;;]]}];
X352data = Transpose[{cycle, indata[[All, 60]][[2 ;;]]}];
X353data = Transpose[{cycle, indata[[All, 61]][[2 ;;]]}];
X354data = Transpose[{cycle, indata[[All, 62]][[2 ;;]]}];
X411data = Transpose[{cycle, indata[[All, 63]][[2 ;;]]}];
X412data = Transpose[{cycle, indata[[All, 64]][[2 ;;]]}];
X413data = Transpose[{cycle, indata[[All, 65]][[2 ;;]]}];
X414data = Transpose[{cycle, indata[[All, 66]][[2 ;;]]}];
X421data = Transpose[{cycle, indata[[All, 67]][[2 ;;]]}];
X422data = Transpose[{cycle, indata[[All, 68]][[2 ;;]]}];
X423data = Transpose[{cycle, indata[[All, 69]][[2 ;;]]}];
X424data = Transpose[{cycle, indata[[All, 70]][[2 ;;]]}];
X431data = Transpose[{cycle, indata[[All, 71]][[2 ;;]]}];
X432data = Transpose[{cycle, indata[[All, 72]][[2 ;;]]}];
X433data = Transpose[{cycle, indata[[All, 73]][[2 ;;]]}];
X434data = Transpose[{cycle, indata[[All, 74]][[2 ;;]]}];
X441data = Transpose[{cycle, indata[[All, 75]][[2 ;;]]}];
```

```
X442data = Transpose[{cycle, indata[[All, 76]][[2 ;;]]}];
X443data = Transpose[{cycle, indata[[All, 77]][[2 ;;]]}];
X444data = Transpose[{cycle, indata[[All, 78]][[2 ;;]]}];
X451data = Transpose[{cycle, indata[[All, 79]][[2 ;;]]}];
X452data = Transpose[{cycle, indata[[All, 80]][[2 ;;]]}];
X453data = Transpose[{cycle, indata[[All, 81]][[2 ;;]]}];
X454data = Transpose[{cycle, indata[[All, 82]][[2 ;;]]}];
X511data = Transpose[{cycle, indata[[All, 83]][[2 ;;]]}];
X512data = Transpose[{cycle, indata[[All, 84]][[2 ;;]]}];
X513data = Transpose[{cycle, indata[[All, 85]][[2 ;;]]}];
X514data = Transpose[{cycle, indata[[All, 86]][[2 ;;]]}];
X521data = Transpose[{cycle, indata[[All, 87]][[2 ;;]]}];
X522data = Transpose[{cycle, indata[[All, 88]][[2 ;;]]}];
X523data = Transpose[{cycle, indata[[All, 89]][[2 ;;]]}];
X524data = Transpose[{cycle, indata[[All, 90]][[2 ;;]]}];
X531data = Transpose[{cycle, indata[[All, 91]][[2 ;;]]}];
X532data = Transpose[{cycle, indata[[All, 92]][[2 ;;]]}];
X533data = Transpose[{cycle, indata[[All, 93]][[2 ;;]]}];
X534data = Transpose[{cycle, indata[[All, 94]][[2 ;;]]}];
X541data = Transpose[{cycle, indata[[All, 95]][[2 ;;]]}];
X542data = Transpose[{cycle, indata[[All, 96]][[2 ;;]]}];
X543data = Transpose[{cycle, indata[[All, 97]][[2 ;;]]}];
X544data = Transpose[{cycle, indata[[All, 98]][[2 ;;]]}];
X551data = Transpose[{cycle, indata[[All, 99]][[2 ;;]]}];
X552data = Transpose[{cycle, indata[[All, 100]][[2 ;;]]}];
X553data = Transpose[{cycle, indata[[All, 101]][[2 ;;]]}];
X554data = Transpose[{cycle, indata[[All, 102]][[2 ;;]]}];
X611data = Transpose[{cycle, indata[[All, 103]][[2 ;;]]}];
X612data = Transpose[{cycle, indata[[All, 104]][[2 ;;]]}];
X613data = Transpose[{cycle, indata[[All, 105]][[2 ;;]]}];
X614data = Transpose[{cycle, indata[[All, 106]][[2 ;;]]}];
X621data = Transpose[{cycle, indata[[All, 107]][[2 ;;]]}];
X622data = Transpose[{cycle, indata[[All, 108]][[2 ;;]]}];
X623data = Transpose[{cycle, indata[[All, 109]][[2 ;;]]}];
X624data = Transpose[{cycle, indata[[All, 110]][[2 ;;]]}];
X631data = Transpose[{cycle, indata[[All, 111]][[2 ;;]]}];
X632data = Transpose[{cycle, indata[[All, 112]][[2 ;;]]}];
X633data = Transpose[{cycle, indata[[All, 113]][[2 ;;]]}];
X634data = Transpose[{cycle, indata[[All, 114]][[2 ;;]]}];
X641data = Transpose[{cycle, indata[[All, 115]][[2 ;;]]}];
X642data = Transpose[{cycle, indata[[All, 116]][[2 ;;]]}];
X643data = Transpose[{cycle, indata[[All, 117]][[2 ;;]]}];
X644data = Transpose[{cycle, indata[[All, 118]][[2 ;;]]}];
X651data = Transpose[{cycle, indata[[All, 119]][[2 ;;]]}];
X652data = Transpose[{cycle, indata[[All, 120]][[2 ;;]]}];
X653data = Transpose[{cycle, indata[[All, 121]][[2 ;;]]}];
X654data = Transpose[{cycle, indata[[All, 122]][[2 ;;]]}];
```

```
X111exp =
 NonlinearModelFit[X111data[[1 ;; 14]], b + d0 * a^x, {b, d0, a}, x, MaxIterations → 1000]
```

FittedModel[$-0.00219 + 0.000191693 \ll 19 \gg^{x}$]

```
X111exp["BestFit"]
```

$-0.00219 + 0.000191693\ 1.62508^{x}$

```
Show[ListPlot[X111data[[1 ;; 14]], PlotRange → All], Plot[X111exp[x], {x, 1, 15}]]
```

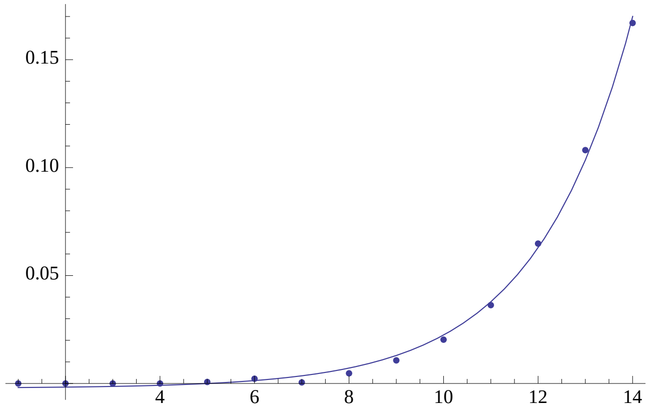


```
X112exp =
 NonlinearModelFit[X112data[[1 ;; 14]], b + d0 * a^x, {b, d0, a}, x, MaxIterations → 1000]
```

FittedModel[$-0.00238513 + 0.000226237 \ll 19 \gg^{x}$]

```
X112exp["BestFit"]
```

$-0.00238513 + 0.000226237\ 1.62638^{x}$

```
Show[ListPlot[X112data[[1 ;; 14]], PlotRange → All], Plot[X112exp[x], {x, 1, 15}]]
```

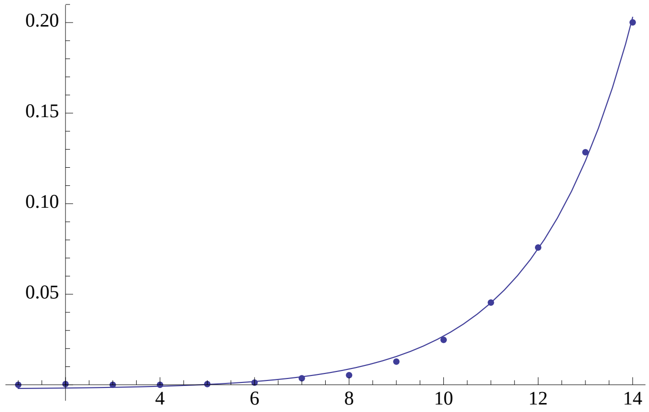


```
X113exp =
 NonlinearModelFit[X113data[[1 ;; 14]], b + d0 * a^x, {b, d0, a}, x, MaxIterations → 1000]
```

FittedModel[$-0.00191785 + 0.000166168 \ll 18 \gg^{x}$]

```
X113exp["BestFit"]
```

$-0.00191785 + 0.000166168\ 1.6501^{x}$

```
Show[ListPlot[X113data[[1 ;; 14]], PlotRange → All], Plot[X113exp[x], {x, 1, 15}]]
```

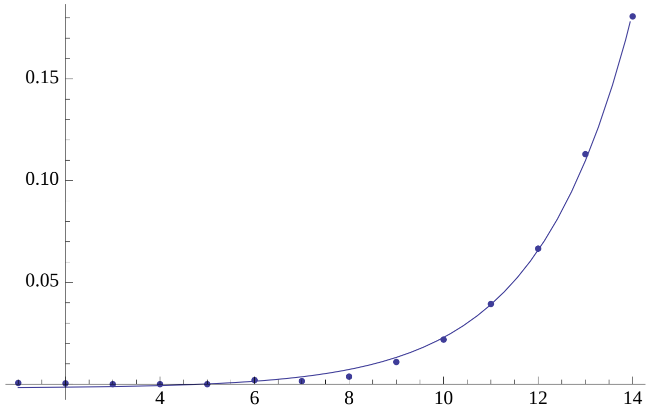


```
X114exp =
 NonlinearModelFit[X114data[[1 ;; 14]], b + d0 * a^x, {b, d0, a}, x, MaxIterations → 1000]
```

FittedModel[$-0.0020944 + 0.000187687 \ll 19 \gg^{x}$]

```
X114exp["BestFit"]
```

$-0.0020944 + 0.000187687\ 1.63819^{x}$

```
Show[ListPlot[X114data[[1 ;; 14]], PlotRange → All], Plot[X114exp[x], {x, 1, 15}]]
```

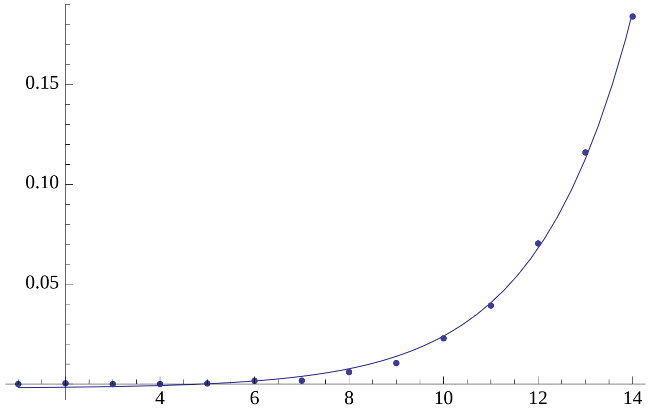


```
X121exp =
 NonlinearModelFit[X121data[[1 ;; 14]], b + d0 * a^x, {b, d0, a}, x, MaxIterations → 1000]
```

FittedModel[$-0.00161267 + 0.000160324 \ll 19 \gg^{x}$]

```
X121exp["BestFit"]
```

$-0.00161267 + 0.000160324\ 1.62227^{x}$

```
Show[ListPlot[X121data[[1 ;; 14]], PlotRange → All], Plot[X121exp[x], {x, 1, 15}]]
```

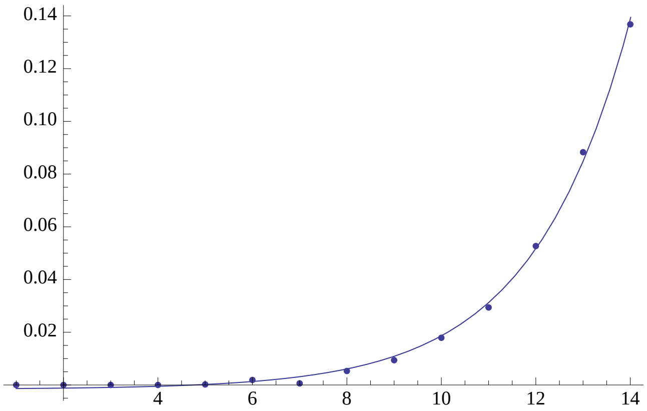


```
X122exp =
 NonlinearModelFit[X122data[[1 ;; 14]], b + d0 * a^x, {b, d0, a}, x, MaxIterations → 1000]
```

FittedModel[$-0.00145194 + 0.000118936 \ll 19 \gg^{x}$]

```
X122exp["BestFit"]
```

$-0.00145194 + 0.000118936\ 1.66126^{x}$

```
Show[ListPlot[X122data[[1 ;; 14]], PlotRange → All], Plot[X122exp[x], {x, 1, 15}]]
```

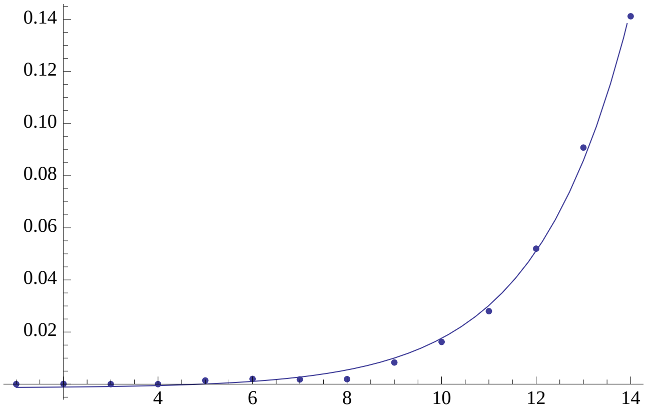


```
X123exp =
 NonlinearModelFit[X123data[[2 ;; 14]], b + d0 * a^x, {b, d0, a}, x, MaxIterations → 1000]
```

FittedModel[$-0.00159448 + 0.000156214 \ll 18 \gg^{x}$]

```
X123exp["BestFit"]
```

$-0.00159448 + 0.000156214\ 1.6133^{x}$

```
Show[ListPlot[X123data[[1 ;; 14]], PlotRange → All], Plot[X123exp[x], {x, 1, 15}]]
```

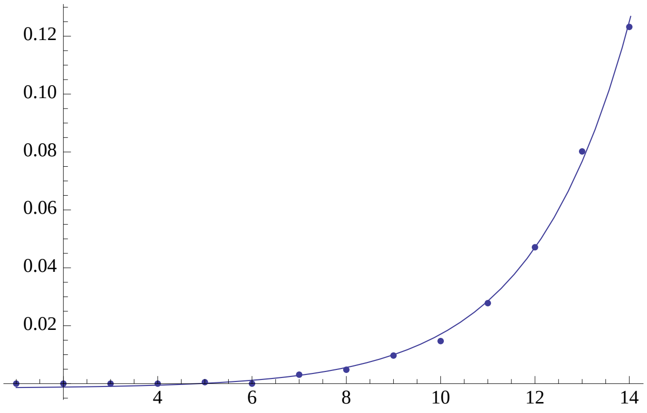


```
X124exp =
 NonlinearModelFit[X124data[[2 ;; 14]], b + d0 * a^x, {b, d0, a}, x, MaxIterations → 1000]
```

FittedModel[$-0.00133576 + 0.000125712 \ll 19 \gg^{x}$]

```
X124exp["BestFit"]
```

$-0.00133576 + 0.000125712\ 1.63696^{x}$

```
Show[ListPlot[X124data[[1 ;; 14]], PlotRange → All], Plot[X124exp[x], {x, 1, 15}]]
```

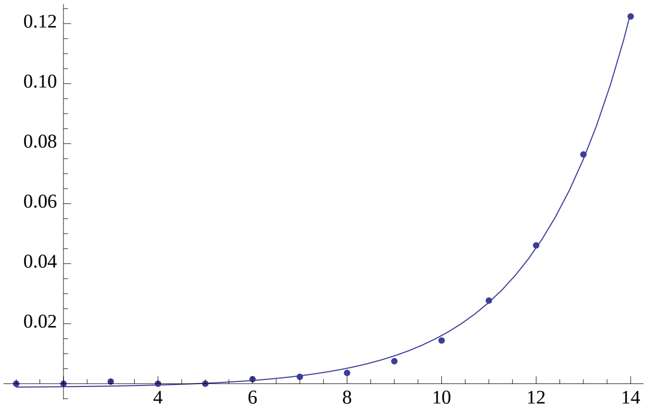


```
X131exp =
 NonlinearModelFit[X131data[[2 ;; 14]], b + d0 * a^x, {b, d0, a}, x, MaxIterations → 1000]
```

FittedModel[$-0.00100539 + 0.000137609 \ll 18 \gg^{x}$]

```
X131exp["BestFit"]
```

$-0.00100539 + 0.000137609\ 1.61801^{x}$

```
Show[ListPlot[X131data[[1 ;; 14]], PlotRange → All], Plot[X131exp[x], {x, 1, 15}]]
```

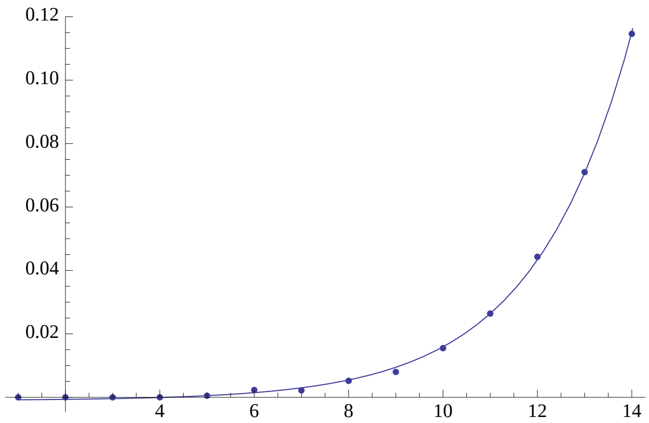


```
X132exp =
 NonlinearModelFit[X132data[[2 ;; 14]], b + d0 * a^x, {b, d0, a}, x, MaxIterations → 1000]
```

FittedModel[$-0.00155154 + 0.000164489 \ll 18 \gg^{x}$]

```
X132exp["BestFit"]
```

$-0.00155154 + 0.000164489\ 1.63397^{x}$

```
Show[ListPlot[X132data[[1 ;; 14]], PlotRange → All], Plot[X132exp[x], {x, 1, 15}]]
```

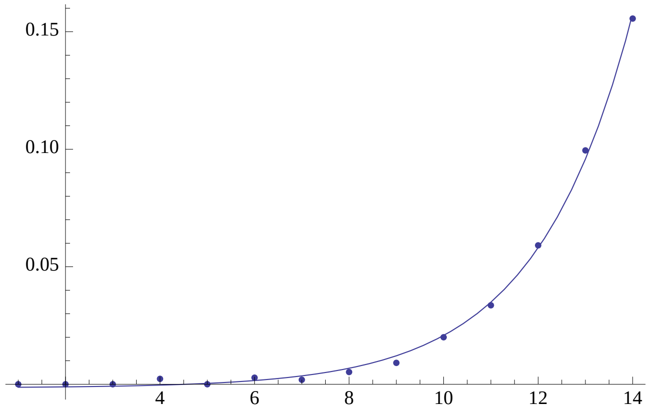


```
X133exp =
 NonlinearModelFit[X133data[[2 ;; 14]], b + d0 * a^x, {b, d0, a}, x, MaxIterations → 1000]
```

FittedModel[$-0.00176055 + 0.000176786 \ll 19 \gg^{x}$]

```
X133exp["BestFit"]
```

$-0.00176055 + 0.000176786\ 1.61879^{x}$

```
Show[ListPlot[X133data[[1 ;; 14]], PlotRange → All], Plot[X133exp[x], {x, 1, 15}]]
```

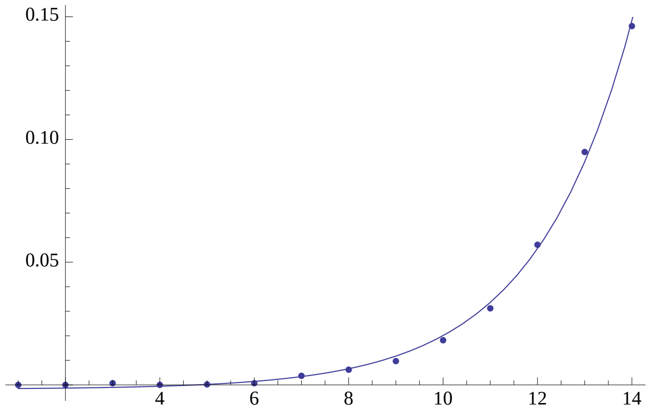


```
X134exp =
 NonlinearModelFit[X134data[[2 ;; 14]], b + d0 * a^x, {b, d0, a}, x, MaxIterations → 1000]
```

FittedModel[$-0.00103387 + 0.000145468 \ll 19 \gg^{x}$]

```
X134exp["BestFit"]
```

$-0.00103387 + 0.000145468\ 1.62944^{x}$

```
Show[ListPlot[X134data[[1 ;; 14]], PlotRange → All], Plot[X134exp[x], {x, 1, 15}]]
```

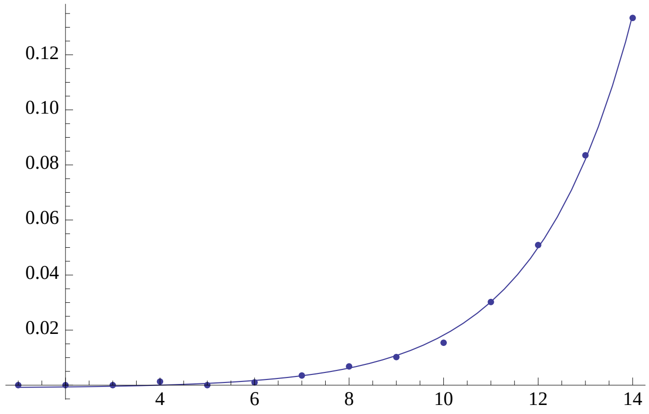


```
X141exp =
 NonlinearModelFit[X141data[[2 ;; 14]], b + d0 * a^x, {b, d0, a}, x, MaxIterations → 1000]
```

FittedModel[$-0.00144893 + 0.000119027 \ll 19 \gg^{x}$]

```
X141exp["BestFit"]
```

$-0.00144893 + 0.000119027\ 1.63747^{x}$

```
Show[ListPlot[X141data[[1 ;; 14]], PlotRange → All], Plot[X141exp[x], {x, 1, 15}]]
```

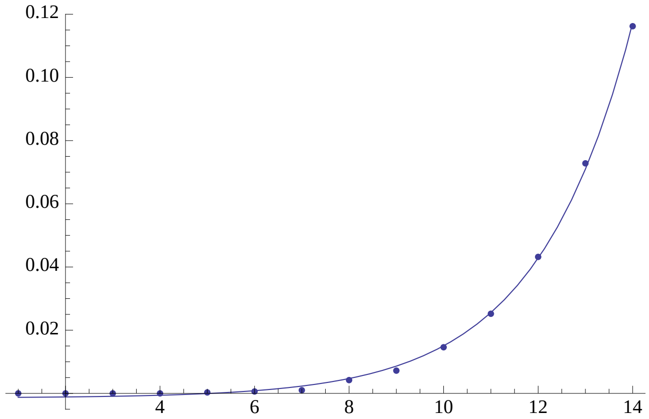


```
X142exp =
 NonlinearModelFit[X142data[[2 ;; 14]], b + d0 * a^x, {b, d0, a}, x, MaxIterations → 1000]
```

```
FittedModel[ −0.00170321 + 0.000156386 ≪19≫^x ]
```

```
X142exp["BestFit"]
```

$-0.00170321 + 0.000156386\ 1.62582^{x}$

```
Show[ListPlot[X142data[[1 ;; 14]], PlotRange → All], Plot[X142exp[x], {x, 1, 15}]]
```

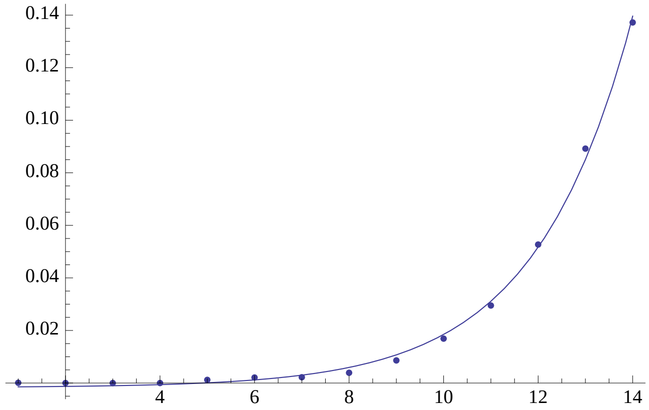


```
X143exp =
 NonlinearModelFit[X143data[[2 ;; 14]], b + d0 * a^x, {b, d0, a}, x, MaxIterations → 1000]
```

```
FittedModel[ −0.00201219 + 0.000157495 ≪19≫^x ]
```

```
X143exp["BestFit"]
```

$-0.00201219 + 0.000157495\ 1.61995^{x}$

```
Show[ListPlot[X143data[[1 ;; 14]], PlotRange → All], Plot[X143exp[x], {x, 1, 15}]]
```

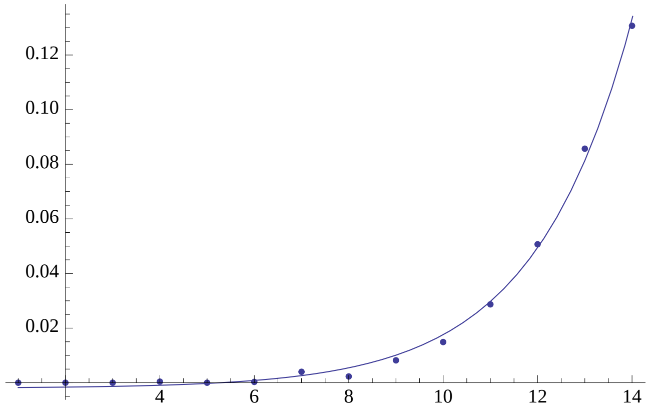


```
X144exp =
 NonlinearModelFit[X144data[[2 ;; 14]], b + d0 * a^x, {b, d0, a}, x, MaxIterations → 1000]
```

FittedModel[$-0.00162929 + 0.000189251 \ll 19 \gg^{x}$]

```
X144exp["BestFit"]
```

$-0.00162929 + 0.000189251\ 1.60171^{x}$

```
Show[ListPlot[X144data[[1 ;; 14]], PlotRange → All], Plot[X144exp[x], {x, 1, 15}]]
```

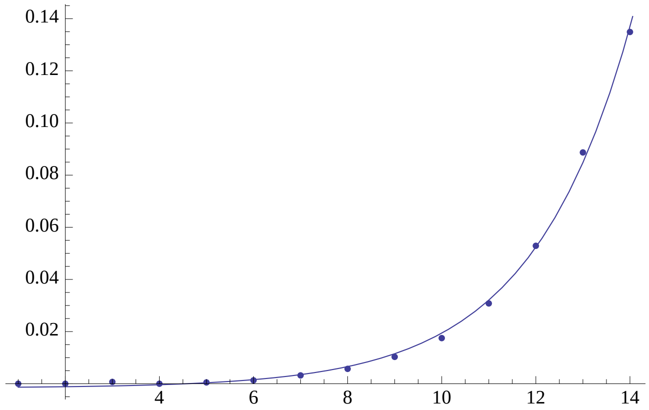


```
X151exp =
 NonlinearModelFit[X151data[[2 ;; 14]], b + d0 * a^x, {b, d0, a}, x, MaxIterations → 1000]
```

FittedModel[$-0.00233291 + 0.000168334 \ll 19 \gg^{x}$]

```
X151exp["BestFit"]
```

$-0.00233291 + 0.000168334\ 1.62716^{x}$

```
Show[ListPlot[X151data[[1 ;; 14]], PlotRange → All], Plot[X151exp[x], {x, 1, 15}]]
```

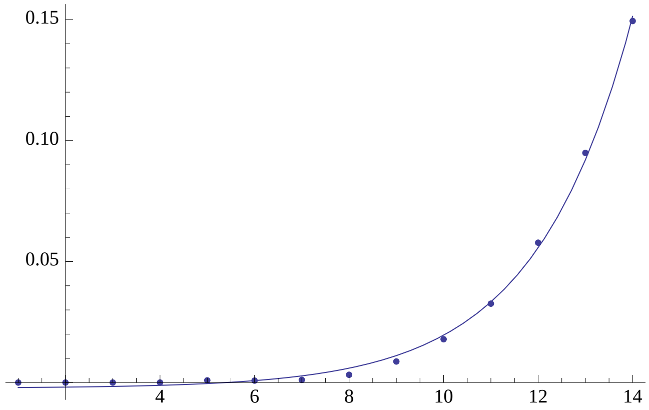


```
X152exp =
 NonlinearModelFit[X152data[[2 ;; 14]], b + d0 * a^x, {b, d0, a}, x, MaxIterations → 1000]
```

FittedModel[$-0.0021698 + 0.000170541 \ll 19 \gg^{x}$]

```
X152exp["BestFit"]
```

$-0.0021698 + 0.000170541\ 1.6316^{x}$

```
Show[ListPlot[X152data[[1 ;; 14]], PlotRange → All], Plot[X152exp[x], {x, 1, 15}]]
```

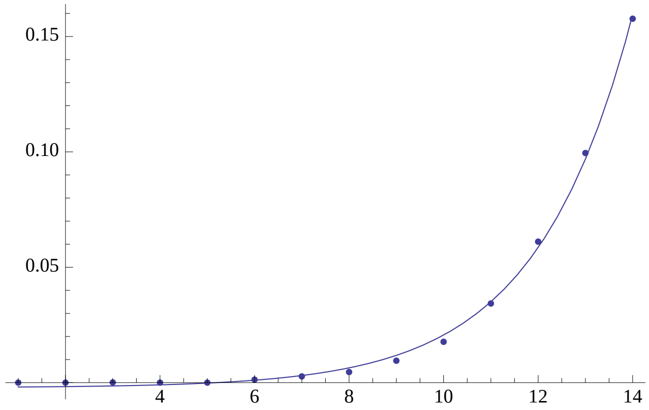


```
X153exp =
 NonlinearModelFit[X153data[[2 ;; 14]], b + d0 * a^x, {b, d0, a}, x, MaxIterations → 1000]
```

FittedModel[$-0.00209415 + 0.000197078 \ll 19 \gg^{x}$]

```
X153exp["BestFit"]
```

$-0.00209415 + 0.000197078\ 1.61031^{x}$

```
Show[ListPlot[X153data[[1 ;; 14]], PlotRange → All], Plot[X153exp[x], {x, 1, 15}]]
```

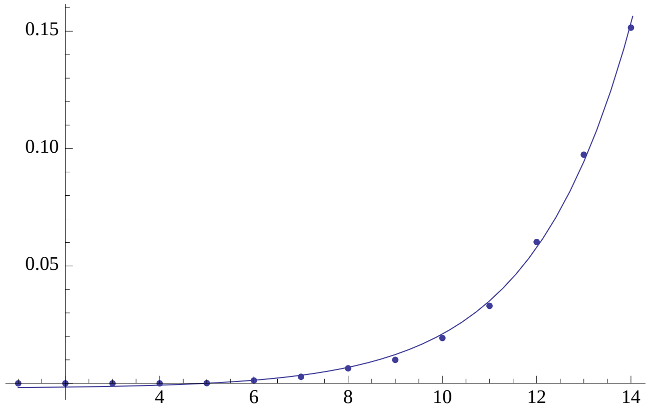


```
X154exp =
 NonlinearModelFit[X154data[[2 ;; 14]], b + d0 * a^x, {b, d0, a}, x, MaxIterations → 1000]
```

FittedModel[$-0.00176367 + 0.000138434 \ll 19 \gg^{x}$]

```
X154exp["BestFit"]
```

$-0.00176367 + 0.000138434\ 1.64197^{x}$

```
Show[ListPlot[X154data[[1 ;; 14]], PlotRange → All], Plot[X154exp[x], {x, 1, 15}]]
```

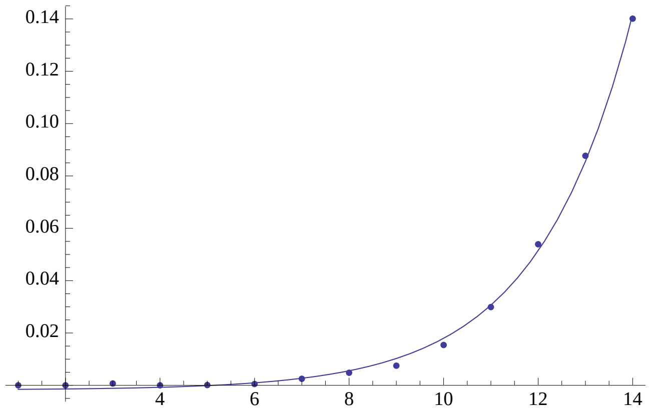


```
X211exp =
 NonlinearModelFit[X211data[[1 ;; 15]], b + d0 * a^x, {b, d0, a}, x, MaxIterations → 1000]
```

FittedModel[$0.000110522 + 6.11733 \times 10^{-6} \ll 19 \gg^{x}$]

```
X211exp["BestFit"]
```

$0.000110522 + 6.11733 \times 10^{-6}\ 1.8182^{x}$

```
Show[ListPlot[X211data[[1 ;; 20]], PlotRange → All], Plot[X211exp[x], {x, 1, 20}]]
```

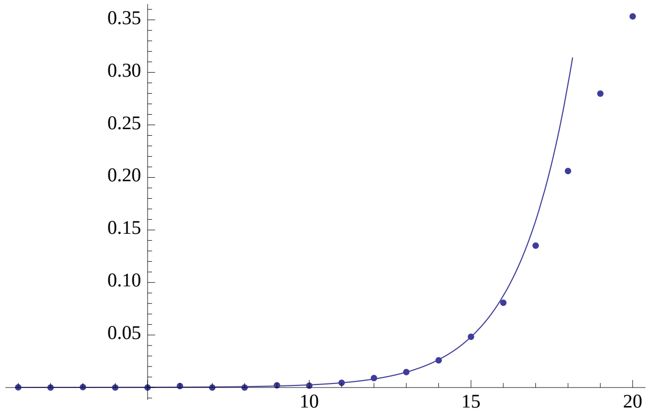


```
X212exp =
 NonlinearModelFit[X212data[[1 ;; 15]], b + d0 * a^x, {b, d0, a}, x, MaxIterations → 1000]
```

FittedModel[$0.000063897 + 2.87221 \times 10^{-6} \ll 19 \gg^{x}$]

```
X212exp["BestFit"]
```

$0.000063897 + 2.87221 \times 10^{-6}\, 1.90837^{x}$

```
Show[ListPlot[X212data[[1 ;; 15]], PlotRange → All], Plot[X212exp[x], {x, 1, 16}]]
```

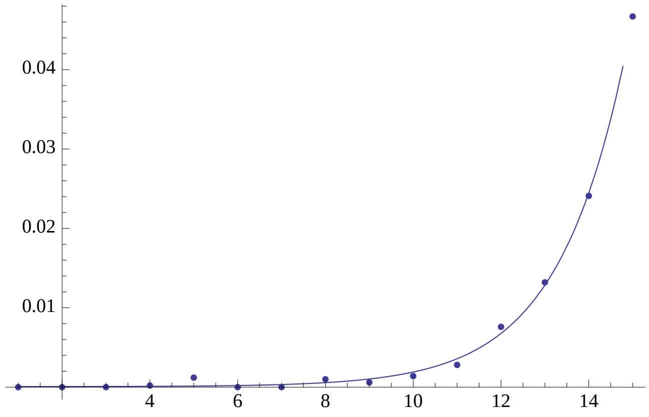


```
X213exp =
 NonlinearModelFit[X213data[[2 ;; 15]], b + d0 * a^x, {b, d0, a}, x, MaxIterations → 1000]
```

FittedModel[$2.46294 \times 10^{-6} + 8.81896 \times 10^{-7} \ll 18 \gg^{x}$]

```
X213exp["BestFit"]
```

$2.46294 \times 10^{-6} + 8.81896 \times 10^{-7}\, 2.02643^{x}$

```
Show[ListPlot[X213data[[1 ;; 15]], PlotRange → All], Plot[X213exp[x], {x, 1, 17}]]
```

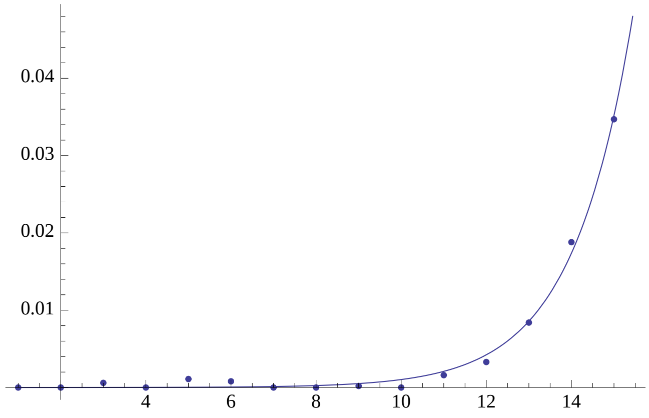


```
X214exp =
 NonlinearModelFit[X214data[[2 ;; 15]], b + d0 * a^x, {b, d0, a}, x, MaxIterations → 1000]
```

FittedModel[$0.000103384 + 3.18872 \times 10^{-6} \ll 19 \gg^{x}$]

```
X214exp["BestFit"]
```

$0.000103384 + 3.18872 \times 10^{-6}\, 1.86465^{x}$

```
Show[ListPlot[X214data[[1 ;; 20]], PlotRange → All], Plot[X214exp[x], {x, 1, 22}]]
```

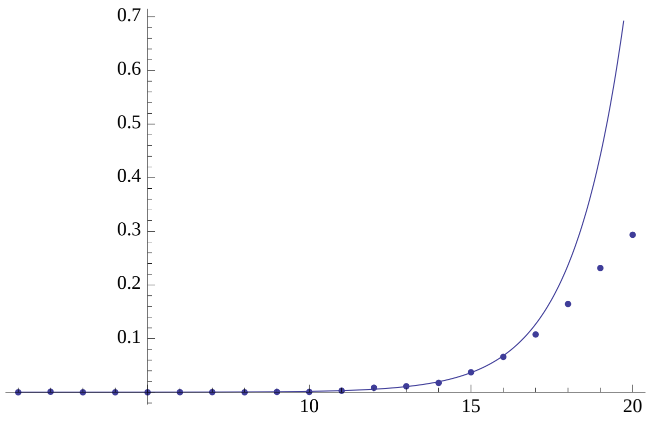


```
X221exp =
 NonlinearModelFit[X221data[[1 ;; 15]], b + d0 * a^x, {b, d0, a}, x, MaxIterations → 1000]
```

FittedModel[$0.0000345803 + 0.0000176973 \ll 19 \gg^{x}$]

```
X221exp["BestFit"]
```

$0.0000345803 + 0.0000176973\, 1.66956^{x}$

```
Show[ListPlot[X221data[[1 ;; 20]], PlotRange → All], Plot[X221exp[x], {x, 1, 20}]]
```

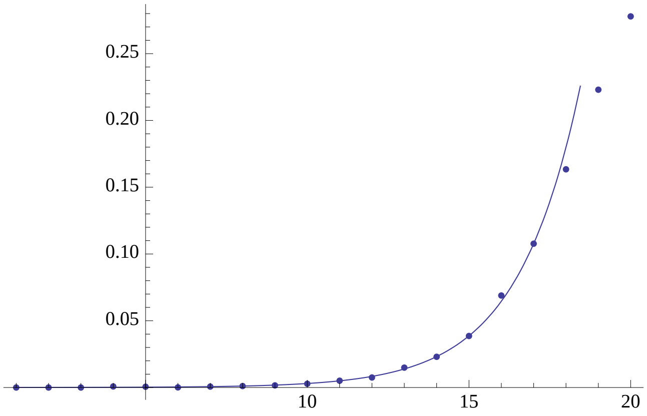


```
X222exp =
 NonlinearModelFit[X222data[[1 ;; 15]], b + d0 * a^x, {b, d0, a}, x, MaxIterations → 1000]
```

FittedModel[$0.000543117 + 8.4109 \times 10^{-6} \ll 19 \gg^{x}$]

```
X222exp["BestFit"]
```

$0.000543117 + 8.4109 \times 10^{-6}\ 1.74762^{x}$

```
Show[ListPlot[X222data[[1 ;; 15]], PlotRange → All], Plot[X222exp[x], {x, 1, 16}]]
```

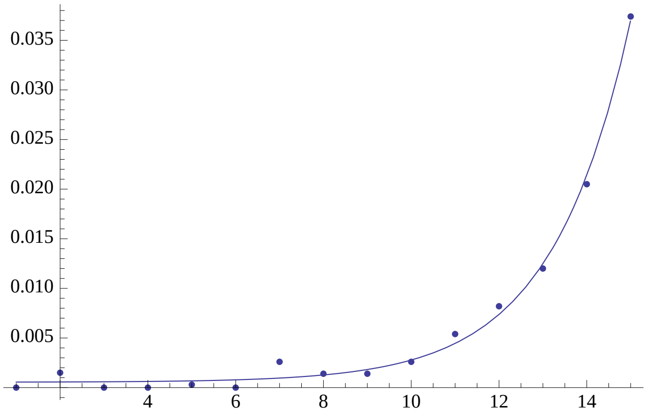


```
X223exp =
 NonlinearModelFit[X223data[[2 ;; 15]], b + d0 * a^x, {b, d0, a}, x, MaxIterations → 1000]
```

FittedModel[$-0.000158784 + 6.77524 \times 10^{-6} \ll 19 \gg^{x}$]

```
X223exp["BestFit"]
```

$-0.000158784 + 6.77524 \times 10^{-6}\ 1.77526^{x}$

```
Show[ListPlot[X223data[[1 ;; 15]], PlotRange → All], Plot[X223exp[x], {x, 1, 17}]]
```

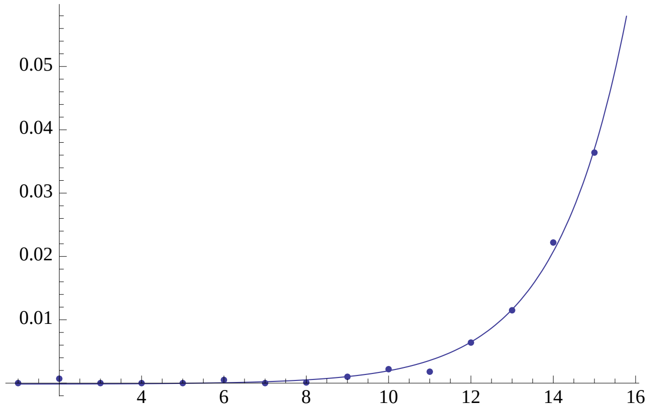


```
X224exp =
 NonlinearModelFit[X224data[[2 ;; 15]], b + d0 * a^x, {b, d0, a}, x, MaxIterations → 1000]
```

FittedModel[$0.000588167 + 5.36974 \times 10^{-6} \ll 19 \gg^{x}$]

```
X224exp["BestFit"]
```

$0.000588167 + 5.36974 \times 10^{-6}\, 1.80439^{x}$

```
Show[ListPlot[X224data[[1 ;; 20]], PlotRange → All], Plot[X224exp[x], {x, 1, 22}]]
```

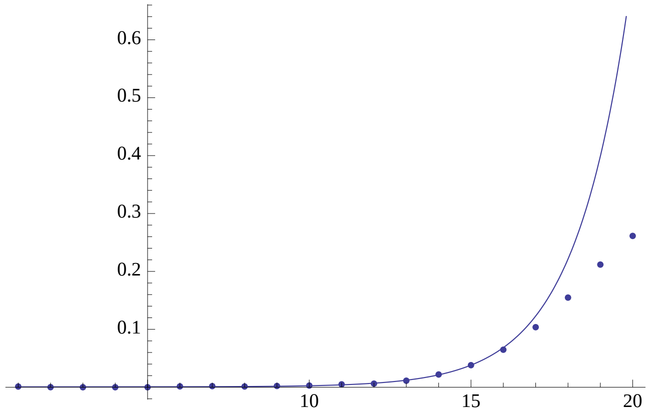


```
X231exp =
 NonlinearModelFit[X231data[[1 ;; 15]], b + d0 * a^x, {b, d0, a}, x, MaxIterations → 1000]
```

FittedModel[$-0.0000314852 + 9.43912 \times 10^{-6} \ll 19 \gg^{x}$]

```
X231exp["BestFit"]
```

$-0.0000314852 + 9.43912 \times 10^{-6}\, 1.74424^{x}$

```
Show[ListPlot[X231data[[1 ;; 20]], PlotRange → All], Plot[X231exp[x], {x, 1, 20}]]
```

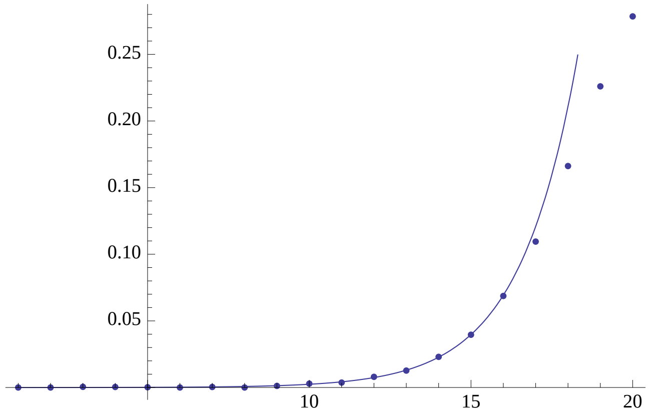


```
X232exp =
 NonlinearModelFit[X232data[[1 ;; 15]], b + d0 * a^x, {b, d0, a}, x, MaxIterations → 1000]
```

FittedModel[$0.000101681 + 6.19129\times10^{-6} \ll19\gg^{x}$]

```
X232exp["BestFit"]
```

$0.000101681 + 6.19129\times10^{-6}\, 1.78595^{x}$

```
Show[ListPlot[X232data[[1 ;; 15]], PlotRange → All], Plot[X232exp[x], {x, 1, 16}]]
```

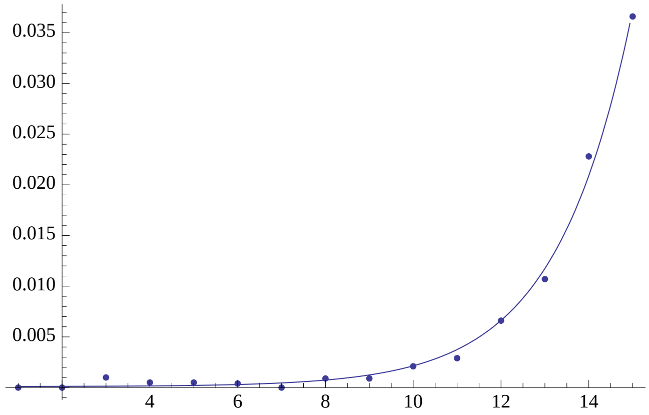


```
X233exp =
 NonlinearModelFit[X233data[[2 ;; 15]], b + d0 * a^x, {b, d0, a}, x, MaxIterations → 1000]
```

FittedModel[$0.000396906 + 0.0000190444 \ll19\gg^{x}$]

```
X233exp["BestFit"]
```

$0.000396906 + 0.0000190444\, 1.65027^{x}$

```
Show[ListPlot[X233data[[1 ;; 15]], PlotRange → All], Plot[X233exp[x], {x, 1, 17}]]
```

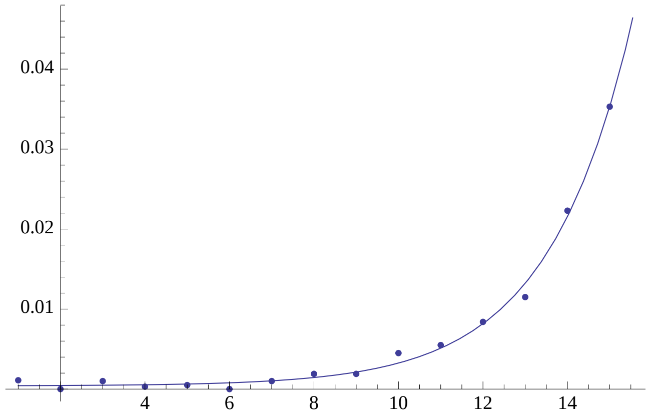


```
X234exp =
 NonlinearModelFit[X234data[[2 ;; 15]], b + d0 * a^x, {b, d0, a}, x, MaxIterations → 1000]
```

FittedModel[$0.000286217 + 5.04736\times10^{-6} \ll 19 \gg^{x}$]

```
X234exp["BestFit"]
```

$0.000286217 + 5.04736 \times 10^{-6}\, 1.80303^{x}$

```
Show[ListPlot[X234data[[1 ;; 20]], PlotRange → All], Plot[X234exp[x], {x, 1, 22}]]
```

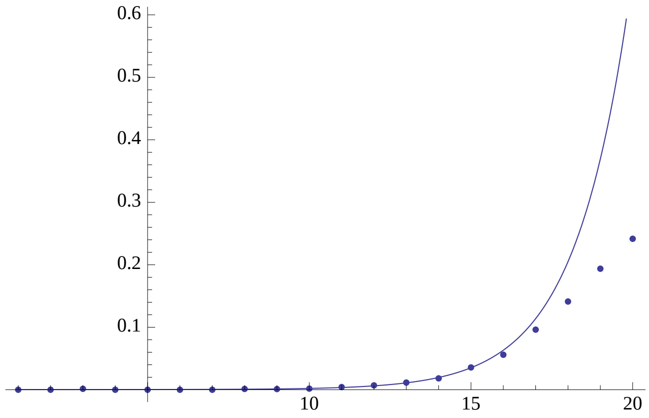


```
X241exp =
 NonlinearModelFit[X241data[[1 ;; 15]], b + d0 * a^x, {b, d0, a}, x, MaxIterations → 1000]
```

FittedModel[$0.0000187071 + 0.0000100793 \ll 19 \gg^{x}$]

```
X241exp["BestFit"]
```

$0.0000187071 + 0.0000100793\, 1.75597^{x}$

```
Show[ListPlot[X241data[[1 ;; 20]], PlotRange → All], Plot[X241exp[x], {x, 1, 20}]]
```

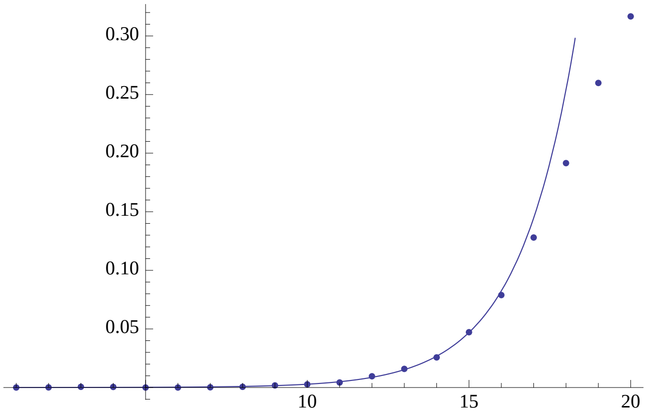


```
X242exp =
 NonlinearModelFit[X242data[[1 ;; 15]], b + d0 * a^x, {b, d0, a}, x, MaxIterations → 1000]
```

FittedModel[$0.000103583 + 5.04605\times10^{-6} \ll 19 \gg^{x}$]

```
X242exp["BestFit"]
```

$0.000103583 + 5.04605 \times 10^{-6}\, 1.81483^{x}$

```
Show[ListPlot[X242data[[1 ;; 15]], PlotRange → All], Plot[X242exp[x], {x, 1, 16}]]
```

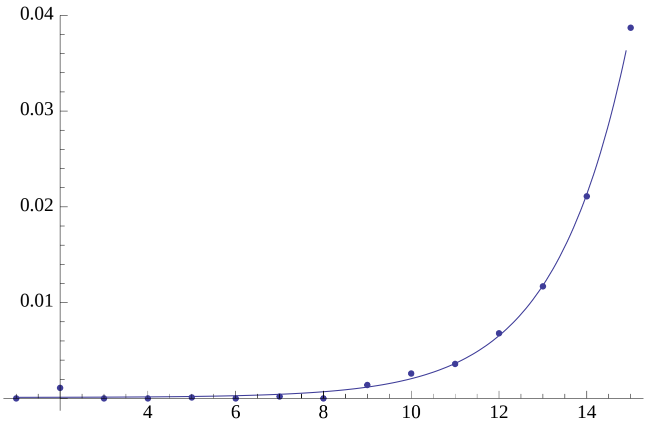


```
X243exp =
 NonlinearModelFit[X243data[[2 ;; 15]], b + d0 * a^x, {b, d0, a}, x, MaxIterations → 1000]
```

FittedModel[$-0.0000868314 + 5.18919\times10^{-6} \ll 19 \gg^{x}$]

```
X243exp["BestFit"]
```

$-0.0000868314 + 5.18919 \times 10^{-6}\, 1.78797^{x}$

```
Show[ListPlot[X243data[[1 ;; 15]], PlotRange → All], Plot[X243exp[x], {x, 1, 17}]]
```

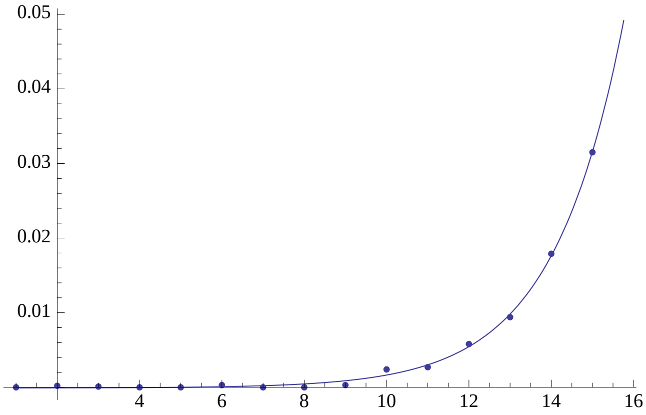


```
X244exp =
 NonlinearModelFit[X244data[[2 ;; 15]], b + d0 * a^x, {b, d0, a}, x, MaxIterations → 1000]
```

FittedModel[$-6.79183\times10^{-6} + 4.03432\times10^{-6}$ ≪19≫^{x}]

```
X244exp["BestFit"]
```

$-6.79183\times10^{-6} + 4.03432\times10^{-6}\, 1.81664^x$

```
Show[ListPlot[X244data[[1 ;; 20]], PlotRange → All], Plot[X244exp[x], {x, 1, 22}]]
```

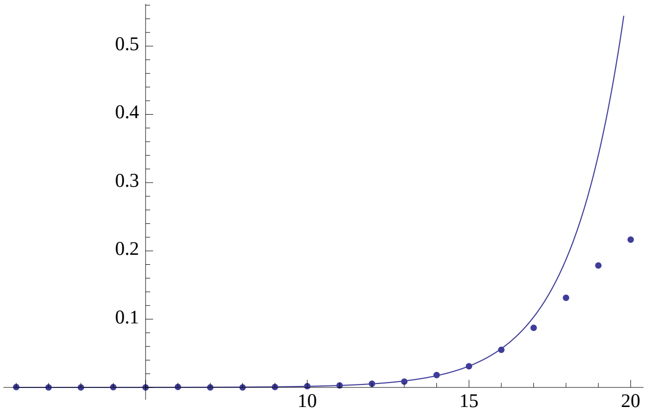


```
X251exp =
 NonlinearModelFit[X251data[[1 ;; 15]], b + d0 * a^x, {b, d0, a}, x, MaxIterations → 1000]
```

FittedModel[$0.000279727 + 0.0000186937$ ≪19≫^{x}]

```
X251exp["BestFit"]
```

$0.000279727 + 0.0000186937\, 1.66883^x$

```
Show[ListPlot[X251data[[1 ;; 20]], PlotRange → All], Plot[X251exp[x], {x, 1, 20}]]
```

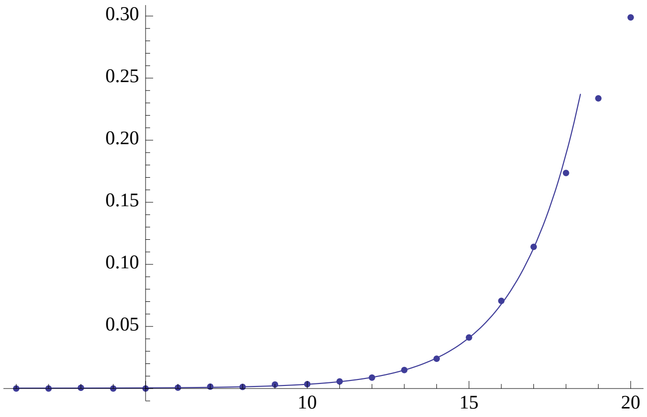


```
X252exp =
 NonlinearModelFit[X252data[[1 ;; 15]], b + d0 * a^x, {b, d0, a}, x, MaxIterations → 1000]
```

FittedModel[$0.0000186645 + 9.61072\times10^{-6} \ll 19 \gg^{x}$]

```
X252exp["BestFit"]
```

$0.0000186645 + 9.61072\times10^{-6}\, 1.74947^{x}$

```
Show[ListPlot[X252data[[1 ;; 15]], PlotRange → All], Plot[X252exp[x], {x, 1, 16}]]
```

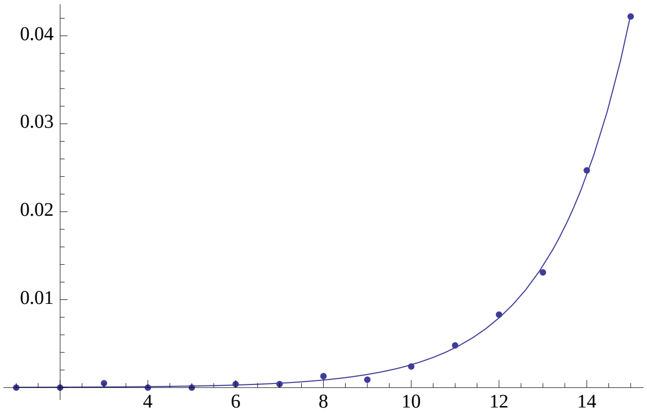


```
X253exp =
 NonlinearModelFit[X253data[[2 ;; 15]], b + d0 * a^x, {b, d0, a}, x, MaxIterations → 1000]
```

FittedModel[$0.0000711672 + 5.78513\times10^{-6} \ll 19 \gg^{x}$]

```
X253exp["BestFit"]
```

$0.0000711672 + 5.78513\times10^{-6}\, 1.78574^{x}$

```
Show[ListPlot[X253data[[1 ;; 15]], PlotRange → All], Plot[X253exp[x], {x, 1, 17}]]
```

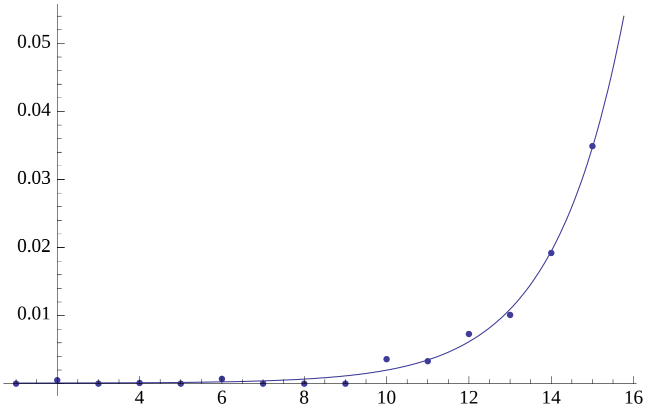


```
X254exp =
 NonlinearModelFit[X254data[[2 ;; 15]], b + d0 * a^x, {b, d0, a}, x, MaxIterations → 1000]
```

FittedModel[$0.000550572 + 2.88881\times 10^{-6} \ll 19 \gg^{x}$]

```
X254exp["BestFit"]
```

$0.000550572 + 2.88881 \times 10^{-6}\, 1.87563^{x}$

```
Show[ListPlot[X254data[[1 ;; 20]], PlotRange → All], Plot[X254exp[x], {x, 1, 22}]]
```

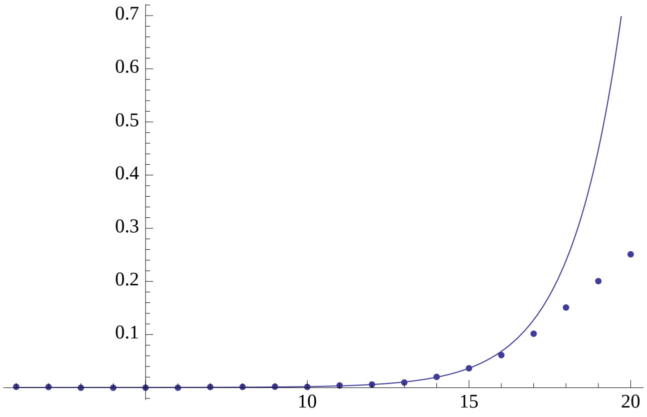


```
X311exp =
 NonlinearModelFit[X311data[[2 ;; 19]], b + d0 * a^x, {b, d0, a}, x, MaxIterations → 1000]
```

FittedModel[$0.000297039 + 2.37729\times 10^{-7} \ll 18 \gg^{x}$]

```
X311exp["BestFit"]
```

$0.000297039 + 2.37729 \times 10^{-7}\, 1.88632^{x}$

```
Show[ListPlot[X311data[[1 ;; 19]], PlotRange → All], Plot[X311exp[x], {x, 1, 21}]]
```

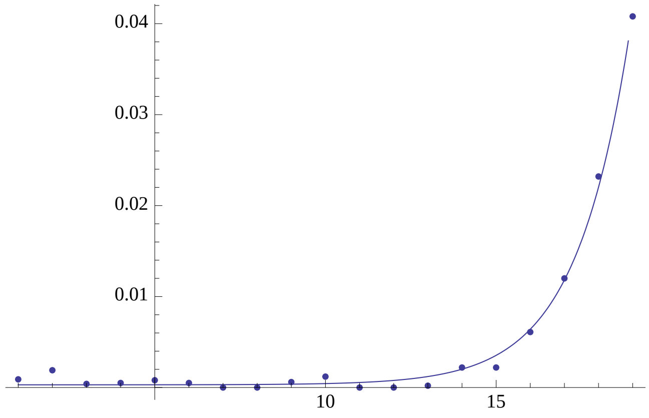


```
X312exp =
 NonlinearModelFit[X312data[[2 ;; 19]], b + d0 * a^x, {b, d0, a}, x, MaxIterations → 1000]
```

FittedModel[$0.000161324 + 7.06402\times10^{-7} \ll 19 \gg^{x}$]

```
X312exp["BestFit"]
```

$0.000161324 + 7.06402 \times 10^{-7}\, 1.78783^{x}$

```
Show[ListPlot[X312data[[1 ;; 19]], PlotRange → All], Plot[X312exp[x], {x, 1, 21}]]
```

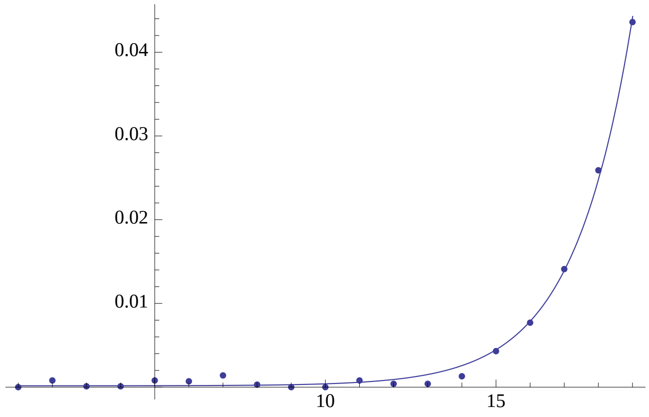


```
X313exp =
 NonlinearModelFit[X313data[[2 ;; 19]], b + d0 * a^x, {b, d0, a}, x, MaxIterations → 1000]
```

FittedModel[$-0.000109957 + 1.40857\times10^{-6} \ll 19 \gg^{x}$]

```
X313exp["BestFit"]
```

$-0.000109957 + 1.40857 \times 10^{-6}\, 1.70873^{x}$

```
Show[ListPlot[X313data[[1 ;; 19]], PlotRange → All], Plot[X313exp[x], {x, 1, 21}]]
```

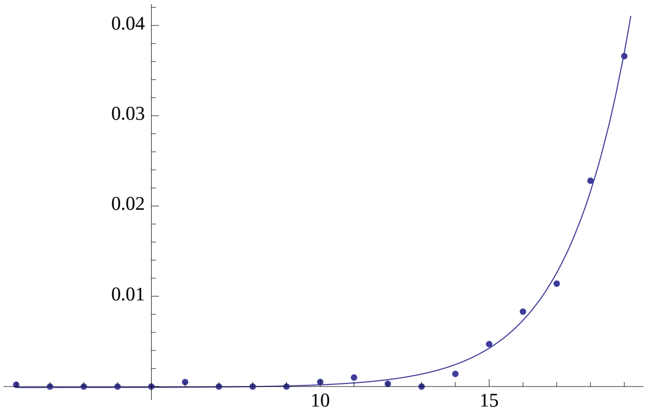


```
X314exp =
 NonlinearModelFit[X314data[[2 ;; 19]], b + d0 * a^x, {b, d0, a}, x, MaxIterations → 1000]
```

FittedModel[$0.000290109 + 7.68663\times10^{-7} \ll 19\gg^{x}$]

```
X314exp["BestFit"]
```

$0.000290109 + 7.68663 \times 10^{-7}\, 1.76487^{x}$

```
Show[ListPlot[X314data[[1 ;; 19]], PlotRange → All], Plot[X314exp[x], {x, 1, 21}]]
```

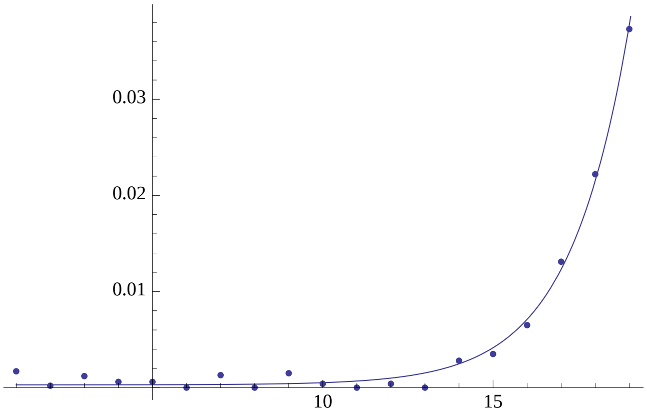


```
X321exp =
 NonlinearModelFit[X321data[[2 ;; 19]], b + d0 * a^x, {b, d0, a}, x, MaxIterations → 1000]
```

FittedModel[$-0.000171617 + 5.09911\times10^{-7} \ll 19\gg^{x}$]

```
X321exp["BestFit"]
```

$-0.000171617 + 5.09911 \times 10^{-7}\, 1.80669^{x}$

```
Show[ListPlot[X321data[[1 ;; 20]], PlotRange → All], Plot[X321exp[x], {x, 1, 20}]]
```

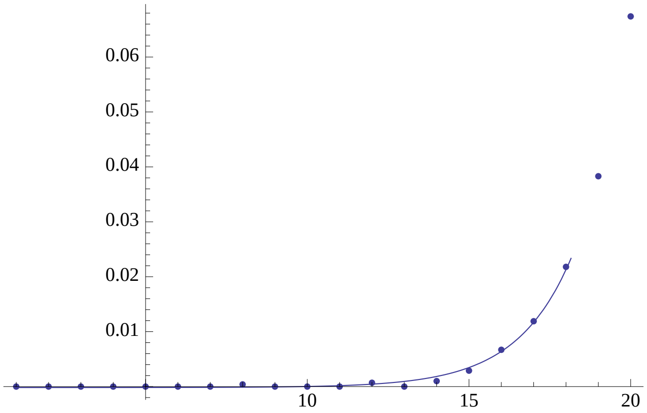


```
X322exp =
 NonlinearModelFit[X322data[[2 ;; 19]], b + d0 * a^x, {b, d0, a}, x, MaxIterations → 1000]
```

FittedModel[$-0.000117151 + 5.72873\times10^{-7} \ll18\gg^{x}$]

```
X322exp["BestFit"]
```

$-0.000117151 + 5.72873\times10^{-7}\, 1.80331^{x}$

```
Show[ListPlot[X322data[[1 ;; 19]], PlotRange → All], Plot[X322exp[x], {x, 1, 22}]]
```

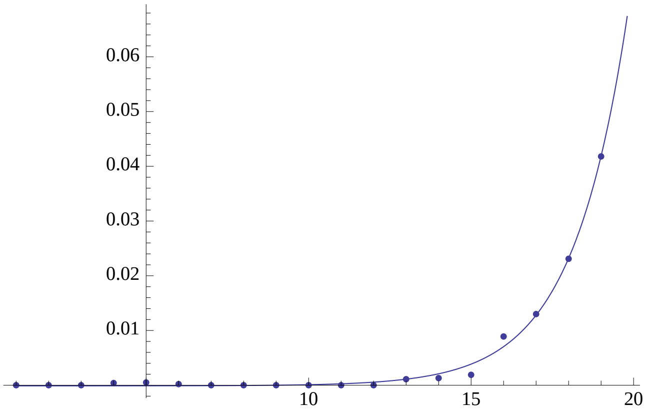


```
X323exp =
 NonlinearModelFit[X323data[[2 ;; 19]], b + d0 * a^x, {b, d0, a}, x, MaxIterations → 1000]
```

FittedModel[$-0.0000529368 + 8.26306\times10^{-7} \ll18\gg^{x}$]

```
X323exp["BestFit"]
```

$-0.0000529368 + 8.26306\times10^{-7}\, 1.78125^{x}$

```
Show[ListPlot[X323data[[1 ;; 19]], PlotRange → All], Plot[X323exp[x], {x, 1, 21}]]
```

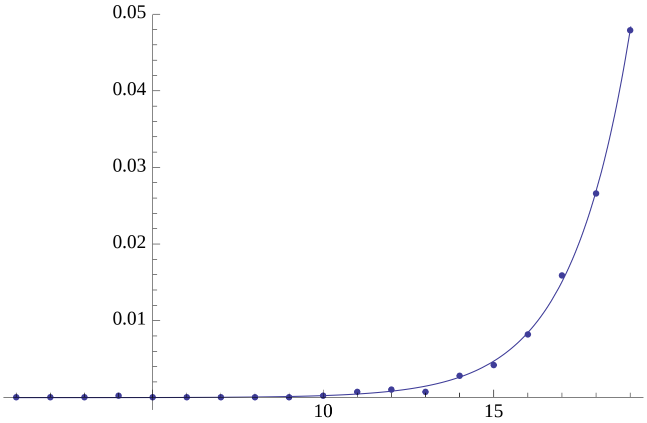


```
X324exp =
 NonlinearModelFit[X324data[[2 ;; 19]], b + d0 * a^x, {b, d0, a}, x, MaxIterations → 1000]
```

FittedModel[$-0.000136721 + 6.20397 \times 10^{-7} \ll 19 \gg^{x}$]

```
X324exp["BestFit"]
```

$-0.000136721 + 6.20397 \times 10^{-7}\, 1.81211^{x}$

```
Show[ListPlot[X324data[[1 ;; 20]], PlotRange → All], Plot[X324exp[x], {x, 1, 22}]]
```

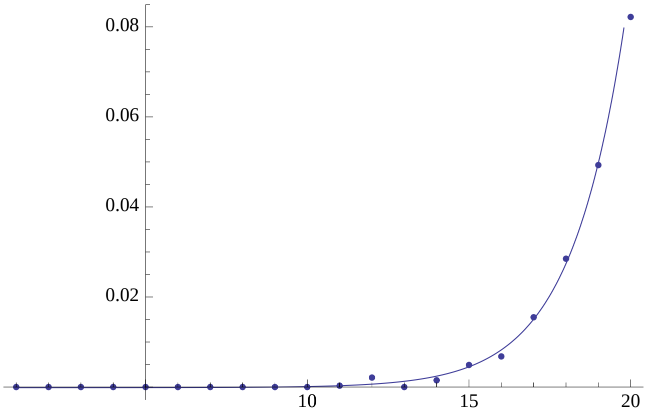


```
X331exp =
 NonlinearModelFit[X331data[[2 ;; 19]], b + d0 * a^x, {b, d0, a}, x, MaxIterations → 1000]
```

FittedModel[$-0.0000362272 + 4.23537 \times 10^{-7} \ll 18 \gg^{x}$]

```
X331exp["BestFit"]
```

$-0.0000362272 + 4.23537 \times 10^{-7}\, 1.84691^{x}$

```
Show[ListPlot[X331data[[1 ;; 20]], PlotRange → All], Plot[X331exp[x], {x, 1, 23}]]
```

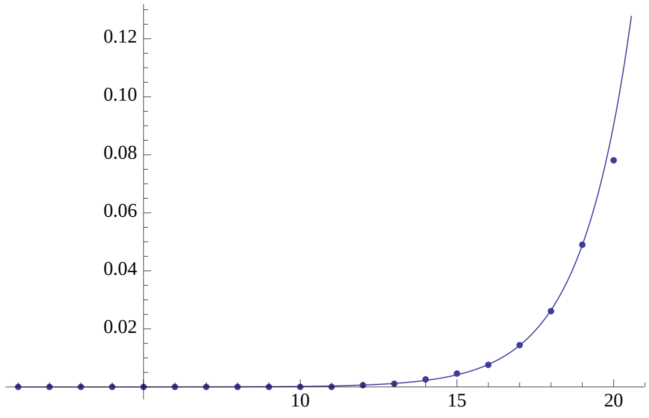


```
X332exp =
 NonlinearModelFit[X332data[[10 ;; 19]], b + d0 * a^x, {b, d0, a}, x, MaxIterations → 1000]
```

FittedModel[$-0.000677626 + 6.47734 \times 10^{-7} \ll 19 \gg^{x}$]

```
X332exp["BestFit"]
```

$-0.000677626 + 6.47734 \times 10^{-7}\, 1.82007^{x}$

```
Show[ListPlot[X332data[[1 ;; 19]], PlotRange → All], Plot[X332exp[x], {x, 1, 22}]]
```

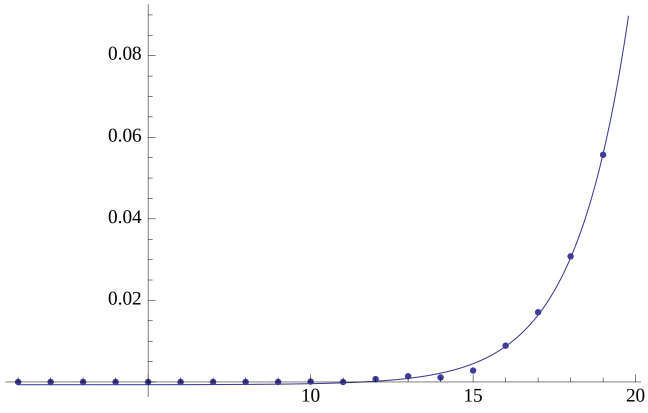


```
X333exp =
 NonlinearModelFit[X333data[[2 ;; 19]], b + d0 * a^x, {b, d0, a}, x, MaxIterations → 1000]
```

FittedModel[$-0.00014129 + 6.76233 \times 10^{-7} \ll 19 \gg^{x}$]

```
X333exp["BestFit"]
```

$-0.00014129 + 6.76233 \times 10^{-7}\, 1.80744^{x}$

```
Show[ListPlot[X333data[[1 ;; 19]], PlotRange → All], Plot[X333exp[x], {x, 1, 22}]]
```

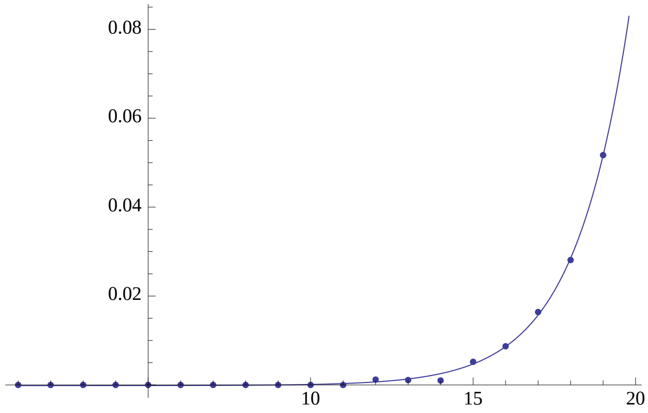


```
X334exp =
 NonlinearModelFit[X334data[[10 ;; 19]], b + d0 * a^x, {b, d0, a}, x, MaxIterations → 1000]
```

FittedModel[$-0.000581152 + 1.2912\times10^{-6} \ll 19 \gg^{x}$]

```
X334exp["BestFit"]
```

$-0.000581152 + 1.2912\times10^{-6}\, 1.754^{x}$

```
Show[ListPlot[X334data[[1 ;; 20]], PlotRange → All], Plot[X334exp[x], {x, 1, 22}]]
```

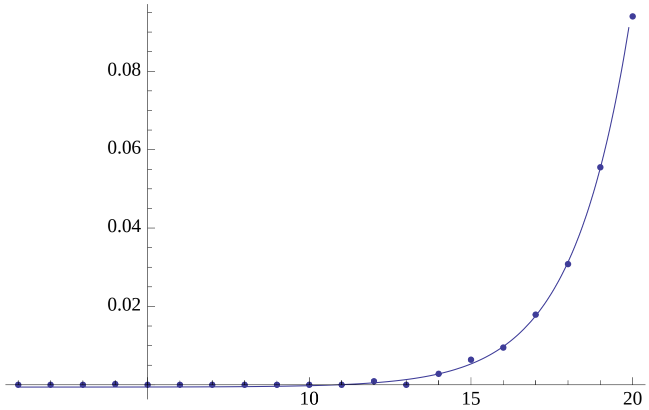


```
X341exp =
 NonlinearModelFit[X341data[[2 ;; 19]], b + d0 * a^x, {b, d0, a}, x, MaxIterations → 1000]
```

FittedModel[$0.000197982 + 5.11667\times10^{-7} \ll 19 \gg^{x}$]

```
X341exp["BestFit"]
```

$0.000197982 + 5.11667\times10^{-7}\, 1.80593^{x}$

```
Show[ListPlot[X341data[[1 ;; 20]], PlotRange → All], Plot[X341exp[x], {x, 1, 22}]]
```

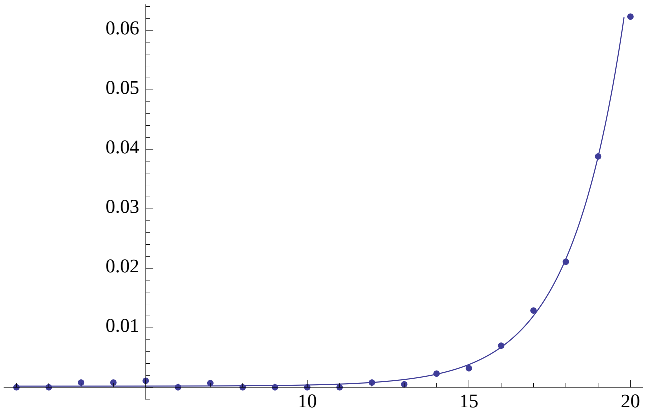


```
X342exp =
 NonlinearModelFit[X342data[[2 ;; 19]], b + d0 * a^x, {b, d0, a}, x, MaxIterations → 1000]
```

FittedModel[$-0.000130338 + 8.42584\times10^{-7}\ \ll 19 \gg^{x}$]

```
X342exp["BestFit"]
```

$-0.000130338 + 8.42584\times10^{-7}\ 1.77362^{x}$

```
Show[ListPlot[X342data[[1 ;; 20]], PlotRange → All], Plot[X342exp[x], {x, 1, 22}]]
```

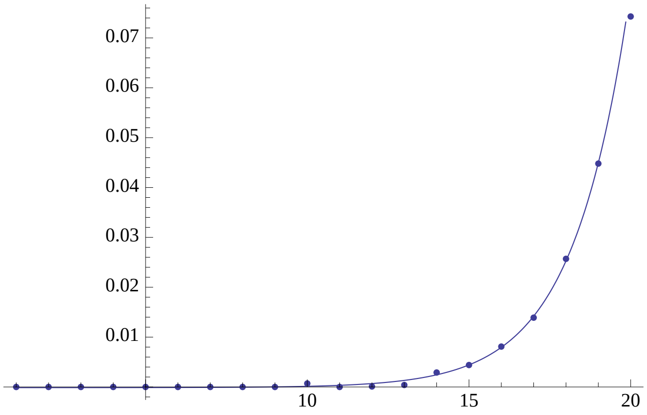


```
X343exp =
 NonlinearModelFit[X343data[[2 ;; 19]], b + d0 * a^x, {b, d0, a}, x, MaxIterations → 1000]
```

FittedModel[$-0.000264434 + 1.40858\times10^{-6}\ \ll 19 \gg^{x}$]

```
X343exp["BestFit"]
```

$-0.000264434 + 1.40858\times10^{-6}\ 1.71202^{x}$

```
Show[ListPlot[X343data[[1 ;; 20]], PlotRange → All], Plot[X343exp[x], {x, 1, 22}]]
```

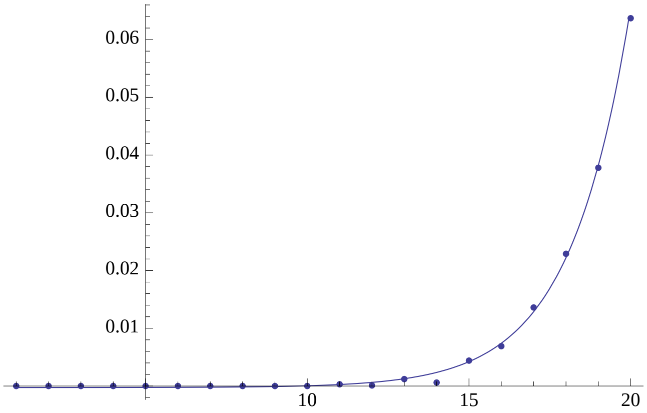


```
X344exp =
 NonlinearModelFit[X344data[[2 ;; 19]], b + d0 * a^x, {b, d0, a}, x, MaxIterations → 1000]
```

FittedModel[$-0.000290228 + 2.38968\times10^{-6} \ll 18 \gg^{x}$]

```
X344exp["BestFit"]
```

$-0.000290228 + 2.38968\times10^{-6}\, 1.66801^{x}$

```
Show[ListPlot[X344data[[1 ;; 20]], PlotRange → All], Plot[X344exp[x], {x, 1, 22}]]
```

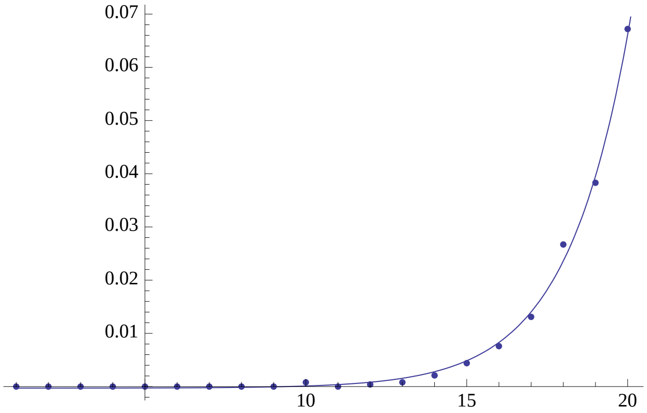


```
X351exp =
 NonlinearModelFit[X351data[[2 ;; 19]], b + d0 * a^x, {b, d0, a}, x, MaxIterations → 1000]
```

FittedModel[$-0.000056511 + 9.06115\times10^{-7} \ll 19 \gg^{x}$]

```
X351exp["BestFit"]
```

$-0.000056511 + 9.06115\times10^{-7}\, 1.7564^{x}$

```
Show[ListPlot[X351data[[1 ;; 20]], PlotRange → All], Plot[X351exp[x], {x, 1, 22}]]
```

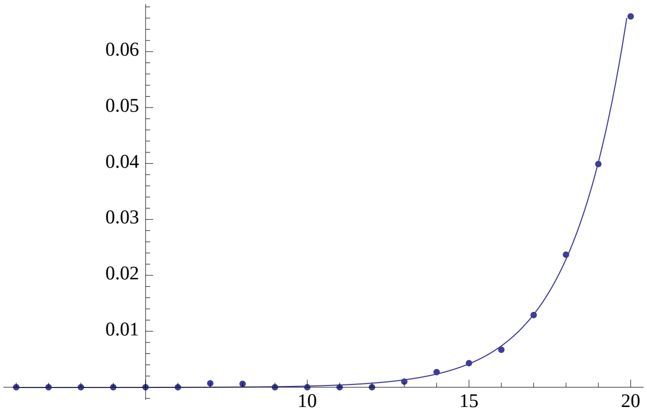


```
X352exp =
 NonlinearModelFit[X352data[[2 ;; 19]], b + d0 * a^x, {b, d0, a}, x, MaxIterations → 1000]
```

FittedModel[$-0.0000753951 + 4.33935\times10^{-7}\ \ll 19\gg^{x}$]

```
X352exp["BestFit"]
```

$-0.0000753951 + 4.33935\times10^{-7}\ 1.84402^{x}$

```
Show[ListPlot[X352data[[1 ;; 20]], PlotRange → All], Plot[X352exp[x], {x, 1, 22}]]
```

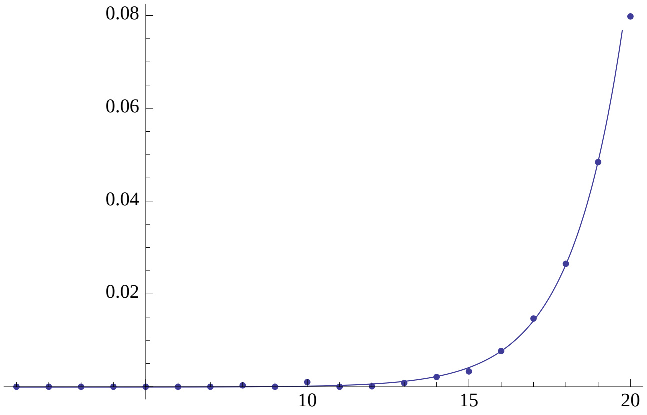


```
X353exp =
 NonlinearModelFit[X353data[[2 ;; 19]], b + d0 * a^x, {b, d0, a}, x, MaxIterations → 1000]
```

FittedModel[$0.0000138645 + 5.21369\times10^{-7}\ \ll 18\gg^{x}$]

```
X353exp["BestFit"]
```

$0.0000138645 + 5.21369\times10^{-7}\ 1.82324^{x}$

```
Show[ListPlot[X353data[[1 ;; 20]], PlotRange → All], Plot[X353exp[x], {x, 1, 22}]]
```

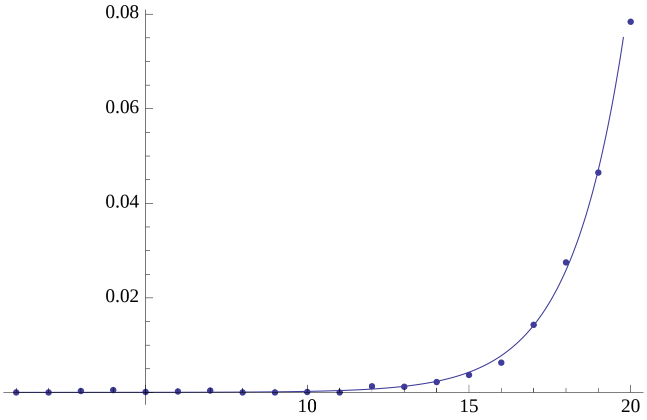


```
X354exp =
 NonlinearModelFit[X354data[[5 ;; 20]], b + d0 * a^x, {b, d0, a}, x, MaxIterations → 1000]
```

FittedModel[$-0.00024931 + 1.32729\times10^{-6} \ll 19 \gg^{x}$]

```
X354exp["BestFit"]
```

$-0.00024931 + 1.32729 \times 10^{-6}\ 1.75395^{x}$

```
Show[ListPlot[X354data[[1 ;; 20]], PlotRange → All], Plot[X354exp[x], {x, 1, 22}]]
```

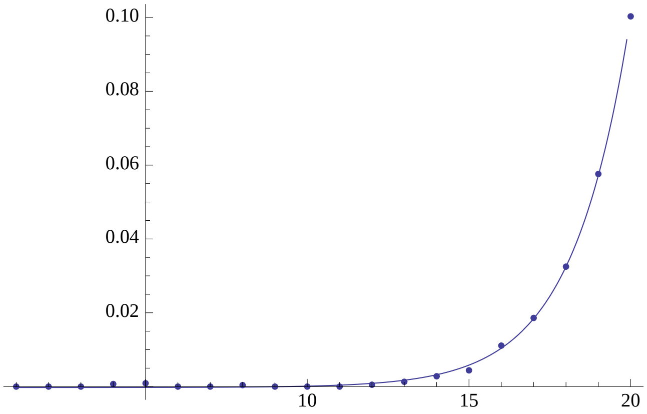


```
X411exp =
 NonlinearModelFit[X411data[[13 ;; 24]], b + d0 * a^x, {b, d0, a}, x, MaxIterations → 1000]
```

FittedModel[$-0.00098573 + 3.4953\times10^{-7} \ll 19 \gg^{x}$]

```
X411exp["BestFit"]
```

$-0.00098573 + 3.4953 \times 10^{-7}\ 1.66586^{x}$

```
Show[ListPlot[X411data[[1 ;; 26]], PlotRange → All], Plot[X411exp[x], {x, 1, 28}]]
```

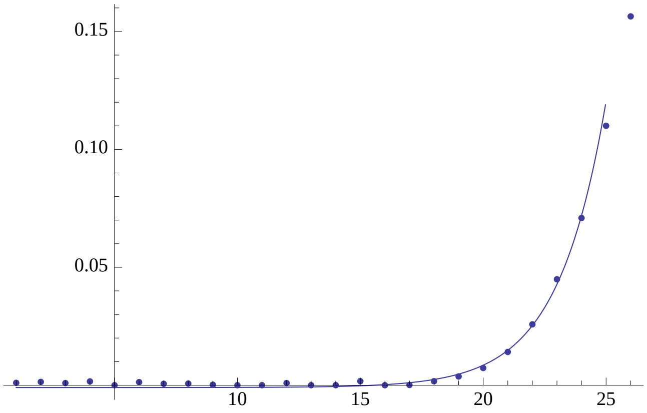


```
X412exp =
 NonlinearModelFit[X412data[[13 ;; 24]], b + d0 * a^x, {b, d0, a}, x, MaxIterations → 1000]
```

FittedModel[$-0.000772384 + 3.98604\times10^{-7}$ ≪19≫^{x}]

```
X412exp["BestFit"]
```

$-0.000772384 + 3.98604\times10^{-7}\ 1.65521^{x}$

```
Show[ListPlot[X412data[[1 ;; 26]], PlotRange → All], Plot[X412exp[x], {x, 1, 29}]]
```

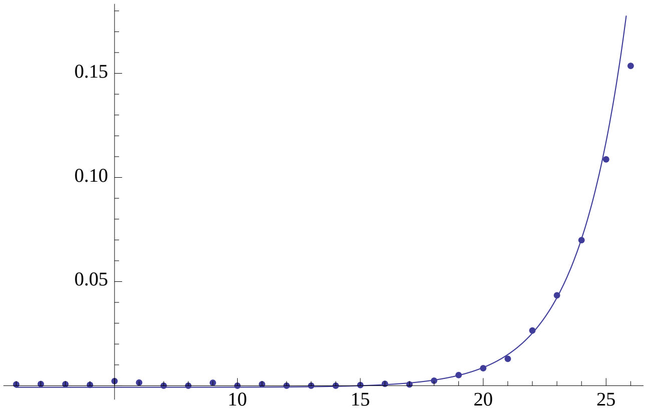


```
X413exp =
 NonlinearModelFit[X413data[[13 ;; 24]], b + d0 * a^x, {b, d0, a}, x, MaxIterations → 1000]
```

FittedModel[$-0.000930879 + 1.87063\times10^{-7}$ ≪19≫^{x}]

```
X413exp["BestFit"]
```

$-0.000930879 + 1.87063\times10^{-7}\ 1.70983^{x}$

```
Show[ListPlot[X413data[[1 ;; 24]], PlotRange → All], Plot[X413exp[x], {x, 1, 28}]]
```

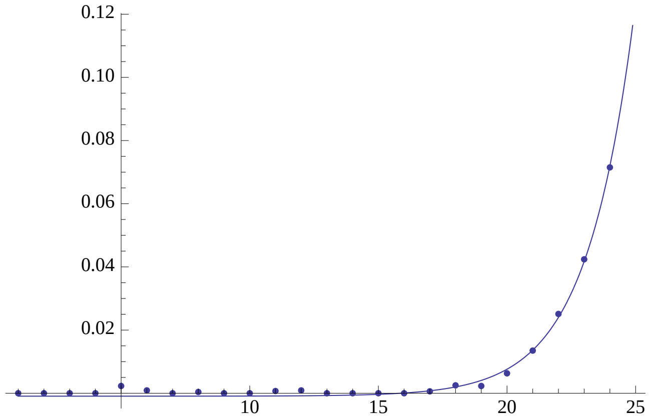


```
X414exp =
 NonlinearModelFit[X414data[[13 ;; 24]], b + d0 * a^x, {b, d0, a}, x, MaxIterations → 1000]
```

FittedModel[$-0.000674251 + 2.07316\times10^{-7}\ll19\gg^{x}$]

```
X414exp["BestFit"]
```

$-0.000674251 + 2.07316\times10^{-7}\,1.70565^{x}$

```
Show[ListPlot[X414data[[1 ;; 24]], PlotRange → All], Plot[X414exp[x], {x, 1, 28}]]
```

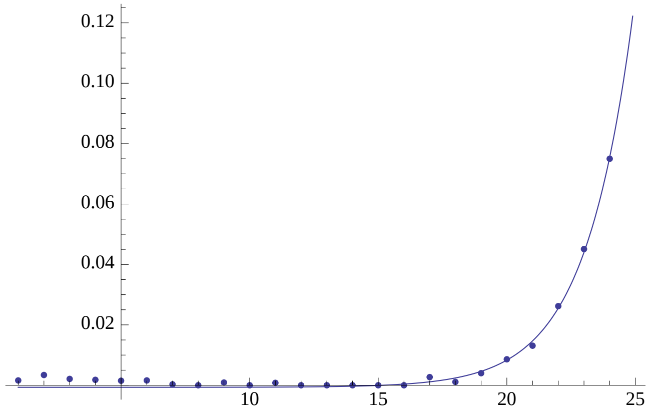


```
X421exp =
 NonlinearModelFit[X421data[[13 ;; 24]], b + d0 * a^x, {b, d0, a}, x, MaxIterations → 1000]
```

FittedModel[$-0.000618286 + 3.8546\times10^{-7}\ll19\gg^{x}$]

```
X421exp["BestFit"]
```

$-0.000618286 + 3.8546\times10^{-7}\,1.66564^{x}$

```
Show[ListPlot[X421data[[1 ;; 23]], PlotRange → All], Plot[X421exp[x], {x, 1, 26}]]
```

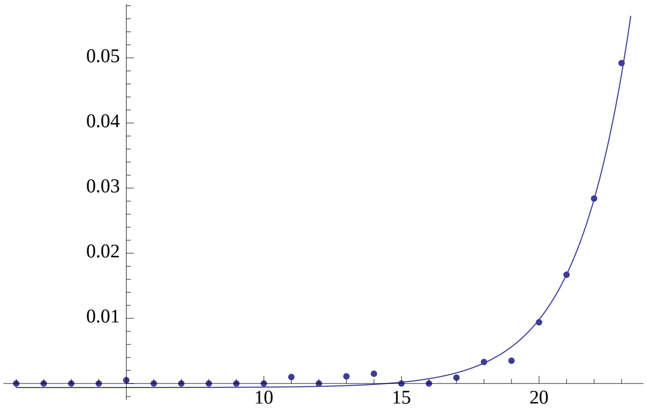


```
X422exp =
 NonlinearModelFit[X422data[[10 ;; 23]], b + d0 * a^x, {b, d0, a}, x, MaxIterations → 1000]
```

FittedModel[$-0.000032854 + 1.83662\times10^{-7} \ll19\gg^{x}$]

```
X422exp["BestFit"]
```

$-0.000032854 + 1.83662 \times 10^{-7}\, 1.73312^{x}$

```
Show[ListPlot[X422data[[1 ;; 23]], PlotRange → All], Plot[X422exp[x], {x, 1, 26}]]
```

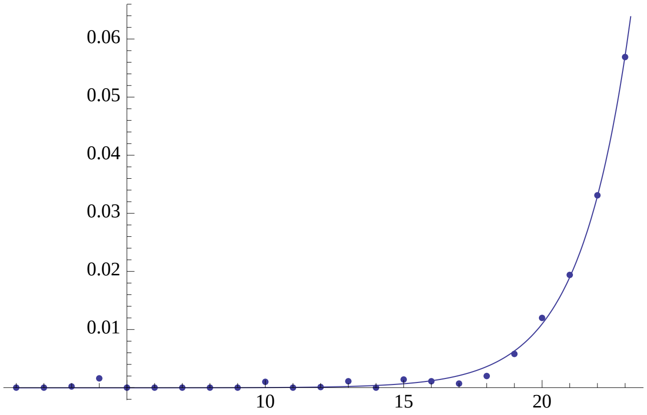


```
X423exp =
 NonlinearModelFit[X423data[[12 ;; 25]], b + d0 * a^x, {b, d0, a}, x, MaxIterations → 1000]
```

FittedModel[$-0.0013683 + 1.28066\times10^{-6} \ll19\gg^{x}$]

```
X423exp["BestFit"]
```

$-0.0013683 + 1.28066 \times 10^{-6}\, 1.58555^{x}$

```
Show[ListPlot[X423data[[1 ;; 23]], PlotRange → All], Plot[X423exp[x], {x, 1, 26}]]
```

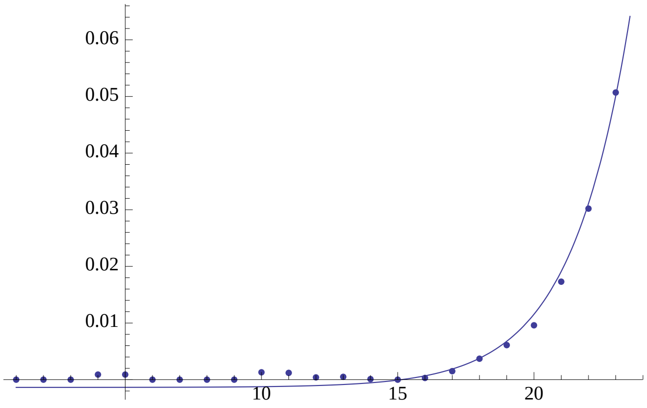


```
X424exp =
 NonlinearModelFit[X424data[[12 ;; 25]], b + d0 * a^x, {b, d0, a}, x, MaxIterations → 1000]
```

FittedModel[$-0.000183795 + 9.28537\times10^{-7} \ll19\gg^{x}$]

```
X424exp["BestFit"]
```

$-0.000183795 + 9.28537\times10^{-7}\,1.60154^{x}$

```
Show[ListPlot[X424data[[1 ;; 25]], PlotRange → All], Plot[X424exp[x], {x, 1, 28}]]
```

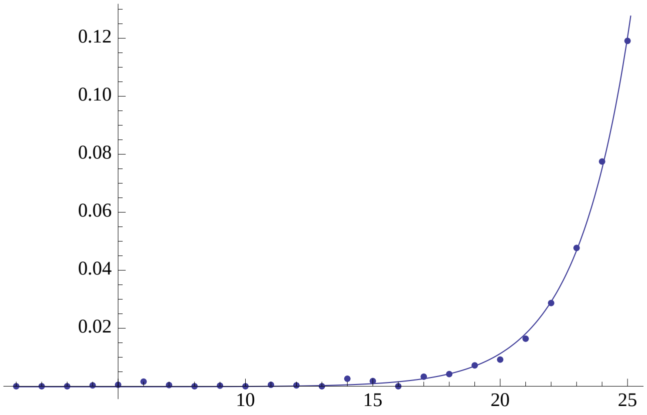


```
X431exp =
 NonlinearModelFit[X431data[[11 ;; 24]], b + d0 * a^x, {b, d0, a}, x, MaxIterations → 1000]
```

FittedModel[$-0.000400246 + 3.79123\times10^{-7} \ll19\gg^{x}$]

```
X431exp["BestFit"]
```

$-0.000400246 + 3.79123\times10^{-7}\,1.68011^{x}$

```
Show[ListPlot[X431data[[1 ;; 23]], PlotRange → All], Plot[X431exp[x], {x, 1, 26}]]
```

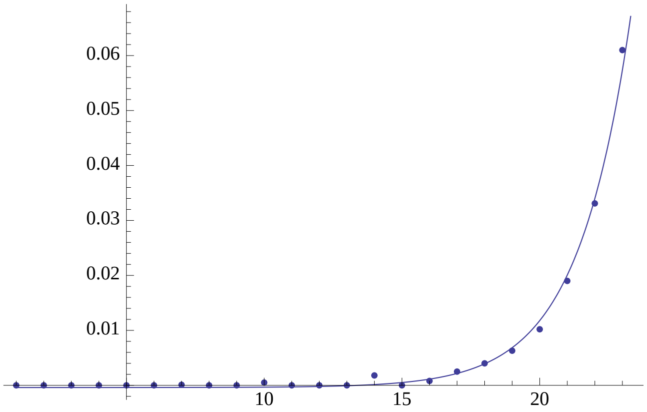


```
X432exp =
 NonlinearModelFit[X432data[[11 ;; 24]], b + d0 * a^x, {b, d0, a}, x, MaxIterations → 1000]
```

FittedModel[$-0.000351816 + 3.13799 \times 10^{-7} \ll 18 \gg^{x}$]

```
X432exp["BestFit"]
```

$-0.000351816 + 3.13799 \times 10^{-7}\, 1.69303^{x}$

```
Show[ListPlot[X432data[[1 ;; 23]], PlotRange → All], Plot[X432exp[x], {x, 1, 26}]]
```

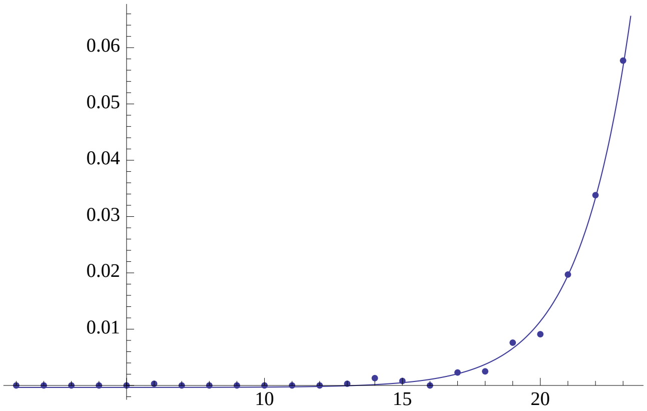


```
X433exp =
 NonlinearModelFit[X433data[[11 ;; 24]], b + d0 * a^x, {b, d0, a}, x, MaxIterations → 1000]
```

FittedModel[$-0.000280012 + 3.30429 \times 10^{-7} \ll 18 \gg^{x}$]

```
X433exp["BestFit"]
```

$-0.000280012 + 3.30429 \times 10^{-7}\, 1.68116^{x}$

```
Show[ListPlot[X433data[[1 ;; 23]], PlotRange → All], Plot[X433exp[x], {x, 1, 26}]]
```

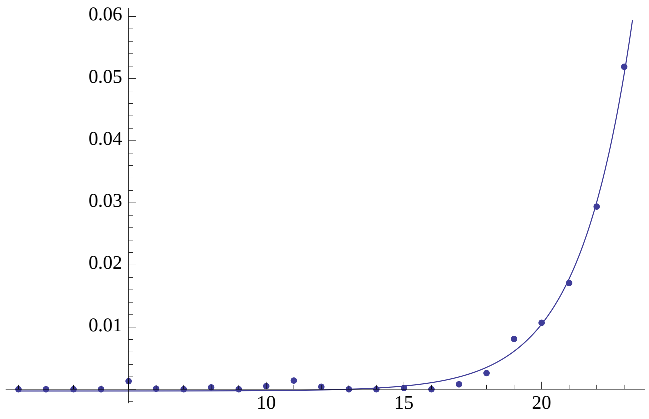


```
X434exp =
 NonlinearModelFit[X434data[[11 ;; 24]], b + d0 * a^x, {b, d0, a}, x, MaxIterations → 1000]
```

FittedModel[$-0.0000122649 + 3.10098 \times 10^{-7} \ll 19 \gg^{x}$]

```
X434exp["BestFit"]
```

$-0.0000122649 + 3.10098 \times 10^{-7}\, 1.68435^{x}$

```
Show[ListPlot[X434data[[1 ;; 23]], PlotRange → All], Plot[X434exp[x], {x, 1, 26}]]
```

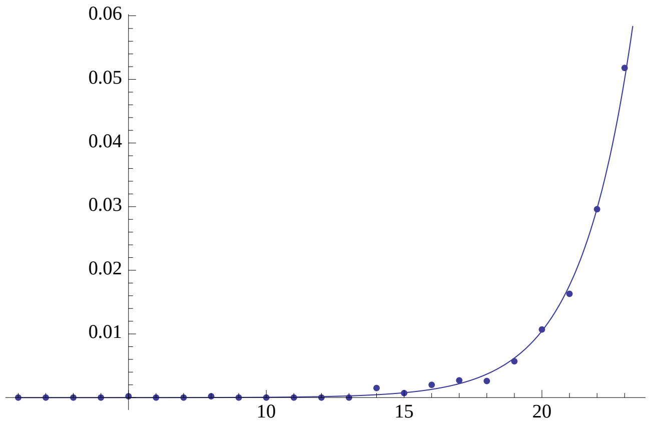


```
X441exp =
 NonlinearModelFit[X441data[[13 ;; 24]], b + d0 * a^x, {b, d0, a}, x, MaxIterations → 1000]
```

FittedModel[$-0.00030012 + 3.19644 \times 10^{-7} \ll 19 \gg^{x}$]

```
X441exp["BestFit"]
```

$-0.00030012 + 3.19644 \times 10^{-7}\, 1.66867^{x}$

```
Show[ListPlot[X441data[[1 ;; 24]], PlotRange → All], Plot[X441exp[x], {x, 1, 27}]]
```

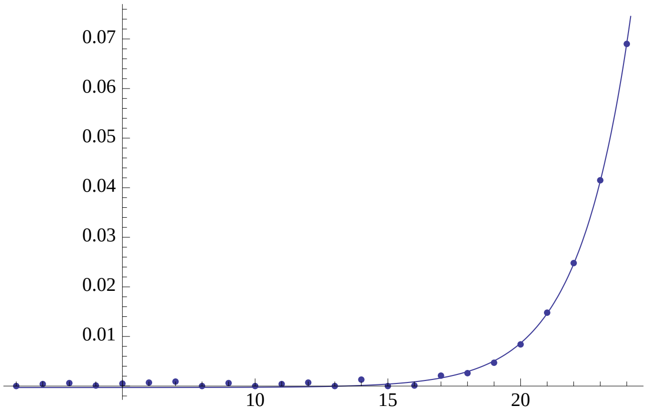


```
X442exp =
 NonlinearModelFit[X442data[[13 ;; 24]], b + d0 * a^x, {b, d0, a}, x, MaxIterations → 1000]
```

FittedModel[$-0.00028773 + 3.67806\times10^{-7}$ ≪19≫^{x}]

```
X442exp["BestFit"]
```

$-0.00028773 + 3.67806\times10^{-7}\ 1.6634^{x}$

```
Show[ListPlot[X442data[[1 ;; 23]], PlotRange → All], Plot[X442exp[x], {x, 1, 26}]]
```

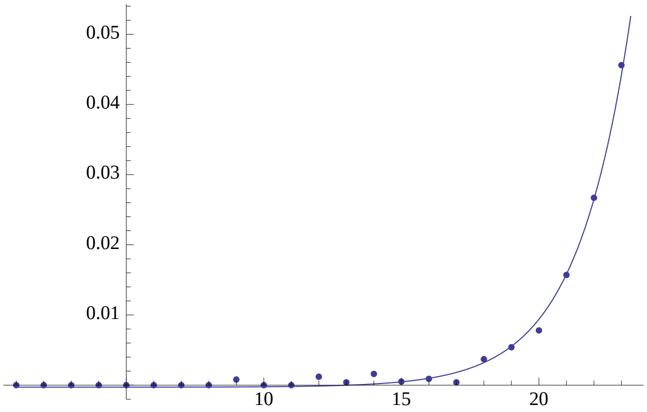


```
X443exp =
 NonlinearModelFit[X443data[[13 ;; 24]], b + d0 * a^x, {b, d0, a}, x, MaxIterations → 1000]
```

FittedModel[$0.000177021 + 2.62147\times10^{-7}$ ≪19≫^{x}]

```
X443exp["BestFit"]
```

$0.000177021 + 2.62147\times10^{-7}\ 1.67969^{x}$

```
Show[ListPlot[X443data[[1 ;; 23]], PlotRange → All], Plot[X443exp[x], {x, 1, 26}]]
```

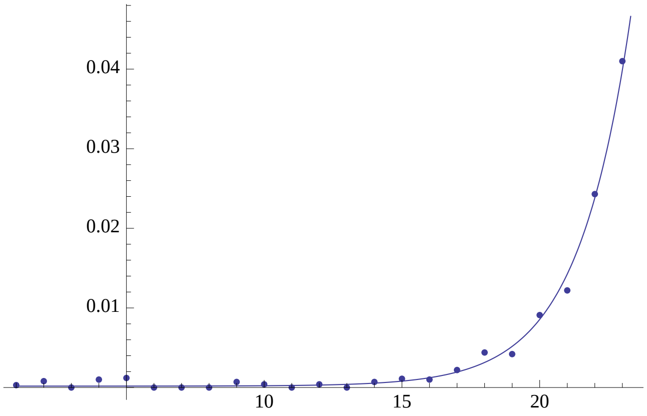


```
X444exp =
 NonlinearModelFit[X444data[[13 ;; 24]], b + d0 * a^x, {b, d0, a}, x, MaxIterations → 1000]
```

FittedModel[$-0.000290316 + 2.91505\times10^{-7}$ ≪19≫^{x}]

```
X444exp["BestFit"]
```

$-0.000290316 + 2.91505\times10^{-7}\ 1.67459^{x}$

```
Show[ListPlot[X444data[[1 ;; 24]], PlotRange → All], Plot[X444exp[x], {x, 1, 27}]]
```

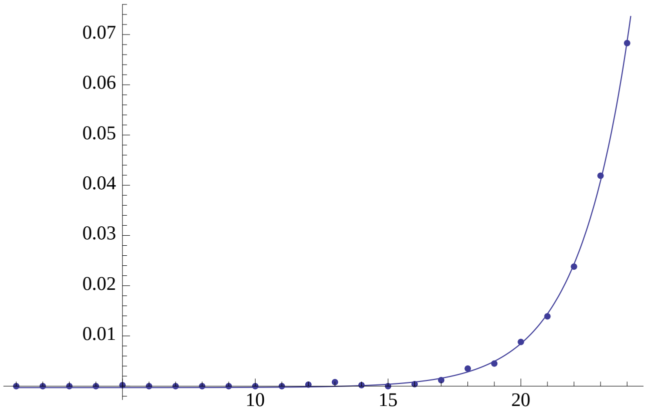


```
X451exp =
 NonlinearModelFit[X451data[[13 ;; 24]], b + d0 * a^x, {b, d0, a}, x, MaxIterations → 1000]
```

FittedModel[$-0.000971878 + 5.64705\times10^{-7}$ ≪19≫^{x}]

```
X451exp["BestFit"]
```

$-0.000971878 + 5.64705\times10^{-7}\ 1.63888^{x}$

```
Show[ListPlot[X451data[[1 ;; 24]], PlotRange → All], Plot[X451exp[x], {x, 1, 27}]]
```

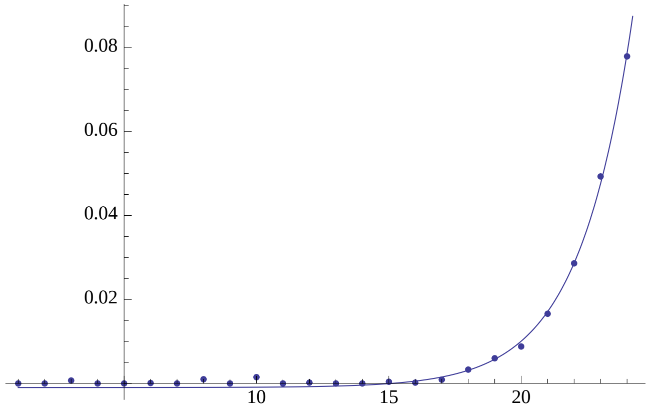


```
X452exp =
 NonlinearModelFit[X452data[[11 ;; 24]], b + d0 * a^x, {b, d0, a}, x, MaxIterations → 1000]
```

FittedModel[$-0.000454658 + 3.80166\times10^{-7}$ ≪18≫^{x}]

```
X452exp["BestFit"]
```

$-0.000454658 + 3.80166\times10^{-7}\ 1.6833^{x}$

```
Show[ListPlot[X452data[[1 ;; 24]], PlotRange → All], Plot[X452exp[x], {x, 1, 27}]]
```

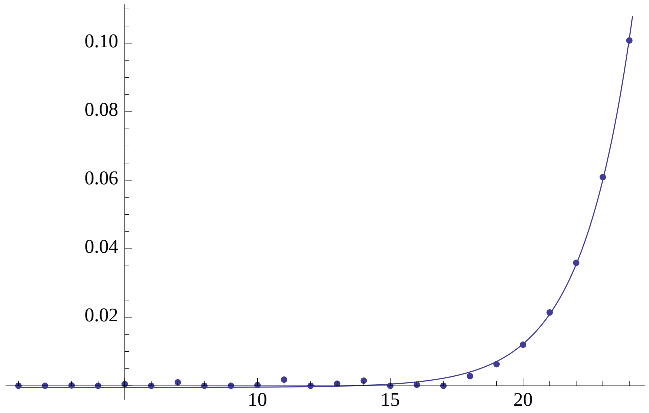


```
X453exp =
 NonlinearModelFit[X453data[[11 ;; 24]], b + d0 * a^x, {b, d0, a}, x, MaxIterations → 1000]
```

FittedModel[$-0.000238962 + 4.96792\times10^{-7}$ ≪19≫^{x}]

```
X453exp["BestFit"]
```

$-0.000238962 + 4.96792\times10^{-7}\ 1.65712^{x}$

```
Show[ListPlot[X453data[[1 ;; 24]], PlotRange → All], Plot[X453exp[x], {x, 1, 27}]]
```

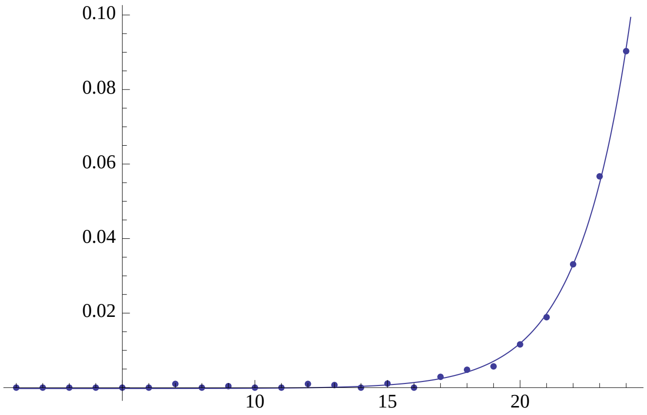


```
X454exp =
 NonlinearModelFit[X454data[[13 ;; 24]], b + d0 * a^x, {b, d0, a}, x, MaxIterations → 1000]
```

FittedModel[$-0.000516062 + 2.61187\times10^{-7}$ ≪19≫^{x}]

```
X454exp["BestFit"]
```

$-0.000516062 + 2.61187\times10^{-7}\,1.69537^{x}$

```
Show[ListPlot[X454data[[1 ;; 24]], PlotRange → All], Plot[X454exp[x], {x, 1, 27}]]
```

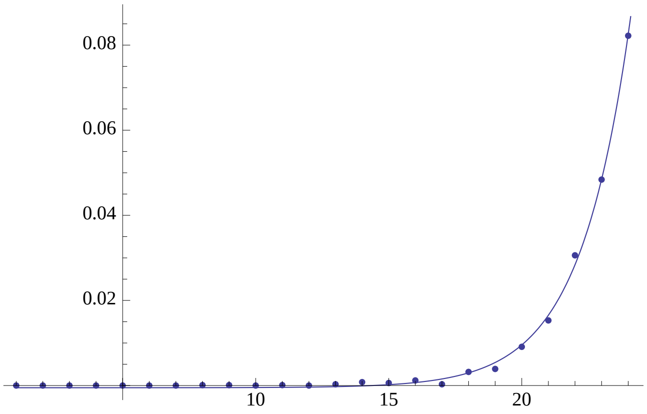


```
X511exp =
 NonlinearModelFit[X511data[[15 ;; 27]], b + d0 * a^x, {b, d0, a}, x, MaxIterations → 1000]
```

FittedModel[$-0.000738987 + 4.47959\times10^{-8}$ ≪19≫^{x}]

```
X511exp["BestFit"]
```

$-0.000738987 + 4.47959\times10^{-8}\,1.71203^{x}$

Show[ListPlot[X511data[[1 ;; 28]], PlotRange → All], Plot[X511exp[x], {x, 1, 30}]]

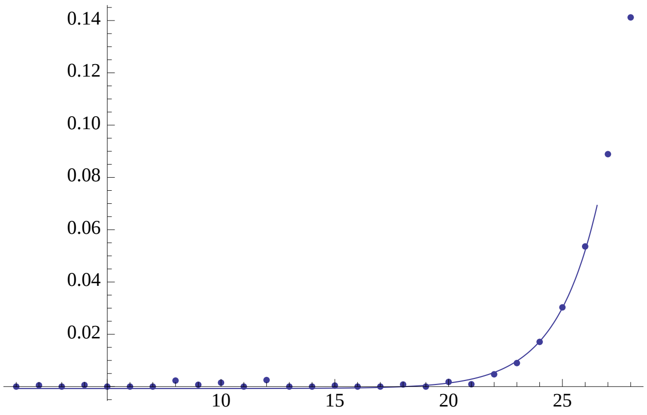

X512exp =
NonlinearModelFit[X512data[[15 ;; 27]], b + d0 * a^x, {b, d0, a}, x, MaxIterations → 1000]

FittedModel[$-0.000331286 + 2.66729\times 10^{-8} \ll 19 \gg^{x}$]

X512exp["BestFit"]

$-0.000331286 + 2.66729 \times 10^{-8}\, 1.74297^{x}$

Show[ListPlot[X512data[[1 ;; 27]], PlotRange → All], Plot[X512exp[x], {x, 1, 30}]]

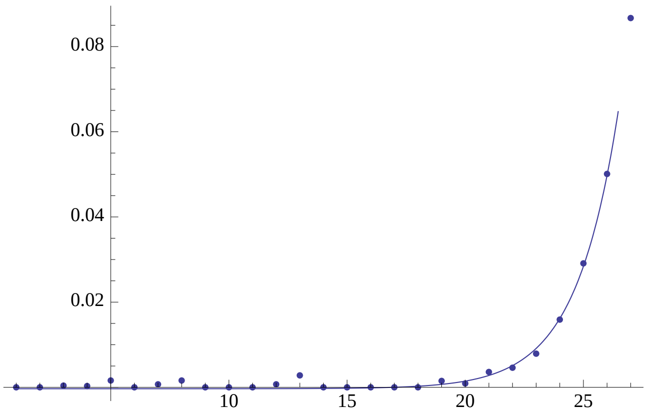

X513exp =
NonlinearModelFit[X513data[[15 ;; 27]], b + d0 * a^x, {b, d0, a}, x, MaxIterations → 1000]

FittedModel[$-0.000301367 + 3.83936\times 10^{-8} \ll 19 \gg^{x}$]

X513exp["BestFit"]

$-0.000301367 + 3.83936 \times 10^{-8}\, 1.71503^{x}$

```
Show[ListPlot[X513data[[1 ;; 27]], PlotRange → All], Plot[X513exp[x], {x, 1, 31}]]
```

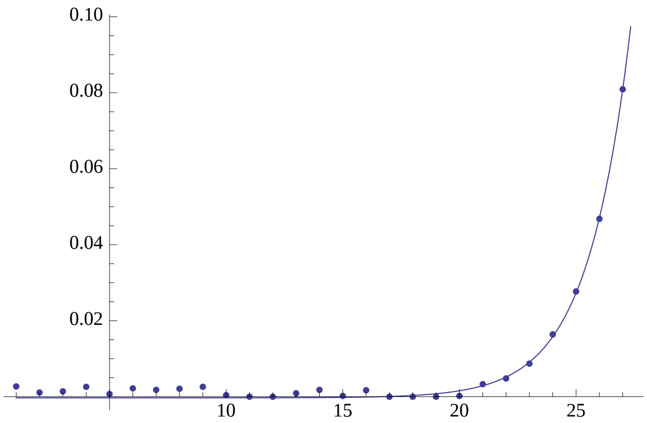


```
X514exp =
 NonlinearModelFit[X514data[[15 ;; 27]], b + d0 * a^x, {b, d0, a}, x, MaxIterations → 1000]
```

FittedModel[$-0.000209407 + 2.03618\times10^{-8} \ll 19 \gg^{x}$]

```
X514exp["BestFit"]
```

$-0.000209407 + 2.03618\times10^{-8}\ 1.74617^{x}$

```
Show[ListPlot[X514data[[1 ;; 27]], PlotRange → All], Plot[X514exp[x], {x, 1, 30}]]
```

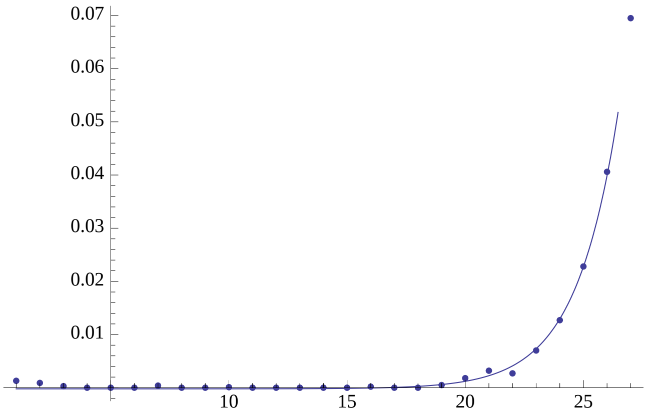


```
X521exp =
 NonlinearModelFit[X521data[[13 ;; 27]], b + d0 * a^x, {b, d0, a}, x, MaxIterations → 1000]
```

FittedModel[$-0.000285885 + 4.9818\times10^{-8} \ll 18 \gg^{x}$]

```
X521exp["BestFit"]
```

$-0.000285885 + 4.9818\times10^{-8}\ 1.69672^{x}$

```
Show[ListPlot[X521data[[1 ;; 27]], PlotRange → All], Plot[X521exp[x], {x, 1, 31}]]
```

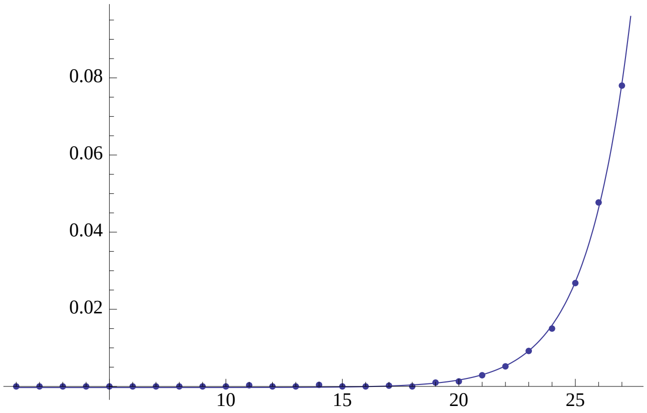


```
X522exp =
 NonlinearModelFit[X522data[[13 ;; 27]], b + d0 * a^x, {b, d0, a}, x, MaxIterations → 1000]
```

FittedModel[$-0.000318698 + 5.26792 \times 10^{-8} \ll 19 \gg^{x}$]

```
X522exp["BestFit"]
```

$-0.000318698 + 5.26792 \times 10^{-8}\, 1.6907^{x}$

```
Show[ListPlot[X522data[[1 ;; 27]], PlotRange → All], Plot[X522exp[x], {x, 1, 31}]]
```

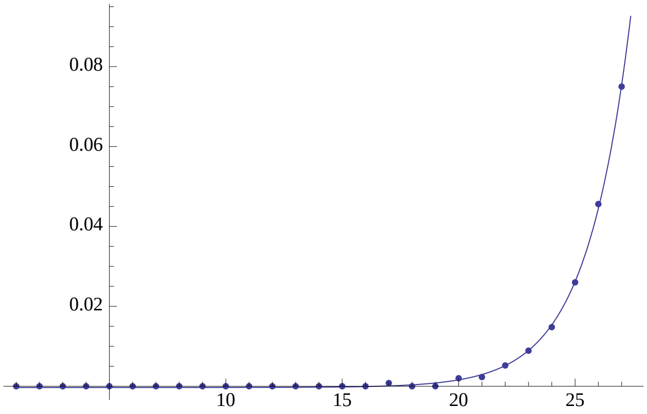


```
X523exp =
 NonlinearModelFit[X523data[[13 ;; 27]], b + d0 * a^x, {b, d0, a}, x, MaxIterations → 1000]
```

FittedModel[$-0.000354803 + 3.74318 \times 10^{-8} \ll 19 \gg^{x}$]

```
X523exp["BestFit"]
```

$-0.000354803 + 3.74318 \times 10^{-8}\, 1.716^{x}$

```
Show[ListPlot[X523data[[1 ;; 27]], PlotRange → All], Plot[X523exp[x], {x, 1, 31}]]
```

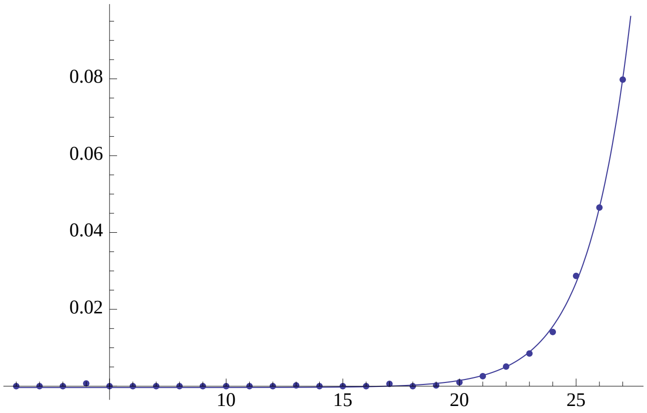


```
X524exp =
 NonlinearModelFit[X524data[[13 ;; 27]], b + d0 * a^x, {b, d0, a}, x, MaxIterations → 1000]
```

FittedModel[$-0.000200681 + 2.2863\times 10^{-8} \ll 18 \gg^{x}$]

```
X524exp["BestFit"]
```

$-0.000200681 + 2.2863\times 10^{-8}\ 1.7433^{x}$

```
Show[ListPlot[X524data[[1 ;; 27]], PlotRange → All], Plot[X524exp[x], {x, 1, 31}]]
```

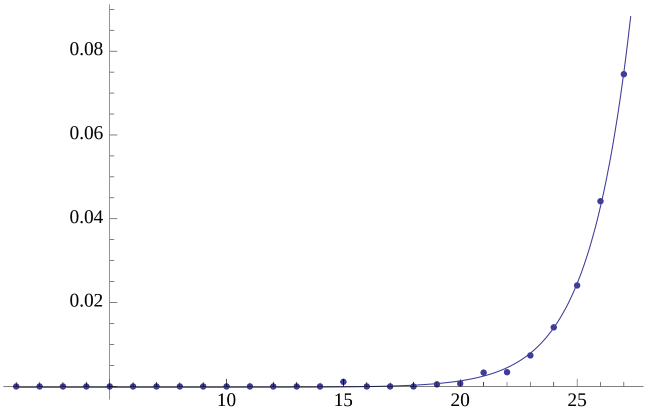


```
X531exp =
 NonlinearModelFit[X531data[[13 ;; 27]], b + d0 * a^x, {b, d0, a}, x, MaxIterations → 1000]
```

FittedModel[$-0.000509783 + 7.10334\times 10^{-8} \ll 19 \gg^{x}$]

```
X531exp["BestFit"]
```

$-0.000509783 + 7.10334\times 10^{-8}\ 1.67938^{x}$

```
Show[ListPlot[X531data[[1 ;; 27]], PlotRange → All], Plot[X531exp[x], {x, 1, 31}]]
```

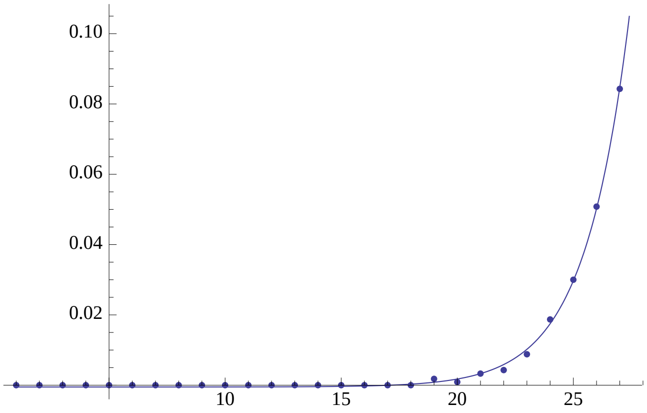


```
X532exp =
 NonlinearModelFit[X532data[[13 ;; 27]], b + d0 * a^x, {b, d0, a}, x, MaxIterations → 1000]
```

FittedModel[$-0.000262809 + 4.7395\times10^{-8} \ll 18 \gg^{x}$]

```
X532exp["BestFit"]
```

$-0.000262809 + 4.7395 \times 10^{-8}\, 1.7032^{x}$

```
Show[ListPlot[X532data[[1 ;; 27]], PlotRange → All], Plot[X532exp[x], {x, 1, 31}]]
```

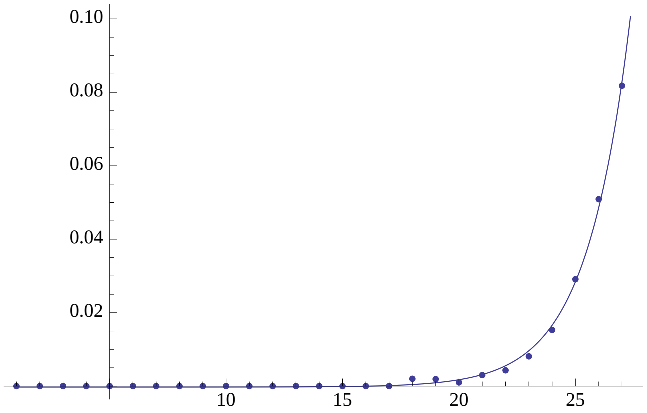


```
X533exp =
 NonlinearModelFit[X533data[[13 ;; 27]], b + d0 * a^x, {b, d0, a}, x, MaxIterations → 1000]
```

FittedModel[$-0.000370126 + 3.02444\times10^{-8} \ll 19 \gg^{x}$]

```
X533exp["BestFit"]
```

$-0.000370126 + 3.02444 \times 10^{-8}\, 1.73662^{x}$

```
Show[ListPlot[X533data[[1 ;; 27]], PlotRange → All], Plot[X533exp[x], {x, 1, 31}]]
```

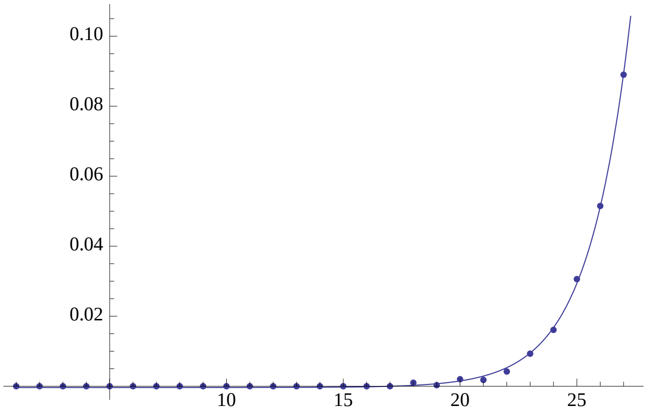


```
X534exp =
 NonlinearModelFit[X534data[[13 ;; 27]], b + d0 * a^x, {b, d0, a}, x, MaxIterations → 1000]
```

FittedModel[$-0.000524581 + 6.14339\times10^{-8}\ \ll19\gg^{x}$]

```
X534exp["BestFit"]
```

$-0.000524581 + 6.14339 \times 10^{-8}\ 1.68776^{x}$

```
Show[ListPlot[X534data[[1 ;; 27]], PlotRange → All], Plot[X534exp[x], {x, 1, 31}]]
```

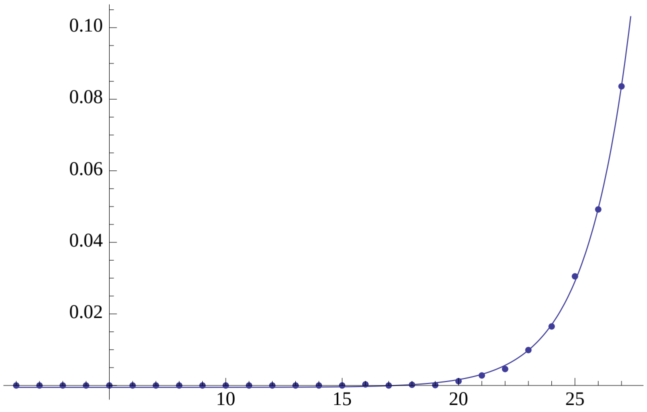


```
X541exp =
 NonlinearModelFit[X541data[[14 ;; 28]], b + d0 * a^x, {b, d0, a}, x, MaxIterations → 1000]
```

FittedModel[$-0.00113956 + 1.33382\times10^{-7}\ \ll19\gg^{x}$]

```
X541exp["BestFit"]
```

$-0.00113956 + 1.33382 \times 10^{-7}\ 1.62869^{x}$

```
Show[ListPlot[X541data[[1 ;; 28]], PlotRange → All], Plot[X541exp[x], {x, 1, 32}]]
```

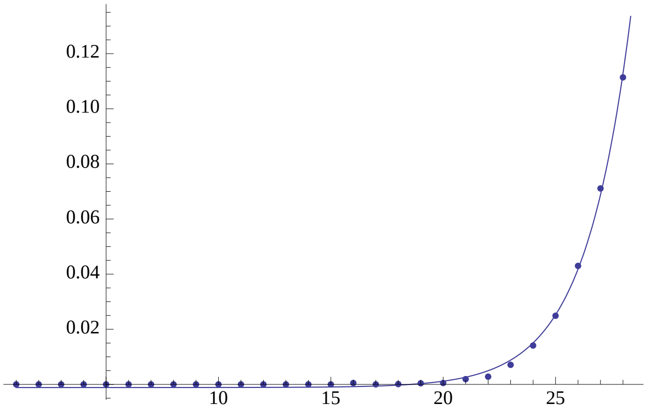


```
X542exp =
 NonlinearModelFit[X542data[[14 ;; 28]], b + d0 * a^x, {b, d0, a}, x, MaxIterations → 1000]
```

FittedModel[$-0.000649883 + 4.58214\times10^{-8} \ll 16 \gg^{x}$]

```
X542exp["BestFit"]
```

$-0.000649883 + 4.58214\times10^{-8}\, 1.68604^{x}$

```
Show[ListPlot[X542data[[1 ;; 28]], PlotRange → All], Plot[X542exp[x], {x, 1, 32}]]
```

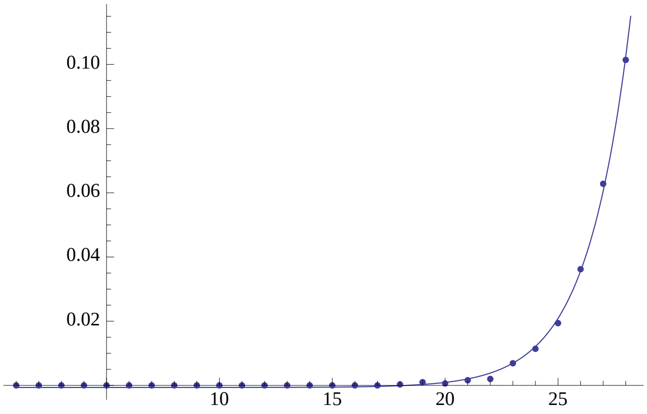


```
X543exp =
 NonlinearModelFit[X543data[[13 ;; 27]], b + d0 * a^x, {b, d0, a}, x, MaxIterations → 1000]
```

FittedModel[$-0.00040915 + 3.17519\times10^{-8} \ll 19 \gg^{x}$]

```
X543exp["BestFit"]
```

$-0.00040915 + 3.17519\times10^{-8}\, 1.72533^{x}$

```
Show[ListPlot[X543data[[1 ;; 27]], PlotRange → All], Plot[X543exp[x], {x, 1, 31}]]
```

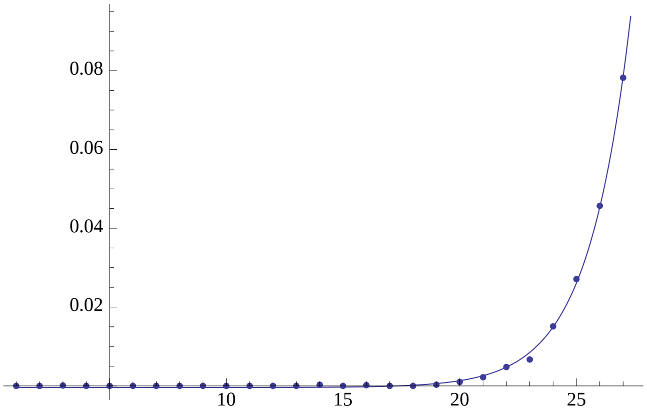


```
X544exp =
 NonlinearModelFit[X544data[[14 ;; 28]], b + d0 * a^x, {b, d0, a}, x, MaxIterations → 1000]
```

FittedModel[$-0.000889306 + 1.44809\times10^{-7}$ ≪19≫^{x}]

```
X544exp["BestFit"]
```

$-0.000889306 + 1.44809\times10^{-7}\ 1.61614^{x}$

```
Show[ListPlot[X544data[[1 ;; 28]], PlotRange → All], Plot[X544exp[x], {x, 1, 32}]]
```

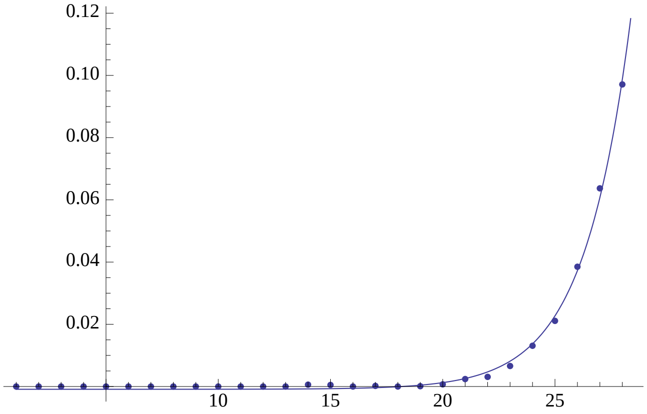


```
X551exp =
 NonlinearModelFit[X551data[[13 ;; 27]], b + d0 * a^x, {b, d0, a}, x, MaxIterations → 1000]
```

FittedModel[$-0.000287049 + 4.57546\times10^{-8}$ ≪18≫^{x}]

```
X551exp["BestFit"]
```

$-0.000287049 + 4.57546\times10^{-8}\ 1.69713^{x}$

```
Show[ListPlot[X551data[[1 ;; 27]], PlotRange → All], Plot[X551exp[x], {x, 1, 31}]]
```

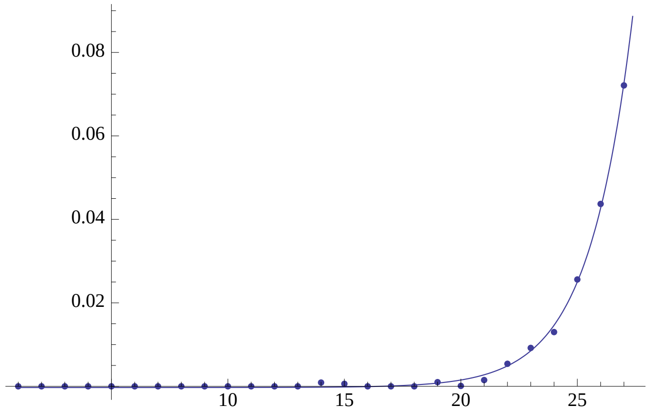


```
X552exp =
 NonlinearModelFit[X552data[[13 ;; 27]], b + d0 * a^x, {b, d0, a}, x, MaxIterations → 1000]
```

FittedModel[$-0.000357023 + 2.83702\times10^{-8} \ll 19 \gg^{x}$]

```
X552exp["BestFit"]
```

$-0.000357023 + 2.83702\times10^{-8}\, 1.72954^{x}$

```
Show[ListPlot[X552data[[1 ;; 27]], PlotRange → All], Plot[X552exp[x], {x, 1, 31}]]
```

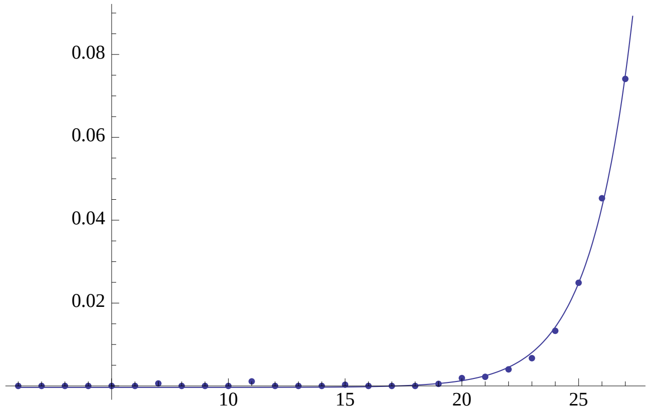


```
X553exp =
 NonlinearModelFit[X553data[[13 ;; 27]], b + d0 * a^x, {b, d0, a}, x, MaxIterations → 1000]
```

FittedModel[$-0.000262054 + 4.40371\times10^{-8} \ll 19 \gg^{x}$]

```
X553exp["BestFit"]
```

$-0.000262054 + 4.40371\times10^{-8}\, 1.70584^{x}$

```
Show[ListPlot[X553data[[1 ;; 27]], PlotRange → All], Plot[X553exp[x], {x, 1, 31}]]
```

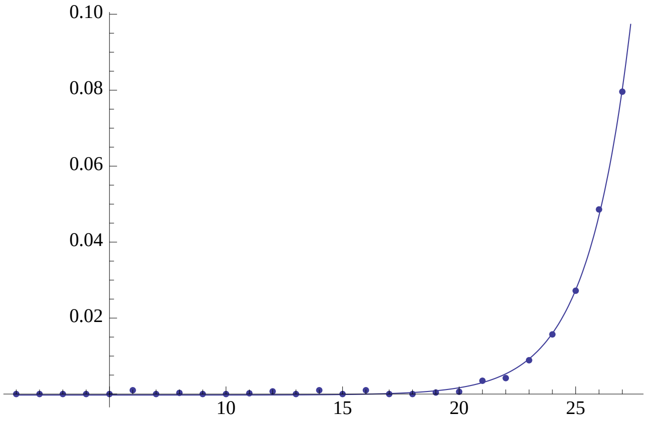


```
X554exp =
 NonlinearModelFit[X554data[[14 ;; 28]], b + d0 * a^x, {b, d0, a}, x, MaxIterations → 1000]
```

FittedModel[$-0.000684677 + 8.24391\times10^{-8}$ ≪19≫^{x}]

```
X554exp["BestFit"]
```

$-0.000684677 + 8.24391 \times 10^{-8}\, 1.65668^{x}$

```
Show[ListPlot[X554data[[1 ;; 28]], PlotRange → All], Plot[X554exp[x], {x, 1, 32}]]
```

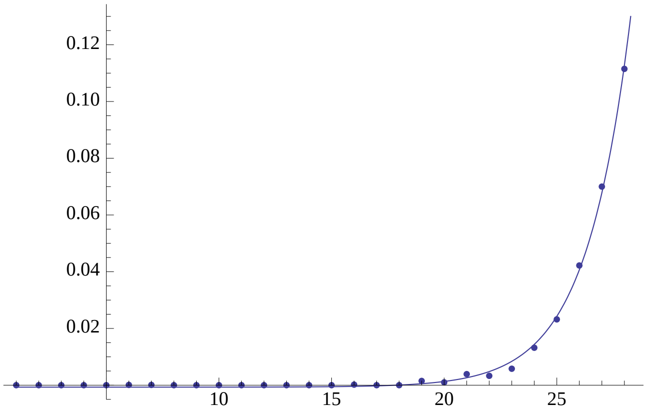


```
X611exp =
 NonlinearModelFit[X611data[[18 ;; 30]], b + d0 * a^x, {b, d0, a}, x, MaxIterations → 1000]
```

FittedModel[$-0.0000908231 + 2.98514\times10^{-9}$ ≪19≫^{x}]

```
X611exp["BestFit"]
```

$-0.0000908231 + 2.98514 \times 10^{-9}\, 1.74162^{x}$

```
Show[ListPlot[X611data[[1 ;; 30]], PlotRange → All], Plot[X611exp[x], {x, 1, 35}]]
```

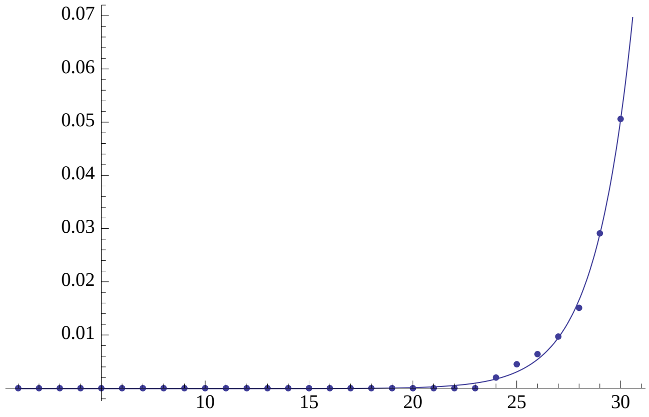


```
X612exp =
 NonlinearModelFit[X612data[[18 ;; 30]], b + d0 * a^x, {b, d0, a}, x, MaxIterations → 1000]
```

FittedModel[$-0.000518142 + 4.44221\times10^{-9}$ ≪19≫^{x}]

```
X612exp["BestFit"]
```

$-0.000518142 + 4.44221 \times 10^{-9}\ 1.72556^{x}$

```
Show[ListPlot[X612data[[1 ;; 30]], PlotRange → All], Plot[X612exp[x], {x, 1, 35}]]
```

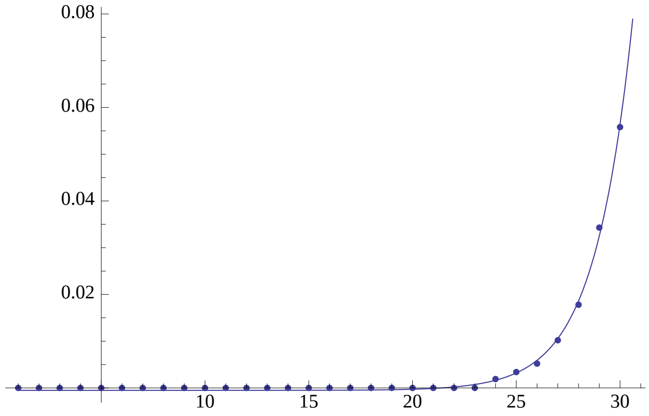


```
X613exp =
 NonlinearModelFit[X613data[[18 ;; 30]], b + d0 * a^x, {b, d0, a}, x, MaxIterations → 1000]
```

FittedModel[$-0.000404431 + 1.63179\times10^{-9}$ ≪19≫^{x}]

```
X613exp["BestFit"]
```

$-0.000404431 + 1.63179 \times 10^{-9}\ 1.78923^{x}$

```
Show[ListPlot[X613data[[1 ;; 30]], PlotRange → All], Plot[X613exp[x], {x, 1, 35}]]
```

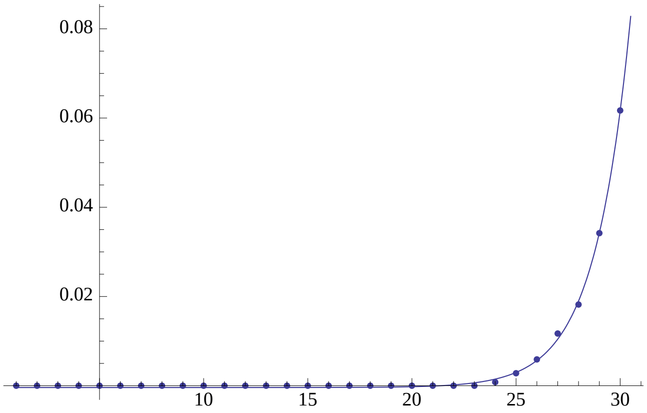


```
X614exp =
 NonlinearModelFit[X614data[[18 ;; 30]], b + d0 * a^x, {b, d0, a}, x, MaxIterations → 1000]
```

FittedModel[$-0.000431056 + 2.66141 \times 10^{-9} \ll 18 \gg^{x}$]

```
X614exp["BestFit"]
```

$-0.000431056 + 2.66141 \times 10^{-9}\, 1.76091^{x}$

```
Show[ListPlot[X614data[[1 ;; 30]], PlotRange → All], Plot[X614exp[x], {x, 1, 35}]]
```

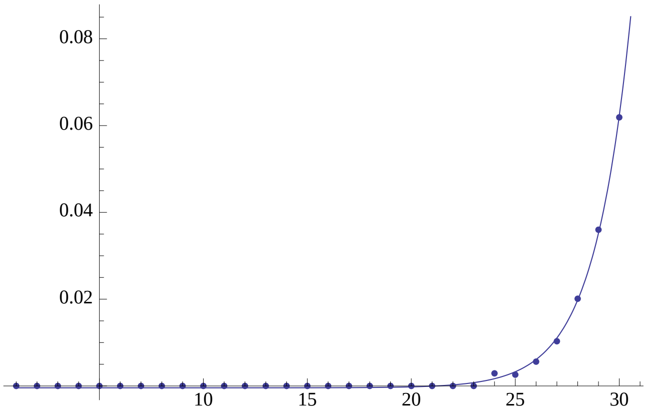


```
X621exp =
 NonlinearModelFit[X621data[[15 ;; 31]], b + d0 * a^x, {b, d0, a}, x, MaxIterations → 1000]
```

FittedModel[$-0.000225625 + 8.27961 \times 10^{-9} \ll 19 \gg^{x}$]

```
X621exp["BestFit"]
```

$-0.000225625 + 8.27961 \times 10^{-9}\, 1.68574^{x}$

```
Show[ListPlot[X621data[[1 ;; 31]], PlotRange → All], Plot[X621exp[x], {x, 1, 35}]]
```

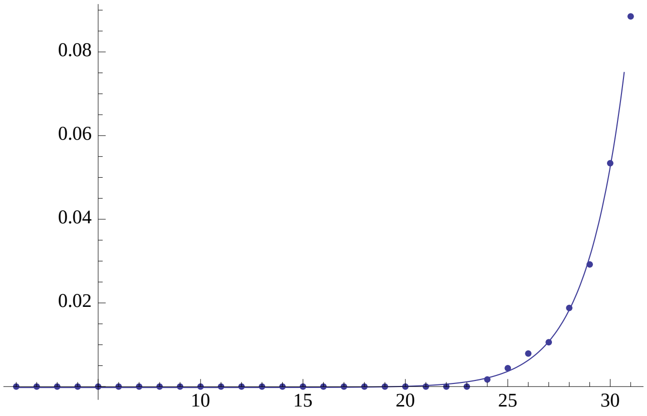


```
X622exp =
 NonlinearModelFit[X622data[[15 ;; 31]], b + d0 * a^x, {b, d0, a}, x, MaxIterations → 1000]
```

FittedModel[$-0.000538851 + 8.27626\times10^{-9}$ ≪19≫^{x}]

```
X622exp["BestFit"]
```

$-0.000538851 + 8.27626\times10^{-9}\ 1.68927^{x}$

```
Show[ListPlot[X622data[[1 ;; 31]], PlotRange → All], Plot[X622exp[x], {x, 1, 35}]]
```

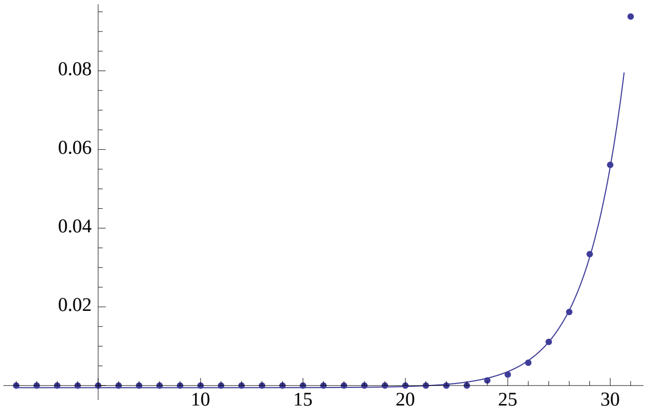


```
X623exp =
 NonlinearModelFit[X623data[[15 ;; 31]], b + d0 * a^x, {b, d0, a}, x, MaxIterations → 1000]
```

FittedModel[$-0.000440008 + 8.04808\times10^{-9}$ ≪19≫^{x}]

```
X623exp["BestFit"]
```

$-0.000440008 + 8.04808\times10^{-9}\ 1.69071^{x}$

```
Show[ListPlot[X623data[[1 ;; 31]], PlotRange → All], Plot[X623exp[x], {x, 1, 35}]]
```

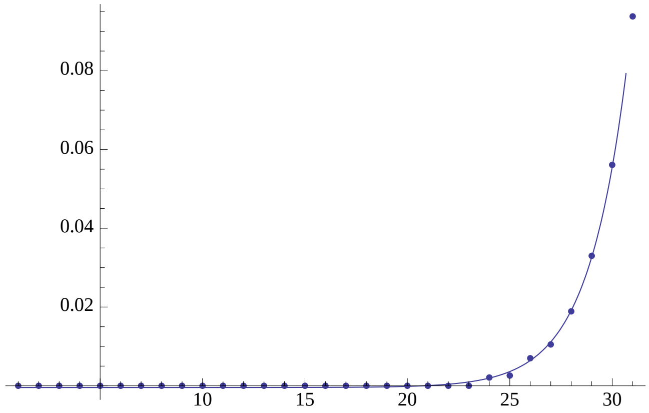


```
X624exp =
 NonlinearModelFit[X624data[[15 ;; 31]], b + d0 * a^x, {b, d0, a}, x, MaxIterations → 1000]
```

FittedModel[$-0.00061082 + 1.01173\times10^{-8} \ll 19\gg^{x}$]

```
X624exp["BestFit"]
```

$-0.00061082 + 1.01173\times10^{-8}\ 1.67564^{x}$

```
Show[ListPlot[X624data[[1 ;; 31]], PlotRange → All], Plot[X624exp[x], {x, 1, 35}]]
```

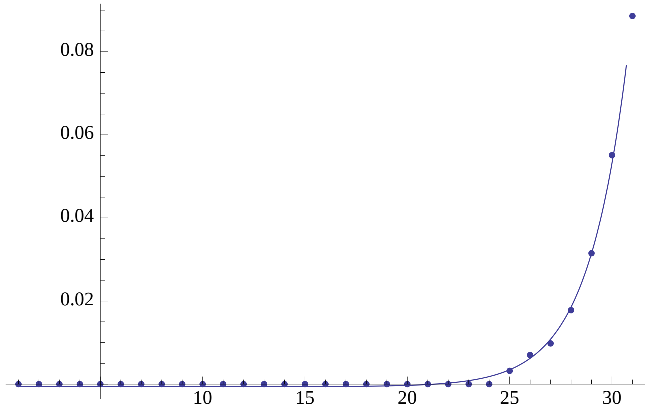


```
X631exp =
 NonlinearModelFit[X631data[[15 ;; 31]], b + d0 * a^x, {b, d0, a}, x, MaxIterations → 1000]
```

FittedModel[$-0.000361452 + 8.02935\times10^{-9} \ll 18\gg^{x}$]

```
X631exp["BestFit"]
```

$-0.000361452 + 8.02935\times10^{-9}\ 1.69356^{x}$

```
Show[ListPlot[X631data[[1 ;; 31]], PlotRange → All], Plot[X631exp[x], {x, 1, 35}]]
```

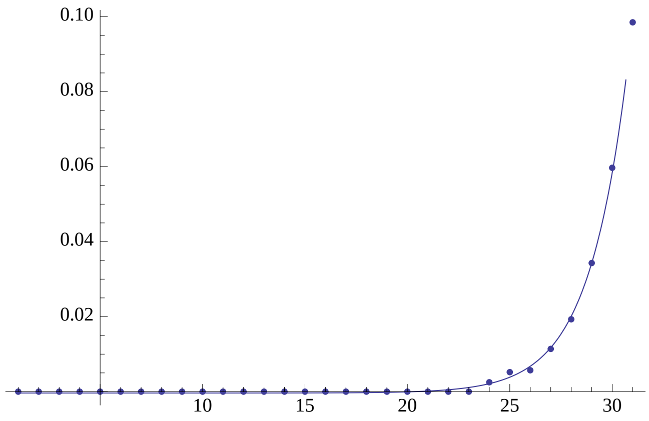


```
X632exp =
 NonlinearModelFit[X632data[[15 ;; 31]], b + d0 * a^x, {b, d0, a}, x, MaxIterations → 1000]
```

FittedModel[$-0.000649099 + 7.51224\times10^{-9} \ll19\gg^{x}$]

```
X632exp["BestFit"]
```

$-0.000649099 + 7.51224 \times 10^{-9}\, 1.6991^{x}$

```
Show[ListPlot[X632data[[1 ;; 31]], PlotRange → All], Plot[X632exp[x], {x, 1, 35}]]
```

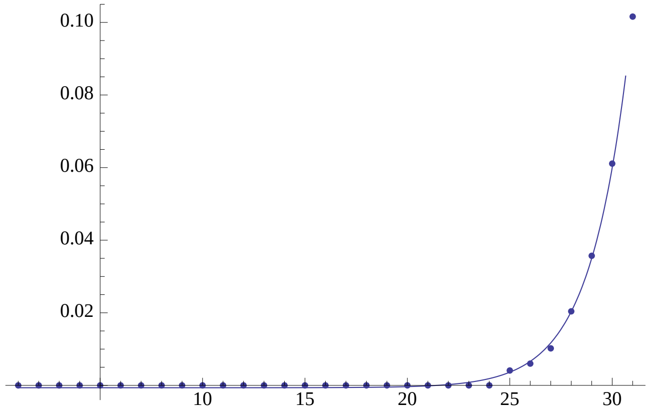


```
X633exp =
 NonlinearModelFit[X633data[[15 ;; 31]], b + d0 * a^x, {b, d0, a}, x, MaxIterations → 1000]
```

FittedModel[$-0.00073083 + 1.83628\times10^{-8} \ll19\gg^{x}$]

```
X633exp["BestFit"]
```

$-0.00073083 + 1.83628 \times 10^{-8}\, 1.6502^{x}$

```
Show[ListPlot[X633data[[1 ;; 31]], PlotRange → All], Plot[X633exp[x], {x, 1, 35}]]
```

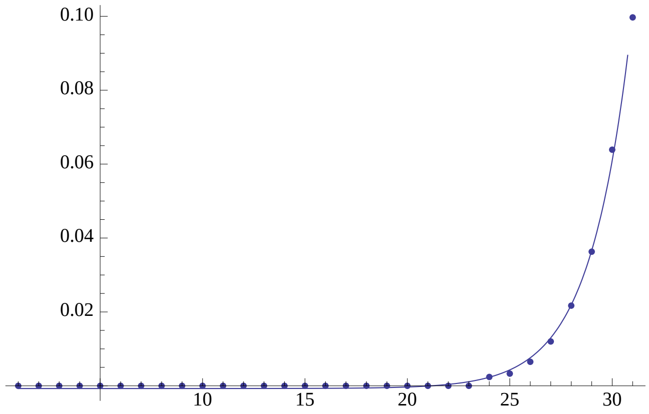


```
X634exp =
 NonlinearModelFit[X634data[[15 ;; 31]], b + d0 * a^x, {b, d0, a}, x, MaxIterations → 1000]
```

FittedModel[$-0.000689452 + 9.46028\times10^{-9} \ll 17 \gg^{x}$]

```
X634exp["BestFit"]
```

$-0.000689452 + 9.46028\times10^{-9}\ 1.68271^{x}$

```
Show[ListPlot[X634data[[1 ;; 31]], PlotRange → All], Plot[X634exp[x], {x, 1, 35}]]
```

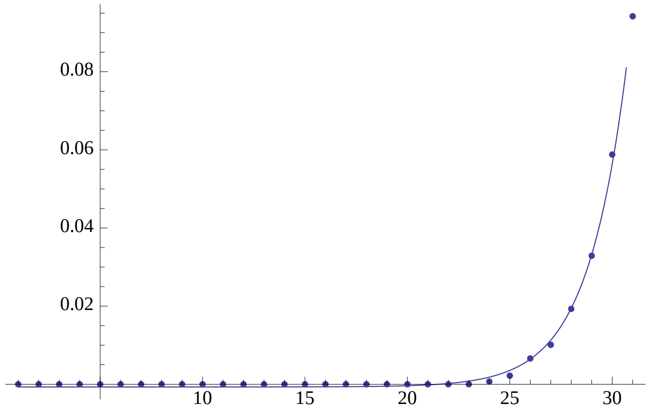


```
X641exp =
 NonlinearModelFit[X641data[[15 ;; 31]], b + d0 * a^x, {b, d0, a}, x, MaxIterations → 1000]
```

FittedModel[$-0.000648255 + 7.73739\times10^{-9} \ll 19 \gg^{x}$]

```
X641exp["BestFit"]
```

$-0.000648255 + 7.73739\times10^{-9}\ 1.68535^{x}$

```
Show[ListPlot[X641data[[1 ;; 31]], PlotRange → All], Plot[X641exp[x], {x, 1, 35}]]
```

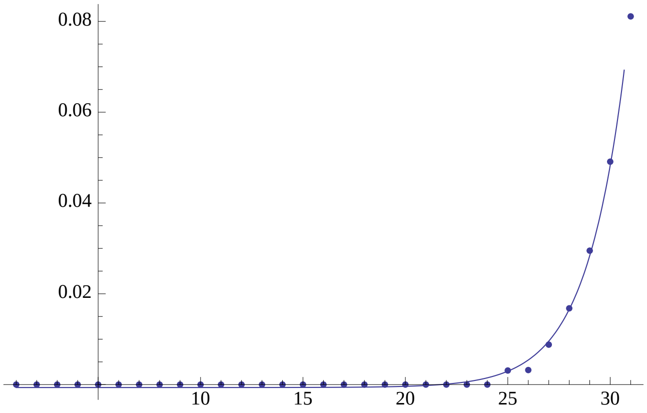


```
X642exp =
 NonlinearModelFit[X642data[[15 ;; 31]], b + d0 * a^x, {b, d0, a}, x, MaxIterations → 1000]
```

FittedModel[$-0.000661835 + 1.13516\times10^{-8}\ \ll19\gg^{x}$]

```
X642exp["BestFit"]
```

$-0.000661835 + 1.13516\times10^{-8}\ 1.65859^{x}$

```
Show[ListPlot[X642data[[1 ;; 31]], PlotRange → All], Plot[X642exp[x], {x, 1, 35}]]
```

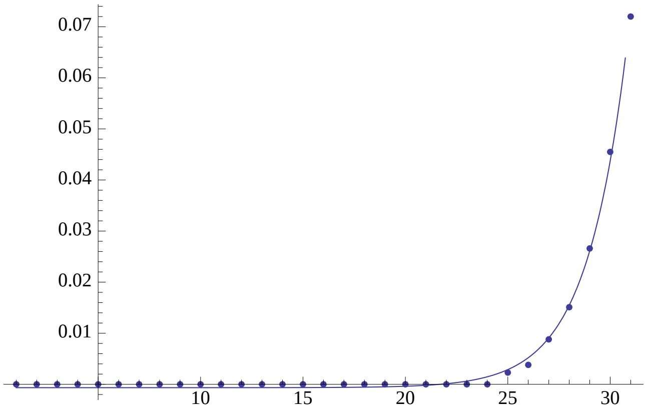


```
X643exp =
 NonlinearModelFit[X643data[[15 ;; 32]], b + d0 * a^x, {b, d0, a}, x, MaxIterations → 1000]
```

FittedModel[$-0.000810245 + 3.94578\times10^{-8}\ \ll19\gg^{x}$]

```
X643exp["BestFit"]
```

$-0.000810245 + 3.94578\times10^{-8}\ 1.58553^{x}$

```
Show[ListPlot[X643data[[1 ;; 32]], PlotRange → All], Plot[X643exp[x], {x, 1, 37}]]
```

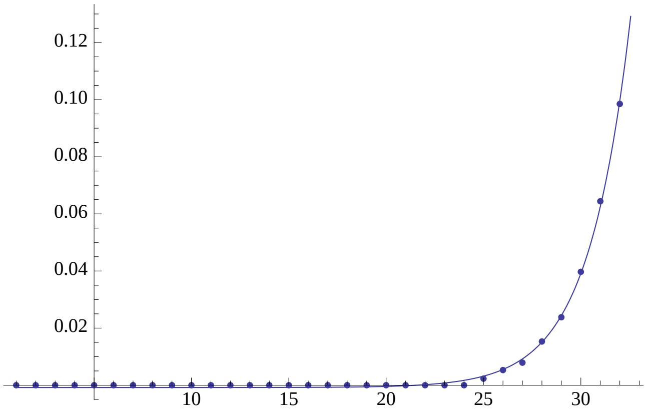


```
X644exp =
 NonlinearModelFit[X644data[[15 ;; 32]], b + d0 * a^x, {b, d0, a}, x, MaxIterations → 1000]
```

FittedModel[$-0.00102383 + 2.40278 \times 10^{-8} \ll 18 \gg^{x}$]

```
X644exp["BestFit"]
```

$-0.00102383 + 2.40278 \times 10^{-8}\, 1.61025^{x}$

```
Show[ListPlot[X644data[[1 ;; 32]], PlotRange → All], Plot[X644exp[x], {x, 1, 37}]]
```

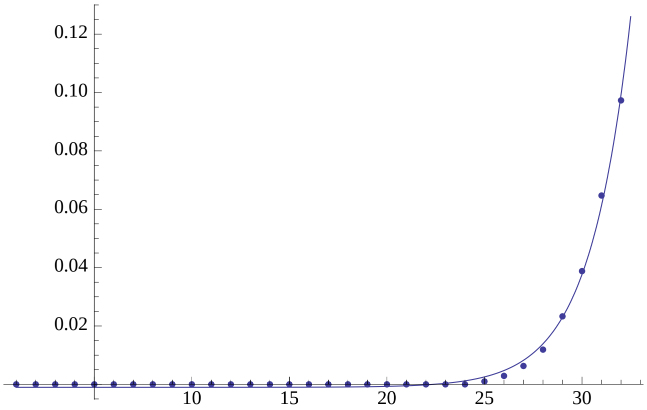


```
X651exp =
 NonlinearModelFit[X651data[[15 ;; 31]], b + d0 * a^x, {b, d0, a}, x, MaxIterations → 1000]
```

FittedModel[$-0.000700728 + 1.03121 \times 10^{-8} \ll 19 \gg^{x}$]

```
X651exp["BestFit"]
```

$-0.000700728 + 1.03121 \times 10^{-8}\, 1.67175^{x}$

```
Show[ListPlot[X651data[[1 ;; 31]], PlotRange → All], Plot[X651exp[x], {x, 1, 37}]]
```

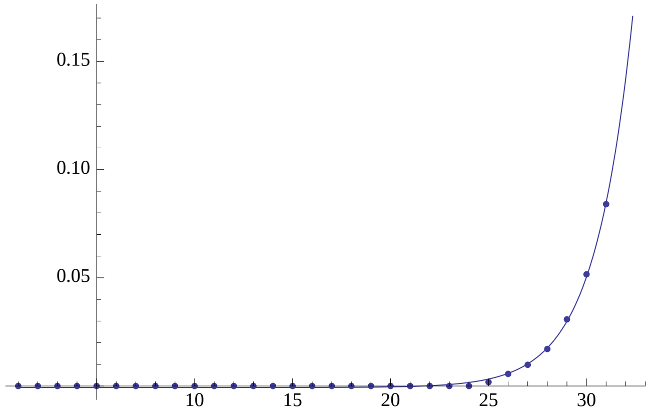


```
X652exp =
 NonlinearModelFit[X652data[[15 ;; 31]], b + d0 * a^x, {b, d0, a}, x, MaxIterations → 1000]
```

FittedModel[$-0.00067847 + 8.8\times10^{-9}$ ≪19≫^{x}]

```
X652exp["BestFit"]
```

$-0.00067847 + 8.8\times10^{-9}\,1.68613^{x}$

```
Show[ListPlot[X652data[[1 ;; 31]], PlotRange → All], Plot[X652exp[x], {x, 1, 36}]]
```

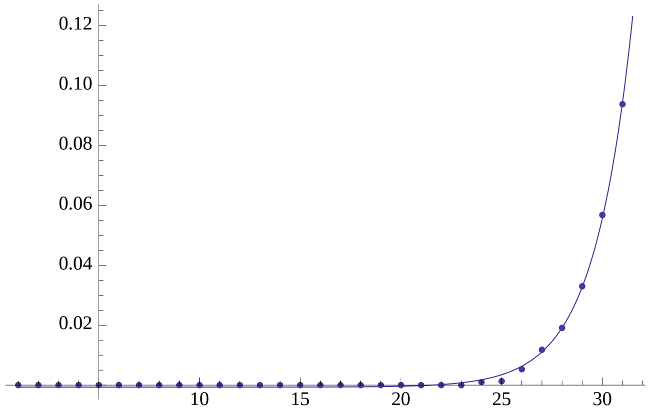


```
X653exp =
 NonlinearModelFit[X653data[[15 ;; 31]], b + d0 * a^x, {b, d0, a}, x, MaxIterations → 1000]
```

FittedModel[$-0.000706141 + 9.94051\times10^{-9}$ ≪19≫^{x}]

```
X653exp["BestFit"]
```

$-0.000706141 + 9.94051\times10^{-9}\,1.67423^{x}$

```
Show[ListPlot[X653data[[1 ;; 31]], PlotRange → All], Plot[X653exp[x], {x, 1, 36}]]
```

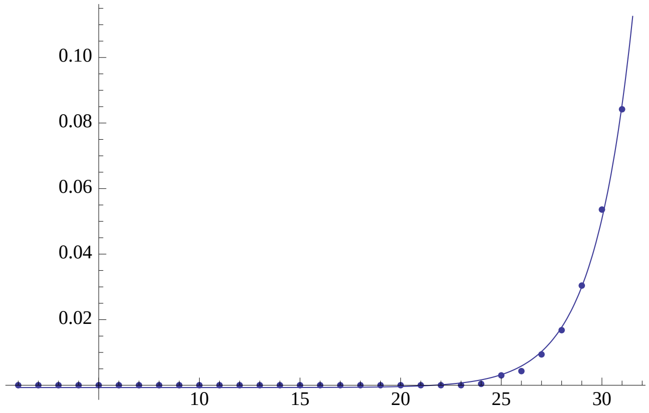


```
X654exp =
 NonlinearModelFit[X654data[[15 ;; 31]], b + d0 * a^x, {b, d0, a}, x, MaxIterations → 1000]
```

FittedModel[$-0.000464563 + 6.94442\times10^{-9} \ll19\gg^{x}$]

```
X654exp["BestFit"]
```

$-0.000464563 + 6.94442\times10^{-9}\ 1.6828^{x}$

```
Show[ListPlot[X654data[[1 ;; 31]], PlotRange → All], Plot[X654exp[x], {x, 1, 36}]]
```

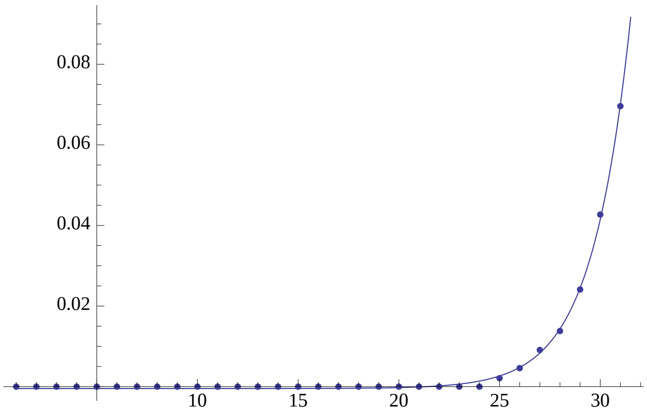


```
indata = Import["reps.csv"];
cycle = indata[[All, 1]][[2 ;;]];

F11data = Transpose[{cycle, indata[[All, 3]][[2 ;;]]}];
F12data = Transpose[{cycle, indata[[All, 4]][[2 ;;]]}];
F13data = Transpose[{cycle, indata[[All, 5]][[2 ;;]]}];
F14data = Transpose[{cycle, indata[[All, 6]][[2 ;;]]}];
F21data = Transpose[{cycle, indata[[All, 7]][[2 ;;]]}];
F22data = Transpose[{cycle, indata[[All, 8]][[2 ;;]]}];
F23data = Transpose[{cycle, indata[[All, 9]][[2 ;;]]}];
F24data = Transpose[{cycle, indata[[All, 10]][[2 ;;]]}];
F31data = Transpose[{cycle, indata[[All, 11]][[2 ;;]]}];
F32data = Transpose[{cycle, indata[[All, 12]][[2 ;;]]}];
F33data = Transpose[{cycle, indata[[All, 13]][[2 ;;]]}];
F34data = Transpose[{cycle, indata[[All, 14]][[2 ;;]]}];
F41data = Transpose[{cycle, indata[[All, 15]][[2 ;;]]}];
F42data = Transpose[{cycle, indata[[All, 16]][[2 ;;]]}];
F43data = Transpose[{cycle, indata[[All, 17]][[2 ;;]]}];
F44data = Transpose[{cycle, indata[[All, 18]][[2 ;;]]}];
F51data = Transpose[{cycle, indata[[All, 19]][[2 ;;]]}];
F52data = Transpose[{cycle, indata[[All, 20]][[2 ;;]]}];
F53data = Transpose[{cycle, indata[[All, 21]][[2 ;;]]}];
F54data = Transpose[{cycle, indata[[All, 22]][[2 ;;]]}];
F61data = Transpose[{cycle, indata[[All, 23]][[2 ;;]]}];
F62data = Transpose[{cycle, indata[[All, 24]][[2 ;;]]}];
F63data = Transpose[{cycle, indata[[All, 25]][[2 ;;]]}];
F64data = Transpose[{cycle, indata[[All, 26]][[2 ;;]]}];
F71data = Transpose[{cycle, indata[[All, 27]][[2 ;;]]}];
F72data = Transpose[{cycle, indata[[All, 28]][[2 ;;]]}];
F73data = Transpose[{cycle, indata[[All, 29]][[2 ;;]]}];
F74data = Transpose[{cycle, indata[[All, 30]][[2 ;;]]}];

F11exp = NonlinearModelFit[F11data[[1 ;; 15]], b + d0 * a^x, {b, d0, a}, x, MaxIterations → 1000]

FittedModel[ 0.00100241 + 0.00036582 ≪19≫^x ]
```

```
F11exp["BestFit"]

0.00100241 + 0.00036582 1.74643^x

Show[ListPlot[F11data[[1 ;; 15]], PlotRange → All], Plot[F11exp[x], {x, 1, 17}]]
```

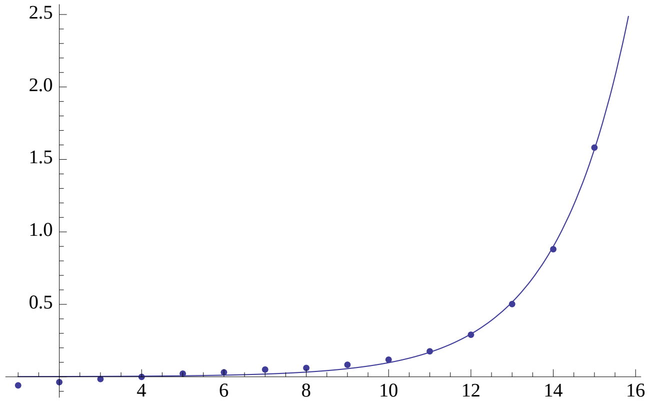


```
F12exp = NonlinearModelFit[F12data[[1 ;; 15]], b + d0 * a^x, {b, d0, a}, x, MaxIterations → 1000]

FittedModel[ 0.00134432 + 0.000404047 ≪19≫^x ]

F12exp["BestFit"]

0.00134432 + 0.000404047 1.75124^x

Show[ListPlot[F12data[[1 ;; 15]], PlotRange → All], Plot[F12exp[x], {x, 1, 17}]]
```

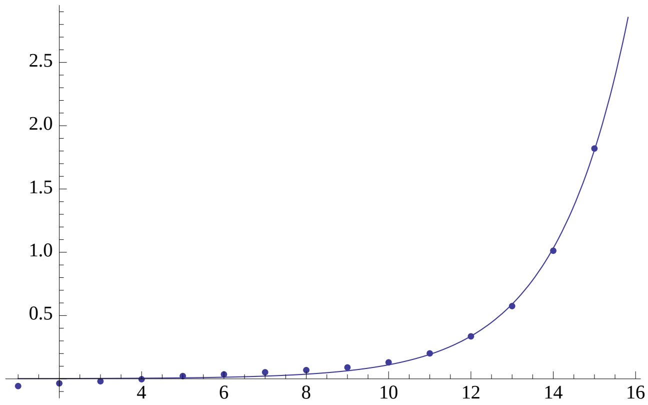


```
F13exp = NonlinearModelFit[F13data[[1 ;; 15]], b + d0 * a^x, {b, d0, a}, x, MaxIterations → 1000]

FittedModel[ 0.023234 + 0.00101156 ≪19≫^x ]

F13exp["BestFit"]

0.023234 + 0.00101156 1.63668^x
```

```
Show[ListPlot[F13data[[1 ;; 15]], PlotRange → All], Plot[F13exp[x], {x, 1, 17}]]
```

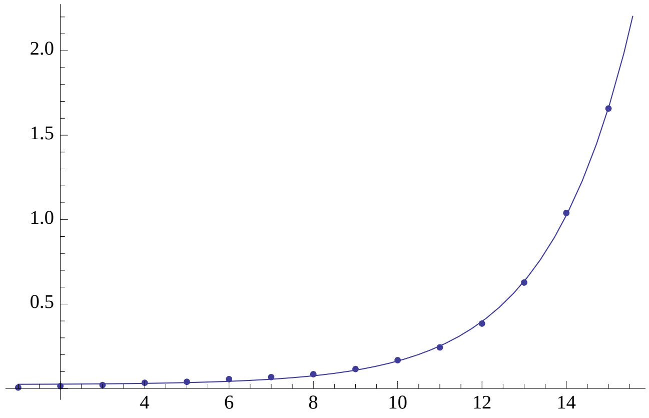


```
F14exp = NonlinearModelFit[F14data[[1 ;; 15]], b + d0 * a^x, {b, d0, a}, x, MaxIterations → 1000]
```

FittedModel[$0.00938176 + 0.000466019 \ll 19 \gg^{x}$]

```
F14exp["BestFit"]
```

$0.00938176 + 0.000466019\ 1.69869^{x}$

```
Show[ListPlot[F14data[[1 ;; 15]], PlotRange → All], Plot[F14exp[x], {x, 1, 17}]]
```

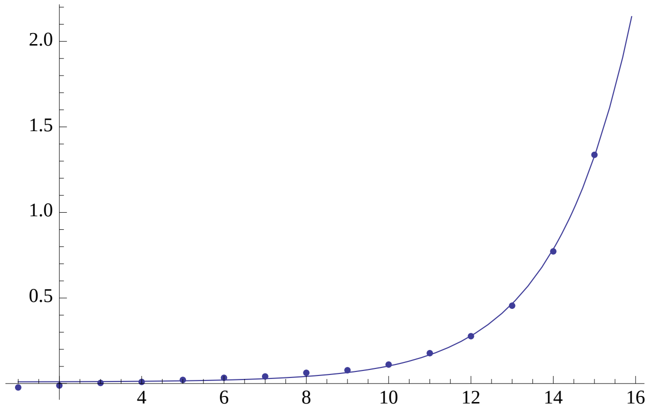


```
F21exp = NonlinearModelFit[F21data[[1 ;; 18]], b + d0 * a^x, {b, d0, a}, x, MaxIterations → 1000]
```

FittedModel[$0.0116202 + 0.0000396458 \ll 15 \gg^{x}$]

```
F21exp["BestFit"]
```

$0.0116202 + 0.0000396458\ 1.77338^{x}$

```
Show[ListPlot[F21data[[1 ;; 18]], PlotRange → All], Plot[F21exp[x], {x, 1, 20}]]
```

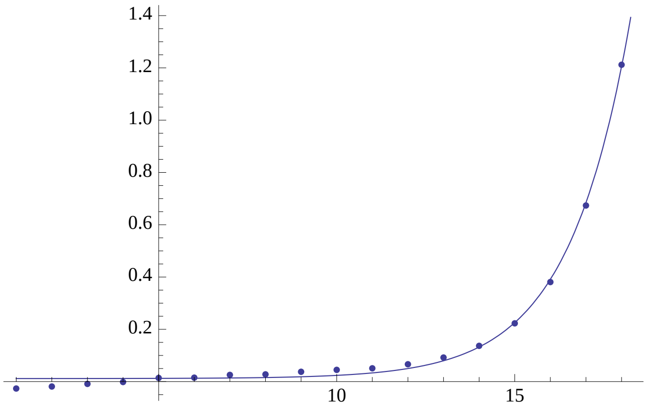


```
F22exp = NonlinearModelFit[F22data[[1 ;; 18]], b + d0 * a^x, {b, d0, a}, x, MaxIterations → 1000]
```

FittedModel[$0.0115172 + 0.0000431857 \ll 19 \gg^{x}$]

```
F22exp["BestFit"]
```

$0.0115172 + 0.0000431857\ 1.78057^{x}$

```
Show[ListPlot[F22data[[1 ;; 18]], PlotRange → All], Plot[F22exp[x], {x, 1, 20}]]
```

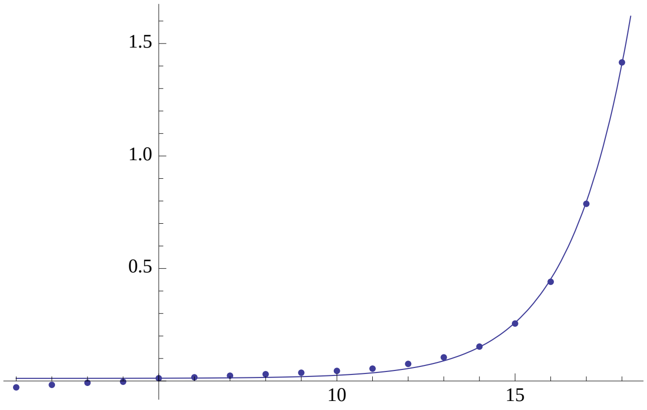


```
F23exp = NonlinearModelFit[F23data[[1 ;; 18]], b + d0 * a^x, {b, d0, a}, x, MaxIterations → 1000]
```

FittedModel[$0.0251573 + 0.00020661 \ll 19 \gg^{x}$]

```
F23exp["BestFit"]
```

$0.0251573 + 0.00020661\ 1.64007^{x}$

```
Show[ListPlot[F23data[[1 ;; 18]], PlotRange → All], Plot[F23exp[x], {x, 1, 20}]]
```

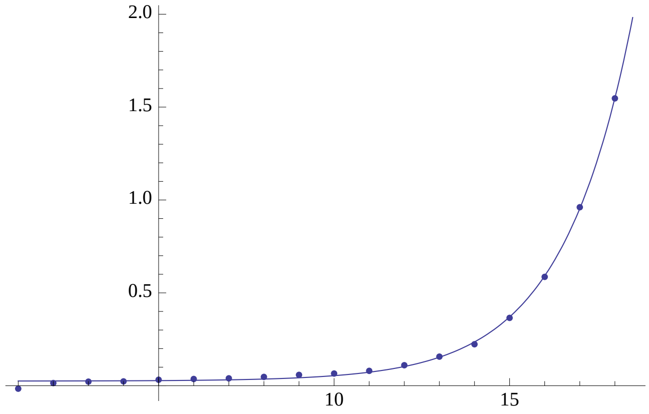


```
F24exp = NonlinearModelFit[F24data[[1 ;; 18]], b + d0 * a^x, {b, d0, a}, x, MaxIterations → 1000]
```

FittedModel[$0.0187197 + 0.000219314 \ll 18 \gg^{x}$]

```
F24exp["BestFit"]
```

$0.0187197 + 0.000219314\ 1.67487^{x}$

```
Show[ListPlot[F24data[[1 ;; 18]], PlotRange → All], Plot[F24exp[x], {x, 1, 20}]]
```

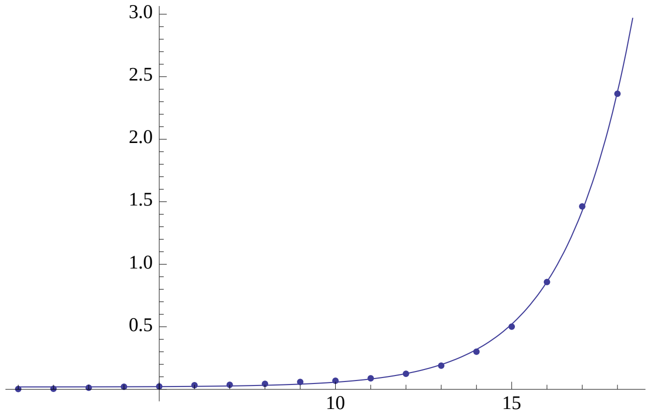


```
F31exp = NonlinearModelFit[F31data[[1 ;; 22]], b + d0 * a^x, {b, d0, a}, x, MaxIterations → 1000]
```

FittedModel[$0.0189188 + 4.87297 \times 10^{-6} \ll 19 \gg^{x}$]

```
F31exp["BestFit"]
```

$0.0189188 + 4.87297 \times 10^{-6}\ 1.79821^{x}$

```
Show[ListPlot[F31data[[1 ;; 22]], PlotRange → All], Plot[F31exp[x], {x, 1, 25}]]
```

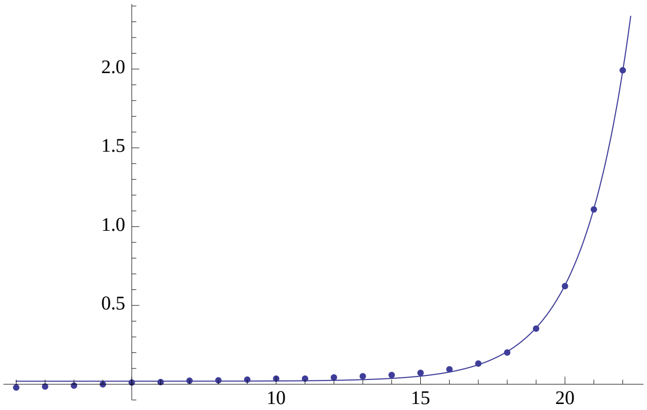


```
F32exp = NonlinearModelFit[F32data[[1 ;; 22]], b + d0 * a^x, {b, d0, a}, x, MaxIterations → 1000]
```

FittedModel[$0.0159663 + 5.17298 \times 10^{-6} \ll 19 \gg^{x}$]

```
F32exp["BestFit"]
```

$0.0159663 + 5.17298 \times 10^{-6}\, 1.80446^{x}$

```
Show[ListPlot[F32data[[1 ;; 22]], PlotRange → All], Plot[F32exp[x], {x, 1, 25}]]
```

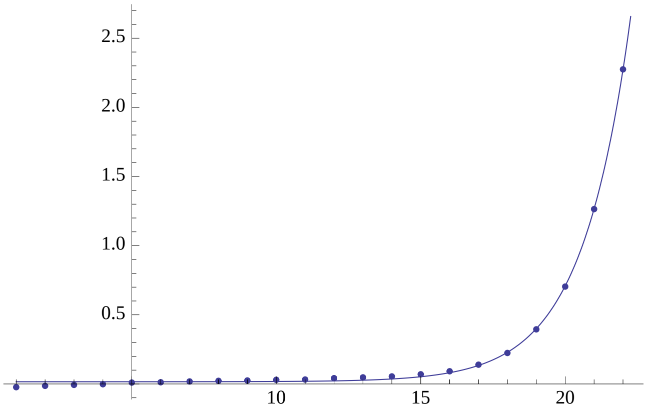


```
F33exp = NonlinearModelFit[F33data[[1 ;; 22]], b + d0 * a^x, {b, d0, a}, x, MaxIterations → 1000]
```

FittedModel[$0.0310752 + 0.0000869497 \ll 19 \gg^{x}$]

```
F33exp["BestFit"]
```

$0.0310752 + 0.0000869497\, 1.59358^{x}$

```
Show[ListPlot[F33data[[1 ;; 22]], PlotRange → All], Plot[F33exp[x], {x, 1, 25}]]
```

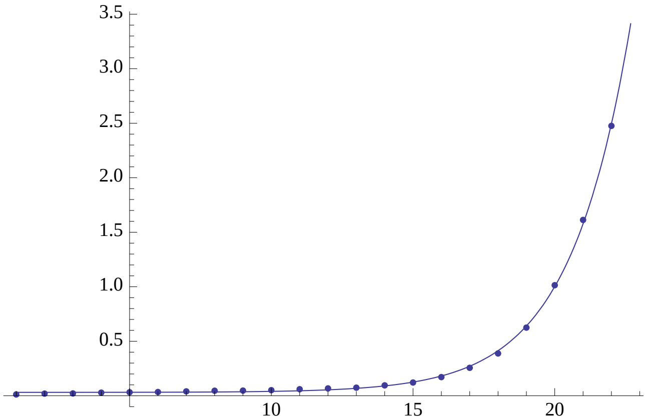

```
F34exp = NonlinearModelFit[F34data[[1 ;; 22]], b + d0 * a^x, {b, d0, a}, x, MaxIterations → 1000]
```

FittedModel[0.0254402 + 0.0000485877 ≪19≫^x]

```
F34exp["BestFit"]
```

$0.0254402 + 0.0000485877\ 1.64621^{x}$

```
Show[ListPlot[F34data[[1 ;; 22]], PlotRange → All], Plot[F34exp[x], {x, 1, 25}]]
```

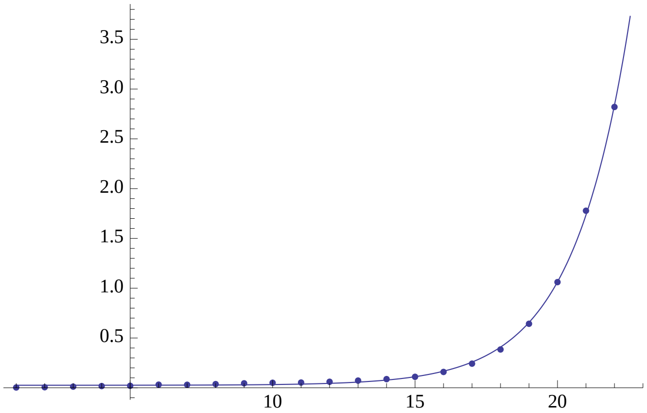

```
F41exp = NonlinearModelFit[F41data[[1 ;; 24]], b + d0 * a^x, {b, d0, a}, x, MaxIterations → 1000]
```

FittedModel[$0.0218628 + 1.48735 \times 10^{-6}$ ≪19≫^x]

```
F41exp["BestFit"]
```

$0.0218628 + 1.48735 \times 10^{-6}\ 1.74076^{x}$

```
Show[ListPlot[F41data[[1 ;; 24]], PlotRange → All], Plot[F41exp[x], {x, 1, 27}]]
```

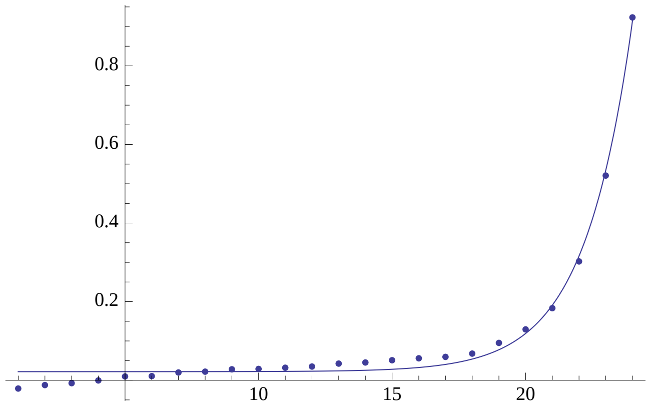


```
F42exp = NonlinearModelFit[F42data[[1 ;; 24]], b + d0 * a^x, {b, d0, a}, x, MaxIterations → 1000]
```

FittedModel[$0.0247966 + 1.13024 \times 10^{-6} \ll 18 \gg^{x}$]

```
F42exp["BestFit"]
```

$0.0247966 + 1.13024 \times 10^{-6}\, 1.77639^{x}$

```
Show[ListPlot[F42data[[1 ;; 24]], PlotRange → All], Plot[F42exp[x], {x, 1, 27}]]
```

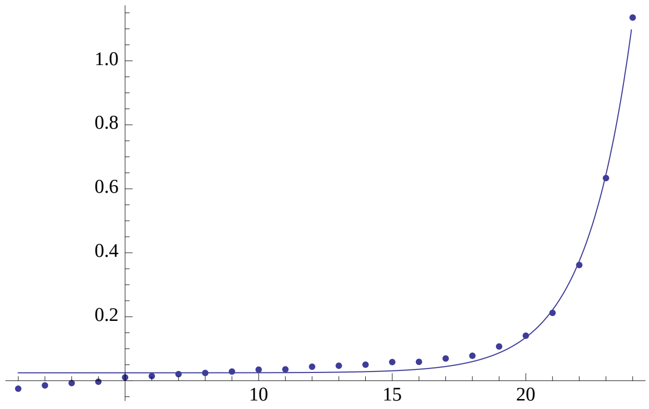


```
F43exp = NonlinearModelFit[F43data[[1 ;; 24]], b + d0 * a^x, {b, d0, a}, x, MaxIterations → 1000]
```

FittedModel[$0.0408012 + 8.62537 \times 10^{-6} \ll 19 \gg^{x}$]

```
F43exp["BestFit"]
```

$0.0408012 + 8.62537 \times 10^{-6}\, 1.62557^{x}$

```
Show[ListPlot[F43data[[1 ;; 24]], PlotRange → All], Plot[F43exp[x], {x, 1, 27}]]
```

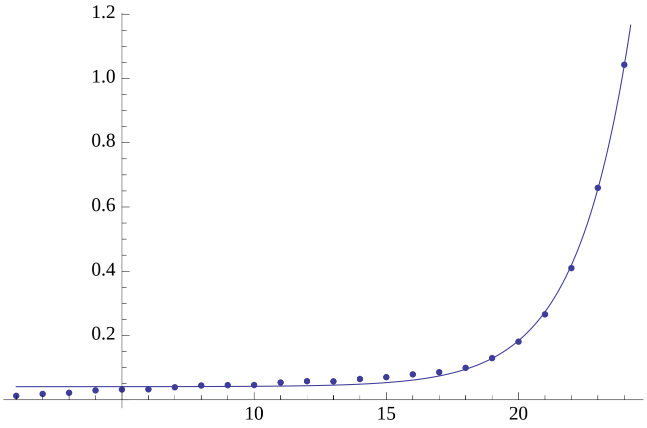


```
F44exp = NonlinearModelFit[F44data[[1 ;; 24]], b + d0 * a^x, {b, d0, a}, x, MaxIterations → 1000]
```

FittedModel[$0.0378632 + 3.7217\times10^{-6} \ll19\gg^{x}$]

```
F44exp["BestFit"]
```

$0.0378632 + 3.7217\times10^{-6}\, 1.69894^{x}$

```
Show[ListPlot[F44data[[1 ;; 24]], PlotRange → All], Plot[F44exp[x], {x, 1, 27}]]
```

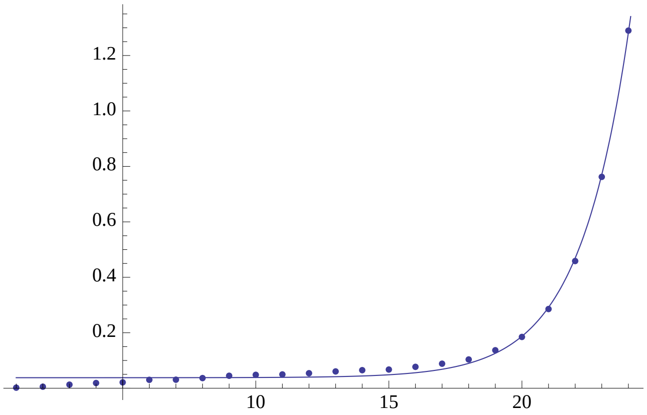


```
F51exp = NonlinearModelFit[F51data[[1 ;; 28]], b + d0 * a^x, {b, d0, a}, x, MaxIterations → 1000]
```

FittedModel[$0.0299306 + 1.10969\times10^{-7} \ll19\gg^{x}$]

```
F51exp["BestFit"]
```

$0.0299306 + 1.10969\times10^{-7}\, 1.80258^{x}$

```
Show[ListPlot[F51data[[1 ;; 28]], PlotRange → All], Plot[F51exp[x], {x, 1, 30}]]
```

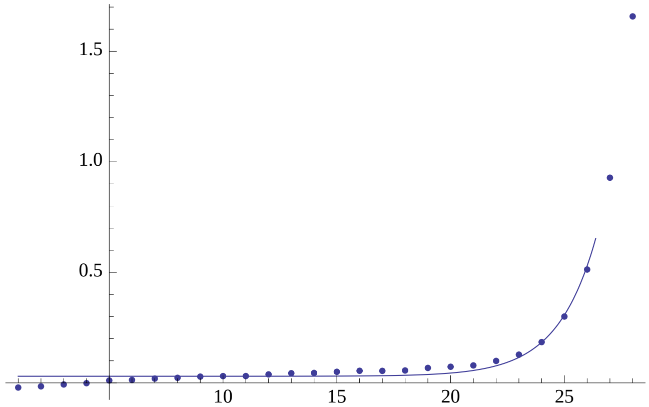


```
F52exp = NonlinearModelFit[F52data[[1 ;; 28]], b + d0 * a^x, {b, d0, a}, x, MaxIterations → 1000]
```

FittedModel[$0.0301569 + 1.09252\times10^{-7}$ ≪19≫^{x}]

```
F52exp["BestFit"]
```

$0.0301569 + 1.09252\times10^{-7}\ 1.80832^{x}$

```
Show[ListPlot[F52data[[1 ;; 28]], PlotRange → All], Plot[F52exp[x], {x, 1, 31}]]
```

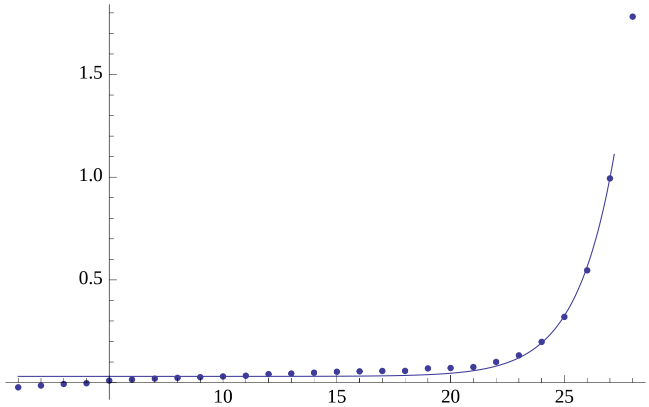


```
F53exp = NonlinearModelFit[F53data[[1 ;; 28]], b + d0 * a^x, {b, d0, a}, x, MaxIterations → 1000]
```

FittedModel[$0.0449902 + 2.02889\times10^{-6}$ ≪19≫^{x}]

```
F53exp["BestFit"]
```

$0.0449902 + 2.02889\times10^{-6}\ 1.62525^{x}$

```
Show[ListPlot[F53data[[1 ;; 28]], PlotRange → All], Plot[F53exp[x], {x, 1, 31}]]
```

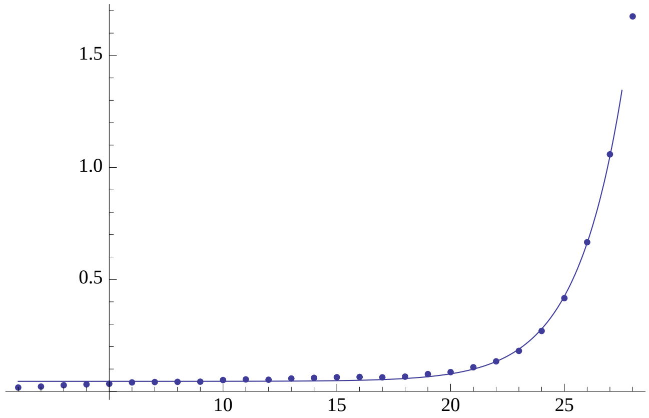


```
F54exp = NonlinearModelFit[F54data[[1 ;; 28]], b + d0 * a^x, {b, d0, a}, x, MaxIterations → 1000]
```

FittedModel[$0.0398918 + 6.62153 \times 10^{-7} \ll 19 \gg^{x}$]

```
F54exp["BestFit"]
```

$0.0398918 + 6.62153 \times 10^{-7}\, 1.68975^{x}$

```
Show[ListPlot[F54data[[1 ;; 28]], PlotRange → All], Plot[F54exp[x], {x, 1, 31}]]
```

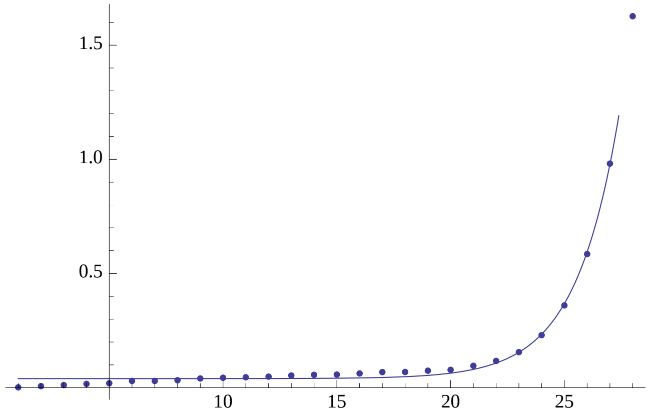


```
F61exp = NonlinearModelFit[F61data[[1 ;; 31]], b + d0 * a^x, {b, d0, a}, x, MaxIterations → 1000]
```

FittedModel[$0.0347957 + 2.00307 \times 10^{-8} \ll 19 \gg^{x}$]

```
F61exp["BestFit"]
```

$0.0347957 + 2.00307 \times 10^{-8}\, 1.78157^{x}$

Show[ListPlot[F61data[[1 ;; 31]], PlotRange → All], Plot[F61exp[x], {x, 1, 35}]]

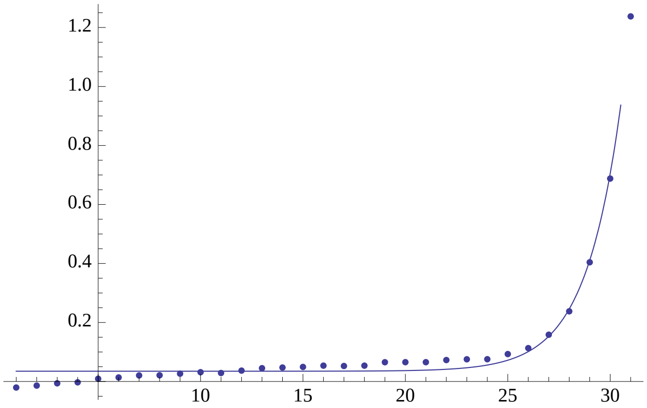

F62exp = NonlinearModelFit[F62data[[1 ;; 31]], b + d0 * a^x, {b, d0, a}, x, MaxIterations → 1000]

FittedModel[$0.037815 + 1.88923\times10^{-8}$ ≪19≫^{x}]

F62exp["BestFit"]

$0.037815 + 1.88923 \times 10^{-8}\, 1.79139^{x}$

Show[ListPlot[F62data[[1 ;; 31]], PlotRange → All], Plot[F62exp[x], {x, 1, 35}]]

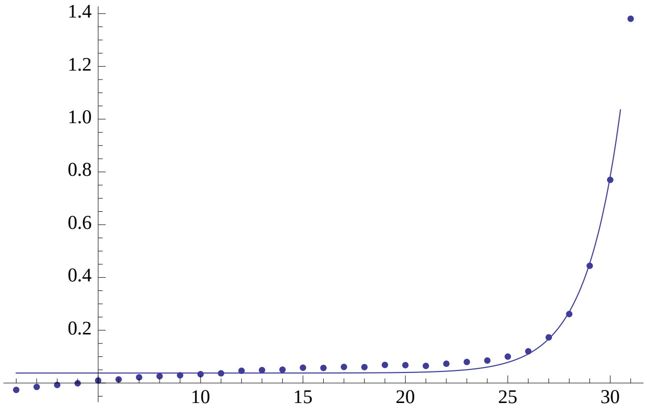

F63exp = NonlinearModelFit[F63data[[1 ;; 31]], b + d0 * a^x, {b, d0, a}, x, MaxIterations → 1000]

FittedModel[$0.0497411 + 3.7214\times10^{-7}$ ≪19≫^{x}]

F63exp["BestFit"]

$0.0497411 + 3.7214 \times 10^{-7}\, 1.61823^{x}$

```
Show[ListPlot[F63data[[1 ;; 31]], PlotRange → All], Plot[F63exp[x], {x, 1, 35}]]
```

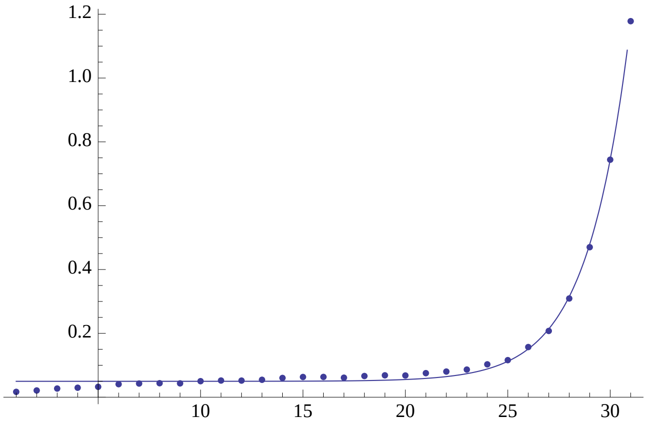


```
F64exp = NonlinearModelFit[F64data[[1 ;; 31]], b + d0 * a^x, {b, d0, a}, x, MaxIterations → 1000]
```

FittedModel[$0.0488819 + 1.00182\times10^{-7}\ll19\gg^{x}$]

```
F64exp["BestFit"]
```

$0.0488819 + 1.00182\times10^{-7}\,1.69067^{x}$

```
Show[ListPlot[F64data[[1 ;; 31]], PlotRange → All], Plot[F64exp[x], {x, 1, 35}]]
```

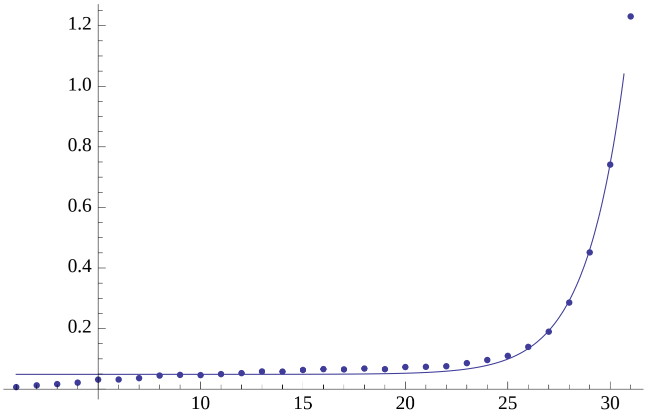


```
F71exp = NonlinearModelFit[F71data[[1 ;; 39]], b + d0 * a^x, {b, d0, a}, x, MaxIterations → 1000]
```

FittedModel[$0.0466201 + 2.5351\times10^{-10}\ll19\gg^{x}$]

```
F71exp["BestFit"]
```

$0.0466201 + 2.5351\times10^{-10}\,1.76705^{x}$

```
Show[ListPlot[F71data[[1 ;; 39]], PlotRange → All], Plot[F71exp[x], {x, 1, 46}]]
```

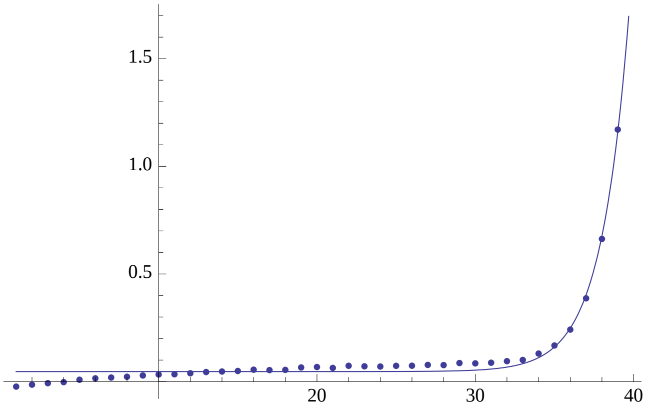


```
F72exp = NonlinearModelFit[F72data[[1 ;; 39]], b + d0 * a^x, {b, d0, a}, x, MaxIterations → 1000]
```

FittedModel[$0.0495575 + 2.2596\times10^{-10} \ll 18 \gg^{x}$]

```
F72exp["BestFit"]
```

$0.0495575 + 2.2596\times10^{-10}\, 1.77982^{x}$

```
Show[ListPlot[F72data[[1 ;; 39]], PlotRange → All], Plot[F72exp[x], {x, 1, 46}]]
```

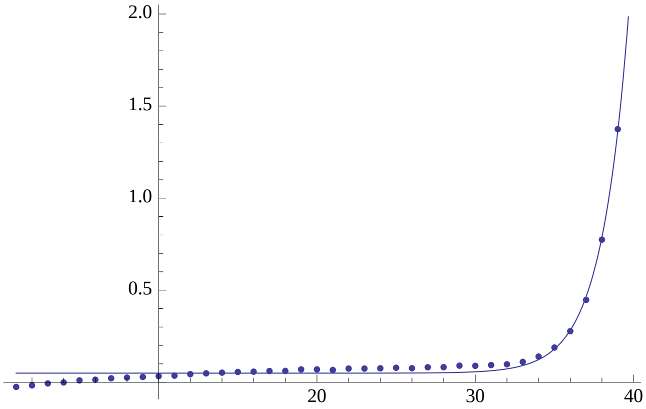


```
F73exp = NonlinearModelFit[F73data[[1 ;; 39]], b + d0 * a^x, {b, d0, a}, x, MaxIterations → 1000]
```

FittedModel[$0.0607448 + 2.18005\times10^{-8} \ll 19 \gg^{x}$]

```
F73exp["BestFit"]
```

$0.0607448 + 2.18005\times10^{-8}\, 1.56834^{x}$

```
Show[ListPlot[F73data[[1 ;; 39]], PlotRange → All], Plot[F73exp[x], {x, 1, 46}]]
```

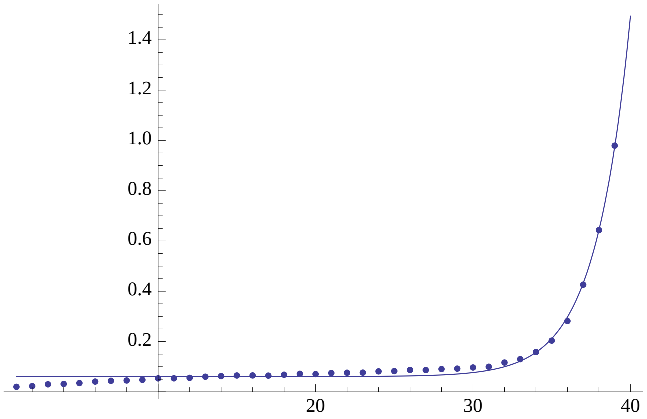


```
F74exp = NonlinearModelFit[F74data[[1 ;; 39]], b + d0 * a^x, {b, d0, a}, x, MaxIterations → 1000]
```

FittedModel[$0.0568989 + 4.85376\times10^{-9} \ll 19 \gg^{x}$]

```
F74exp["BestFit"]
```

$0.0568989 + 4.85376\times10^{-9}\,1.64168^{x}$

```
Show[ListPlot[F74data[[1 ;; 39]], PlotRange → All], Plot[F74exp[x], {x, 1, 46}]]
```

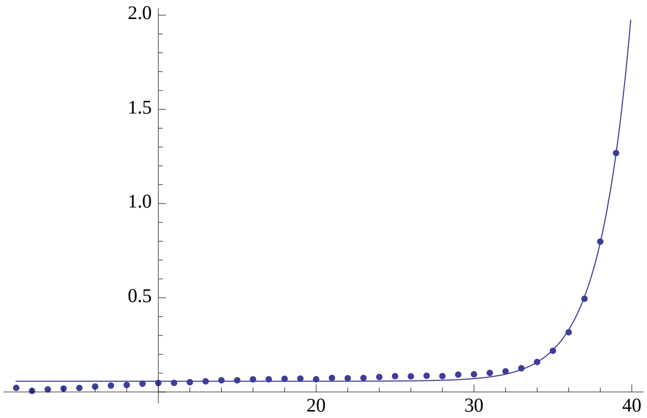

Supplement: Dataset S4 — Data resulting from fitting raw qPCR data with an exponential model. D0 values obtained were used in generating figure 3. (1.38 MB PDF) [file pone.0012355.s007.pdf]
